# Triple Regioselective Functionalization of Cationic [4]Helicenes via Iridium‐Catalyzed Borylation and Suzuki Cross‐Coupling Reactivity

**DOI:** 10.1002/chem.202201853

**Published:** 2022-08-18

**Authors:** Lucas Frédéric, Bibiana Fabri, Laure Guénée, Francesco Zinna, Lorenzo Di Bari, Jérôme Lacour

**Affiliations:** ^1^ Department of Organic Chemistry University of Geneva Quai Ernest Ansermet 30 1211 Geneva 4 Switzerland; ^2^ Laboratoire de Cristallographie University of Geneva Quai Ernest Ansermet 24 1211 Geneva 4 Switzerland; ^3^ Dipartimento di Chimica e Chimica Industriale University of Pisa Via G. Moruzzi 13 Pisa Italy

**Keywords:** cationic helicenes, chiroptical spectroscopy, dyes and fluorophores, Ir-catalyzed borylation •late stage functionalization

## Abstract

In essentially one‐pot, using Ir‐ and Pd‐catalysis, tris(arene)‐functionalized cationic [4]helicenes are synthesized with full regioselectivity and enantiospecificity starting from a trivial precursor (17 examples). This poly‐addition of aryl groups improves key optical properties, that is, fluorescence quantum yields and lifetimes. Electronic circular dichroism and circularly polarized luminescence signatures are observed up to the far‐red domain, in particular with additional arenes prone to aggregation.

## Graphical abstract

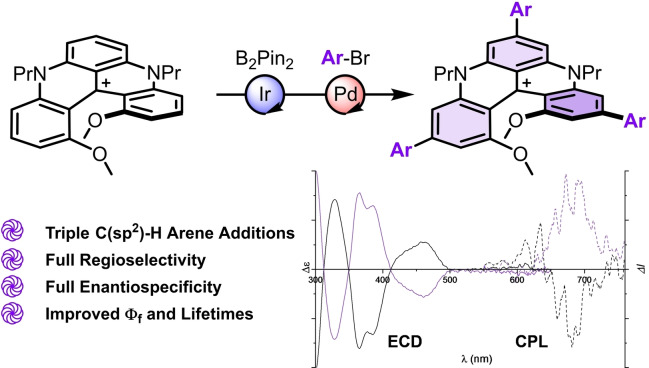

## Introduction

**Scheme 1:**
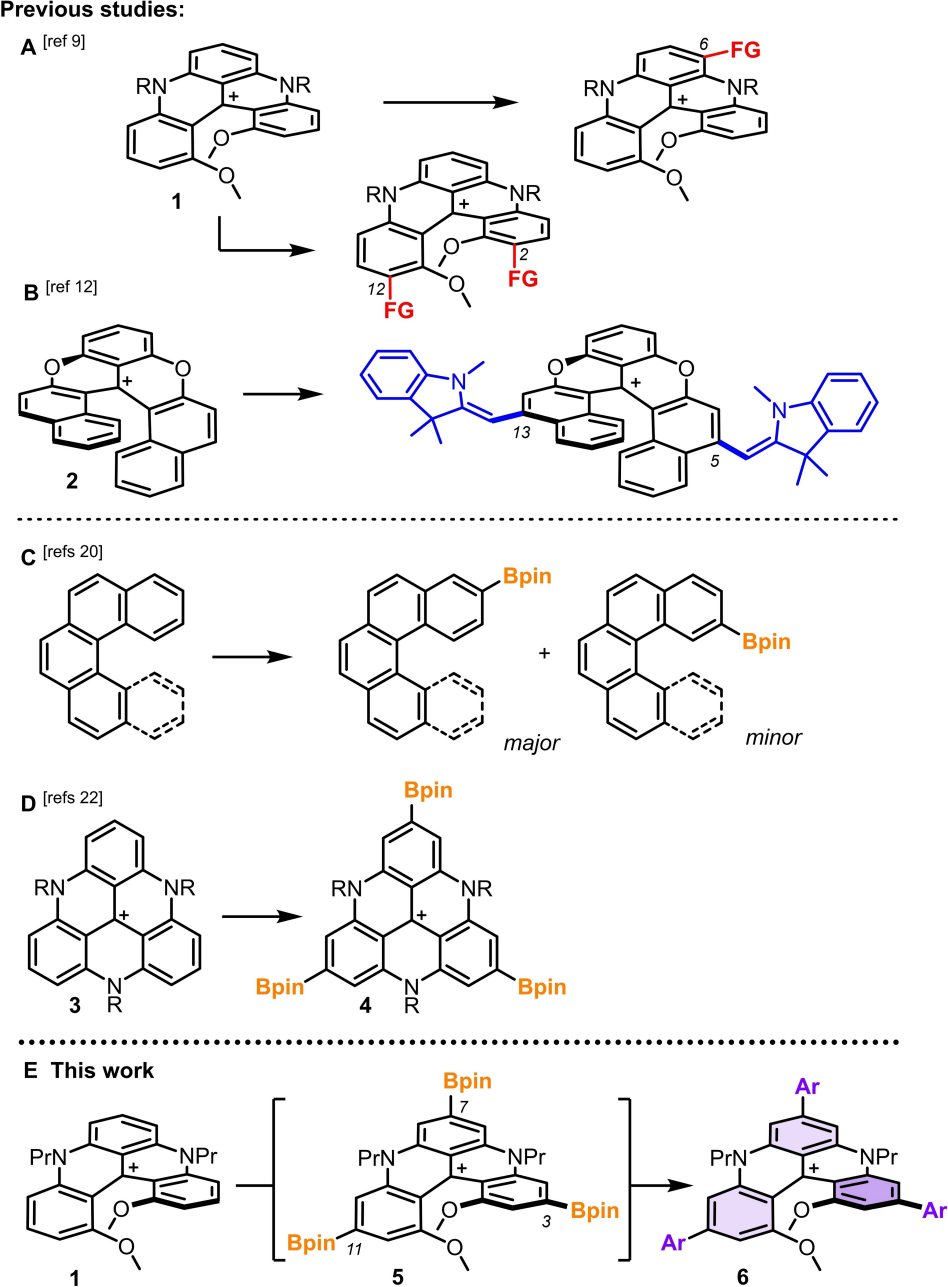
Late‐stage functionalization of polyaromatics. Selected examples. A: cationic [4]helicene 1 by S_*E*_Ar mechanisms. B: Dioxa [6]helicene 2 by oxidative nucleophilic coupling. C: Ir‐catalyzed borylation of carbohelicenes, D: of cationic triaza triangulenes, E: of racemic and enantiopure DMQA 1 (*M* enantiomer shown) and subsequent cross‐coupling derivatizations. FG: functional group. Ar: aryl.

Organic carbohelicenes, defined as a succession of *ortho*‐fused aromatic rings, are an important class of chiral molecules that owe their asymmetry to the steric repulsion occurring between terminal substituents or benzene rings.^[1]^ These conjugated helical moieties permit applications in many domains from asymmetric catalysis and organic electronics, to supramolecular and biological chemistry.[^1f^, ^2^] These compounds display absorption and emission properties mainly in the UV‐visible region together with the corresponding electronic circular dichroism (ECD) and circularly polarized luminescence (CPL).[^3^, ^4^] For applications targeting red to near infrared spectral regions, heterohelicenes or additions of organic or organometallic chromophores can be considered.^[5]^ Cationic [n]helicenes, n=4, 5 and 6, which beneficiate from the extended delocalization provided by the triarylcarbenium framework, are interesting alternatives.^[6]^ For instance, [4]helicene **1** displays absorption and emission centered at 614 and 667 nm respectively (acetonitrile).[^7^, ^8^] Dimethoxyquinacridinium moieties (DMQA) of this type are readily prepared in a two‐step process from 1,3‐dimethoxybenzene and simply resolved on gram scale into single enantiomers of (*M*) (left‐handed) and (*P*) (right‐handed) configurations by a chiral auxiliary approach.^[9]^ Previously, to tune electronic and (chir)optical properties, and develop further applications, late‐stage functionalization (LSF) strategies have been engineered to introduce series of functional groups on the outer rim of the cationic [n]helicenes. Compound **1**, remarkably nucleophilic, can be substituted under S_*E*_Ar reactivity at either positions 6 or 2,12 of the aromatic skeleton, depending on the experimental conditions (Scheme 1, **A**).^[10]^ As a consequence, absorption and emission spectra are blue‐ or red‐shifted (up to the far red region) upon introduction of electron‐withdrawing (EWGs) and electron‐donating groups (EDGs), respectively; luminescence is favored mainly in the presence of electron‐poor substituents. Such modifications are favorable for the development of pH‐sensitive probes, radical intermediate stabilization or biological assay.^[11]^ In terms of regioselectivity, a different pattern can be obtained in the cationic [6]helicene series specifically, using electrophilic dioxa **2** in particular (Scheme 1, **B**). In this case, functionalization by oxidative additions occurs with enamines or indolenines as nucleophiles in favor of mono or double substitutions at positions 5,13.^[12]^ Strongly red‐shifted absorption and emission are noticed in the near infrared allied with a cyanine‐character for some of the dyes.^[13]^ In this context that shows the importance of functionalizations *para* to the formal carbocationic center, care was taken to devise a LSF strategy to achieve such a regioselectivity by direct C(sp^2^)−H functionalization of the most classical DMQA derivatives **1**.^[14]^

In fact, during the last two decades, arene functionalization of unactivated C−H bonds has generated much interest, with new synthetic strategies and methodologies regularly reported.^[15]^ In this domain, Ir‐catalyzed borylation has received a particular attention.^[16]^ Typically, in the presence of classical ligands such as 4,4’‐di‐*tert*‐butyl‐2,2′‐bipyridine and 3,4,7,8‐tetramethyl‐1,10‐phenanthroline (tmphen),^[17]^ regioselectivity is determined by steric considerations. In case of charged substrates, and cationic derivatives in particular, optimized anionic ligands can be considered to control selectivity based primarily on ion‐pairing effects.^[18]^ Specific conditions can also be designed for *ortho*‐ and, to a lesser extent, *meta‐* and *para*‐borylations.[^18^, ^19^] With [4] and [5]helicenes as reactants, borylation was previously observed at terminal ends with regioselectivity ratios varying from 3 : 1 to 8 : 1 (Scheme 1, **C**).^[20]^ Favorably, single regioisomeric functionalization was observed in the case of an aza[7]helicene skeleton by Maeda and Ema.^[21]^ Finally, of importance of the current study, Matsuda and collaborators have studied the reactivity of cationic triazatriangulenes **3** that proceeds with borylation of each of the benzene rings to form *C*_3_‐symmetric tris(Bpin) **4** as major product (Scheme 1, **D**).^[22]^ With these precedents in mind, the investigation of Ir‐catalyzed borylation on cationic DMQA **1** was started. Herein, we report on the direct triple *para*‐functionalization of the [4]helicene skeleton to reach *C*_2_‐symmetric intermediate **5** in a single step. Direct conversion of this moisture‐sensitive molecule was found to be necessary and it was realized by Suzuki−Miyaura cross‐coupling (SMCC, Scheme 1, **E**). Introduction of a variety of aryl groups with EDGs and EWGs was thus achieved leading to the formation of tris(arene) derivatives **6**. Full retention of configuration was achieved in the (*M*)‐ and (*P*)‐series (enantiospecificity, e.s. >99 %). The investigation of their optical properties shows moderate effects on the absorption features (λ_max_ 608–632 nm) but a significant increase of fluorescence quantum yields (Φ_f_, 14→37 %) and fluorescence lifetimes (5.6→10.4 ns, CH_3_CN). All *para*‐functionalized tris(arene) derivatives display ECD signatures from the UV to the far‐red domain. Moderate CPL signals (*g*_lum_±1 ⋅ 10^−4^) are observed to the exception of tris(tetraphenylethene) [4]helicene; its aggregation induces an upsurge of *g*_abs_ value at low energy transitions and a correlated increase of |*g*_lum_| up to 2.4 ⋅ 10^−3^.

## Results and Discussion

As mentioned above, regioselective LSF of cationic [4] and [6]helicenes are particularly useful strategies to manipulate electronic and optical properties and open avenues for new applications. In the case of compound **1**, despite efforts highlighted in Scheme 1 (section **A**), late‐stage introduction of substituents *para* to the formal positive charge, in positions 3, 7 or 11 respectively, had not yet been achieved. It was thus our intention to reach such a regiochemistry of **1** by LSF and extend the scope of further derivatizations. In view of the results reported by Matsuda et al. with related cationic triangulene **4** (section **D**), the Ir‐catalyzed borylation was an obvious choice for the targeted regioselectivity. Since the three *para* positions of **1** present similar steric and electronic environments, chemo and regioselective mono or bis‐borylation were deemed unlikely to occur. To simplify characterizations of the crude reaction mixtures and isolation of products, it was selected to pursue the direct triple Bpin functionalization of **1**.

First attempts of borylation using B_2_pin_2_ (6 equiv) and the catalytic combination of [Ir(cod)OMe]_2_ and tmphen, 20 and 40 mol% respectively, gave promising results in THF at 80 °C. Targeted tris‐borylated adduct **5** was detected as a predominant component by mass spectrometry (MS). However, its isolation became an immediate issue as any attempt to separate **5** from crude media resulted in rapid decompositions. Currently, and unlike related triangulene **4**,^[22a]^ tris‐borylated adduct **5** is found to be sensitive to moisture to the extent of preventing any characterization and (chir)optical studies. Still, under strict anhydrous Schlenk‐like conditions, **5** remains stable in the reaction mixture (>7 days) and can thus be treated as a regular synthetic intermediate for further functionalization. Finally, with a longer reaction time (6→20 h), the amount of Iridium complex and ligand could be reduced to 5 and 10 mol% (see Graphic S1 in Supporting Information).

With tris‐borylated DMQA intermediate **5** in hand, and knowing that extended aromatic arms often promote improved optical properties and supramolecular assemblies,[^22b^, ^23^] Suzuki−Miyaura cross‐coupling reactions were selected for derivatization. Choosing this type of boronic ester transformation presented however a challenge due to the water sensitivity of **5** (see above). In fact, SMCC are often performed in basic aqueous conditions to promote the formation of borate anions that facilitate the mechanistic trans‐metalation step.^[24]^ In this context, working in total exclusion of water was a significant experimental constraint. Care was taken to select a variety of anhydrous bases in search for optimized conditions using Pd(PPh_3_)_4_ as (first‐tested) metal catalyst and 1‐bromo‐4‐fluorobenzene in excess as Ar−Br reagent.

The results are detailed in Table 1 using electrospray ionization low resolution mass spectroscopy analysis, that is, ESI‐LRMS, for the reaction monitoring. Such a method was efficient to screen rapidly and efficiently the progression of the cross‐coupling steps towards tris(arene) **6 b**. In fact, due to the cationic nature of all species of interest, tris(Bpin) **5**, tris(arene) **6 b**, but also mono‐ **7 b** and bis‐arylated **8 b**, MS‐monitoring afforded a direct access to the distribution of the different species in the reaction mixture.^[25]^ This qualitative analysis gave surprisingly reliable results. In fact, all reactions displaying an intense predominant peak at m/z 695 (exact mass of **6 b^+^**) corresponded to the cleanest experiments with the highest isolated yields of tris(arene) **6 b**. With KF and Et_3_N as bases, SMCC reactivity was simply not observed under anhydrous conditions (entries 1 and 2). Cs_2_CO_3_ gave first promising results with the presence of mono, bis and tris(arene) products in the crude mixture; mono **7 b** being however the major component indicating a tendency for C‐BPin reductions in place of C−Ar bond formation (entry 3). Low conversion of **5** was noticed with tripotassium phosphate in favor of BisAr **8 b** this time (entry 4). With NaOH (solid) used as base (entry 5), only a modest amount of tris(arene) **6 b** was afforded. Two other hydroxyl bases, LiOH and KOH (entries 6 and 7), induced a much higher reactivity with a clear preference for the potassium mediated reaction that permits the formation of tris(arene) **6 b** in good and reproducible yields. NMR spectroscopic analysis confirmed readily the expected *para*‐regioselectivity favoring a *C*_2_‐symmetric geometry. Yet, the presence of triphenylphosphine oxide in the medium rendered cumbersome the isolation by chromatography of **6 b**, and of all other derivatives **6** for that matter. Other combinations of palladium metal sources and phosphine ligands were tested and the results are displayed in Graphic S2. In short, Pd(OAc)_2_ and dppf (1,1′‐Bis(diphenylphosphino)ferrocene, 60 mol% each) is the most efficient combination to provide phosphine oxide‐free tris(arene) **6 b**.^[26]^

**Table 1:** Suzuki−Miyaura Cross Couplings: Base optimization.^[a]^

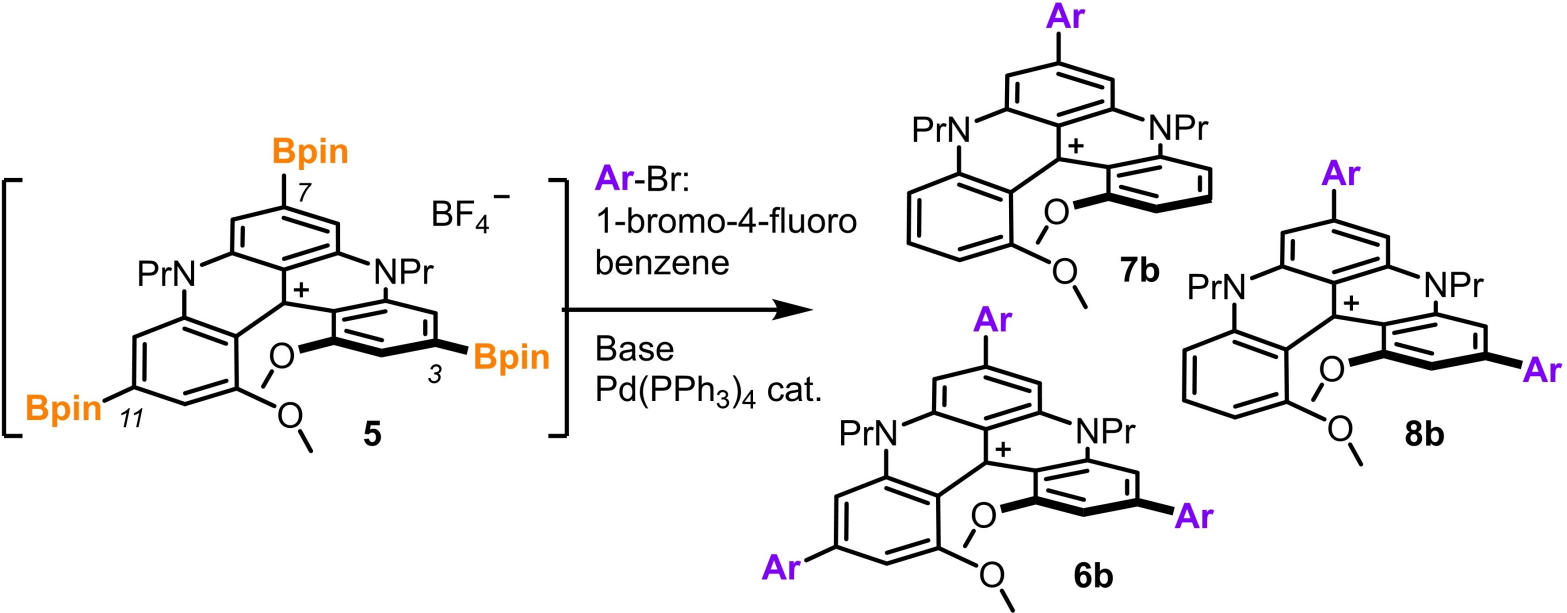						
		Distribution^[b]^				
Entry	Base	**5**	Mono **7 b**	Bis **8 b**	Tris **6 b**	Degr.^[c]^
1	KF	71	0	0	0	29
2	NEt_3_	70	0	0	0	30
3	Cs_2_CO_3_	14	39	28	11	8
4	K_3_PO_4_	61	0	13	0	26
6	NaOH	74	0	0	25	2
5	LiOH	0	8	27	33	33
7	KOH	0	7	10	81	2

[a] Conditions: Base (10 equiv), Ar−Br (10 equiv), Pd(PPh_3_)_4_ (30 mol%), THF, 80 °C. [b] Percentages determined by MS analysis; regiochemistry for mono **7 b** and bis(arene) **8 b** is arbitrary. [c] Degradation products correspond to hydrolyzed boronic esters and starting DMQA **1**.

Finally, as a rule, NMR studies of crude reaction mixtures revealed the presence of minor amounts of neutral *leuco* adducts. Derivative **6‐H** [Equation (1)], and corresponding mono **7‐H** and bis(arene) **8‐H** homologues, can be readily identified by singlet ^1^H NMR signals in the δ 4.5–5 ppm region.[^27^, ^28^] Most probably, these moieties result from the reactivity of cationic precursors with hydride species present in the crude media as the result of the (reductive) Ir‐catalyzed borylation step.^[29]^ With hindsight, the presence of compounds **6‐H** was seen as an advantage to help isolate targeted products **6**. In fact, neutral derivatives **6‐H** are readily purified by chromatography unlike their cationic analogues **6** (see Figure S96‐97 for **6 d‐H**). In addition, transformation of cationic **6** into **6‐H** can be achieved by simple treatment with NaBH_4_ or NaBH_3_CN [Equation (1)]. The reverse oxidation of **6‐H** to **6** happens spontaneously in air over one or two days but is best achieved under visible‐light irradiation (Osram Ultra Vitalux 300 W, 1–2 h) in dichloromethane in presence of aqueous NaBF_4_ (>180 equiv).

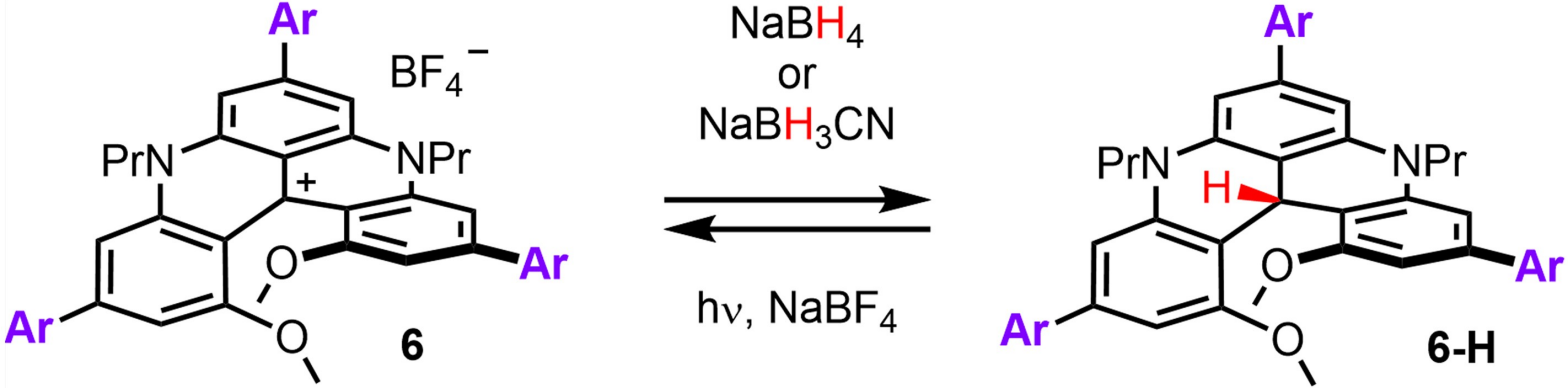




**Figure 1:**
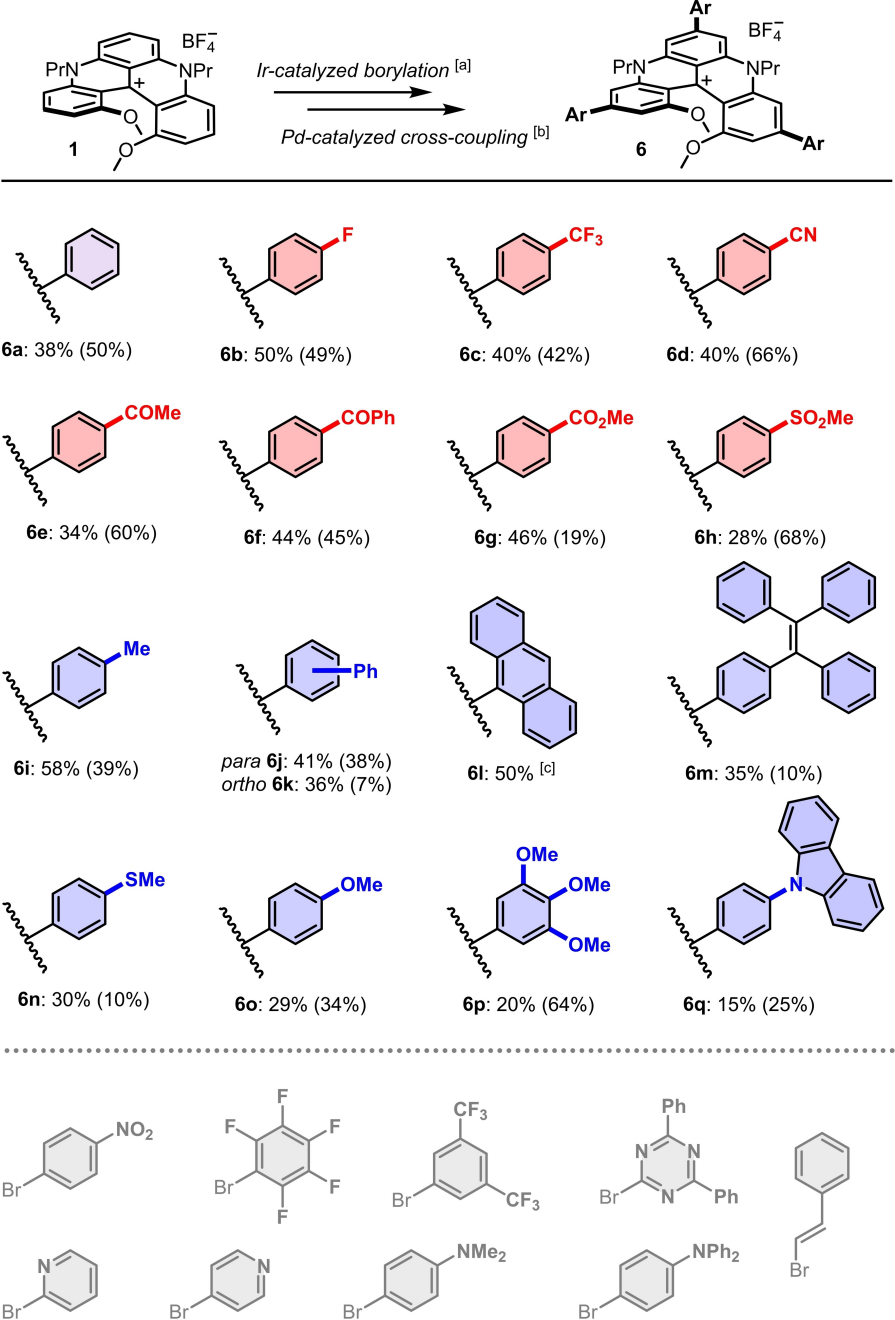
Scope of triple borylations and subsequent triple Suzuki−Miyaura cross couplings. Isolated yields for BF_4_^−^ salts in racemic series and, in parenthesis, for (*M*)‐ and (*P*)‐enantiomers (average of both enantiospecific reactions). EWGs and EDGs in red and blue‐coded series, respectively. With aryl bromides in grey color, a lack of reactivity or purification issues were observed. [a] [Ir(cod)OMe]_2_ (20 mol%), tmphen (40 mol%), B_2_pin_2_ (6 equiv). [b] Pd(OAc)_2_ (60 mol%), dppf (60 mol%), ArBr (9 equiv), KOH (9 equiv). [c] SMCC reaction conditions: Pd(PPh_3_)_4_ (80 mol%), Cs_2_CO_3_ (10 equiv), 9‐Br‐anthracene (10 equiv), THF:1,4‐dioxane (2 : 1). For this single example, (*M*)‐**6 l** and (*P*)‐**6 l** products were obtained from the racemate via a chiral stationary phase HPLC resolution.

Once the conditions for the triple borylation and subsequent triple Suzuki−Miyaura cross coupling had been optimized, the tandem protocol was extended to a variety of aryl bromide reagents carrying either EWGs or EDGs (Figure 1, 17 examples). The yields are reported for isolated BF_4_^−^ salts in the racemic series and, in parenthesis, for the preparation of compounds of (*M*)‐ and (*P*)‐configurations (average of both enantiospecific reactions). Care was taken to verify the excellent enantiospecificity of the reaction (e.s. >99 %). For instance, starting from (*M*)‐**1** in 99.2 % e.e., product **6 j** was obtained with an essentially similar enantiomeric purity of 98.8 % (Figures S3‐S4).^[30]^ Conservation of the absolute configuration is further established with the X‐ray diffraction analyses of (*P*)‐**6 e** and (*M*)‐**6 o** (see below). Of interest, better overall yields, up to 68 %, are obtained with coupling reagents carrying EWGs (red color) that give compounds **6 b** to **6 h**. To the exception of **6 g**, results in the enantiopure series match or improve that of the racemic. Overall, slightly lower yields were obtained with EDGs (blue color) and, on case by case, strong variations were noticed between *rac* and (*M*)‐/(*P*)‐syntheses. For instance, with pyrogallol‐derived *rac*‐**6 p**, a lower yield was obtained due to an unexpectedly high solubility in Et_2_O that limited the final purification by precipitation. With hindsight, this pitfall was corrected for the making of (*M*)‐ or (*P*)‐**6 p**. With poorly soluble 9‐(4‐bromophenyl)carbazole, cross‐coupling reactivity was hampered and a larger proportion of corresponding mono **7 q** and bis **8 q** adducts was obtained leading to an overall lower yield of **6 q**. Lack of reactivity or purification issues limited the formation of products **6** with aryl bromides listed at the bottom of Figure 1.

Additionally, it was decided to test briefly whether previously‐developed LSF protocols (Scheme 1, **A**) could still be applied on derivatives of type **6**, despite the steric encumbrance generated by the additional aryl groups at positions 3, 7 and 11. Using **6 b** as model substrate, a Vilsmeier−Haack reaction was attempted; this formylation procedure being possible on unhindered cationic helicene **1** (82 % yield).[^10b^, ^31^] In this case, no reaction happened. Similarly, attempts at benzoylation of **6 b** in positions 2 and 12 under strongly acidic media conditions failed in this instance.^[10a]^ However and satisfactorily, the nitration of **6 b** occurred with ease in most activated position 6 to afford adduct **9** in 65 % isolated yield (regiochemistry by detailed 2D NMR analysis, see Figures S102‐105, [Equation 2]).

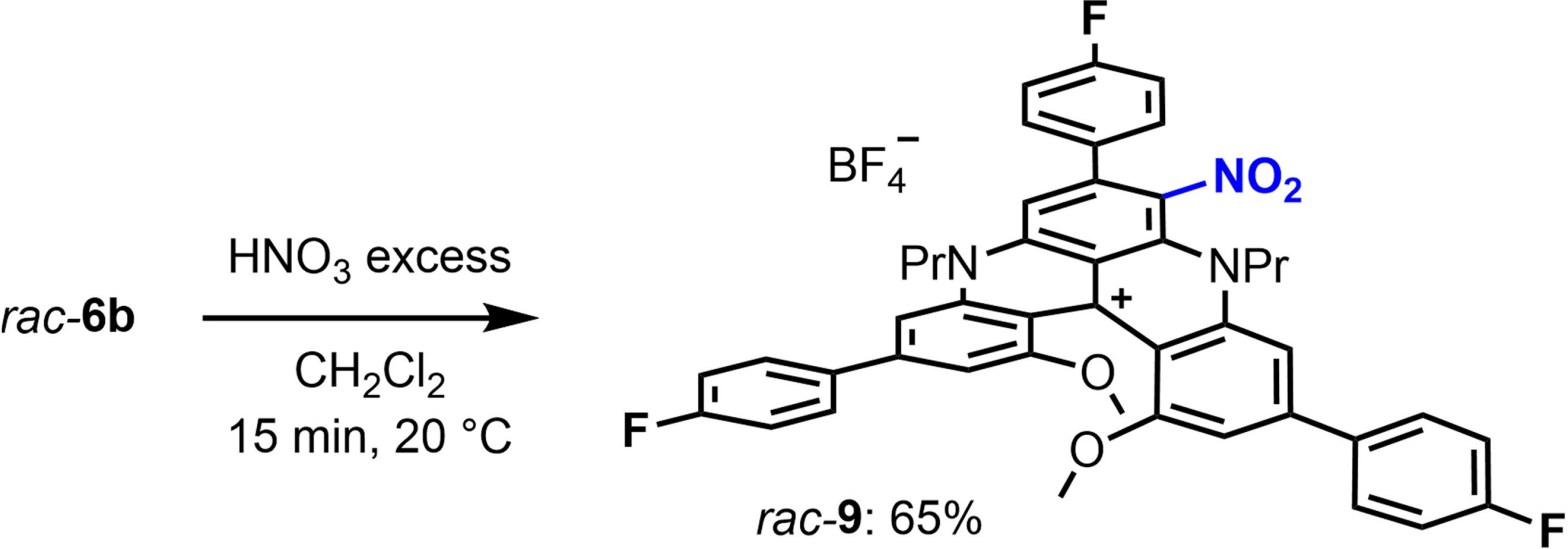




**Figure 2:**
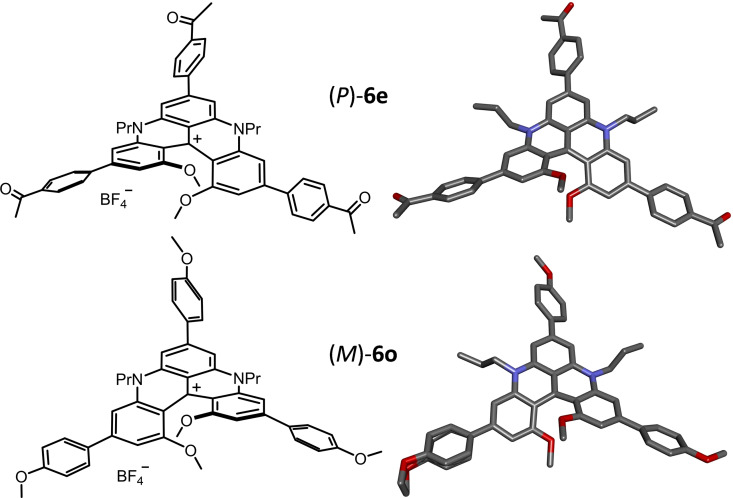
Chemical drawing of (*P*)‐**6 e** and (*M*)‐**6 o** and X‐ray structures of cationic helicenes; hydrogen atoms, BF_4_^−^ counter‐ion and solvate molecules are omitted for clarity purpose.

Single crystals suitable for X‐ray diffraction were obtained for (*P*)‐**6 e** and (*M*)‐**6 o** in two different space groups, orthorhombic *P*2_1_2_1_2_1_ and hexagonal *P*6_5_ respectively (Figure 2).^[32]^ Structurally, the two compounds present similar molecular geometries regardless of the electron‐withdrawing (**6 e**, COMe) or donating (**6 o**, OMe) substituents. Small differences can be noticed in comparison with the previously‐reported structure of the unfunctionalized DMQA **1**.^[7b]^ In fact, the measured distances between the two oxygen atoms of the terminal methoxy (positions 1 and 13) are alike, 2.644(3) Å and 2.674(6) Å for (*M*)‐**6 e** and (*P*)‐**6 o** versus 2.675(3) Å for the naked DMQA.^[10b]^ Similarly, the dihedral angles measured between atoms 13, 14, 15, 16 in the inner rim present comparable values, 26°, 24° and 27° for **6 e**, **6 o** and DMQA **1** respectively (See Figure S106). Furthermore, for **6 e** and **6 o**, the COMe and OMe substituents are essentially coplanar to their adjacent aryl moieties (dihedral angle <5°). However, the interplane angles between the core moiety and the added arenes present values of 28°±6° and 38°±1° depending on their positions on the DMQA skeleton. In fact, in these two structures, more planarized geometries are observed for the aryl substituents in positions 3 and 11 than in position 7. In both structures, aryl substituents participate in intermolecular π–π stacking (see Tables S2 and S6 and Figures S108, S109, S112, S113). In **6 e** and **6 o**, aryl substituents in position 7 and adjacent DMQA core ring stack with two neighboring molecules making four interactions per molecule. In **6 e**, aryl groups in positions 3 and 11 participate also in π‐π stacking with one neighboring molecule, leading to 2 additional interactions per molecule. In total, more interactions in **6 e** (6 per molecule) and **6 o** (4 per molecule) occur than in naked DMQA in which only two π‐π interaction/molecule can be found.^[7b]^ Previously, with other functionalized DMQA derivatives (Scheme 1, **A**),^[10b]^ while four intermolecular interactions per molecule were noticed, those ought to be weaker considering greater interplane angles and plane shifts.

**Figure 3:**
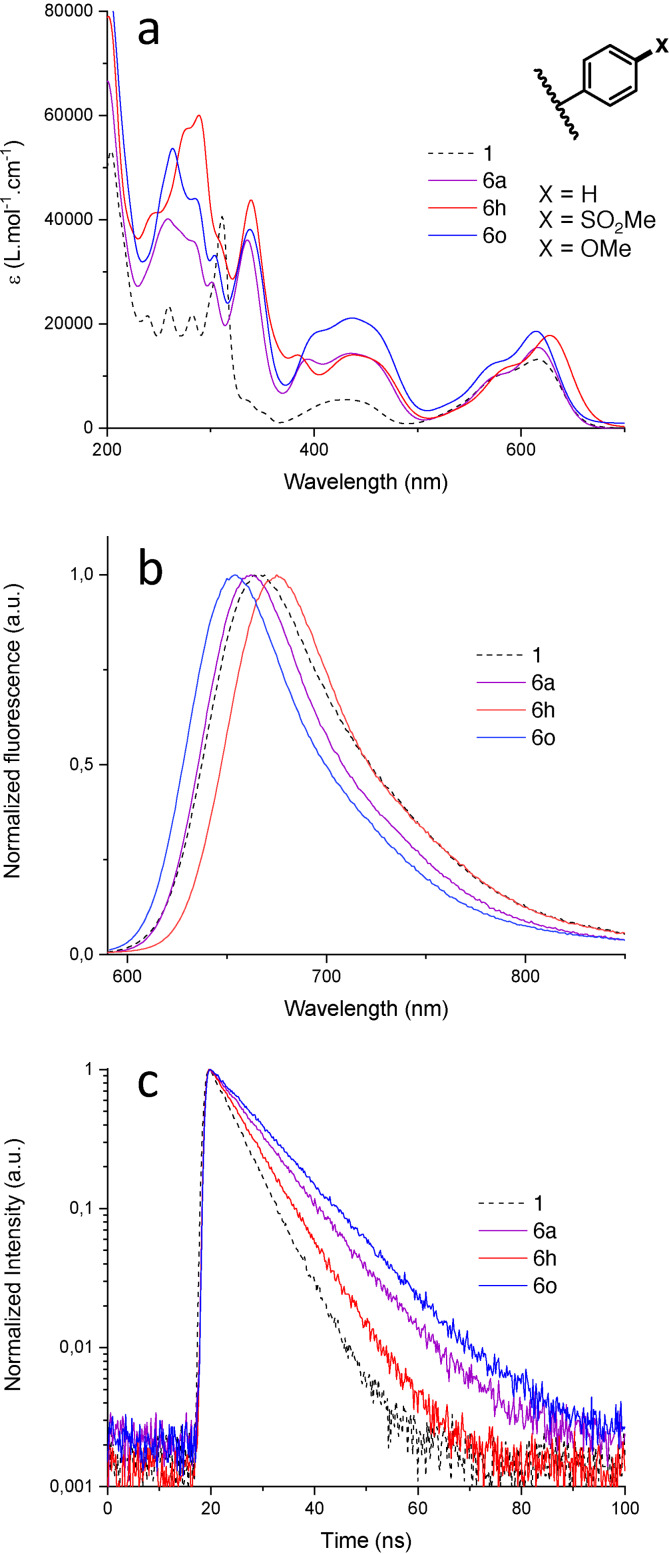
Optical properties of selected derivatives, **1** (dotted black), **6 a** (purple), **6 h** (red) and **6 o** (blue) in acetonitrile. **a**: Absorbance spectra. **b**: Emission spectra. **c**: Fluorescence decay.

Then, with derivatives **6 a** to **6 q** in hand, the influence of the arene substituents was investigated by studying optical and chiroptical properties in priority; all measurements were performed in acetonitrile solutions at room temperature. First, tris(phenyl) derivative **6 a** was compared with unfunctionalized DMQA **1**. Little difference was noticed in the lowest energy absorption as the first absorption band presents no shift and only a slight increase of molar extinction coefficient (ϵ, 13140 M^−1^ ⋅ cm^−1^→15450 M^−1^ ⋅ cm^−1^, Figure 3a). On the other hand, at higher energy, the band centered at 440 nm is greatly increased and also, in the UV domain, a strong impact is observed as the shape of the absorption spectrum changes entirely. These results tend to indicate that simple phenyl rings have little impact on the HOMO‐LUMO transition, which governs the first band of the absorption spectrum, whereas their influence is more marked at higher energy. In presence of substituents on the newly added aryl moieties, compounds **6 b** to **6 q**, only small bathochromic and hypsochromic shifts are observed for EWGs and EDGs respectively. In fact, the biggest deviations are noticed with *p*‐SO_2_Me (**6 h**) and *p*‐OMe (**6 o**) groups but are only modest – up 17 nm (161 cm^−1^) and down 6 nm (438 cm^−1^) respectively, as compared to **6 a** (Figure 3a). In term of molar absorption coefficient, a clear pattern cannot be found for the first absorption band (Table 3). For the second band, centered at 440 nm, EDGs increase the ϵ values while EWGs have little or no influence (vs. **6 a**).

Luminescence was then investigated and, unsurprisingly, **6 a** and DMQA display similar emission maxima (Figure 3b); full‐width half‐maximum is slightly smaller for **6 a** (74 nm) versus **1** (86 nm). Red and blue shifts are observed for the emission maxima of compounds with EWGs (**6 b**‐**6 h**) and EDGs (**6 i**‐**6 q**), as expected from absorbance data. Importantly, in most cases an increase of fluorescence quantum yield (Φ_f_) is measured upon substitution. Anthracene derivative **6 l** is an exception with a Φ_f_ value of 12 % in the same range as simple DMQA (14 %). In comparison to EDGs derivatives, the lower quantum yield in presence of EWGs can be explained by an increase of non‐radiative decay rate constant (k_nr_, Table 2). With a *p*‐OMe group as substituent, Φ_f_ reaches 37 % (**6 o**). These results match effectively fluorescence lifetime measurements as each fluorescence quantum yield increase is reflected by a fluorescence lifetime augmentation (longer decay); the longest lifetime τ reaches 10.4 ns for **6 o**. Overall, the addition of functionalized arene groups *para* to the formal positive charge, irrespective of their electronic nature, favors the radiative decay of the lowest excited state as compared to simple DMQA **1**. These results arise mainly from a decrease of k_nr_, while fluorescence emission rate constant k_f_ remains globally constant within experimental error ((31±5) ⋅ 10^−6^ s^−1^, **6 l** not considered). To finish, nitrated product **9** presented the expected hypsochromic shift upon the introduction of an EWG at position 6 – *in contrario* to the trend observed above for electron‐poor groups in positions 2, 7 or 12. Luminescence properties of **9** reach now interesting values of 0.49 and 14.8 ns for fluorescent quantum yield and lifetime, respectively (Table 2), due to significant lowering of the non‐radiative deactivation (k_nr_).

**Table 2:** Photophysical properties.^[a]^

Product	λ_abs_ (nm)	ϵ (L.mol^−1^ ⋅ cm^−1^)	λ_em_ (nm)	Stokes shift (cm^−1^)	Φ_f_^[b]^	τ_f_ (ns)	k_f_^[c]^ (⋅10^6^ s^−1^)	k_nr_^[d]^ (⋅10^6^ s^−1^)
1	614	13140	667	1294	0.14	5.60	25	154
6 a	615	15450	662	1154	0.26	9.03	29	82
6 b	613	16990	660	1161	0.24	9.20	26	83
6 c	622	17090	673	1218	0.21	7.50	28	105
6 d	632	17270	676	1030	0.21	6.79	31	116
6 e	629	18000	672	1017	0.22	7.35	30	106
6 f	630	19890	674	1036	0.23	7.37	31	104
6 g	628	18780	673	1067	0.23	7.42	31	104
6 h	631	17610	675	1033	0.23	6.97	33	110
6 i	612	18070	658	1142	0.32	9.66	33	70
6 j	615	20560	664	1200	0.33	9.12	36	73
6 k	618	15510	669	1233	0.22	8.75	25	89
6 l	616	16190	663	1151	0.12	5.74	21	153
6 m	620	22010	663	1046	0.30	8.96	33	78
6 n	613	19640	659	1138	0.33	9.59	34	70
6 o	608	17840	654	1156	0.37	10.4	36	61
6 p	613	19280	662	1205	0.30	9.36	32	75
6 q	616	20550	665	1196	0.24	6.70	36	113
9	589	16280	633	1180	0.49^[e]^	14.8	33	34

[a] in acetonitrile[b] Reference: oxazine 170 perchlorate (Φ_f_=0.579 in EtOH). [c] With k_f_=Φ_f_/τ. [d] With k_nr_=(1‐Φ_f_)/τ [e] For this compound, reference was different: cresyl violet (Φ_f_=0.578 in EtOH)

**Figure 4:**
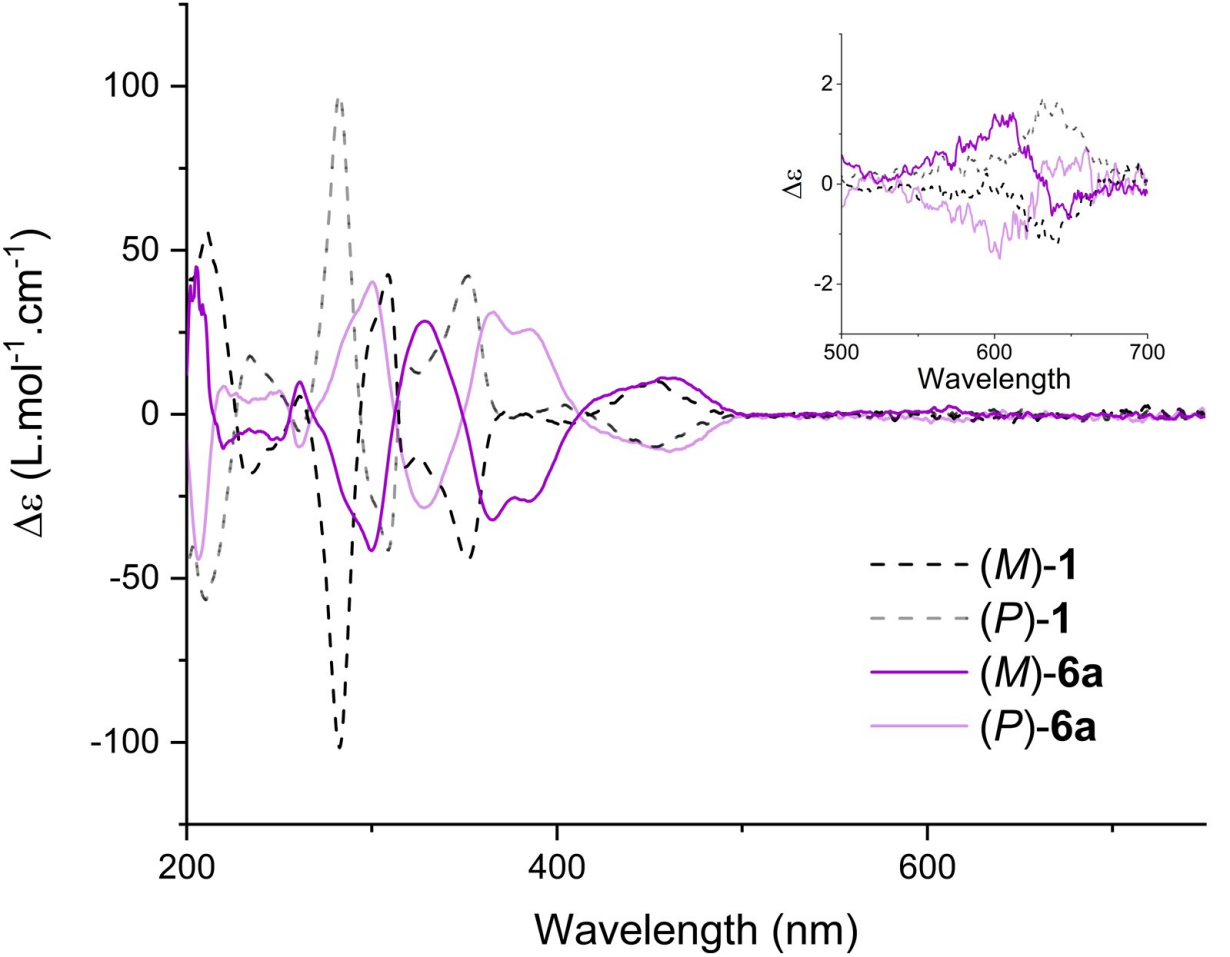
ECD spectra of (*M*)‐**1** (dotted black line), (*P*)‐**1** (dotted grey line), (*M*)‐**6 a** (purple line) and (*P*)‐**6 a** (pink line) in acetonitrile solution. Inset: expansion between 500 and 700 nm.

At this stage, having access to compounds **6 a** to **6 q** as single enantiomers, their chiroptical properties were studied in acetonitrile. All ECD spectra show a mirror image relationship for pairs of enantiomers and are reported in the Supporting Information (Figures S133–S150). As for parent DMQA **1**, the *para* tris(arene) derivatives present pronounced ECD bands in the UV domain reaching |Δϵ| ∼50 L ⋅ mol^−1^ ⋅ cm^−1^. Differences in the shape of the spectra can be noticed, especially between 330 and 430 nm. Compounds **6 a** and **6 i**‐**6 q** bearing electron rich arenes present two peaks of the same sign (Figure S134 and S142–150). In this region, derivatives **6 b**‐**6 h** with electron poor arenes have a single Cotton effect (Figure S135‐141) allied with the peak centered around 380 nm in the absorption spectra (e. g., **6 h** Figure 3a). Above 400 nm, the |Δϵ| values are small, especially for the lowest energy band (∼3 L ⋅ mol^−1^ ⋅ cm^−1^). A noticeable difference can be seen between unfunctionalized compound **1** and tris(arene) derivatives **6**. In fact, naked derivative **1** displays Cotton effects with the same sign between 500–700 nm (λ_max_ 614 nm); (*M*)‐ and (*P*)‐**1** have negative and positive bands, respectively. On the other hand, *para* tris(arene) derivatives such as **6 a** present ECD spectra with an inversion of sign within this 500–700 nm region. This is clearly visible in the inset of Figure 4; ECD of (*M*)‐**6 a** is first positive at 600 nm and becomes negative below 630 nm, and *vice versa* for (*P*)‐**6 a**. This inversion of sign indicates that the 500–700 nm absorption band is most likely composed of more than one transition, the sign and intensity of the Cotton effects being influenced by *para*‐arene substituents introduced on the helical core.^[33]^ In any case, the lowest energy transition (>630 nm) is negative and positive for (*M*)‐ and (*P*)‐enantiomers for all derivatives **1** and **6**.

CPL spectra were also recorded for nine selected derivatives with either EDG or EWG substituents on the added arenes; mirror images for both enantiomers are observed with low ΔI values (Figures S160–S169). As expected, and in relation with the ECD, negative and positive signs for (*M*)‐ and (*P*)‐**6** are obtained, respectively. Overall, compounds **6** present modest *g*_lum_ values around ±1 ⋅ 10^−4^ in acetonitrile. Such low values are again consistent with the absorption dissymmetry factors (*g_abs_*) observed for the most red‐shifted Cotton‐effect in ECD spectra of the same compounds. Such correlation is often observed in helicenes, where the geometry of the ground and the emitting excited state is similar.^[34]^

Finally, care was taken to tackle the possible changes in (chir)optical properties upon aggregation in both solution and solid state. Previously, it was reported that dimeric aggregates of compound **1** are formed in water solution. This affects mostly the fluorescence decay, while little changes are noticeable in absorption and emission spectra.^[7a]^ Herein, the effect of aggregation on the chiroptical properties of **1** was addressed by preparing equimolar solutions in acetonitrile with increasing percentage of water as anti‐solvent (0‐90 %). Absorption and ECD signals are reported in Figures S151–155. As expected, by increasing amount of anti‐solvent, aggregation of **1** does not lead to significant changes in the ECD spectra. Focus was then given to compound **6 m** containing tetraphenylethene (TPE) motifs; these supramolecular building blocks being often capable of forming organized assemblies.^[35]^ The fact that TPE moieties adopt non‐planar conformations in aggregated states^[36]^ was also considered as their association onto the enantiopure helicene core could influence the overall chiroptical properties. Of note, while TPE is one of the best‐known fluorogen for aggregation‐induced emission (AIE),^[37]^ the attachment of three such moieties to the cationic core of **1** was not designed to afford AIE. In fact, benzene group linkers separate the TPE moieties from the helical core which behave then as separated chromophores.^[38]^

**Figure 5:**
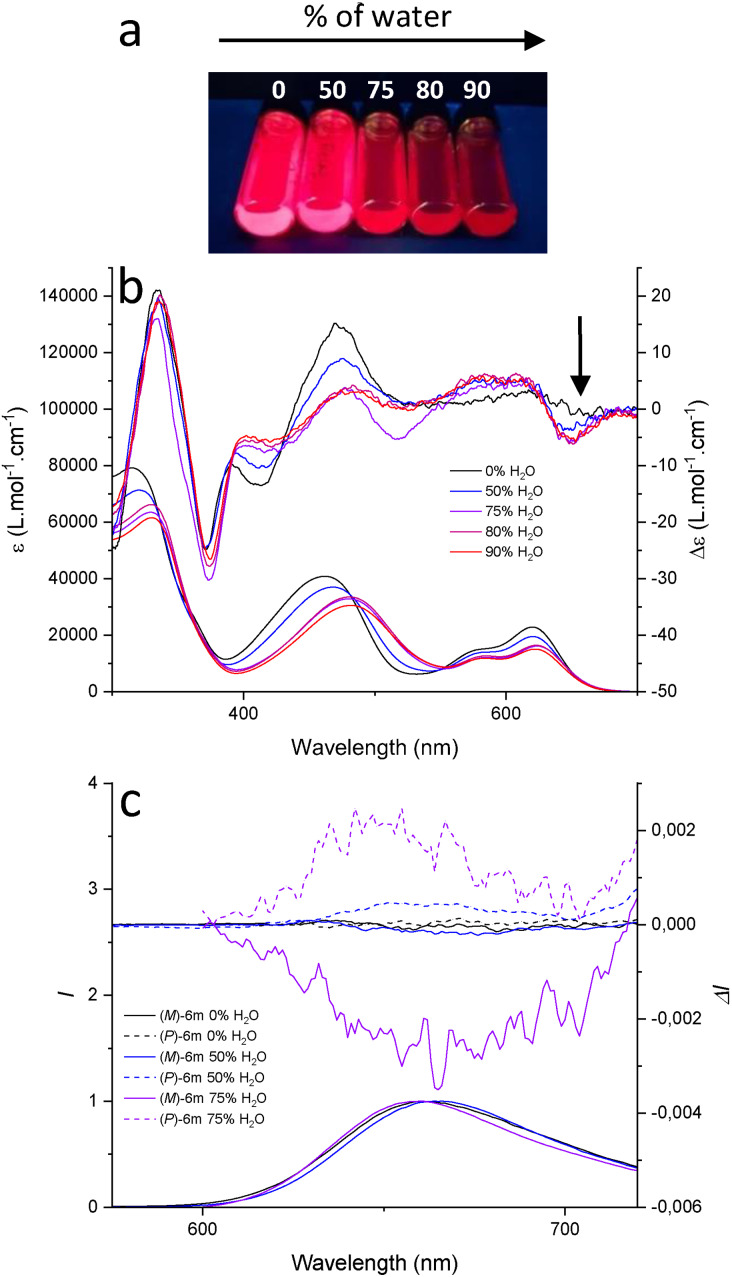
**a**: Picture of **6 m** with increasing amount of water (0‐90 %) in acetonitrile, taken under 365 nm irradiation. **b**: Evolution of absorption and ECD spectra of **6 m** with increasing water content (0–90 %), (*M*)‐enantiomer only shown for clarity; **c**: Emission and CPL spectra of (*M*)‐**6 m** (full lines) and (*P*)‐**6 m** (dotted lines) with increasing water content (0–75 %).

The aggregation behavior of **6 m** was studied via solvophobic effect (water 0–90 % in acetonitrile). Absorption, emission, and allied ECD and CPL signals are represented in Figure 5 and Figures S156–158 and relevant data are reported as well in Table 3. Overall, in absorption and ECD, strong evolutions are observed, those being interpreted as an evidence for the targeted supramolecular aggregation. In contrast to the behavior of the parent compound **1** in acetonitrile/water mixtures (Figures S151–155), these changes point out to the important role of the newly added TPE moieties in forming higher order assemblies. Upon addition of water, a relatively strong ECD band appears with a λ_max_ of 650 nm, reaching a plateau with 75 % of H_2_O and a maximum *g*_abs_ value of ca. 1.0 ⋅ 10^−3^ (Figure 5b). Importantly, this increased ECD response translates into an improved CPL signal. In fact, with solutions of (*P*)‐ and (*M*)‐**6 m** containing 50 % and 75 % of water, *g*_lum_ values equal to +3.9/−2.0 ⋅ 10^−4^ and +1.9/−2.8 ⋅ 10^−3^ are obtained, respectively (Figure 5c). Unfortunately, the Φ_f_ of **6 m** decreases to the estimated values of 0.2 and 0.02 for the 50 % and 75 % of water solutions (Figure S132), respectively,^[39]^ mitigating the strong increase of emission dissymmetry factor.

**Table 3:** Chiroptical properties of **6 m** with increasing amount of water (0–75 %) in acetonitrile.

Solvent	λ_abs_ [nm]	ϵ [L⋅mol^−1^⋅cm^−1^]	|*g*_abs_| ^[a]^	λ_em_ [nm]	Φ_f_	|*g*_lum_|^[b]^	*B*_CPL_ [L⋅mol^−1^⋅cm^−1^]
MeCN	620	22710	–	660	0.3	–	–
50/50 MeCN/H_2_O	620	20890	3.6 ⋅ 10^−4^	660	0.2	3.0 ⋅ 10^−4^	0.63
25/75 MeCN/H_2_O	623	15500	1.0 ⋅ 10^−3^	664	0.02	2.4 ⋅ 10^−3^	0.37

[a] *g*_abs_ values are measured at the λ_max_ of the lowest energy ECD band for each solution; [b] *g*_lum_ are given as average values over the whole emission band.

CPL brightness (*B*_CPL_)^[40]^ calculated for both conditions is equal to 0.63 M^−1^ ⋅ cm^−1^ and 0.37 M^−1^ ⋅ cm^−1^, respectively (Table 3). Therefore, the 50/50 acetonitrile/water mixture gives the best compromise between intense emission and improved circular polarization. Such *B*_CPL_ values in the 0.5 range are favorable in comparison with that of **1** (0.14 M^−1^ ⋅ cm^−1^) and **6 a** (0.22 M^−1^ ⋅ cm^−1^), in particular considering the red domain of emission. To conclude and confirm that the observed changes in (chir)optical properties upon addition of water as anti‐solvent are due to the formation of aggregates, additional ECD measurements were performed in solid state.^[41]^ (*P*)‐ and (*M*)‐**6 m** were first dispersed into a poly(methylmethacrylate) (PMMA) matrix (3 % wt). As expected for the equivalent of a solid solution, the ECD spectra resembled that in acetonitrile (Figure S159). However, studying the thin layers obtained upon drop casting or spin coating procedures of CH_2_Cl_2_ solutions of enantiopure **6 m** afforded ECD spectra analogous to that with high H_2_O% in acetonitrile (Figure S158).^[42]^

## Conclusion

Using classical cationic diaza [4]helicene **1** as the substrate, it was possible to select three out of nine C(sp^2^)−H bonds for triple chemo and regioselective borylations under Ir‐catalyzed conditions. The resulting tris(Bpin) adduct was found to be sensitive to moisture but tandem Suzuki−Miyaura cross‐couplings afforded tris(arene) derivatives efficiently (yields up to 68 %) with a perfect regioselectivity again. The process is enantiospecific and proceeds with full retention of configuration (e.s. 99 %). The poly‐introduction of additional aryl groups is particularly useful for the improvement of certain optical properties, luminescence in particular (quantum yield 14→37 % and lifetime 5.6→10.4 ns in CH_3_CN). Such improvements in the red domain of the visible spectrum should help the preparation of new series of biological probes.^[43]^ Finally, this Ir‐catalyzed LSF protocol can be coupled with previously developed post‐functionalization methods, for example, the nitration of the helical core. Combination of both LSF strategies now afford highly luminescent helicenes in the red domain (Φ_f_ 49 %, 14.8 ns, λ_max_ 633 nm). ECD signatures from the UV to the far‐red domain were further obtained for these tris(arene) derivatives. Moderate CPL signals (*g*_lum_ ±1 ⋅ 10^−4^) are observed for selected derivatives **6** in solution. For tris(TPE) substituted **6 m** water induced aggregation provokes a strong increase of *g*_abs_ and *g*_lum_ (10^−3^ range).

## Experimental Section

Full details of experimental conditions, characterizations of all new compounds, UV‐Vis and fluorescence spectra can be found in the Supporting Information.

Deposition Numbers 2171649 (for **6 e**), 2171650 (for **6 o**) contain the supplementary crystallographic data for this paper. These data are provided free of charge by the joint Cambridge Crystallographic Data Centre and Fachinformationszentrum Karlsruhe Access Structures service.

## Conflict of interest

The authors declare no conflict of interest.

1

## Supporting information

As a service to our authors and readers, this journal provides supporting information supplied by the authors. Such materials are peer reviewed and may be re‐organized for online delivery, but are not copy‐edited or typeset. Technical support issues arising from supporting information (other than missing files) should be addressed to the authors.

Supporting Information

## Data Availability

The data that support the findings of this study are openly available in yareta.unige.ch at https://doi.org/10.26037/yareta:vtd325xp6nfnzpyilnrcww2dxe. It will be preserved for 10 years.
