# Supplementary material for: Triple Regioselective Functionalization of Cationic [4]Helicenes via Iridium‐Catalyzed Borylation and Suzuki Cross‐Coupling Reactivity
Source: Chemistry. 2022 Aug 18;28(56):e202201853. doi: 10.1002/chem.202201853 (PMC9804708; doi:10.1002/chem.202201853)
Supplement: Supplementary file 1 — Supporting Information [file CHEM-28-0-s001.pdf]

Chemistry–A European Journal

Supporting Information

Triple Regioselective Functionalization of Cationic [4]Helicenes via Iridium-Catalyzed Borylation and Suzuki Cross-Coupling Reactivity

Lucas Frédéric, Bibiana Fabri, Laure Guénée, Francesco Zinna, Lorenzo Di Bari, and Jérôme Lacour*

Contents

Synthesis S5

Optimization studies S5

General Procedure – Steps 1 and 2 combined S10

New compounds: synthesis and characterization S11

Chiral Stationary Phase (CSP) HPLC S24

DMQA **1**: enantiomeric excess analysis S24

Tris(arene) **6j**: enantiomeric excess analysis S25

CSP-HPLC resolution of tris(arene) **6l** S26

NMR and IR spectra, and mass spectroscopy S27

Additional NMR and MS spectra for optimization or mechanistic studies S81

Solid state analysis (X-Ray diffraction) S86

Optical Properties S98

Absorbance and luminescence S98

Additional spectrum S107

Chiroptical Properties S108

Electronic Circular Dichroism and g_{abs} S108

Additional spectra S119

Circularly Polarized Luminescence and g_{lum} S124

Fluorescence lifetime S129

References S148

General considerations

Dataset:
The dataset for this article can be found at the following DOI: 10.26037/yareta:vtd325xp6nfnzpyilnrcww2dxe. It will be preserved for 10 years.

Reagents and solvents:
Unless otherwise stated, reagents were purchased from commercial sources and used without further purification. All reactions involving air sensitive compounds were carried out under N_2 via an inert gas/vacuum double manifold line and standard Schlenk techniques using dry solvents. Reactions involving oxygen sensitive reagents were performed using degassed solvents. Tetrahydrofuran (THF) was distillated under N_2 atmosphere over sodium and benzophenone.

Thin layer chromatography and purification:
Analytical thin layer chromatography (TLC) and retardation factors (R_f) were performed with Silica gel 60 F_{254} aluminium plates purchased from Merck. Column chromatography were performed using Silicaflash P60 silicagel (40-63 µm, 60 Å) and with CombiFlash® *R*f 200 with adapted SiO_2 cartridges.

NMR:
NMR spectra were recorded on Bruker Avance III 500 MHz, Bruker Avance III HD-*NanoBay* 400 MHz and Bruker Avance III HD-*NanoBay* 300 MHz spectrometers at room temperature. NMR chemical shifts are given in ppm (δ) relative to Me_4Si with solvent resonances used as internal standards (CD_2Cl_2: 5.32 ppm for ^{1}H and 53.8 for ^{13}C). Proton (^{1}H) NMR information is given in the following format: multiplicity (s, singlet; d, doublet; t, triplet; q, quartet; quin, quintet; sex, sextet; sept, septet; m, multiplet), the prefix br- was applied when the signal was broadened, coupling constant(s) (J) in Hertz (Hz), number of protons.

Infrared Spectroscopy:
IR spectra were recorded on a Perkin-Elmer 1650 FT-IR spectrometer using a diamond ATR Golden Gate sampling.

Mass Spectrometry:
Low resolution mass spectrometry: Electrospray mass spectra in methanol solutions on an API 150EX (AB/MDS Sciex) spectrometer in positive polarity

High resolution mass spectrometry: Electrospray mass spectra were obtained on a Xevo-G2-TOF HRMS by the Department of Mass Spectroscopy of the University of Geneva.

Melting point:
Melting points were measured on a standard melting point apparatus in open capillary vials and are uncorrected.

Optical properties:
All (chir)optical measurements were performed in 1cm optical quartz cells.

Absorption spectra were recorded on a JASCO V-650 spectrophotometer at 20 °C in analytical grade solvents (*ca.* 10^{-5} M).

Electronic circular dichroism (ECD) spectra were recorded on a JASCO J-815 or JASCO J-715 spectrophotometer at 20 °C. Measurements were performed in analytical grade acetonitrile at precise concentration (*ca.* 10^{-5} M).

Steady-state fluorescence spectra were measured using a HORIBA Jobin Yvon Fluoromax Plus spectrofluorimeter. All fluorescence spectra were corrected for the wavelength-dependent sensitivity of the detection. Unless specified, excitation wavelength used was 10 nm before the start of the acquisition of the spectrum. Fluorescence quantum yields Φ were measured in diluted solutions (at least 3 different measurements for each enantiomers) with an optical density lower than 0.1 using the following equation:

$$\Phi_x = \Phi_r \frac{A_r(\lambda)}{A_x(\lambda)} \frac{{n_x}^2}{{n_r}^2} \frac{S_x}{S_r}$$

Where A is the absorbance at the excitation wavelength (λ), n the refractive index and S the area under the emission peak. "r" and "x" stand for reference and sample. The fluorescence quantum yields were measured relative to oxazine 720 (also called oxazine 170) in ethanol (Φ = 0.579) for every derivatives except **9** where Cresyl violet in ethanol (Φ = 0.578) was used.

Fluorescene lifetime: Time Correlated Single Photon Counts (TCSPC) experiments were performed on an Edinburgh FS-5 spectrofluorometer with SC-20 module coupled with a 365 nm Picosecond pulsed diode lasers. A 10 mm*10 mm quartz cell was used. Excitation wavelength were adapted for every compound (depending on their absorption spectra).Fluorescence spectra are fitted with one or two exponential decay(s) using implemented software (Edinburgh instrument). When double decays were used, τ_f was calculated as the weighted average value of both τ (using pre exponential terms). Fluorescence emission rate (k_f) and nonradiative decay rate constants (k_{nr}) were calculated from the fluorescence quantum yield (Φ_f) and the lifetime constant (τ_f) by using following equations:

$$\Phi_f = \frac{k_f}{k_f + k_{nr}} = k_f\, \tau_f$$

$$\tau_f = \frac{1}{k_f + k_{nr}}$$

Circularly polarized luminescence (CPL) spectra were recorded using a home-built spectrofluoropolarimeter [1] under 365 nm irradiation from a commercial LED-source, using a 90° geometry between the excitation and detection direction. All the spectra were recorded in air-equilibrated analytical grade acetonitrile at concentrations *ca.* 10^{-5} M, using the following parameters: scan-speed – 2 nm/sec, integration time – 2 sec, photomultiplier tube driving voltage – 500 V, accumulations – 8. In the case of aggregated compound **6m**, the excitation beam was linearly polarized parallel to the direction of detection.

Crystallography:
All data were collected on a Synergy Hypix dual source diffractometer (Rigaku) using Cu Kα radiation. Data reduction was carried out in the CrysalisPro Software [2]. Using Olex2, [3] the structure was solved with the SHELXT [4] structure solution program using dual space method and refined with the SHELXL [4] refinement package using Least Squares minimisation. All other calculations and drawings were performed with Olex2 [3] and ORTEP [5] programs.

Synthesis

Racemic and enantiopure **[DMQA][BF_4] 1** were synthesized according to reported procedures.[6]

Optimization studies

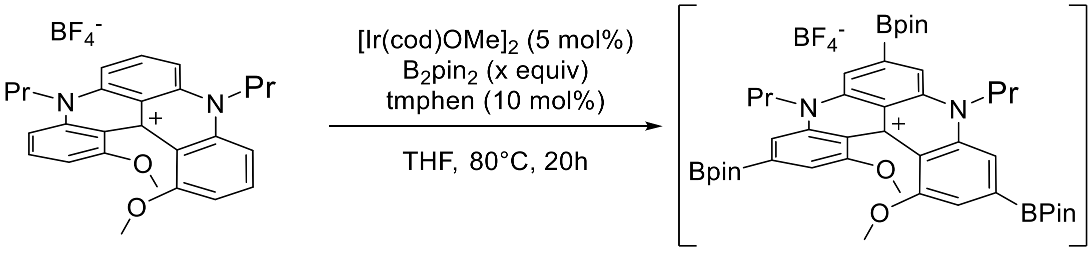

rac-**1** *rac*-**5**

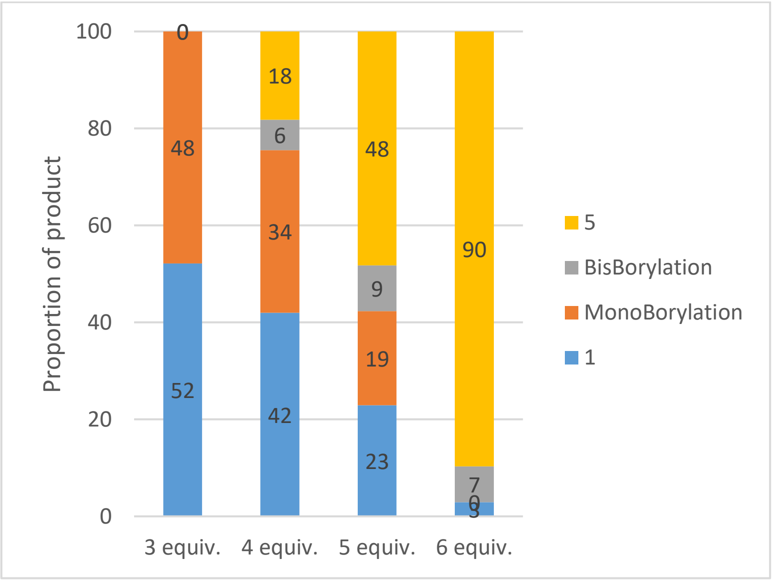

Graphic S1: Optimization of number of equivalents of B_2pin_2

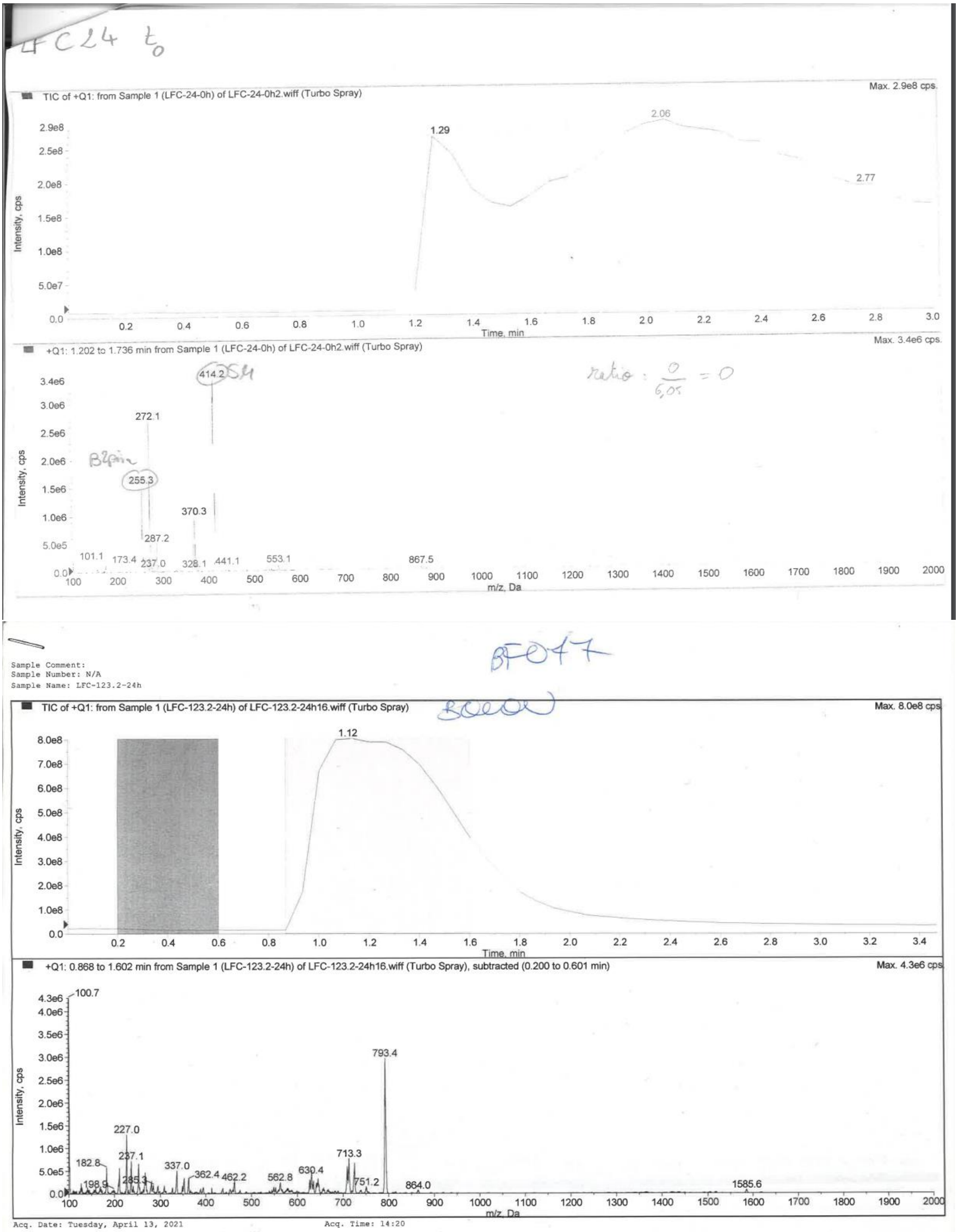

Figure S1: Representative mass spectrometry data before (upper graph) and after (lower graph) the *in-situ* borylation step. m/z 414.2 corresponds to compound **1** and m/z 793.4 to compound **5**.

For graphics **S2** and **S3**, the borylation step was conducted as presented in "step 1" (see below). In step 2 (cross-coupling step) 4-fluorobromobenzene was used as aryl bromide.

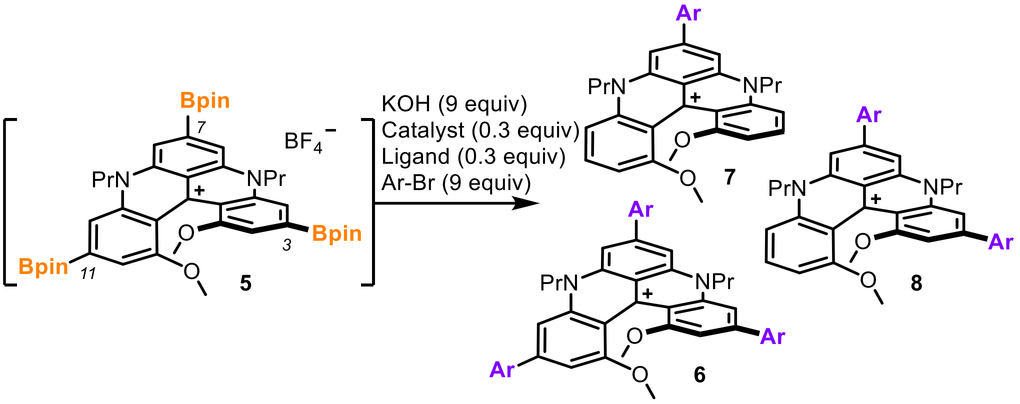

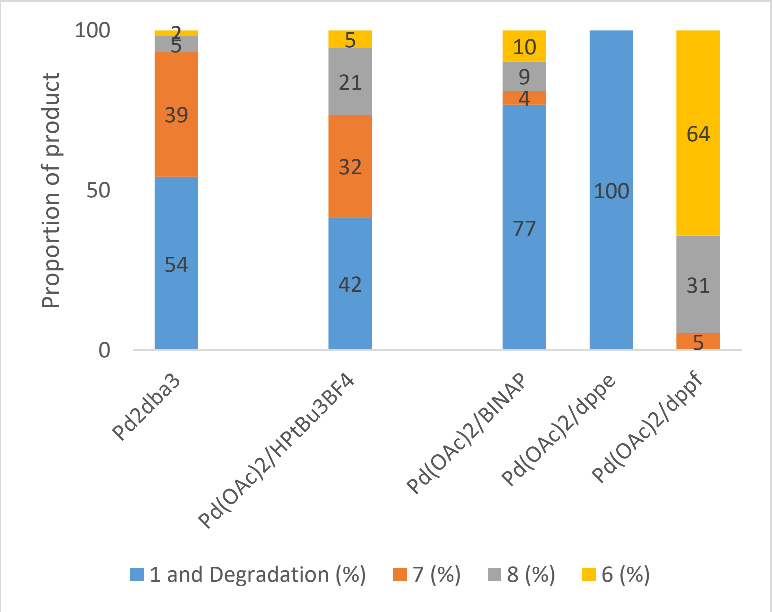

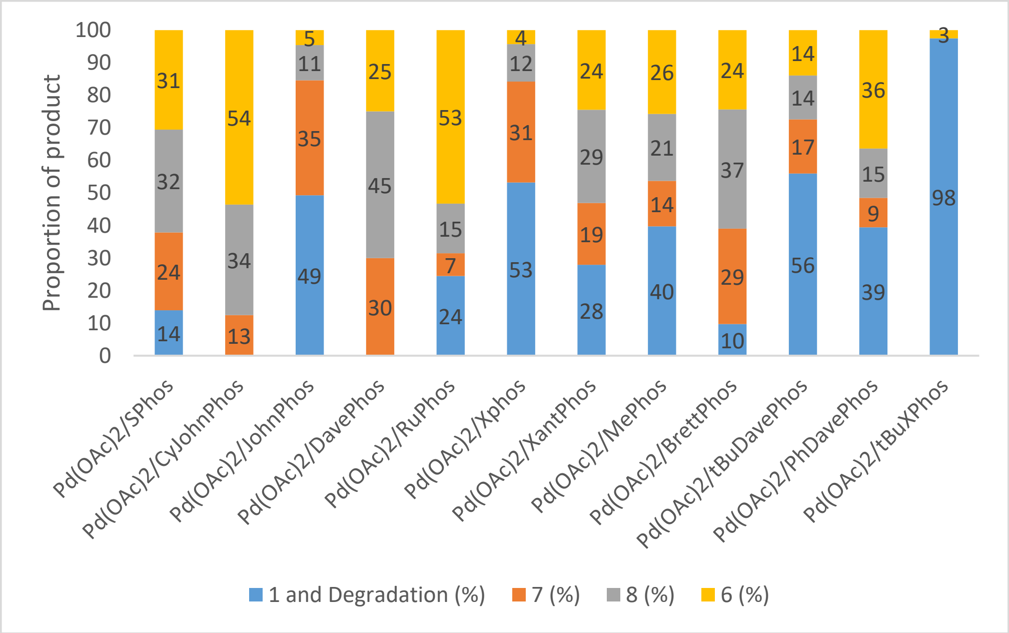

Graphic S2: Screen of catalyst and ligands

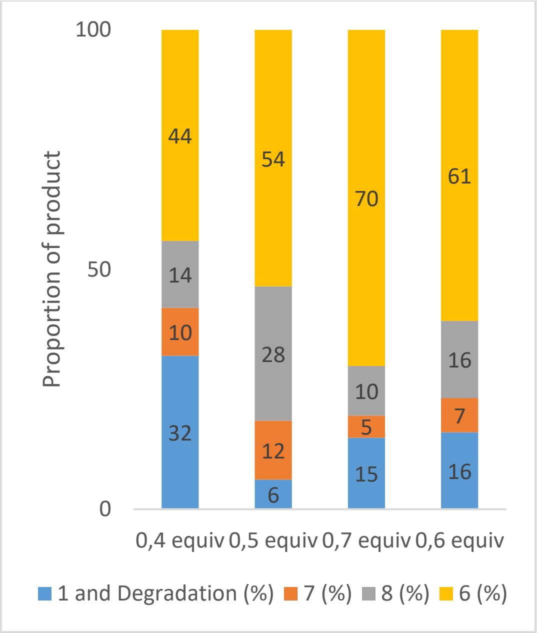

Graphic S3: Optimization of number of equivalent of $Pd(OAc)_2$ and dppf

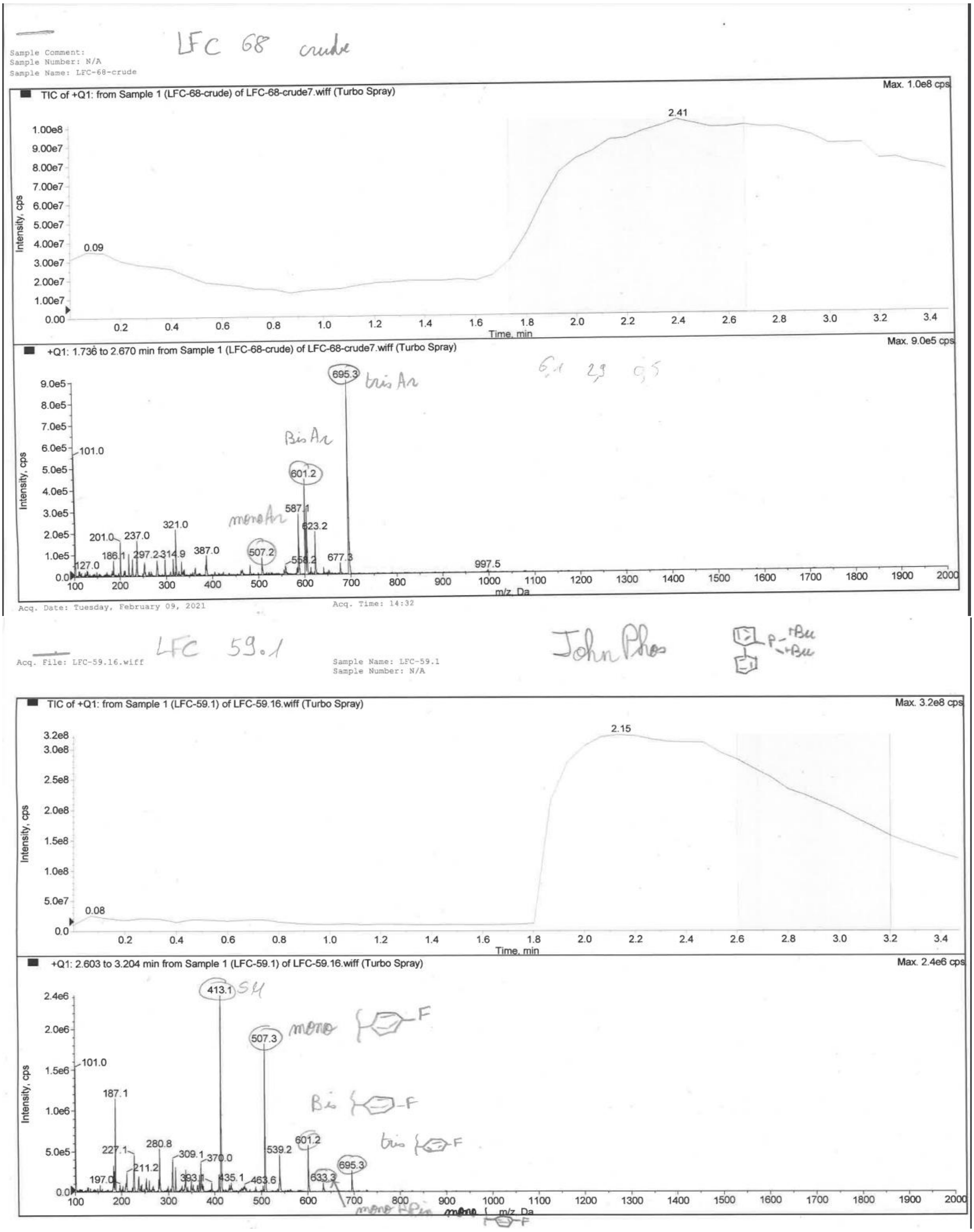

Figure S2: Representative mass spectrometry data for ligand screening. Upper graph: attempt with dppf as ligand. Lower graph: attempt with JohnPhos as ligand. m/z 695.3 corresponds to **6b**, m/z 601.2 corresponds to **8b**, m/z 507.3 corresponds to **7b** and m/z 413.1 corresponds to **1**.

General Procedure – Steps 1 and 2 combined

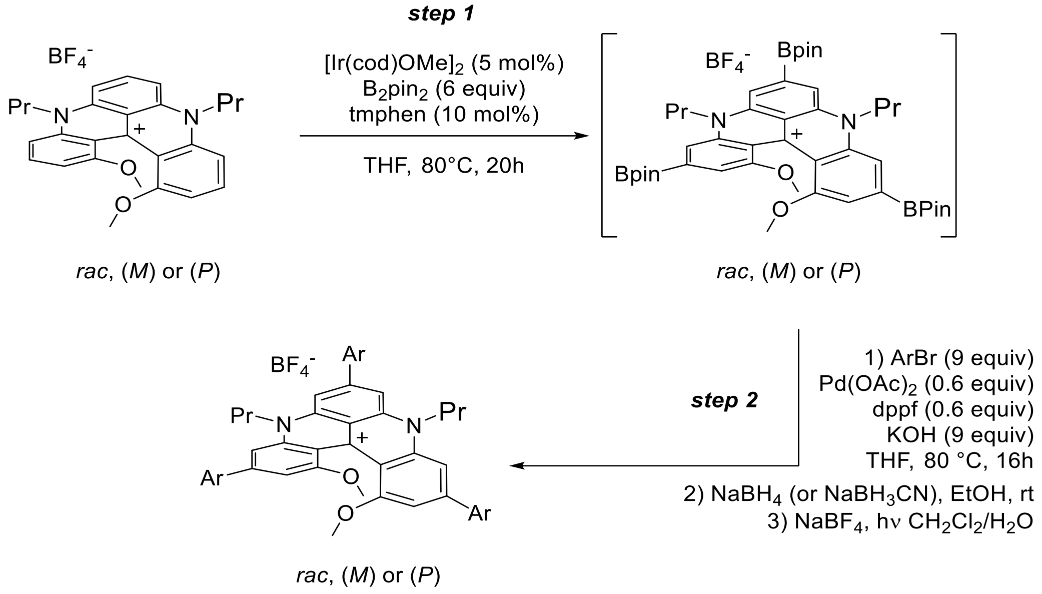

Step 1: In a dry 5 mL microwave vial (Biotage), equipped with a stirring bar, were added racemic or enantiopure (*M*)-/(*P*)-[DMQA][BF_4] (50 mg, 0.1 mmol, 1 equiv), 3,4,7,8-tetramethyl-1,10-phenanthroline (2.36 mg, 0.01 mmol, 10 mol%), (1,5-cyclooctadiene)(methoxy)iridium(I) dimer (3.31mg, 0.005 mmol, 5 mol%) and bis(pinacolato)diboron (152 mg, 0.6 mmol, 6 equiv). The vial was then sealed, put under vacuum and backfilled with dinitrogen three times. Freshly distilled THF (2 mL) was added via a syringe under dinitrogen and the vial was placed in a pre-heated oil bath at 80 °C. Aliquots were collected to follow the borylation by mass-spectroscopy analysis. The reaction was considered as complete when no more racemic, (*M*)- or (*P*)-[DMQA][BF_4] salt could be observed and tris-borylated species **5** was the major component of the crude (after 20-24 h usually). It is important to note that, at this step, *leuco* adducts (of type **5-H** and analogous compounds) can be formed.

Step 2: In a dry 5 mL microwave vial (Biotage), equipped with a stirring bar, palladium acetate (13.5 mg, 0.06 mmol, 0.6 equiv), 1,1′-ferrocenediyl-bis(diphenylphosphine) (33 mg, 0.06 mmol, 0.6 equiv) and freshly ground potassium hydroxide (50 mg, 0.9 mmol, 9 equiv) were added together. If solid, the aryl bromide is added at this step (0.9 mmol, 9 equiv) with the other reagents. Then the vial is sealed, put under vacuum and backfilled with nitrogen three times. If liquid, the aryl bromide is added (0.9 mmol, 9 equiv) and then the mixture resulting from step 1 via syringe or canula and the reaction mixture was heated at 80 °C (oil bath). The reaction was monitored by mass-spectroscopy analysis and considered as complete when no more borylated species could be observed (usually 12-16 h).

After completion, the reaction mixture was filtered through Celite (dichloromethane as solvent). After concentration in vacuo, the mixture was dissolved in ethanol (2-3 mL). Then sodium borohydride (7.5 mg, 0.2 mmol, 2 equiv) was added. If reduction-sensitive substituents are present sur as ketones or nitriles functional groups, sodium cyanoborohydride (12.5 mg, 0.2 mmol, 2 equiv) was used instead. Reduction of crude **6** and formation of **6-H** was monitored by TLC (1 to 4 h depending on the aryl, eluent cyclohexane/ethyle acetate). As hydrido products **6-H** are light-sensitive in open air, after concentration in vacuo, it is recommended to purify products **6-H** rapidly by flash chromatography. Fractions corresponding to the expected product were gathered and evaporated. The white/green solids were dissolved in dichloromethane (20 mL) followed by water (20 mL) plus a spoon of sodium tetrafluoroborate (i.e. 2-3 g). The resulting biphasic mixture, with slightly

green and transparent organic and aqueous layers respectively, was stirred very strongly and submitted to light irradiation (Ostram Ultra Vitalux 300 W) for a minimum of 1 h. Transformation of the organic layer into a deep green solution with red reflects occurred, and completion of the oxidation into **6⁺** was monitored by TLC (eluent dichloromethane/MeOH). After separation, the organic layer was extracted three times, dried over Na_2SO_4 and, after filtration, the solvent was removed under reduced pressure. The product was further purified by flash chromatography to afford the titled compound as a green solid. A last precipitation step is usually performed by solubilizing the product in a minimum of CH_2Cl_2 followed by an addition of Et_2O. After precipitation / centrifugation, the supernatant was removed with a Pasteur pipet.

New compounds: synthesis and characterization

1,13-dimethoxy-3,7,11-triphenyl-5,9-dipropyl-5,9-dihydro-13bH-quinolino[2,3,4-kl]acridin-13b-ylium tetrafluoroborate salt (6a)

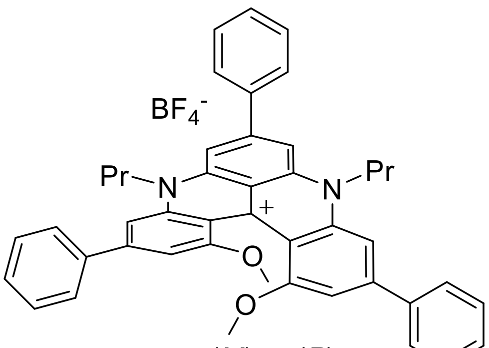

rac, (*M*) or (*P*)

Compound **6a** is obtained following the general procedure for borylation and Suzuki cross-coupling. Bromobenzene (95 µL, 141.31 mg, 0.9 mmol, 9 equiv) and sodium borohydride were used. Purification of the reduced product was performed with flash column chromatography (cyclohexane/ethyl acetate 95/5). After oxidation step in presence of BF_4^- anion (see general procedure), the expected product **6a** was purified with flash column chromatography (dichloromethane/acetone 85/15, R_f= 0.3) giving a dark green solid, 27.6 mg, yield: 38%.

¹H NMR (500 MHz, CD_2Cl_2) δ 7.88 – 7.83 (m, 6H), 7.67 – 7.55 (m, 11H), 7.52 (d, *J* = 1.3 Hz, 2H), 7.13 (d, *J* = 1.4 Hz, 2H), 4.76 (ddd, *J* = 15.2, 10.9, 6.0 Hz, 2H), 4.55 (ddd, *J* = 15.6, 10.8, 5.7 Hz, 2H), 3.89 (s, 6H), 2.32 – 2.17 (m, 4H), 1.29 (t, *J* = 7.4 Hz, 6H)

¹³C NMR (126 MHz, CD_2Cl_2) δ 160.1, 150.1, 149.7, 142.5, 142.0, 140.0, 139.7, 139.4, 129.7, 129.7, 129.5, 129.4, 128.2, 127.6, 118.5, 112.5, 105.3, 103.9, 102.7, 55.9, 51.6, 20.0, 11.1.

¹⁹F NMR (282 MHz, CD_2Cl_2) δ -153.31, -153.36.

IR (neat, cm^{-1}): ν 609.5, 699.2, 764.2, 839.9, 1056.6, 1208, 1289.9, 1349.1, 1416.8, 1509.7, 1565, 1605.7, 2964.3

HRMS (ESI) calculated for $C_{45}H_{41}N_2O_2^+$ (M-BF4): m/z 641.3163, found: 641.3183 (3.1 ppm)

M.P.: 185 °C (decomposition)

3,7,11-tris(4-fluorophenyl)-1,13-dimethoxy-5,9-dipropyl-5,9-dihydro-13bH-quinolino[2,3,4-kl]acridin-13b-ylium tetrafluoroborate (6b)

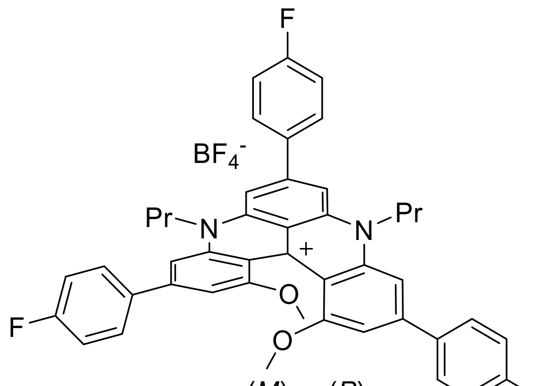

rac, (*M*) or (*P*)

Compound **6b** is obtained following the general procedure for borylation and Suzuki cross-coupling. 1-bromo-4-fluorobenzene (99 µL, 157.5 mg, 0.9 mmol, 9 equiv) and sodium borohydride were used. Purification of the reduced product was performed with flash column chromatography (cyclohexane/ethyl acetate 95/5). After oxidation step in presence of BF_4^- anion (see general procedure), the expected product **6b** was purified with flash column chromatography (dichloromethane/acetone 85/15, R_f = 0.32) giving a dark green solid, 38 mg, yield: 50%.

^{1}H NMR (500 MHz, CD_2Cl_2) δ 7.8 (ddd, *J* = 8.7, 5.2, 1.8 Hz, 6H), 7.5 (s, 2H), 7.5 (d, *J* = 1.4 Hz, 2H), 7.4 – 7.3 (m, 2H), 7.1 (d, *J* = 1.3 Hz, 6H), 4.8 (ddd, *J* = 15.7, 10.7, 5.8 Hz, 2H), 4.5 (ddd, *J* = 15.8, 10.8, 5.7 Hz, 2H), 3.9 (s, 6H), 2.3 – 2.1 (m, 4H), 1.3 (t, *J* = 7.4 Hz, 6H).

^{13}C NMR (126 MHz, CD_2Cl_2) δ 163.9 (d, *J* = 250.1 Hz), 163.8 (d, *J* = 250.1 Hz), 160.2, 149.0, 148.6, 142.5, 142.0, 139.4, 136.2, 135.8, 130.1 (d, *J* = 8.6 Hz), 129.6 (d, *J* = 8.5 Hz), 118.4, 116.5 (d, *J* = 22.1 Hz), 116.3 (d, *J* = 22.1 Hz), 112.5, 105.1, 103.9, 102.6, 55.9, 51.5, 20.0, 11.1.

^{19}F NMR (282 MHz, CD_2Cl_2) δ -112.09, -112.25, -153.06, -153.11.

IR (neat, cm^{-1}): ν 601.2, 825.6, 1054.4, 1132.8, 1162.2, 1237.4, 1290.1, 1347.0, 1400.7, 1463.9, 1516.9, 1567.8, 1603.8, 2970.5

HRMS (ESI) calculated for $C_{45}H_{38}F_3N_2O_2^+$ (M-BF4): m/z 695.2880, found: 695.2922 (6.0 ppm)

M.P.: 175 °C (decomposition)

1,13-dimethoxy-5,9-dipropyl-3,7,11-tris(4-(trifluoromethyl)phenyl)-5,9-dihydro-13bH-quinolino[2,3,4-kl]acridin-13b-ylium tetrafluoroborate salt (6c)

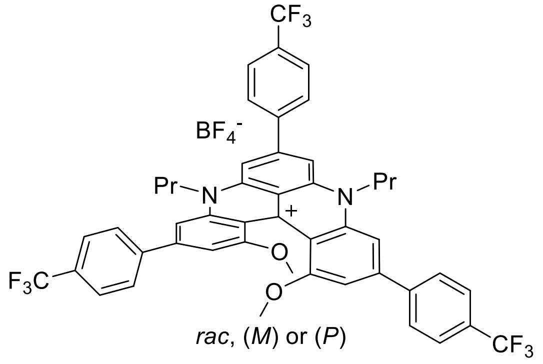

rac, (*M*) or (*P*)

Compound **6c** is obtained following the general procedure for borylation and Suzuki cross-coupling. 4-bromobenzotrifluoride (126 µL, 203 mg, 0.9 mmol, 9 equiv) and sodium borohydride were used. Purification of the reduced product was performed with flash column chromatography (cyclohexane/ethyl acetate 95/5). After oxidation step in presence of BF_4^- anion (see general procedure), the expected product **6c** was purified with flash column chromatography (dichloromethane/acetone 9/1, R_f = 0.26) giving a dark green solid, 38 mg, yield: 40%.

^{1}H NMR (500 MHz, CD_2Cl_2) δ 8.0 (dd, *J* = 8.5, 1.7 Hz, 6H), 7.9 (dd, *J* = 12.7, 8.1 Hz, 6H), 7.6 (s, 2H), 7.5 (d, *J* = 1.3 Hz, 2H), 7.1 (d, *J* = 1.2 Hz, 2H), 4.8 (ddd, *J* = 16.3, 10.8, 5.9 Hz, 2H), 4.6 (ddd, *J* = 15.9, 10.8, 5.7 Hz, 2H), 3.9 (s, 6H), 2.2 (tdd, *J* = 13.2, 10.3, 7.2 Hz, 4H), 1.3 (t, *J* = 7.5 Hz, 6H).

^{13}**C NMR** (126 MHz, CD_2Cl_2) δ 160.3, 148.7, 148.3, 143.6, 143.2, 142.5, 142.2, 139.5,131.6 (q, *J*=33.0 Hz), 131.3 (q *J* = 33.0 Hz), 128.8, 128.2, 126.4 (q, *J* = 3.5 Hz), 126.2 (q, *J* = 3.4 Hz), 124.1 (q, *J* = 272.3 Hz), 124.0 (q, *J*=272.3 Hz) 118.9, 113.0, 105.9, 104.3, 102.8, 56.0, 51.7, 20.1, 11.1.

^{19}**F NMR** (282 MHz, CD_2Cl_2) δ -62.93, -62.96, -152.88, -152.94.

IR (neat, cm^{-1}): ν 603.3, 826.7, 923.7, 1015.5, 1066.9, 1118.5, 1169.2, 1221.9, 1292.9, 1324.0, 1402.1, 1493.6n 1565.1, 1606.9, 2967.1

HRMS (ESI) calculated for $C_{48}H_{35}F_9N_2O_2^+$ (M-BF4): m/z 845.2784, found: 845.2787 (0.4 ppm)

M.P.: 327 °C (decomposition)

3,7,11-tris(4-cyanophenyl)-1,13-dimethoxy-5,9-dipropyl-5,9-dihydro-13bH-quinolino[2,3,4-kl]acridin-13b-ylium tetrafluoroborate salt (6d)

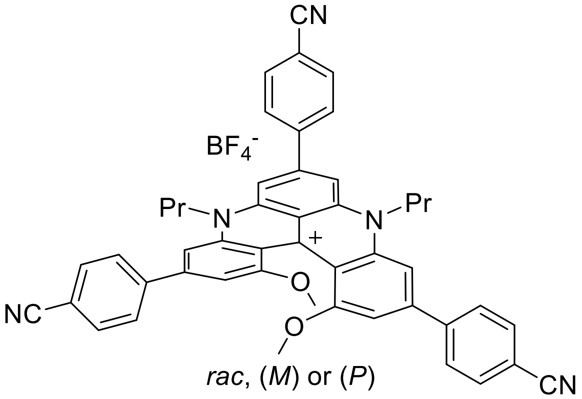

rac, (*M*) or (*P*)

Compound **6d** is obtained following the general procedure for borylation and Suzuki cross-coupling. 4-bromobenzonitrile (164 mg, 0.9 mmol, 9 equiv) and sodium cyano-borohydride were used. Purification of the reduced product was performed with flash column chromatography (cyclohexane/ethyl acetate 8/2). After oxidation step in presence of BF_4^- anion (see general procedure), the expected product **6d** was purified with flash column chromatography (dichloromethane/MeOH 9/1, R_f = 0.51) giving a dark green solid, 32 mg, yield: 40%.

^{1}**H NMR** (500 MHz, CD_2Cl_2) δ 8.0 – 7.9 (m, 6H), 7.6 (s, 2H), 7.5 (d, *J* = 1.3 Hz, 2H), 7.1 (d, *J* = 1.2 Hz, 2H), 4.8 (ddd, *J* = 16.0, 10.7, 5.8 Hz, 2H), 4.6 (ddd, *J* = 15.9, 10.7, 5.6 Hz, 2H), 3.9 (s, 6H), 2.2 (dddd, *J* = 27.1, 16.3, 13.4, 6.9 Hz, 4H), 1.3 (t, *J* = 7.3 Hz, 6H).

^{13}**C NMR** (126 MHz, CD_2Cl_2) δ 160.3, 148.2, 147.8, 144.1, 143.8, 142.5, 142.1, 139.5, 133.2, 133.1, 129.0, 128.5, 119.1, 118.3, 118.3, 113.4, 113.2, 105.9, 104.3, 102.7, 56.1, 51.6, 20.1.

^{19}**F NMR** (282 MHz, CD_2Cl_2) δ -152.49, -152.54.

IR (neat, cm^{-1}): ν 602.0, 708.0, 747.7, 824.6, 923.6, 1056.2, 1133.0, 1179.4, 1207.7, 1291.1, 1346.4, 1401.1, 1466.7, 1490.0, 1514.0, 1557.4, 1575.5, 1602.9, 2227.8, 2968.0, 3633.0.

HRMS (ESI) calculated for $C_{48}H_{38}N_5O_2^+$ (M-BF4): m/z 716.3021, found: 716.3059 (5.3 ppm)

M.P.: 324 °C (decomposition)

3,7,11-tris(4-acetylphenyl)-1,13-dimethoxy-5,9-dipropyl-5,9-dihydro-13bH-quinolino[2,3,4-kl]acridin-13b-ylium tetrafluoroborate salt (6e)

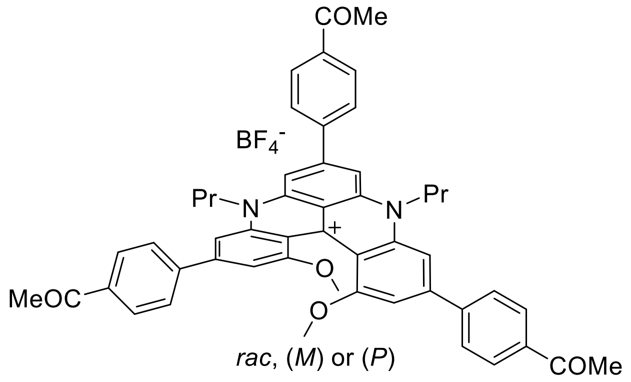

Compound **6e** is obtained following the general procedure for borylation and Suzuki cross-coupling. 4'-bromoacetophenone (179 mg, 0.9 mmol, 9 equiv) and sodium cyano-borohydride were used. Purification of the reduced product was performed with flash column chromatography (cyclohexane/ethyl acetate 7/3). After oxidation step in presence of BF_4^- anion (see general procedure), the expected product **6e** was purified with flash column chromatography (dichloromethane/MeOH 93/7, R_f = 0.38) giving a dark green solid, 29.3 mg, yield: 34%.

^{1}H NMR (500 MHz, CD_2Cl_2) δ 8.2 – 8.1 (m, 6H), 8.0 – 7.9 (m, 6H), 7.6 (s, 2H), 7.6 (d, *J* = 1.3 Hz, 2H), 7.2 (d, *J* = 1.2 Hz, 2H), 4.8 (ddd, *J* = 16.3, 10.7, 5.9 Hz, 2H), 4.6 (ddd, *J* = 15.8, 10.8, 5.7 Hz, 2H), 3.9 (s, 3H), 2.7 (s, 3H), 2.7 (s, 6H), 2.3 – 2.2 (m, 4H), 1.3 (t, *J* = 7.4 Hz, 6H).

^{13}C NMR (126 MHz, CD_2Cl_2) δ 197.3, 160.2, 148.9, 148.5, 144.1, 143.7, 142.5, 142.1, 139.5, 137.8, 137.7, 129.3, 129.2, 128.5, 128.0, 118.9, 113.0, 105.8, 104.2, 102.8, 56.0, 51.6, 26.7, 20.1.

^{19}F NMR (282 MHz, CD_2Cl_2) δ -152.79, -152.84.

IR (neat, cm^{-1}): ν 568.1, 824.6, 1056.0, 1268.7, 1355.7, 1576.6, 1603.0, 1681.1

HRMS (ESI) calculated for $C_{51}H_{47}N_2O_5^+$ (M-BF4): m/z 767.3480, found: 767.3476 (-0.5 ppm)

M.P.: 157 °C (decomposition)

3,7,11-tris(4-benzoylphenyl)-1,13-dimethoxy-5,9-dipropyl-5,9-dihydro-13bH-quinolino[2,3,4-kl]acridin-13b-ylium tetrafluoroborate salt (6f)

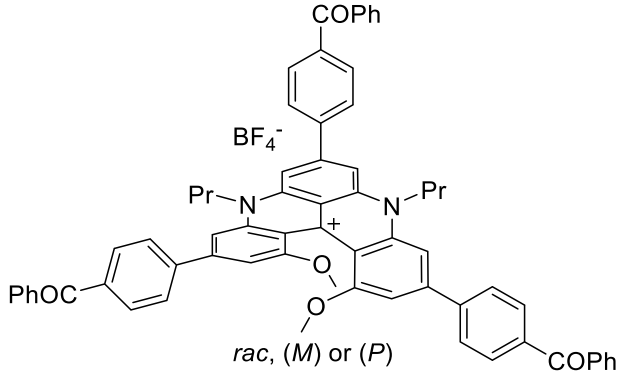

Compound **6f** is obtained following the general procedure for borylation and Suzuki cross-coupling. 4'-bromoacetophenone (179 mg, 0.9 mmol, 9 equiv) and sodium cyano-borohydride were used. Purification of the reduced product was performed with flash column chromatography (cyclohexane/ethyl acetate 8/2). After oxidation step in presence of BF_4^- anion (see general procedure), the expected product **6f** was purified with flash column chromatography (dichloromethane/MeOH 97/3, R_f = 0.14) giving a dark green solid, 46 mg, yield: 44%.

^{1}H NMR (500 MHz, CD_2Cl_2) δ 8.1 – 8.0 (m, 12H), 7.9 (ddd, *J* = 8.6, 7.4, 1.4 Hz, 6H), 7.7 – 7.6 (m, 5H), 7.6 (d, *J* = 1.4 Hz, 2H), 7.6 (td, *J* = 7.8, 3.9 Hz, 6H), 7.2 (d, *J* = 1.1 Hz, 2H), 4.8 (ddd, *J* = 16.1, 10.8, 5.8 Hz, 2H), 4.6 (ddd, *J* = 15.8, 10.8, 5.6 Hz, 2H), 3.9 (s, 6H), 2.4 – 2.2 (m, 4H), 1.3 (t, *J* = 7.4 Hz, 6H).

^{13}C NMR (126 MHz, CD_2Cl_2) δ 195.64, 160.26, 149.03, 148.63, 143.51, 143.19, 142.55, 142.10, 139.51, 138.51, 138.39, 137.29, 137.21, 132.85, 132.78, 130.97, 130.86, 130.01, 129.98, 128.49, 128.46, 128.24, 127.66, 118.90, 113.01, 105.76, 104.21, 102.80, 56.05, 51.70, 20.12, 11.12.

^{19}F NMR (282 MHz, CD_2Cl_2) δ -152.99, -153.04.

IR (neat, cm^{-1}): ν 544.5, 571.0, 595.4, 658.4, 700.7, 746.7, 792.3, 828.0, 922.9, 1048.7, 1177.0, 1208.2, 1274.4, 1314.5, 1346.2, 1399.5, 1488.6, 1572.9, 1600.1, 1651.5, 2924.4

HRMS (ESI) calculated for $C_{66}H_{53}N_2O_5^+$ (M-BF4): m/z 953.3949, found: 953.3951 (0.2 ppm)

M.P.: 197 °C (decomposition)

1,13-dimethoxy-3,7,11-tris(4-(methoxycarbonyl)phenyl)-5,9-dipropyl-5,9-dihydro-13bH-quinolino[2,3,4-kl]acridin-13b-ylium tetrafluoroborate salt (6g)

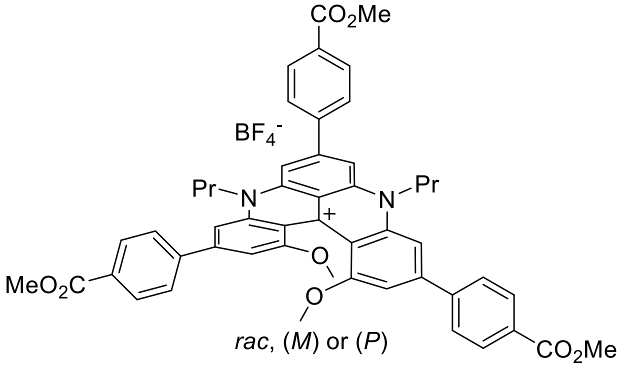

Compound **6g** is obtained following the general procedure for borylation and Suzuki cross-coupling. Methyl 4-bromobenzoate (194 mg, 0.9 mmol, 9 equiv) and sodium cyano-borohydride were used. Purification of the reduced product was performed with flash column chromatography (cyclohexane/ethyl acetate 8/2). After oxidation step in presence of BF_4^- anion (see general procedure), the expected product **6g** was purified with flash column chromatography (dichloromethane/MeOH 95/5, R_f = 0.32) giving a dark green solid, 41 mg, yield: 46%.

^{1}H NMR (500 MHz, CD_2Cl_2) δ 8.3 – 8.2 (m, 6H), 8.0 – 7.9 (m, 6H), 7.6 (s, 2H), 7.6 (d, *J* = 1.4 Hz, 2H), 7.1 (d, *J* = 1.2 Hz, 2H), 4.8 (ddd, *J* = 16.2, 10.9, 6.0 Hz, 2H), 4.6 (ddd, *J* = 15.9, 10.8, 5.7 Hz, 2H), 4.0 (s, 3H), 4.0 (s, 6H), 3.9 (s, 6H), 2.2 (tdd, *J* = 14.3, 12.0, 7.3 Hz, 4H), 1.3 (t, *J* = 7.4 Hz, 6H).

^{13}C NMR (126 MHz, CD_2Cl_2) δ 166.3, 166.3, 160.2, 149.0, 148.6, 144.1, 143.7, 142.5, 142.1, 139.5, 131.3, 131.2, 130.5, 130.4, 128.3, 127.8, 118.9, 113.0, 105.7, 104.2, 102.8, 56.0, 52.3, 52.3, 51.7, 20.1, 11.1.

^{19}F NMR (282 MHz, CD_2Cl_2) δ -153.11, -153.16.

IR (neat, cm^{-1}): ν 525.1, 537.2, 566.2, 596.7, 708.4, 772.0, 834.5, 1017.5, 1057.1, 1110.1, 1222.3, 1280.8, 1346.6, 1401.2, 1435.4, 1491.9, 1562.2, 1578.0, 1604.1, 1718.9, 2953.6, 3592.4

HRMS (ESI) calculated for $C_{51}H_{47}N_2O_8^+$ (M-BF4): m/z 815.3328, found: 815.3318 (-1.2 ppm)

M.P.: 195 °C (decomposition)

1,13-dimethoxy-3,7,11-tris(4-(methylsulfonyl)phenyl)-5,9-dipropyl-5,9-dihydro-13bH-quinolino[2,3,4-kl]acridin-13b-ylium tetrafluoroborate salt (6h)

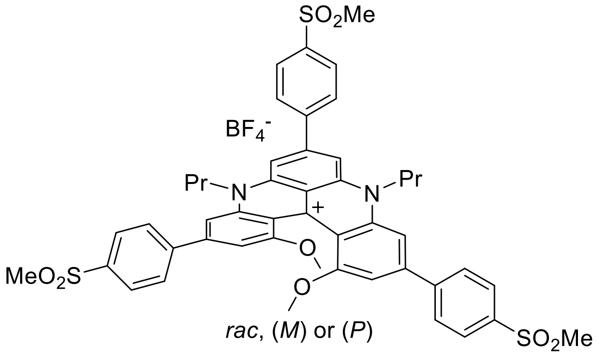

Compound **6h** is obtained following the general procedure for borylation and Suzuki cross-coupling. 1-bromo-4-(methylsulfonyl)benzene (212 mg, 0.9 mmol, 9 equiv) and sodium borohydride were used. Purification of the reduced product was performed with flash column chromatography (cyclohexane/ethyl acetate 30/70). After oxidation step in presence of BF_4^- anion (see general procedure), the expected product **6h** was purified with flash column chromatography (dichloromethane/MeOH 9/1, R_f = 0.5) giving a dark green solid, 27.4 mg, yield: 28%.

^{1}H NMR (500 MHz, CD_2Cl_2) δ 8.2 (ddd, *J* = 14.9, 8.3, 1.9 Hz, 6H), 8.1 – 8.0 (m, 6H), 7.6 (s, 2H), 7.6 (s, 2H), 7.2 (s, 2H), 4.8 (ddd, *J* = 16.5, 10.7, 5.9 Hz, 2H), 4.6 (ddd, *J* = 16.5, 11.2, 5.4 Hz, 2H), 3.9 (s, 6H), 3.1 (dd, *J* = 5.3, 2.0 Hz, 9H), 2.2 (ddt, *J* = 19.4, 13.7, 6.7 Hz, 4H), 1.3 (t, *J* = 7.4 Hz, 6H).

^{13}C NMR (126 MHz, CD_2Cl_2) δ 160.32, 148.27, 147.91, 145.14, 144.77, 142.51, 142.17, 141.59, 141.44, 139.53, 129.36, 128.78, 128.44, 128.30, 128.29, 119.07, 113.21, 106.14, 104.45, 102.88, 56.14, 51.69, 44.41, 20.14, 11.04.

^{19}F NMR (282 MHz, CD_2Cl_2) δ -152.46, -152.51.

IR (neat, cm^{-1}): ν 546.7, 604.4, 729.7, 772.7, 825.7, 957.6, 1056.7, 1149.9, 1207.6, 1306.7, 1346.5, 1395.9, 1486.3, 1507.1, 1561.9, 1578.6, 1607.4, 2931.1, 3619.5

HRMS (ESI) calculated for $C_{48}H_{47}N_2O_8S_3^+$ (M-BF4): m/z 875.2484, found: 875.2511 (3.1 ppm)

M.P.: 237 °C (decomposition)

1,13-dimethoxy-5,9-dipropyl-3,7,11-tri-p-tolyl-5,9-dihydro-13bH-quinolino[2,3,4-kl]acridin-13b-ylium tetrafluoroborate salt (6i)

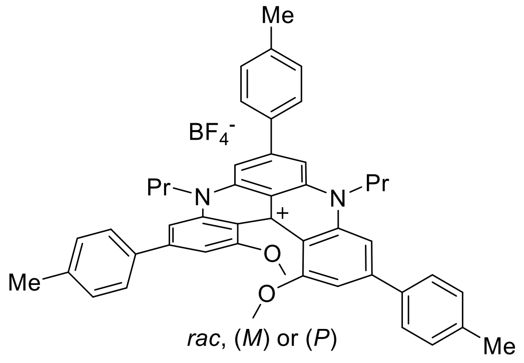

Compound **6i** is obtained following the general procedure for borylation and Suzuki cross-coupling. 4-bromotoluene (111 µL, 154 mg, 0.9 mmol, 9 equiv) and sodium borohydride were used. Purification of the reduced product was performed with flash column chromatography (cyclohexane/ethyl acetate 97/3). After oxidation step in presence of BF_4^- anion (see general procedure), the expected product **6i** was purified with flash column chromatography (dichloromethane/Acetone 9/1, R_f = 0.24) giving a dark green solid, 48 mg, yield: 58%.

^{1}H NMR (500 MHz, CD_2Cl_2) δ 7.8 – 7.7 (m, 6H), 7.6 (s, 2H), 7.5 – 7.4 (m, 8H), 7.1 (d, *J* = 1.2 Hz, 2H), 4.7 (ddd, *J* = 15.7, 10.8, 5.7 Hz, 2H), 4.5 (ddd, *J* = 15.7, 10.8, 5.6 Hz, 2H), 3.9 (s, 6H), 2.5 (s, 3H), 2.5 (s, 6H), 2.3 – 2.2 (m, 4H), 1.3 (t, *J* = 7.4 Hz, 6H).

^{13}C NMR (126 MHz, CD_2Cl_2) δ 160.11, 149.91, 149.45, 142.56, 141.86, 140.30, 140.19, 139.40, 137.01, 136.66, 130.23, 130.04, 127.95, 127.42, 112.31, 104.80, 103.61, 102.43, 55.81, 51.53, 21.04, 19.98, 11.11.

^{19}F NMR (282 MHz, CD_2Cl_2) δ -153.31, -153.36.

IR (neat, cm^{-1}): ν 501.4, 813.3, 1053.9, 1133.1, 1209.7, 1289.3, 1351.7, 1521.7, 1563.0, 1602.9, 2918.1

HRMS (ESI) calculated for $C_{48}H_{47}N_2O_2^+$ (M-BF4): m/z 683.3633, found: 683.3644 (1.6 ppm)

M.P.: 304 °C (decomposition)

3,7,11-tri([1,1'-biphenyl]-4-yl)-1,13-dimethoxy-5,9-dipropyl-5,9-dihydro-13bH-quinolino[2,3,4-kl]acridin-13b-ylium tetrafluoroborate salt (6j)

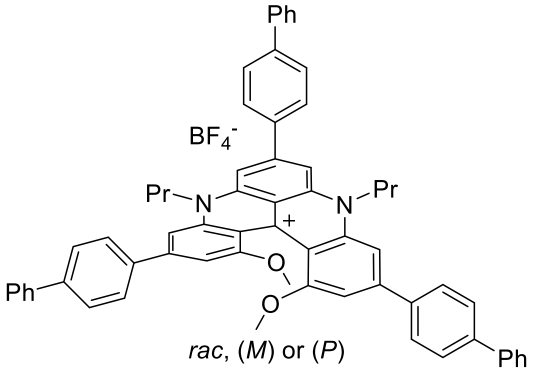

Compound **6j** is obtained following the general procedure for borylation and Suzuki cross-coupling. 4-bromobiphenyl (210 mg, 0.9 mmol, 9 equiv) and sodium borohydride were used. Purification of the reduced product was performed with flash column chromatography (cyclohexane/ethyl acetate 9/1). After oxidation step in presence of BF_4^- anion (see general procedure), the expected product **6j** was purified with flash column chromatography (dichloromethane/MeOH 95/5) followed by a final chromatography (dichloromethane/Acetone 95/5, R_f = 0.25) giving a dark green solid, 39.5 mg, yield: 41%.

^{1}H NMR (500 MHz, CD_2Cl_2) δ 8.0 (d, *J* = 8.0 Hz, 6H), 7.9 (dd, *J* = 12.4, 8.3 Hz, 6H), 7.7 (dd, *J* = 7.6, 2.0 Hz, 6H), 7.6 (s, 2H), 7.6 – 7.6 (m, 2H), 7.5 (td, *J* = 7.7, 3.3 Hz, 6H), 7.4 (ddt, *J* = 8.6, 6.6, 2.5 Hz, 3H), 7.2 (s, 2H), 4.8 (ddd, *J* = 16.1, 10.7, 5.8 Hz, 2H), 4.6 (ddd, *J* = 16.0, 11.0, 5.9 Hz, 2H), 3.9 (s, 6H), 2.3 (ddt, *J* = 17.8, 11.6, 6.8 Hz, 4H), 1.3 (t, *J* = 7.4 Hz, 6 H).

^{13}C NMR (126 MHz, CD_2Cl_2) δ 160.21, 149.50, 149.06, 142.61, 142.55, 142.44, 141.90, 139.88, 139.79, 139.48, 138.73, 138.39, 129.05, 129.02, 128.62, 128.09, 128.02, 127.90, 127.07, 127.04, 118.58, 112.60, 105.05, 103.77, 102.52, 55.93, 51.61, 20.07, 11.16.

^{19}F NMR (282 MHz, CD_2Cl_2) δ -153.16, -153.21.

IR (neat, cm^{-1}): ν 697.7, 765.7, 826.0, 1055.3, 1206.6, 1292.1, 1347.3, 1496.4, 1573.1, 1603.2, 2953.8

HRMS (ESI) calculated for $C_{63}H_{53}N_2O_2^+$ (M-BF4): m/z 869.4102, found: 869.4090 (-1.4 ppm)

M.P.: 308 °C (decomposition)

3,7,11-tri([1,1'-biphenyl]-2-yl)-1,13-dimethoxy-6-nitro-5,9-dipropyl-5,9-dihydro-13bH-quinolino[2,3,4-kl]acridin-13b-ylium tetrafluoroborate salt (6k)

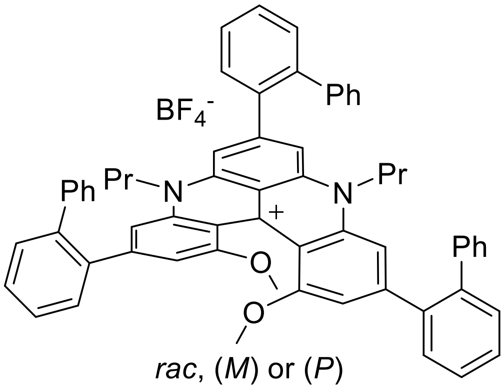

Compound **6k** is obtained following the general procedure for borylation and Suzuki cross-coupling. 2-bromobiphenyl (210 mg, 155 µL 0.9 mmol, 9 equiv) and sodium borohydride were used. Purification of the reduced product was performed with flash column chromatography (cyclohexane/ethyl acetate 95/5). After oxidation step in presence of BF_4^- anion (see general procedure), the expected product **6k** was purified with flash column chromatography (dichloromethane/acetone 85/15, R_f =0.32) giving a dark green solid, 36 mg, yield: 36%.

^{1}H NMR (500 MHz, CD_2Cl_2) δ 7.7 – 7.5 (m, 13H), 7.3 – 7.2 (m, 12H), 7.2 (ddd, J = 6.2, 2.7, 1.5 Hz, 2H), 7.1 (s, 2H), 7.0 (d, J = 1.3 Hz, 2H), 6.7 (d, J = 1.1 Hz, 2H), 4.1 (ddd, J = 15.1, 12.0, 5.4 Hz, 2H), 3.8 – 3.7 (m, 2H), 3.5 (s, 6H), 1.5 – 1.5 (m, 2H), 1.4 (dq, J = 13.3, 6.9, 6.2 Hz, 2H), 1.0 (t, J = 7.4 Hz, 6H).

^{13}C NMR (126 MHz, CD_2Cl_2) δ 159.16, 150.64, 149.84, 141.61, 141.44, 141.15, 141.08, 140.90, 140.84, 138.93, 138.89, 138.32, 131.36, 131.23, 130.84, 130.19, 129.85, 129.69, 129.54, 129.26, 128.53, 128.43, 128.35, 128.05, 127.14, 127.06, 117.94, 111.73, 108.55, 106.78, 105.53, 55.46, 51.39, 19.14, 11.02.

^{19}F NMR (282 MHz, CD_2Cl_2) δ -153.42, -153.48.

IR (neat, cm^{-1}): ν 702.9, 747.6, 840.2, 1054.8, 1215.3, 1345.8, 1493.5, 1571.6, 1607.0, 1943.0, 2169.0, 2927.8

HRMS (ESI) calculated for $C_{63}H_{53}N_2O_2^+$ (M-BF4): m/z 869.4102, found: 869.4090 (-1.4 ppm)

M.P.: 192 °C (decomposition)

3,7,11-tri(anthracen-9-yl)-1,13-dimethoxy-5,9-dipropyl-5,9-dihydro-13bH-quinolino[2,3,4-kl]acridin-13b-ylium tetrafluoroborate salt (6l)

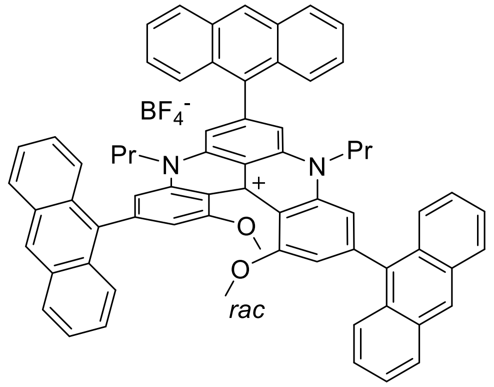

Compound **6l** was obtained following a modified procedure for Suzuki cross-coupling step:

In a dry 5 mL micro-wave vial with a stirrer are added 9-bromoanthracene (257 mg, 1 mmol, 10 equiv), tetrakis(triphenylphosphine)palladium (92 mg, 0.08 mmol, 0.8 equiv) and cesium carbonate (29 mg, 0.09 mmol, 9 equiv). Then the vial is sealed, put under vacuum and backfilled with nitrogen three times. Under N_2 atmosphere, 1 mL of dry 1,4-dioxane is added. The resulting mixture of borylation process is transferred via syringe into the Pd-containing vial and the reaction mixture is put in the oil bath at 80°C. The reaction is monitored by mass-spectroscopy analysis and the reaction is considered as complete when no more borylated species can be observed (around 23 h). After cooling to room temperature, the micro-wave vial is opened and solvents are removed under reduced pressure. The residue is dissolved in dichloromethane and washed with $NaBF_4$ (1M solution in water) and water. Organic layer is dried over Na_2SO_4 and solvents are removed under reduced pressure. The crude reaction mixture is the submitted to oxidation process under light irradiation in CH_2Cl_2/water mixture with $NaBF_4$ (i.e. 2-3 g) until TLC monitoring does not show any more reduced products. The irradiation should not exceed 1h30 to avoid photodegradation of the expected product. Organic layer is separated, dried over Na_2SO_4 and solvent are

removed under reduced pressure and the product is purified through flash column chromatography (dichloromethane/acetone 9/1, R_f= 0.37). The product is obtained as a green blueish solid, 51 mg, yield: 50%. This mixture of enantiomers are separated by chiral stationary phase HPLC (see "Chiral Stationary Phase (CSP) HPLC resolution" section for more details).

^{1}H NMR (500 MHz, CD_2Cl_2) δ 8.7 (s, 1H), 8.7 (s, 2H), 8.2 (ddd, *J* = 7.6, 5.3, 2.8 Hz, 6H), 7.8 (ddd, *J* = 18.8, 8.8, 1.2 Hz, 4H), 7.7 – 7.6 (m, 4H), 7.6 – 7.5 (m, 10H), 7.5 – 7.5 (m, 4H), 7.1 (d, *J* = 1.1 Hz, 2H), 4.6 (ddd, *J* = 16.2, 10.8, 6.1 Hz, 2H), 4.4 (ddd, *J* = 15.7, 10.7, 5.9 Hz, 2H), 3.9 (s, 6H), 2.2 – 2.0 (m, 4H), 0.9 (t, *J* = 7.4 Hz, 6H).

^{13}C NMR (126 MHz, CD_2Cl_2) δ 159.75, 149.43, 148.90, 142.59, 142.27, 139.23, 134.61, 134.54, 131.41, 131.30, 131.25, 129.86, 129.63, 129.54, 128.83, 128.80, 128.34, 128.08, 126.67, 126.55, 126.46, 125.63, 125.59, 125.56, 125.52, 125.47, 119.07, 112.95, 110.05, 108.32, 107.05, 56.12, 53.00, 51.57, 20.05, 10.70.

^{19}F NMR (282 MHz, CD_2Cl_2) δ -153.51, -153.56.

IR (neat, cm^{-1}): ν 673.0, 698.0, 737.6, 793.2, 848.9, 892.5, 916.2, 1057.0, 1125.7, 1163.1, 1183.9, 1206.4, 1251.6, 1336.4, 1374.3, 1417.0, 1458.4, 1483.0, 1566.9, 1606.9, 2853.3, 2925.1

HRMS (ESI) calculated for $C_{69}H_{53}N_2O_2^+$ (M-BF4): m/z 941.4102, found 941.4075 (-2.9 ppm)

M.P.: 276 °C

1,13-dimethoxy-5,9-dipropyl-3,7,11-tris(4-(1,2,2-triphenylvinyl)phenyl)-5,9-dihydro-13bH-quinolino[2,3,4-kl]acridin-13b-ylium tetrafluoroborate salt (6m)

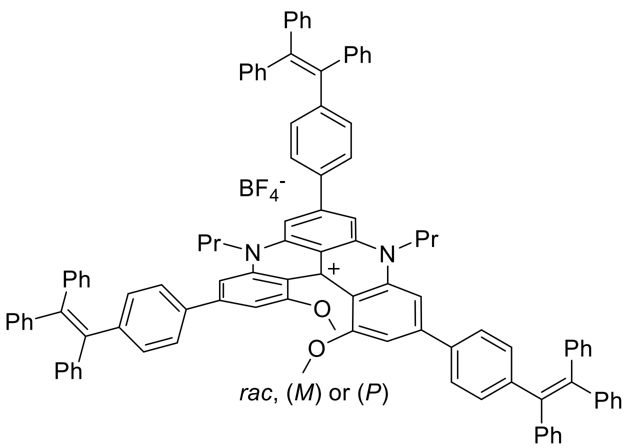

Compound **6m** is obtained following the general procedure for borylation and Suzuki cross-coupling. 1-(4-bromophenyl)-1,2,2-triphenylethylene (370 mg, 0.9 mmol, 9 equiv) and sodium borohydride were used. Purification of the reduced product was performed with flash column chromatography (cyclohexane/ethyl acetate 95/5). After oxidation step in presence of BF_4^- anion (see general procedure), the expected product **6m** was purified with flash column chromatography (dichloromethane/acetone 9/1, R_f = 0.29) giving a dark green solid, 52 mg, yield: 35%.

^{1}H NMR (500 MHz, CD_2Cl_2) δ 7.6 – 7.6 (m, 6H), 7.5 (s, 2H), 7.4 (s, 2H), 7.3 – 7.3 (m, 6H), 7.2 – 7.1 (m, 39H), 7.1 – 7.0 (m, 8H), 4.7 (ddd, *J* = 16.2, 10.6, 5.9 Hz, 2H), 4.5 (ddd, *J* = 15.9, 10.8, 5.9 Hz, 2H), 3.8 (s, 6H), 2.2 – 2.1 (m, 4H), 1.2 (t, *J* = 7.4 Hz, 6H).

^{13}C NMR (126 MHz, CD_2Cl_2) δ 160.06, 149.26, 148.77, 145.71, 145.62, 143.55, 143.44, 142.52, 142.35, 142.21, 141.62, 139.98, 139.98, 139.86, 139.37, 137.40, 137.13, 132.34, 132.16, 131.25, 131.21, 131.11, 127.90, 127.87, 127.84, 127.71, 127.28, 126.78, 126.72, 126.69, 118.49, 112.52, 104.74, 103.47, 102.26, 55.81, 51.38, 20.00, 11.10.

^{19}F NMR (282 MHz, CD_2Cl_2) δ -153.33, -153.38.

IR (neat, cm^{-1}): ν 575.7, 626.7, 699.3, 752.0, 819.8, 1073.9, 1208.0, 1290.6, 1346.7, 1444.5, 1490.8, 1516.0, 1573.6, 1602.0, 2925.0, 3052.7

HRMS (ESI) calculated for $C_{105}H_{83}N_2O_2^+$ (M-BF4): m/z 1403.6450, found 1403.6457 (0.5 ppm)

M.P.: 195 °C (decomposition)

1,13-dimethoxy-3,7,11-tris(4-(methylthio)phenyl)-5,9-dipropyl-5,9-dihydro-13bH-quinolino[2,3,4-kl]acridin-13b-ylium tetrafluoroborate salt (6n)

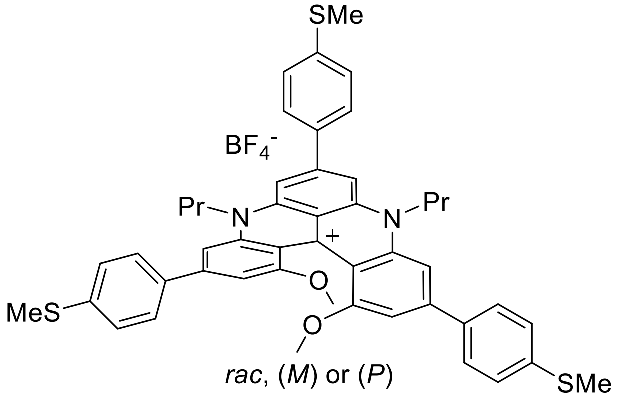

Compound **6n** is obtained following the general procedure for borylation and Suzuki cross-coupling. 4-bromothioanisole (183 mg, 0.9 mmol, 9 equiv) and sodium borohydride were used. Purification of the reduced product was performed with flash column chromatography (cyclohexane/ethyl acetate 95/5). After oxidation step in presence of BF_4^- anion (see general procedure), the expected product **6n** was purified with flash column chromatography (dichloromethane/MeOH 95/5, R_f = 0.29) giving a dark green solid, 26.2 mg, yield: 30%.

^{1}H NMR (500 MHz, CD_2Cl_2) δ 7.8 – 7.7 (m, 6H), 7.5 (s, 2H), 7.5 – 7.4 (m, 8H), 7.1 (d, *J* = 1.2 Hz, 2H), 4.7 (ddd, *J* = 16.2, 10.8, 5.9 Hz, 2H), 4.5 (ddd, *J* = 15.7, 10.7, 5.7 Hz, 2H), 3.9 (s, 6H), 2.6 (s, 3H), 2.6 (s, 6H), 2.3 – 2.2 (m, 4H), 1.3 (t, *J* = 7.4 Hz, 6H).

^{13}C NMR (126 MHz, CD_2Cl_2) δ 160.16, 149.25, 148.81, 142.59, 141.88, 141.64, 139.45, 136.00, 135.69, 128.34, 127.83, 126.56, 126.42, 118.41, 112.42, 104.60, 103.41, 102.19, 55.83, 51.52, 19.99, 15.10, 11.11.

^{19}F NMR (282 MHz, CD_2Cl_2) δ -153.27, -153.32.

IR (neat, cm^{-1}): ν 579.1, 815.1, 1055.4, 1098.5, 1132.9, 1204.8, 1289.7, 1348.2, 1398.5, 1507.3, 1553.5, 1573.3, 1603.0, 2924.5

HRMS (ESI) calculated for $C_{48}H_{47}N_2O_2S_3^+$ (M-BF4): m/z 779.2795, found: 779.2815 (-2.6 ppm)

M.P.: 189 °C (decomposition)

1,13-dimethoxy-3,7,11-tris(4-methoxyphenyl)-5,9-dipropyl-5,9-dihydro-13bH-quinolino[2,3,4-kl]acridin-13b-ylium tetrafluoroborate salt (6o)

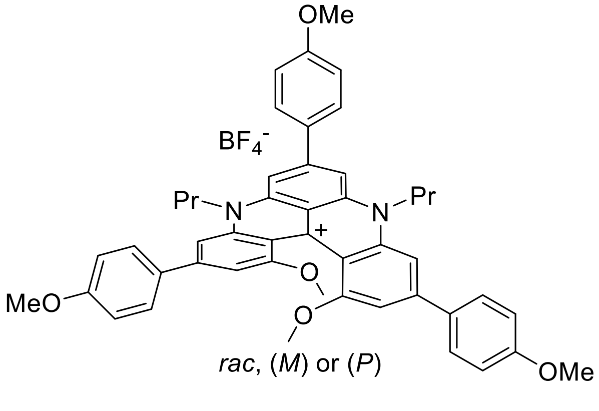

Compound **6o** is obtained following the general procedure for borylation and Suzuki cross-coupling. 4-bromoanisole (112 µL, 168 mg, 0.9 mmol, 9 equiv) and sodium borohydride were used. Purification of the reduced product was performed with flash column chromatography (cyclohexane/ethyl acetate 9/1). After oxidation step in presence of BF_4^- anion (see general procedure), the expected product **6o** was purified with flash column chromatography (dichloromethane/MeOH

9/1, R_f = 0.58) giving a dark green solid, 23.4 mg, yield: 29%.

^{1}H NMR (500 MHz, CD_2Cl_2) δ 7.8 (d, *J* = 8.3 Hz, 6H), 7.5 (s, 2H), 7.4 (s, 2H), 7.2 (dd, *J* = 11.6, 8.7 Hz, 6H), 7.1 (s, 2H), 4.7 (ddd, *J* = 16.2, 10.7, 5.9 Hz, 2H), 4.5 (ddd, *J* = 15.9, 10.8, 5.7 Hz, 2H), 3.9 (d, *J* = 2.4 Hz, 9H), 3.9 (s, 6H), 2.3 – 2.2 (m, 4H), 1.3 (t, *J* = 7.4 Hz, 6H).

^{13}C NMR (126 MHz, CD_2Cl_2) δ 161.18, 160.12, 149.39, 148.93, 142.61, 141.64, 139.42, 132.06, 131.75, 129.37, 129.35, 128.85, 118.15, 114.95, 114.73, 112.08, 104.26, 103.23, 102.12, 55.77, 55.58, 55.55, 51.44, 19.96, 11.12.

^{19}F NMR (282 MHz, CD_2Cl_2) δ -153.25, -153.31.

IR (neat, cm^{-1}): ν 602.4, 823.1, 1031.2, 1054.1, 1132.1, 1180.0, 1208.4, 1254.0, 1293.3, 1348.3, 1408.6, 1460.2, 1520.8, 1563.8, 1580.2, 1597.4, 2853.4, 2924.8

HRMS (ESI) calculated for $C_{48}H_{47}N_2O_5^+$ (M-BF4): m/z 731.3480, found: 731.3460 (-2.7 ppm)

M.P.: 232 °C (decomposition)

1,13-dimethoxy-5,9-dipropyl-3,7,11-tris(3,4,5-trimethoxyphenyl)-5,9-dihydro-13bH-quinolino[2,3,4-kl]acridin-13b-ylium tetrafluoroborate salt (6p)

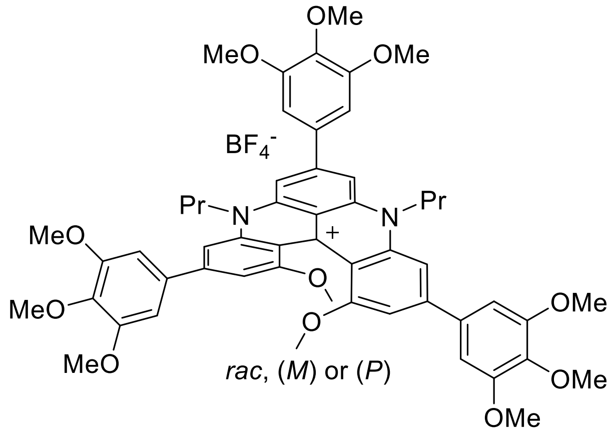

Compound **6p** is obtained following the general procedure for borylation and Suzuki cross-coupling. 5-bromo-1,2,3-trimethoxybenzene (222 mg, 0.9 mmol, 9 equiv) and sodium borohydride were used. Purification of the reduced product was performed with flash column chromatography (cyclohexane/ethyl acetate 7/3). After oxidation step in presence of BF_4^- anion (see general procedure), the expected product **6p** was purified with flash column chromatography (dichloromethane/MeOH 93/7, R_f = 0.43) giving a dark green solid, 19.9 mg, yield: 20%.

^{1}H NMR (500 MHz, CD_2Cl_2) δ 7.5 (s, 2H), 7.4 (d, *J* = 1.4 Hz, 2H), 7.1 (d, *J* = 1.4 Hz, 2H), 7.0 (s, 2H), 7.0 (s, 4H), 4.8 (ddd, *J* = 16.1, 10.7, 5.9 Hz, 2H), 4.6 (ddd, *J* = 15.9, 10.6, 5.7 Hz, 2H), 4.0 (s, 18H), 3.9 (s, 6H), 3.9 (s, 3H), 3.9 (s, 6H), 2.3 – 2.2 (m, 4H), 1.3 (t, *J* = 7.4 Hz, 6H).

^{13}C NMR (126 MHz, CD_2Cl_2) δ 160.08, 154.09, 153.98, 150.14, 149.64, 142.44, 141.98, 139.85, 139.79, 139.32, 135.70, 135.41, 118.45, 112.48, 105.74, 105.24, 105.18, 103.84, 102.67, 60.68, 56.48, 55.99, 51.44, 20.05, 11.12.

^{19}F NMR (282 MHz, CD_2Cl_2) δ -152.71, -152.76.

IR (neat, cm^{-1}): ν 565.9, 823.4, 1056.5, 1128.0, 1168.6, 1243.6, 1333.4, 1405.3, 1464.9, 1508.7, 1566.4, 1604.9, 2939.1

HRMS (ESI) calculated for $C_{54}H_{59}N_2O_{11}^+$ (M-BF4): m/z 1136.4899, found: 1136.4895 (-0.4 ppm)

M.P.: 128 °C (decomposition)

3,7,11-tris(4-(9H-carbazol-9-yl)phenyl)-1,13-dimethoxy-5,9-dipropyl-5,9-dihydro-13bH-quinolino[2,3,4-kl]acridin-13b-ylium tetrafluoroborate salt (6q)

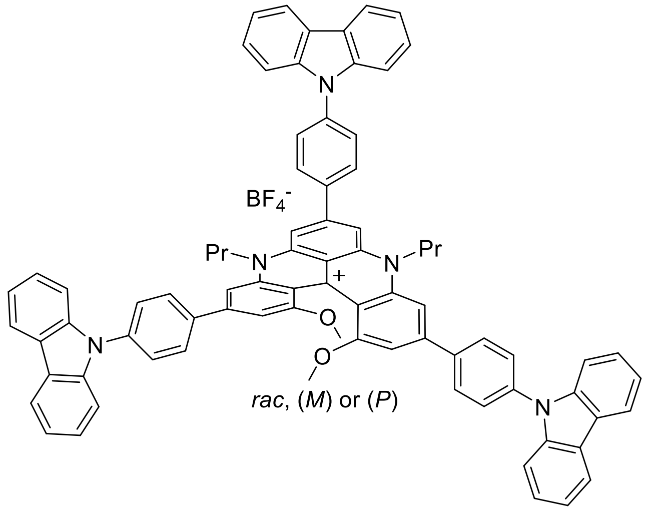

Compound **6q** is obtained following the general procedure for borylation and Suzuki cross-coupling. 9-(4-Bromophenyl)carbazole (290 mg, 0.9 mmol, 9 equiv) and sodium borohydride were used. Purification of the reduced product was performed with flash column chromatography (cyclohexane/ethyl acetate 95/5). After oxidation step in presence of BF_4^- anion (see general procedure), the expected product **6q** was purified with flash column chromatography (dichloromethane/acetone 87.5/12.5, R_f = 0.31) giving a dark green solid, 18.8 mg, yield: 15%.

^{1}H NMR (500 MHz, CD_2Cl_2) δ 8.2 (dd, *J* = 7.8, 1.1 Hz, 4H), 8.1 (td, *J* = 10.5, 8.2 Hz, 8H), 7.9 (dd, *J* = 10.6, 8.3 Hz, 6H), 7.7 (s, 2H), 7.6 (d, *J* = 1.2 Hz, 2H), 7.6 (dd, *J* = 8.2, 7.1 Hz, 6H), 7.5 (ddt, *J* = 8.2, 7.1, 1.2 Hz, 6H), 7.4 – 7.3 (m, 6H), 7.3 (d, *J* = 1.2 Hz, 2H), 4.8 (ddd, *J* = 16.2, 11.0, 5.8 Hz, 2H), 4.6 (ddd, *J* = 30.9, 20.1, 4.9 Hz, 2H), 4.0 (s, 6H), 2.4 – 2.3 (m, 4H), 1.4 (t, *J* = 7.3 Hz, 6H).

^{13}C NMR (126 MHz, CD_2Cl_2) δ 160.33, 149.17, 148.70, 142.66, 141.96, 140.56, 140.50, 139.55, 139.24, 139.14, 138.62, 138.35, 129.77, 129.24, 127.67, 127.54, 126.18, 123.65, 120.41, 118.69, 112.77, 109.72, 105.34, 103.96, 102.69, 56.04, 51.72, 20.12, 11.22.

^{19}F NMR (282 MHz, CD_2Cl_2) δ -153.04, -153.09.

IR (neat, cm^{-1}): ν 524.0, 539.6, 567.3, 600.7, 622.7, 724.0, 748.7, 822.6, 932.3, 1050.2, 1133.0, 1171.7, 1225.5, 1289.3, 1335.4, 1449.1, 1478.2, 1520.4, 1573.4, 1599.6, 170.9, 2167.7, 2929.5, 3050.3

HRMS (ESI) calculated for $C_{81}H_{62}N_5O_2^+$ (M-BF4): m/z 1136.4899, found: 1136.4895 (-0.4 ppm)

M.P.: 243 °C (decomposition)

3,7,11-tris(4-fluorophenyl)-1,13-dimethoxy-6-nitro-5,9-dipropyl-5,9-dihydro-13bH-quinolino[2,3,4-kl]acridin-13b-ylium tetrafluoroborate salt (9)

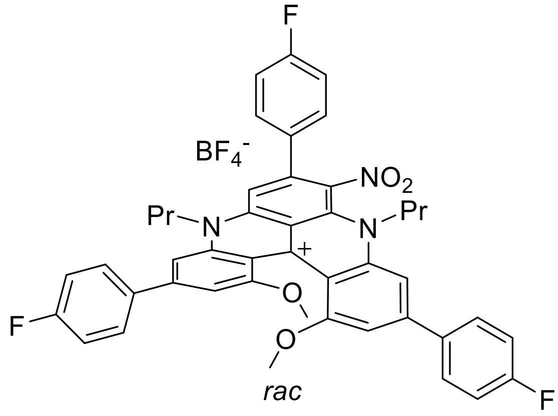

Compound **9** is obtained following the following procedure:

Compound **9** (20 mg, 26 µmol) was solubilized in methylene chloride. Then HNO_3 65% in water (100 µL, 47 equiv) was added to the solution. The reaction was let stir for 15-20 min at room temperature until the starting material was entirely consumed. Reaction mixture was quenched with aqueous NaOH (1 M). The biphasic solution was extracted with methylene chloride 3 times, organic layers were gather and washed with aqueous HBF_4 (1 M) and $NaBF_4$ (0.2 M), dry over Na_2SO_4 and solvents were removed

under vaccum. The expected product was purified with flash column chromatography (dichloromethane/MeOH 95/5, R_f = 0.26) giving a brown reddish solid, 15.4 mg, yield 65 %

^{1}H NMR (500 MHz, CD_2Cl_2) δ 7.9 – 7.9 (m, 2H), 7.9 – 7.8 (m, 2H), 7.6 (d, *J* = 1.4 Hz, 1H), 7.5 – 7.4 (m, 3H), 7.4 (s, 1H), 7.4 – 7.2 (m, 7H), 7.2 (d, *J* = 1.4 Hz, 1H), 4.9 – 4.7 (m, 2H), 4.7 – 4.6 (m, 1H), 4.0 (s, 3H), 3.9 (s, 3H), 3.8 (dt, *J* = 14.8, 7.6 Hz, 1H), 2.3 – 2.2 (m, 2H), 2.0 – 1.8 (m, 2H), 1.2 (t, *J* = 7.4 Hz, 3H), 0.6 (t, *J* = 7.3 Hz, 3H).

^{13}C NMR (126 MHz, CD_2Cl_2) δ 164.1 (d, *J* = 251.6 Hz), 163.9 (d, *J* = 250.6 Hz), 163.5 (d, *J* = 250.0 Hz), 160.2, 159.4, 150.9, 149.3, 143.5, 142.6, 142.2, 141.1, 138.2, 135.3, 135.3, 134.9, 133.3, 132.6, 132.6, 129.9 (d, *J* = 8.7 Hz), 129.8 (d, *J* = 8.7 Hz), 129.6 (d, *J* = 8.6 Hz), 119.6, 116.5 (d, *J* = 20.2 Hz), 116,3 (d, *J* = 19.7 Hz), 116.1 (d, *J* = 21.7 Hz), 115.9, 113.3, 108.4, 107.6, 105.3, 104.3, 104.0, 56.4, 56.2, 54.9, 52.1, 22.1, 20.6, 11.0, 10.4.

^{19}F NMR (282 MHz, CD_2Cl_2) δ -111.27, -111.70, -111.73, -153.01, -153.06.

IR (neat, cm^{-1}): ν 598.2, 830.9, 1055.1, 1162.6, 1235.8, 1291.6, 1345.8, 1517.4, 1570.6, 1599.2, 2936,7

HRMS (ESI) calculated for $C_{45}H_{37}F_3N_3O4^+$ (M-BF4): m/z 740.2731, found: 740.2737 (0.8 ppm)

M.P.: 210 °C

Chiral Stationary Phase (CSP) HPLC

<u>DMQA 1: enantiomeric excess analysis</u>

Conditions:
Columns: CHIRALPAK IC analytic

Mobile phase:

A: MeOH/EtOH (50:50) + Et_3N 0.4% +TFA 0.6%

Elution mixture : A (100%)

Elution : 1 mL/min, 25°C

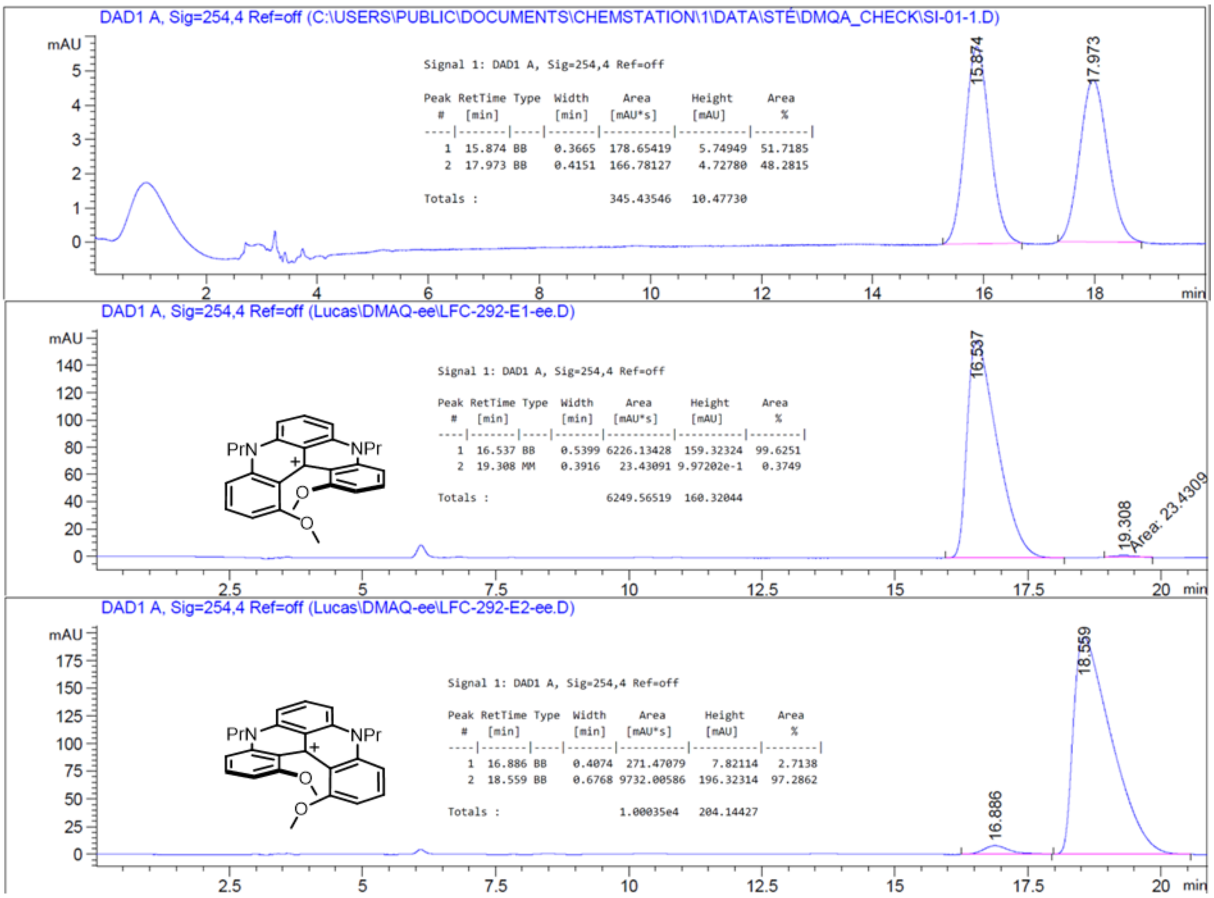

Figure S3: CSP-HPLC chromatograms of *rac*-**1** (first chromatogram) and (*M*)-**1** (second chromatogram) and (*P*)-**1** (third chromatogram). (*M*)- and (*P*)- enantiomers are resolved through chemical resolution and results are in accordance with ref S6.

Tris(arene) **6j**: enantiomeric excess analysis

Conditions:
Columns: CHIRALPAK IB analytic

Mobile phase:

A: MeOH/EtOH (50:50) + TFA 0.3% +TEA 0.5%

Elution mixture : A (100%)

Elution : 1 mL/min, 25°C

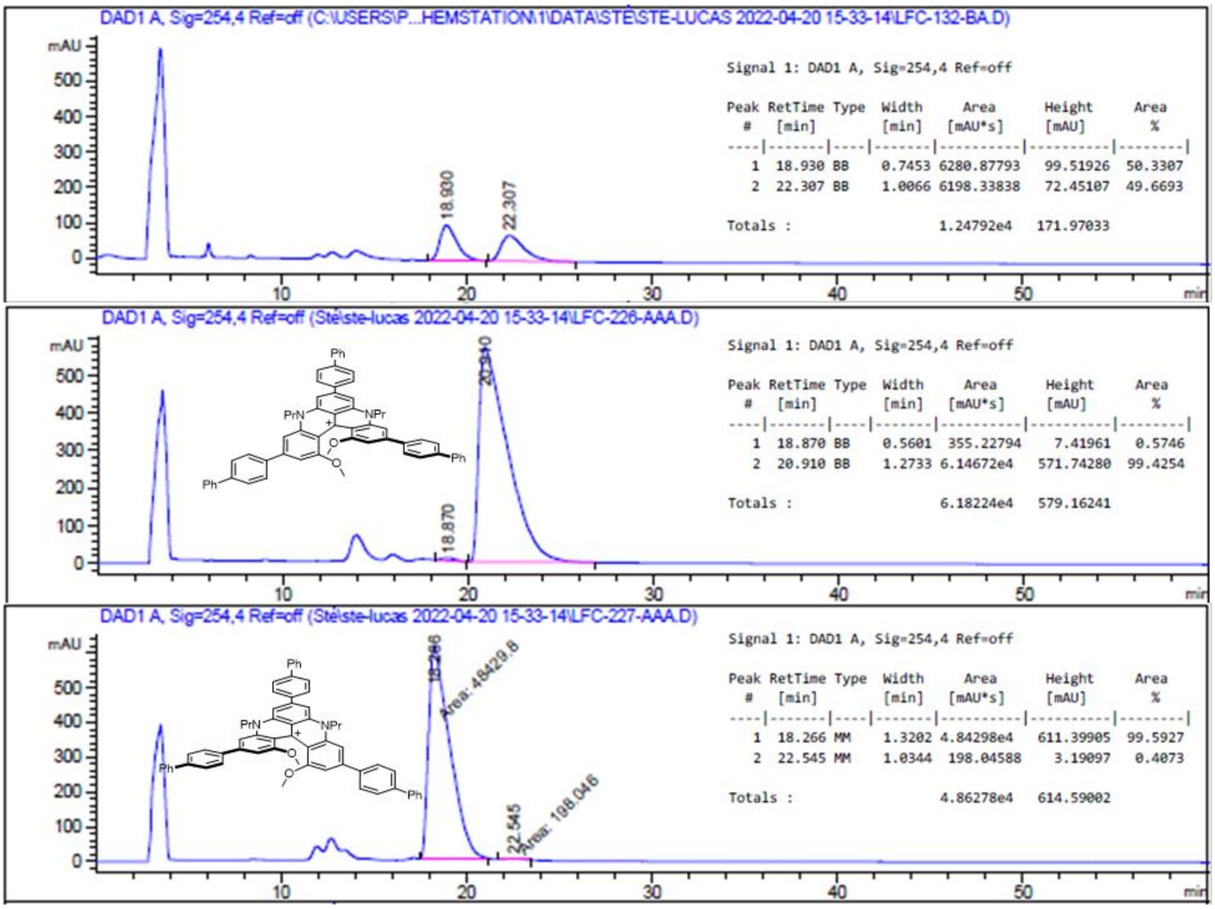

Figure S4: CSP-HPLC chromatograms of *rac*-**6j** (first chromatogram) and (*M*)-**6j** (second chromatogram) and (*P*)-**6j** (third chromatogram). (*M*)- and (*P*)- enantiomers are obtained with corresponding enantiopure (*M*)-/(*P*)-**1**.

CSP-HPLC resolution of tris(arene) **6l**

Conditions:
Columns: CHIRALPAK IC analytic and semi-preparative.

Mobile phase:

A: MeOH/EtOH (50:50) + 0.1% DEA (diethylamine)

B: Acetonitrile

Elution mixture A/B: 9/1

Elution: 1 mL/min, 25 °C (for semi-preparative) or 1 mL/min, 25 °C (for analytical).

Due to the presence of DEA in the mobile phase, the separated enantiomer solutions were evaporated, dissolved in CH_2Cl_2 and washed three times with 1 M aq HBF_4 and once with 1.5 M aq NaOH. The organic layer was next dried over Na_2SO_4, filtered and evaporated.

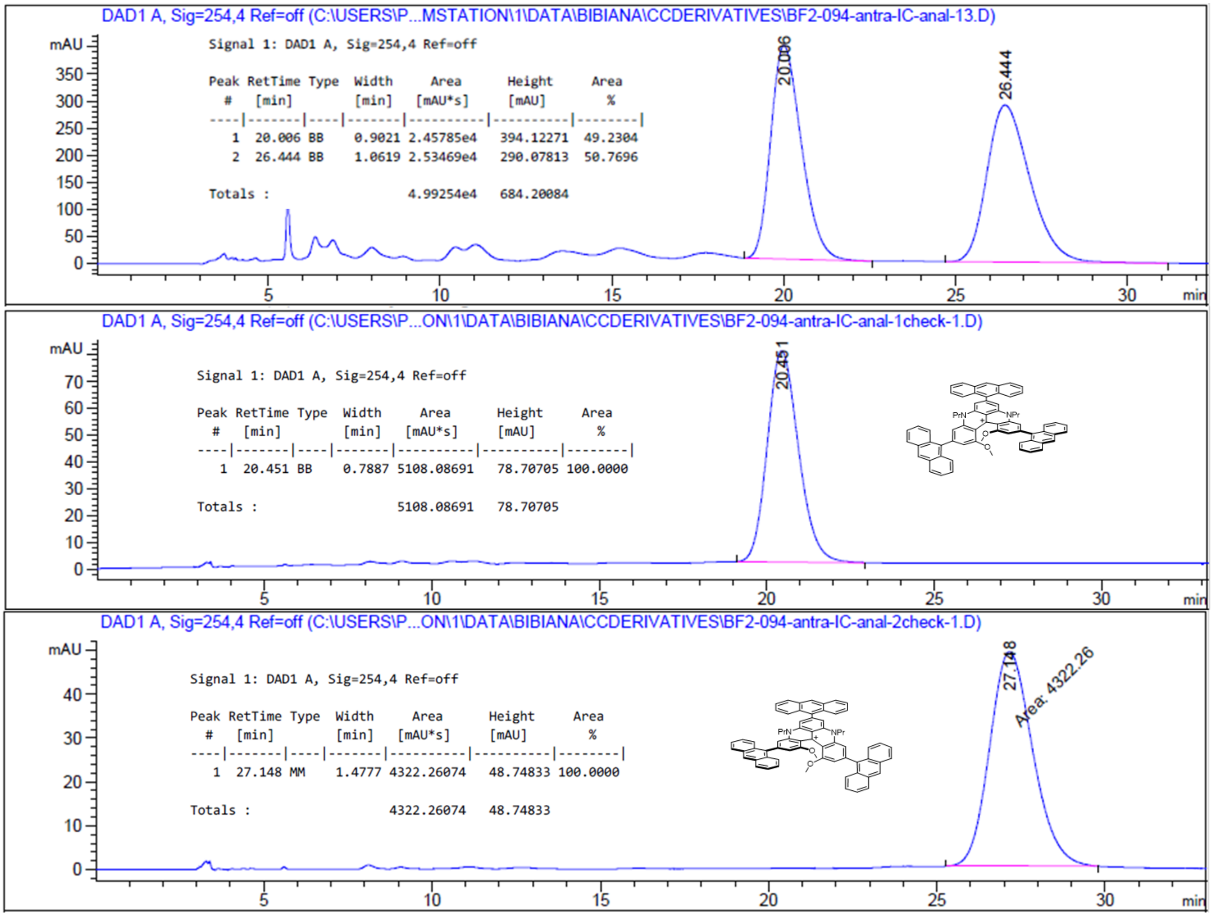

Figure S5: HPLC chromatograms of *rac*- (first chromatogram), (*M*)-**6l** (second chromatogram) and (*P*)-**6l** (third chromatogram) recorded after HPLC resolution of *rac*-**6l**

According to ECD spectra, and in comparison with the other compounds, first enantiomer eluted correspond to (*M*)-**6l** and *s*econd enantiomer eluted to (*P*)-**6l**.

NMR and IR spectra, and mass spectroscopy

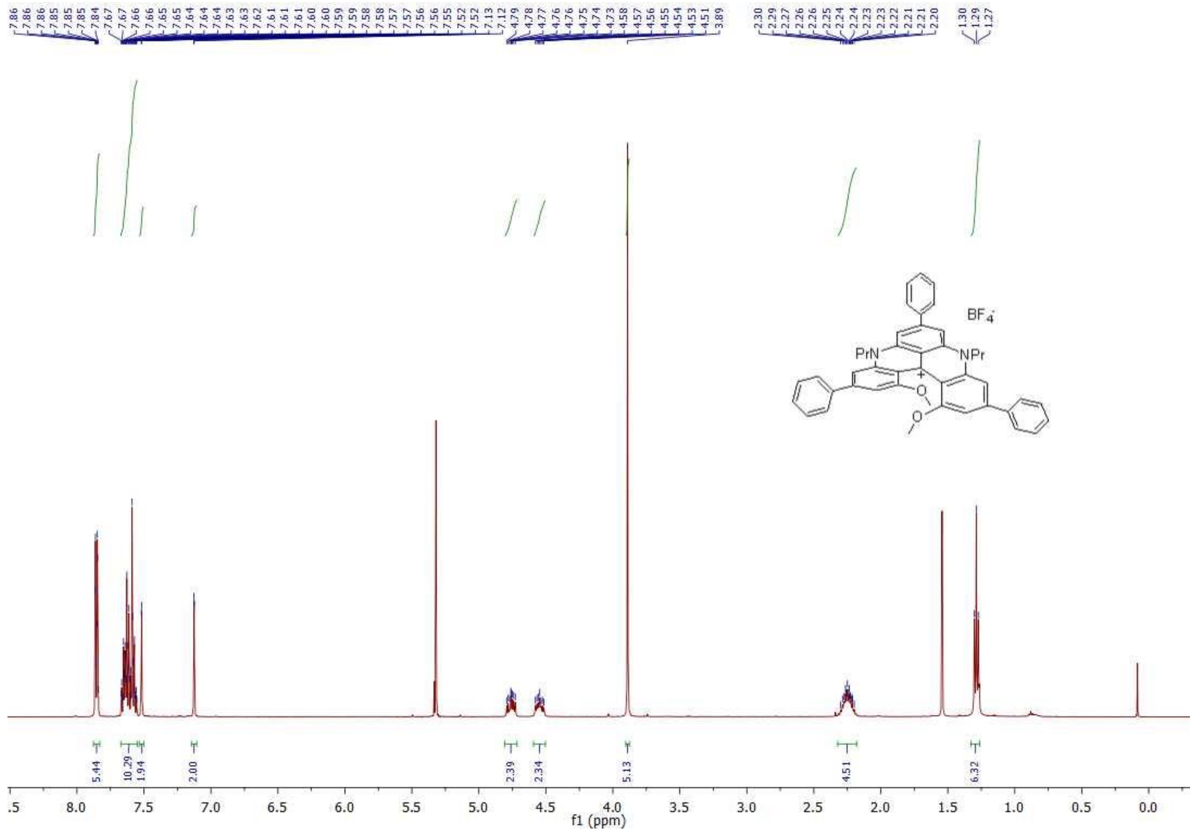

Figure S6. ^{1}H-NMR spectra of **6a** in CD_2Cl_2, 500 MHz.

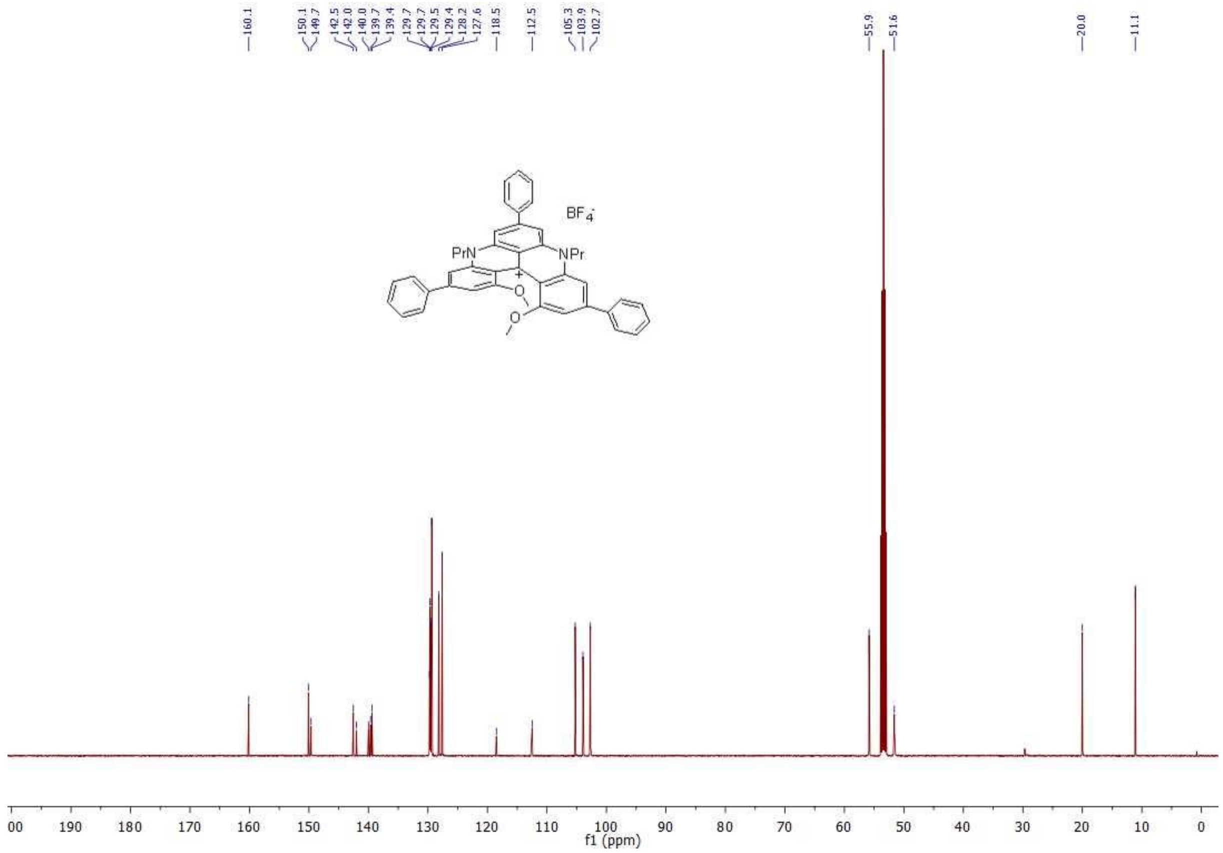

Figure S7. ^{13}C-NMR spectra of **6a** in CD_2Cl_2, 125 MHz.

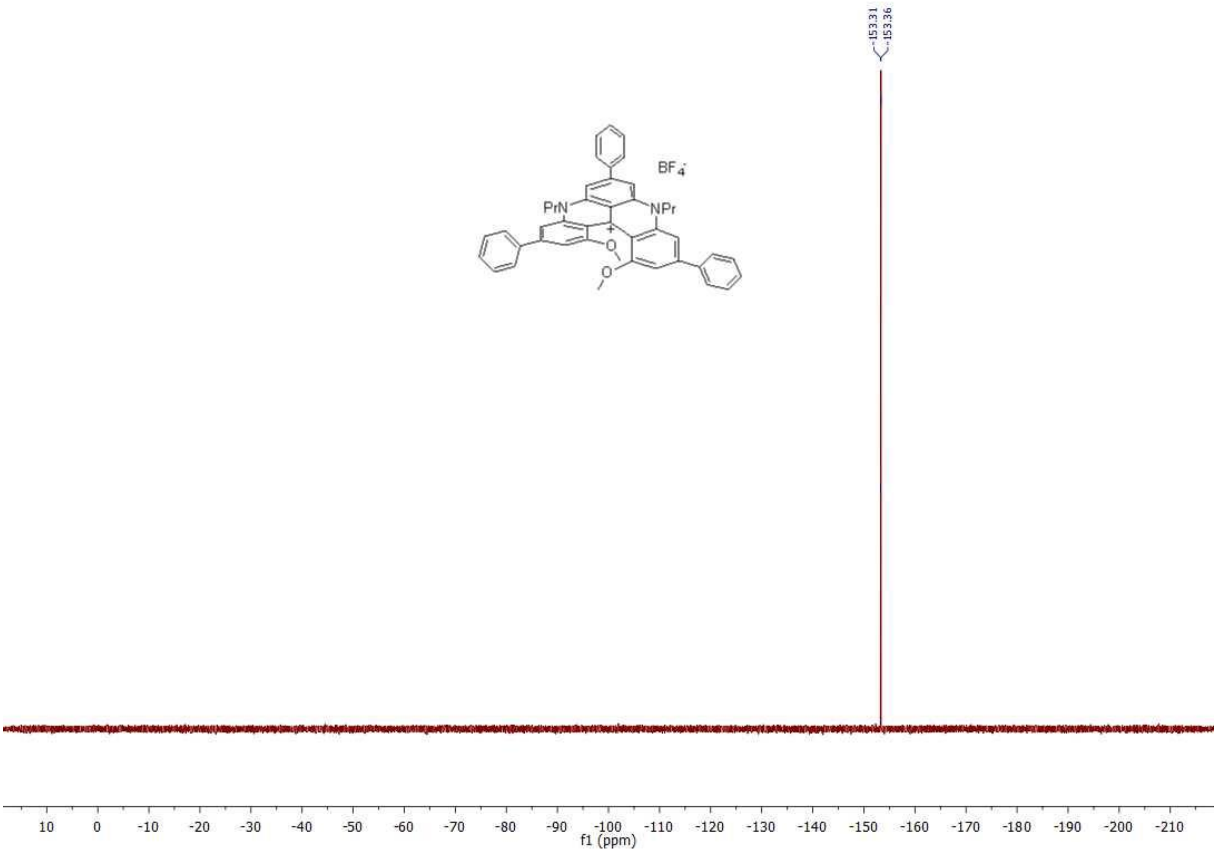

Figure S8. ^{19}F-NMR spectra of **6a** in CD_2Cl_2, 282 MHz.

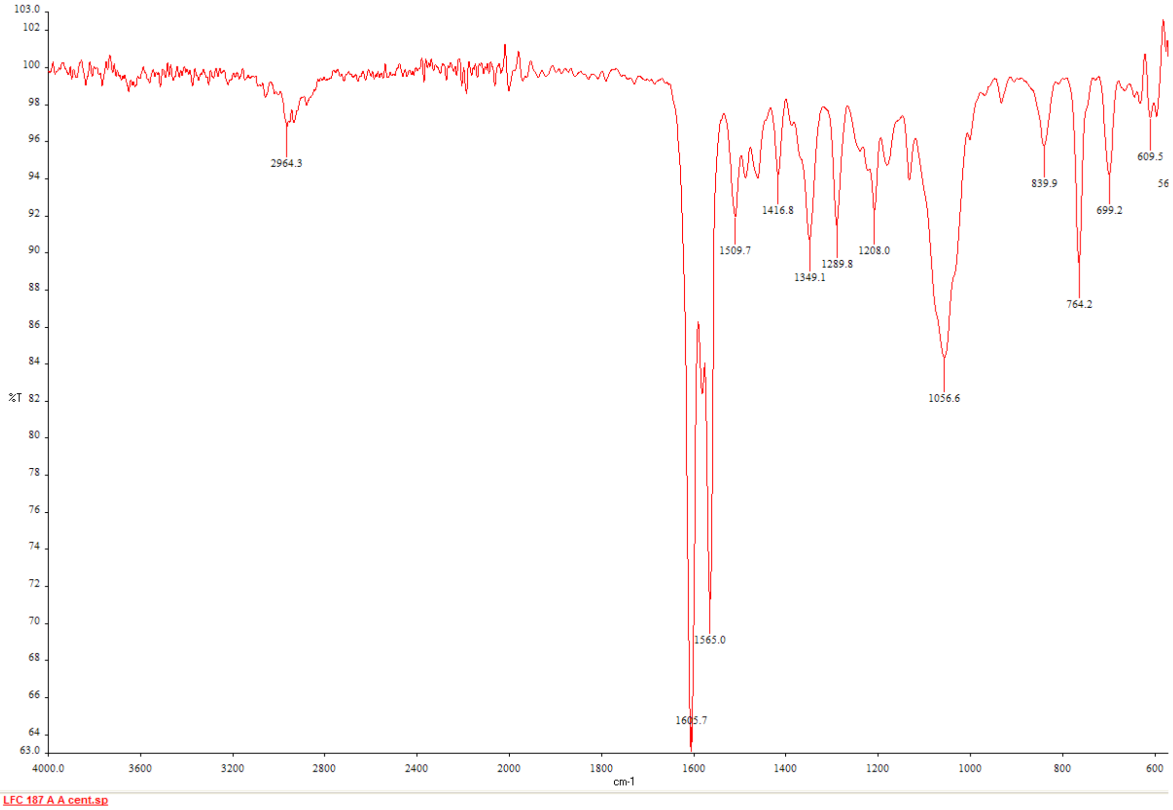

Figure S9. IR spectrum (neat) of **6a**.

Mass Spectrometry Core Facility

Lacour Group – University of Geneva

UNIVERSITÉ DE GENÈVE
FACULTÉ DES SCIENCES

ESI-HRMS – Certificate of Analysis

Applicant:	Lucas Frederic	Date of certificate:	29/07/21
Sample name:	LFC-187	Instrument:	Xevo G2 Tof (TOF)
Folder:	290721.PRO	Mobile phase:	MeOH (100 µl/min)
Analyst:	Stéphane Grass	Ionisation mode:	ESI (positive polarity)

Elemental Formula	Ion type	*Masslynx values **** calc. m/z	*Masslynx values **** meas. m/z	Calc. *m/z*	Meas. *m/z*	Accuracy [a] *(ppm)*
$C_{45}H_{41}N_2O_2$	$[M]^+$	*641.3168*	*641.3188*	641.3163	641.3183	3.1

[a] *Mass spectrum is calibrated by the use of the MS lockspray system (LeuEnk calibration solution).*

**** MassLynx software does not take into account the mass of the electron for ionic species, therefore the shift of m/z 0.000459.*

Zoomed mass spectrum – Isotopic distribution.

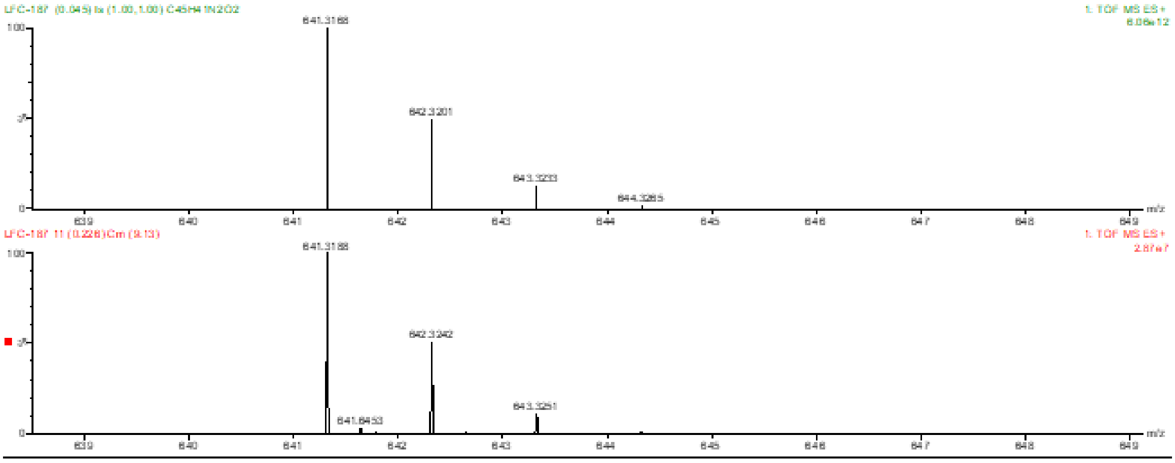

Figure S10. HRMS analysis (ESI, MeOH) report of **6a**.

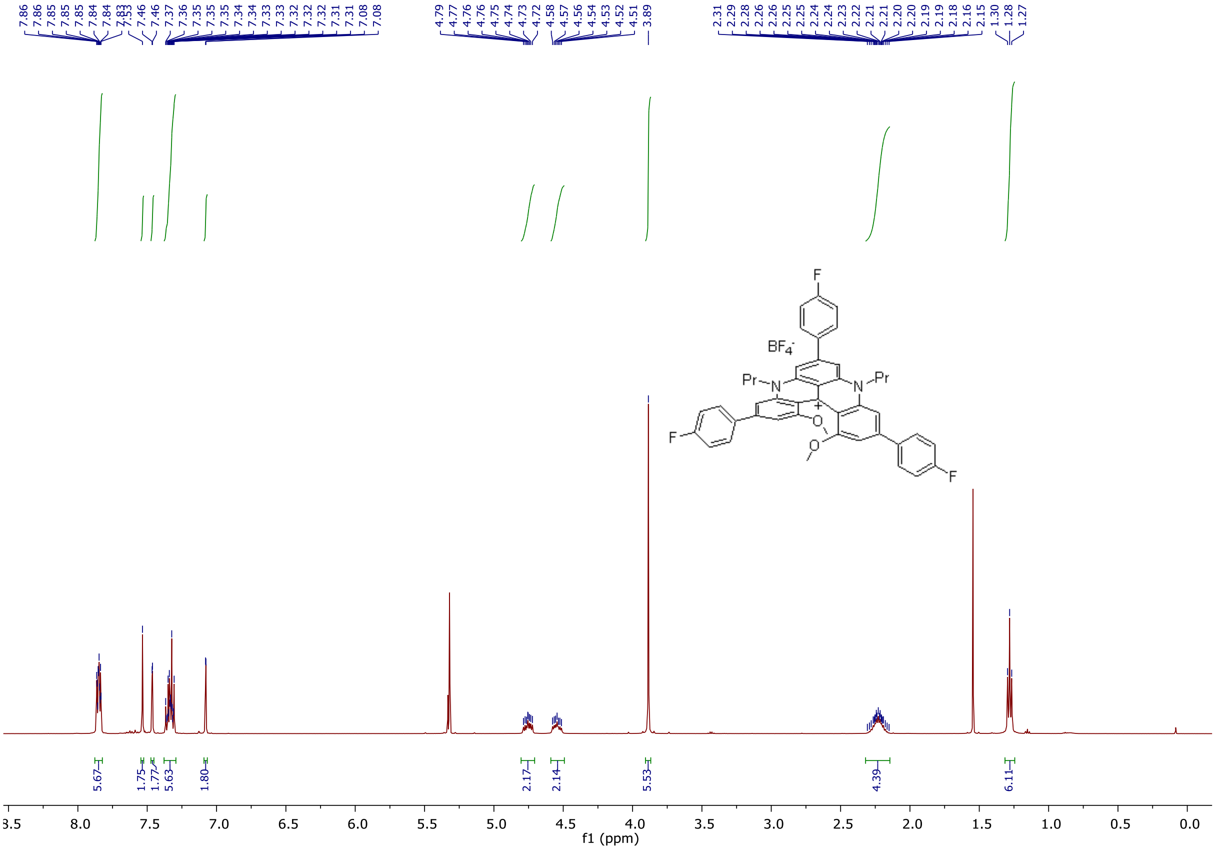

Figure S11. ^{1}H-NMR spectra of **6b** in CD_2Cl_2, 500 MHz.

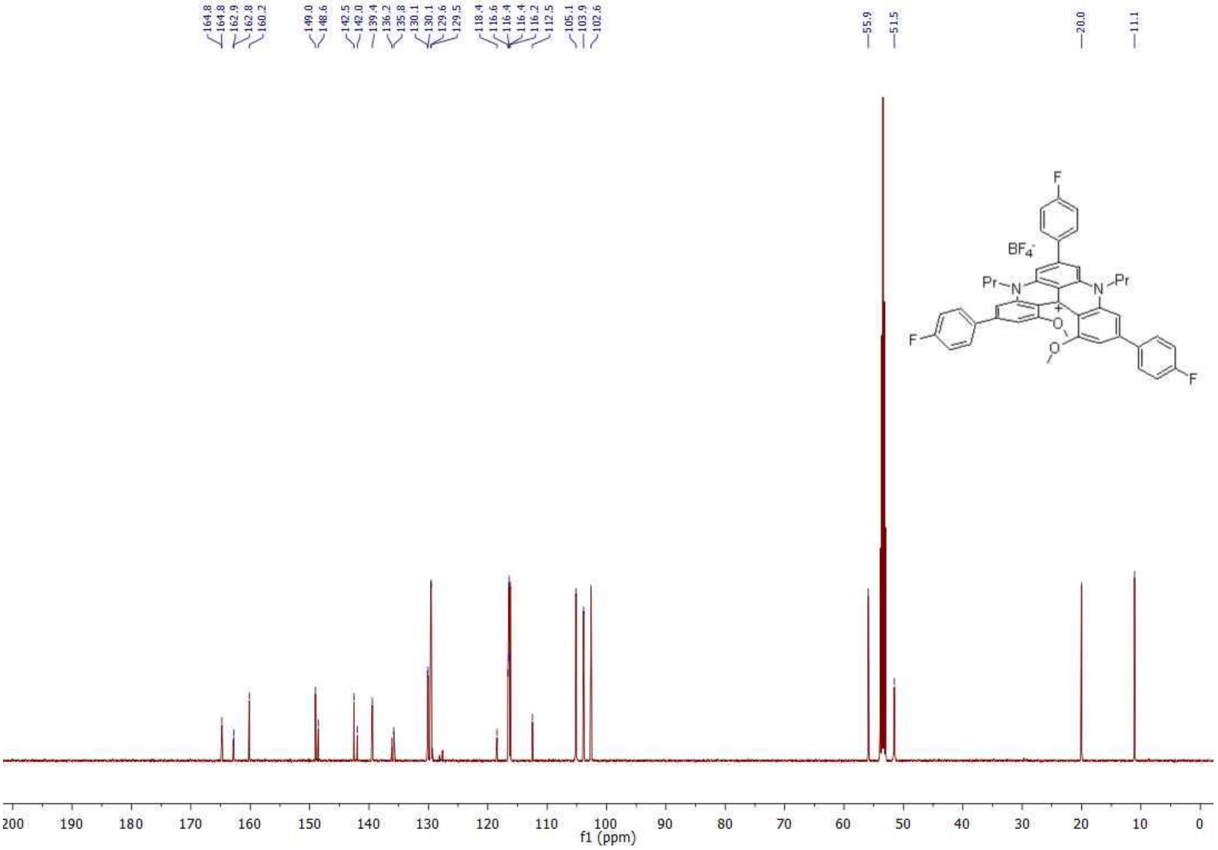

Figure S12. ^{13}C-NMR spectra of **6b** in CD_2Cl_2, 125 MHz.

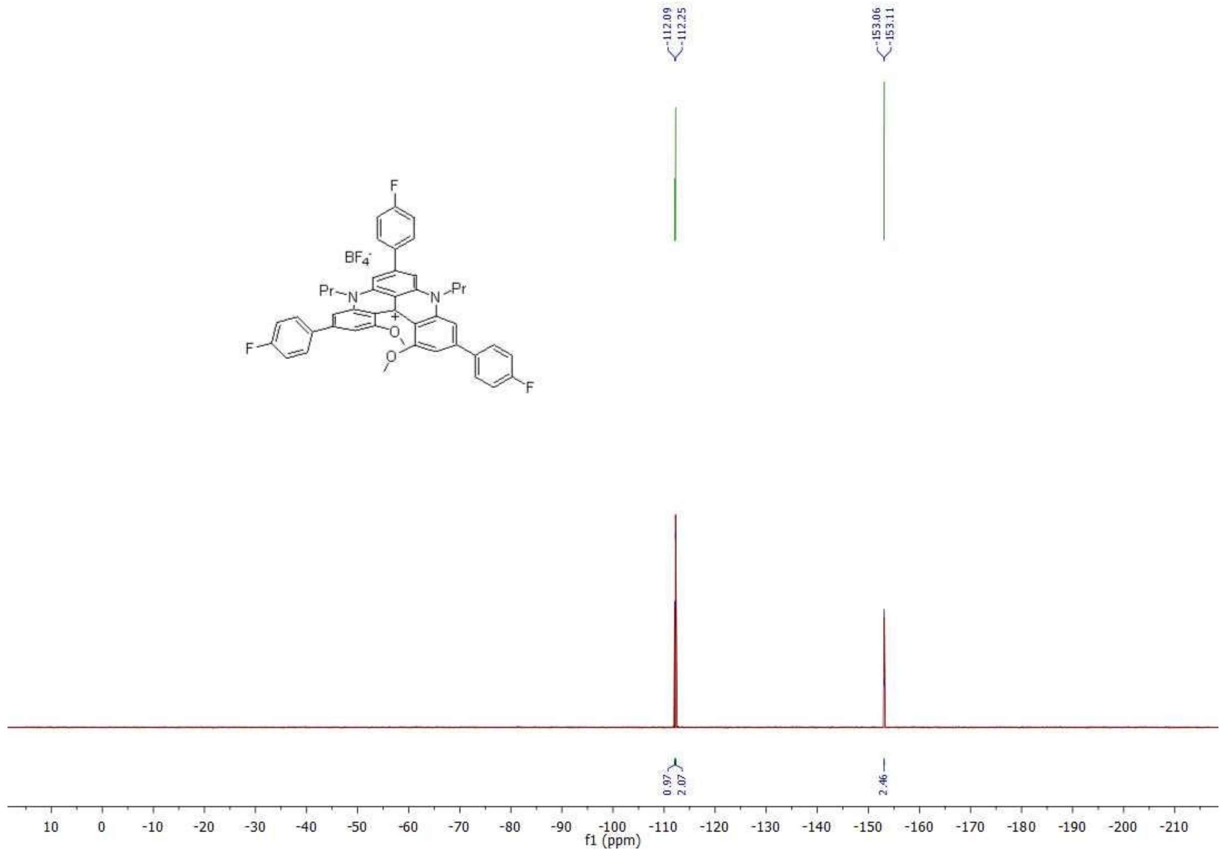

Figure S13. ^{19}F-NMR spectra of **6b** in CD_2Cl_2, 282 MHz.

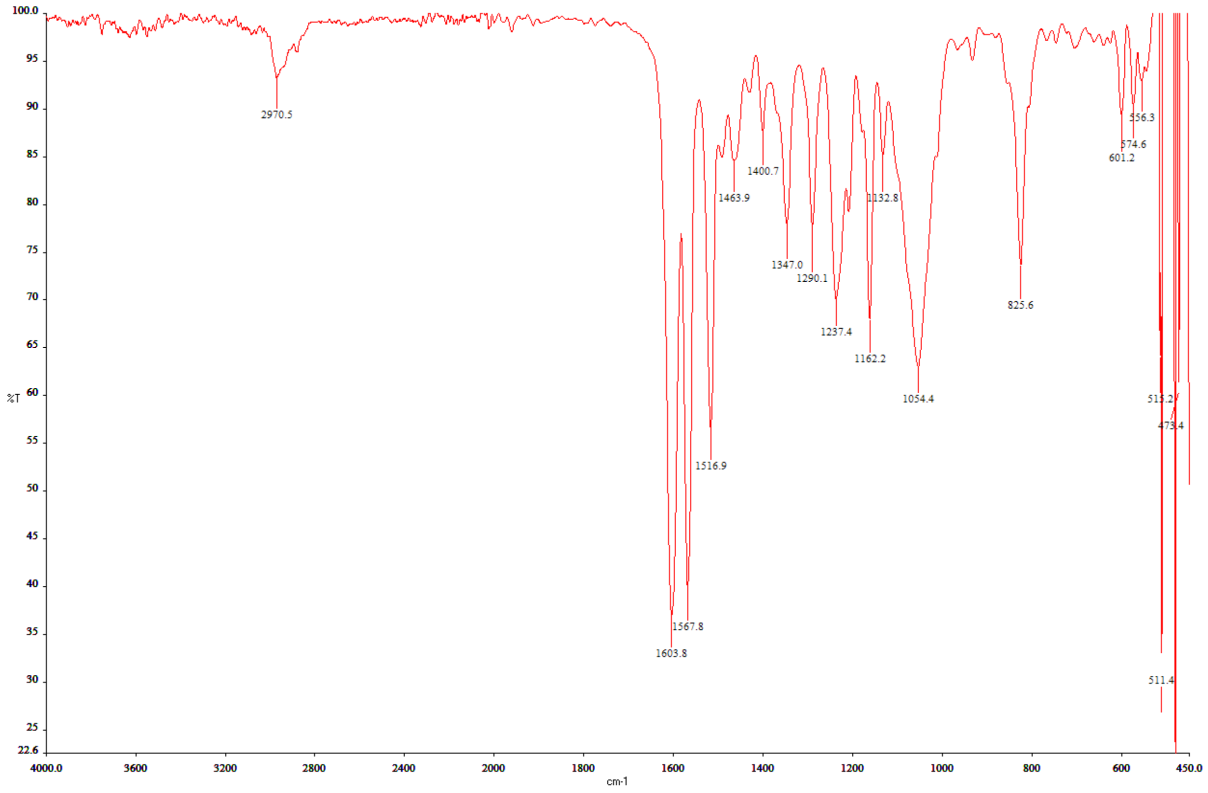

Figure S14. IR spectrum (neat) of **6b**.

Mass Spectrometry Core Facility

Lacour Group – University of Geneva

UNIVERSITÉ DE GENÈVE
FACULTÉ DES SCIENCES

ESI-HRMS – Certificate of Analysis

Applicant:	Lucas Frederic	Date of certificate:	29/07/21
Sample name:	LFC-192	Instrument:	Xevo G2 Tof (TOF)
Folder:	290721.PRO	Mobile phase:	MeOH (100 µl/min)
Analyst:	Stéphane Grass	Ionisation mode:	ESI (positive polarity)

Elemental Formula	Ion type	*Masslynx values **** *calc. m/z*	*meas. m/z*	Calc. *m/z*	Meas. *m/z*	Accuracy [a] *(ppm)*
$C_{45}H_{38}F_3N_2O_2$	$[M]^+$	*695.2885*	*695.2927*	695.2880	695.2922	6.0

[a] *Mass spectrum is calibrated by the use of the MS lockspray system (LeuEnk calibration solution).*

**** MassLynx software does not take into account the mass of the electron for ionic species, therefore the shift of m/z 0.000459.*

Zoomed mass spectrum – Isotopic distribution.

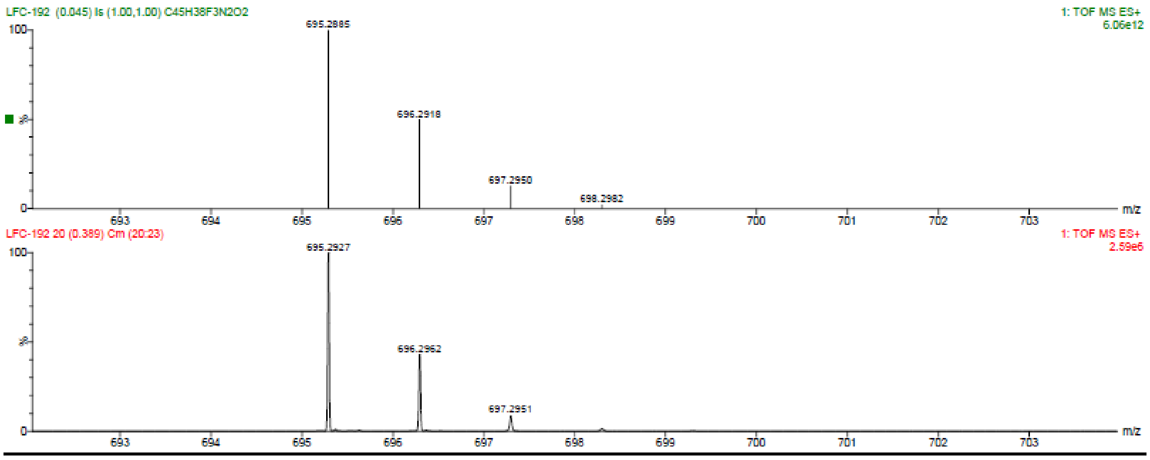

Figure S15. HRMS analysis (ESI, MeOH) report of **6b**.

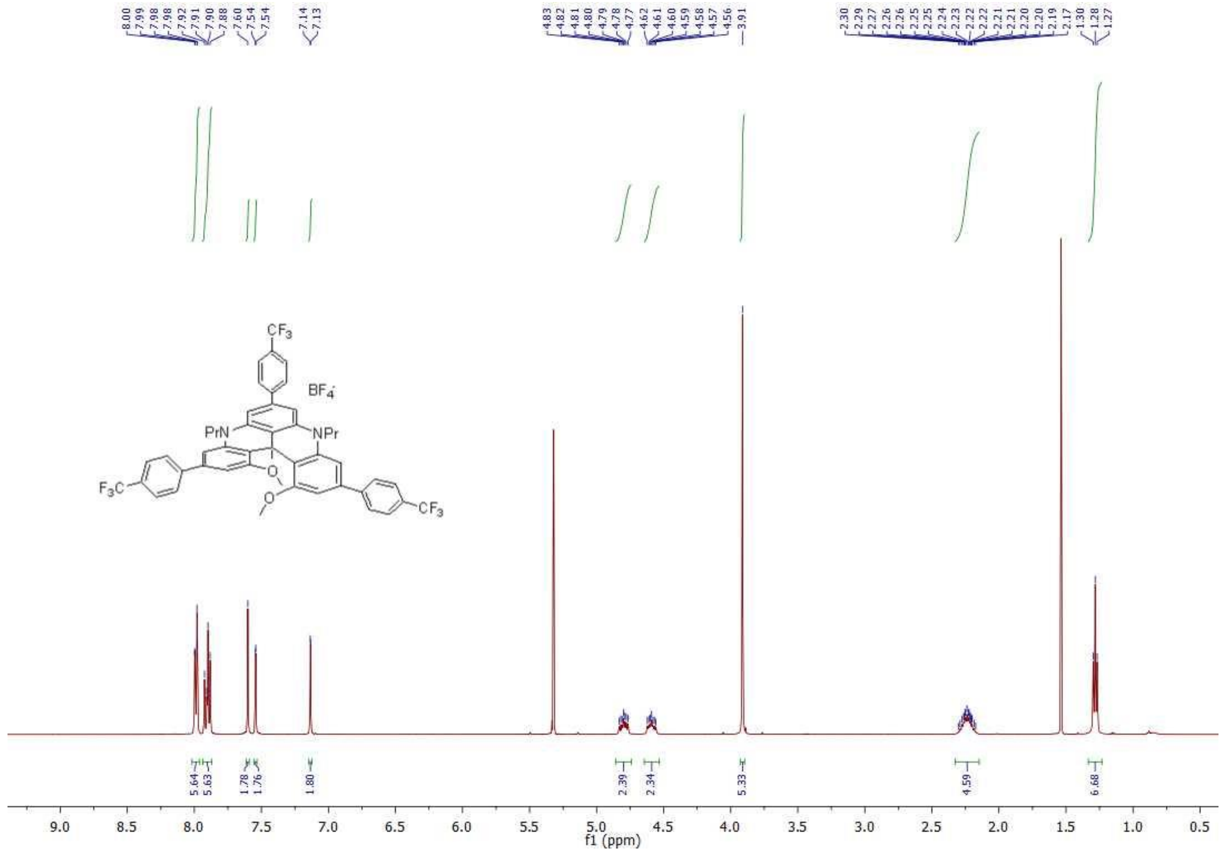

Figure S16. ^{1}H-NMR spectra of **6c** in CD_2Cl_2, 500 MHz.

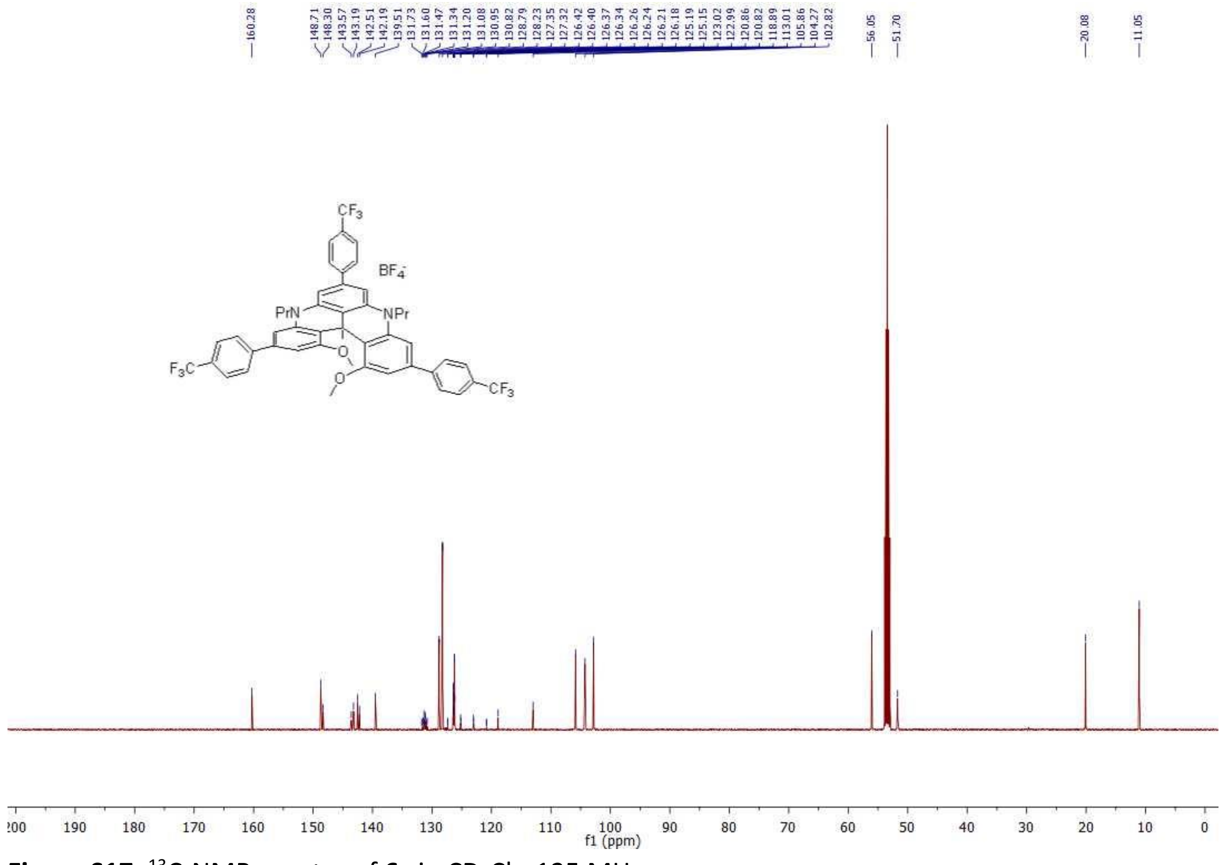

Figure S17. ^{13}C-NMR spectra of **6c** in CD_2Cl_2, 125 MHz.

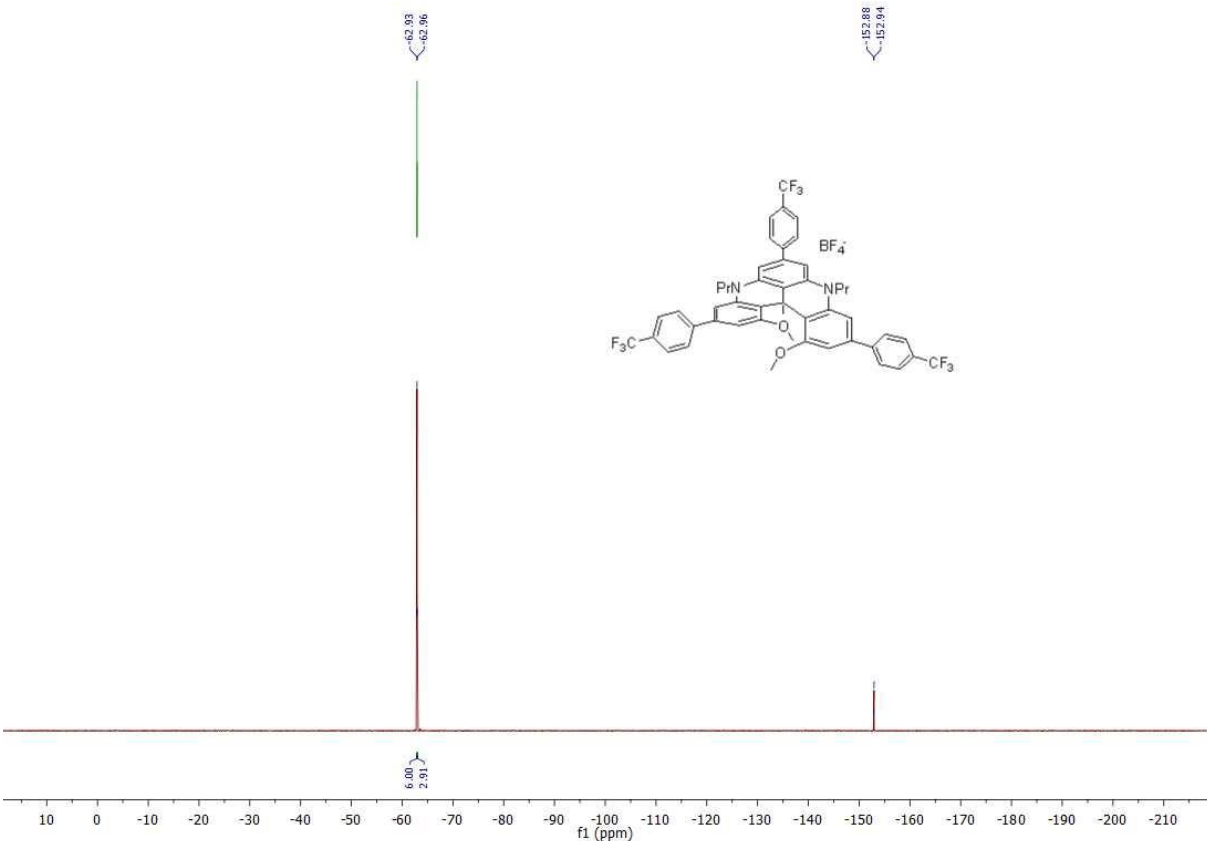

Figure S18. ^{19}F-NMR spectra of **6c** in CD_2Cl_2, 282 MHz.

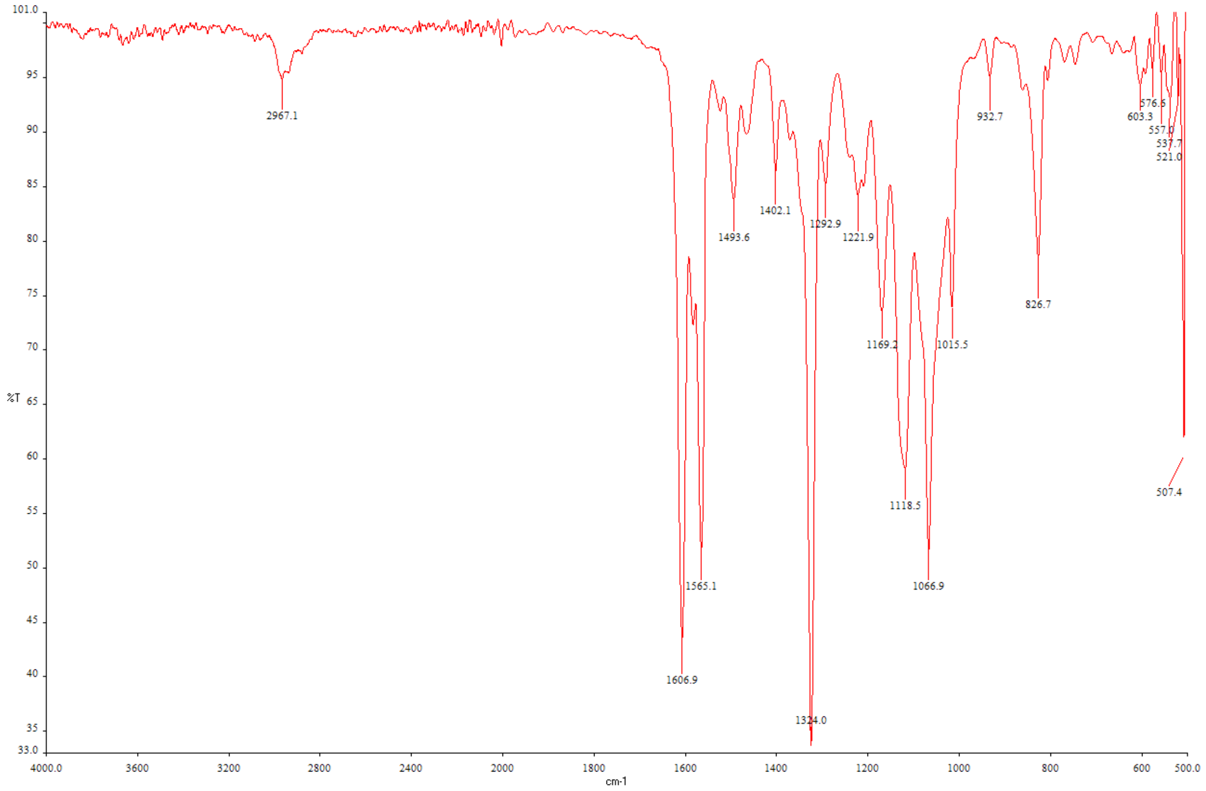

Figure S19. IR spectrum (neat) of **6c**.

Mass Spectrometry Core Facility

Lacour Group – University of Geneva

UNIVERSITÉ DE GENÈVE
FACULTÉ DES SCIENCES

ESI-HRMS – Certificate of Analysis

Applicant:	Lucas Frederic	Date of certificate:	29/07/21
Sample name:	LFC-199	Instrument:	Xevo G2 Tof (TOF)
Folder:	290721.PRO	Mobile phase:	MeOH (100 µl/min)
Analyst:	Stéphane Grass	Ionisation mode:	ESI (positive polarity)

Elemental Formula	Ion type	*Masslynx values* *** *calc. m/z*	*meas. m/z*	Calc. *m/z*	Meas. *m/z*	Accuracy [a] *(ppm)*
$C_{48}H_{38}F_9N_2O_2$	$[M]^+$	*845.2789*	*845.2792*	845.2784	845.2787	0.4

[a] *Mass spectrum is calibrated by the use of the MS lockspray system (LeuEnk calibration solution).*

**** MassLynx software does not take into account the mass of the electron for ionic species, therefore the shift of m/z 0.000459.*

<u>Zoomed mass spectrum – Isotopic distribution</u>.

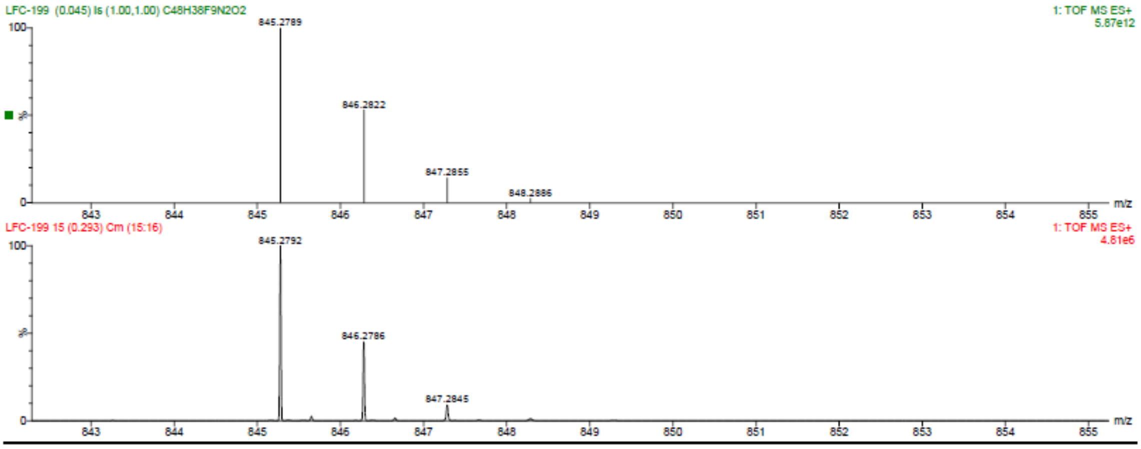

Figure S20. HRMS analysis (ESI, MeOH) report of **6c**.

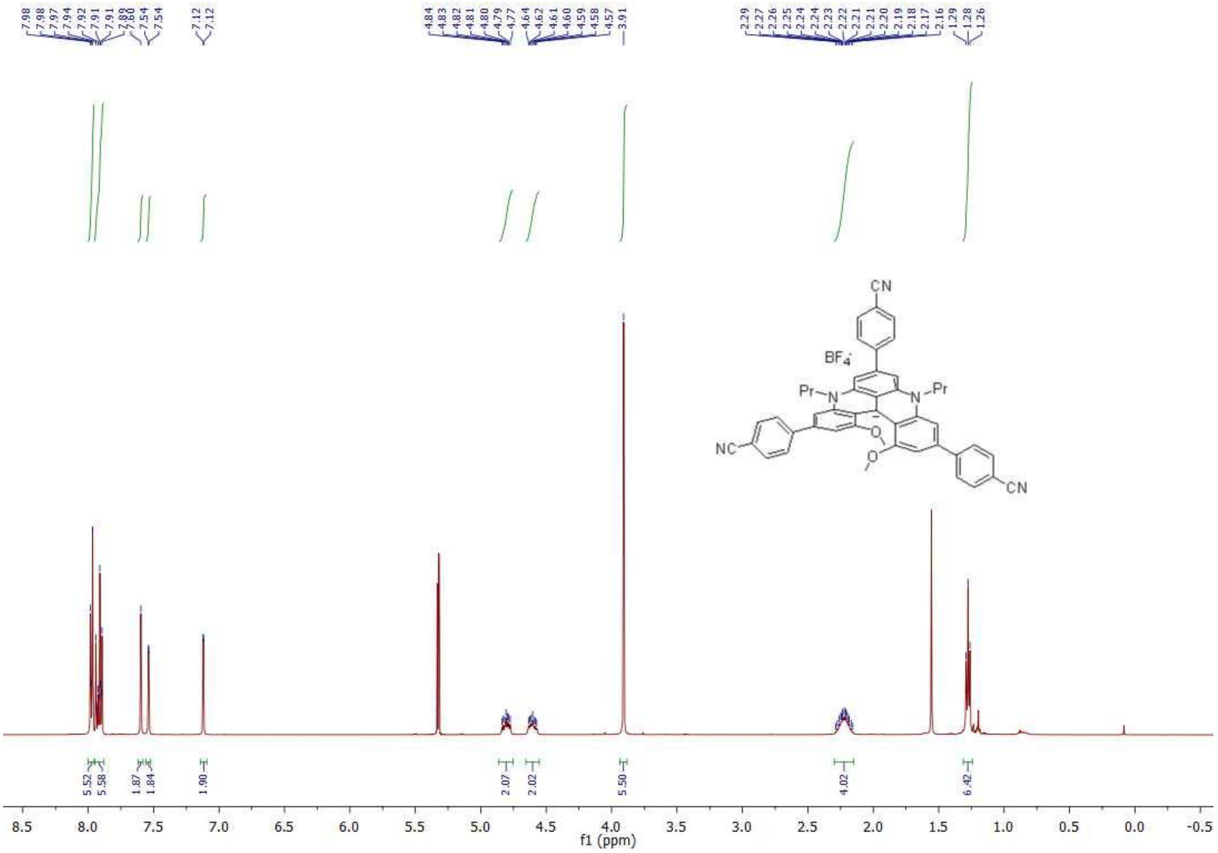

Figure S21. ^{1}H-NMR spectra of **6d** in CD_2Cl_2, 500 MHz.

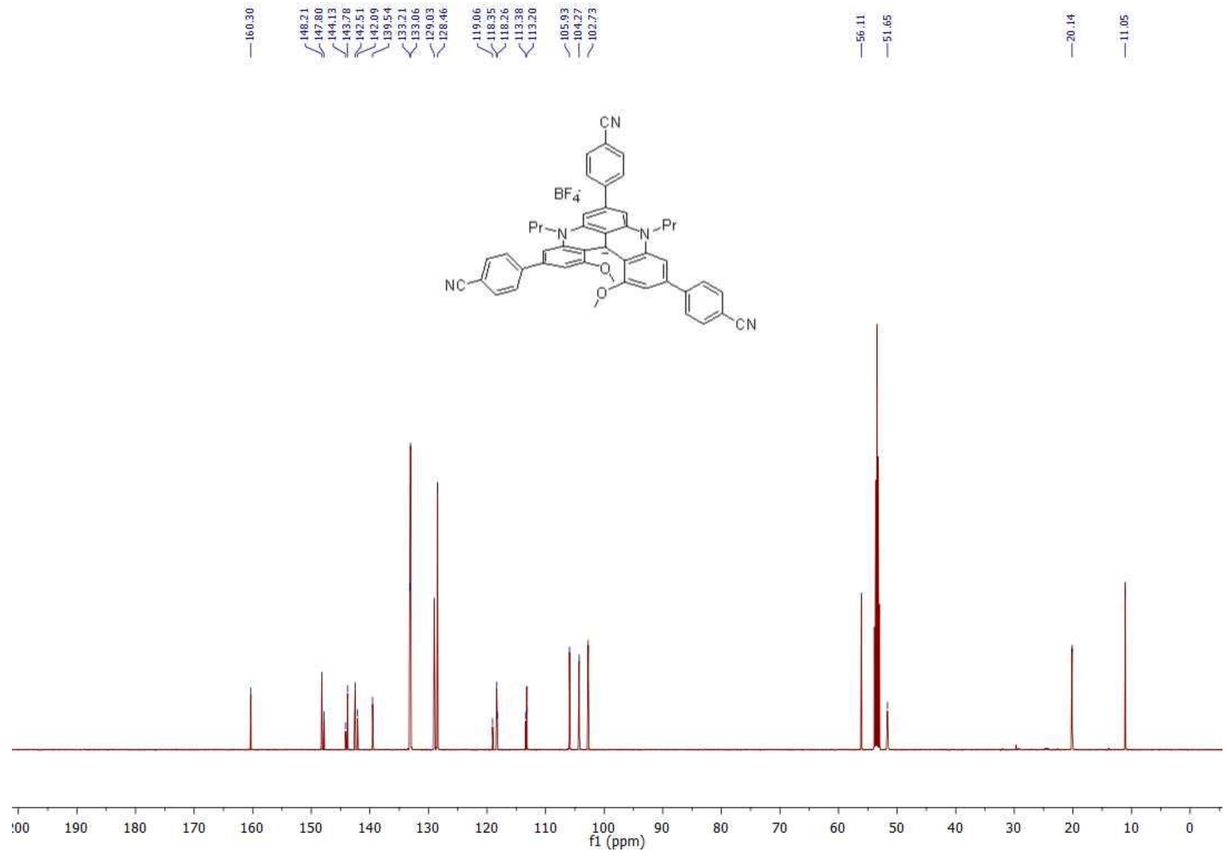

Figure S22. ^{13}C-NMR spectra of **6d** in CD_2Cl_2, 125 MHz.

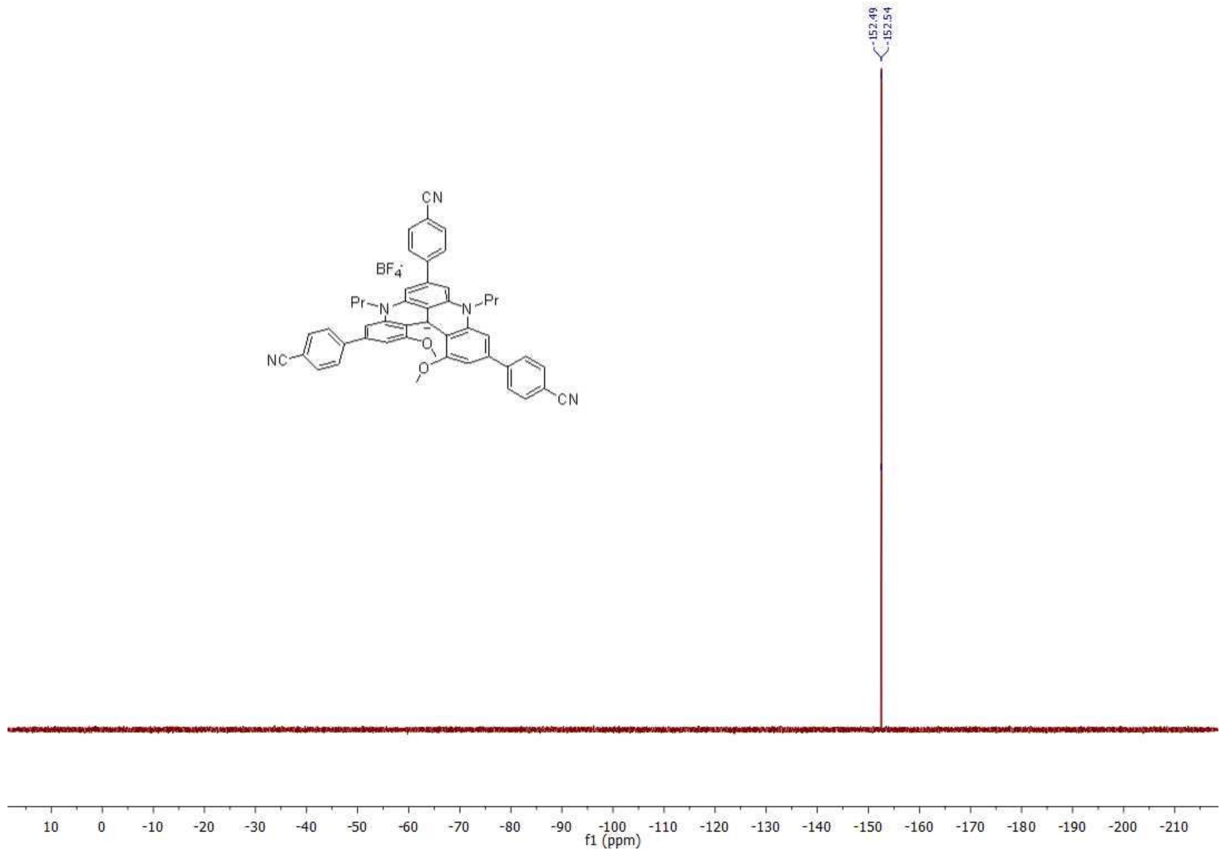

Figure S23. ^{19}F-NMR spectra of **6d** in CD_2Cl_2, 282 MHz.

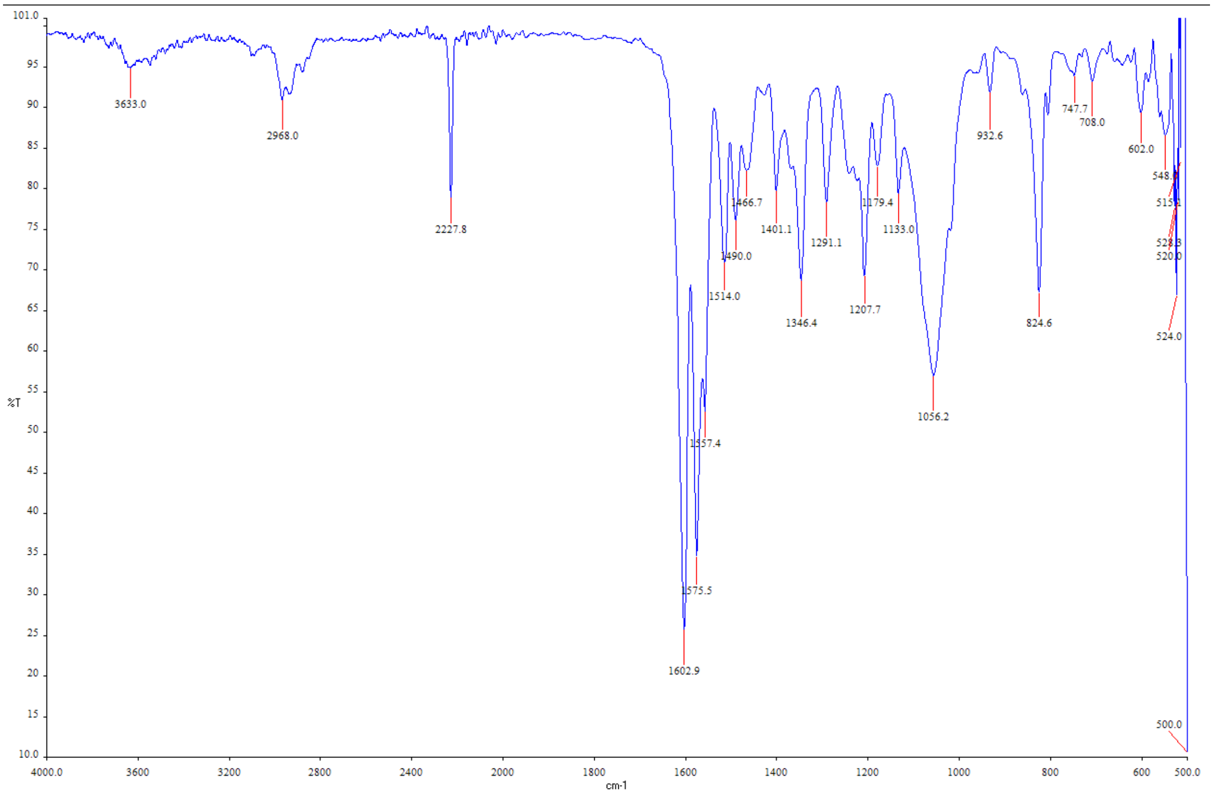

Figure S24. IR spectrum (neat) of **6d**.

Mass Spectrometry Core Facility

Lacour Group – University of Geneva

UNIVERSITÉ DE GENÈVE
FACULTÉ DES SCIENCES

ESI-HRMS – Certificate of Analysis

Applicant:	Lucas Frederic	Date of certificate:	29/07/21
Sample name:	LFC-201	Instrument:	Xevo G2 Tof (TOF)
Folder:	290721.PRO	Mobile phase:	MeOH (100 µl/min)
Analyst:	Stéphane Grass	Ionisation mode:	ESI (positive polarity)

Elemental Formula	Ion type	*Masslynx values **** *calc. m/z*	*meas. m/z*	Calc. *m/z*	Meas. *m/z*	Accuracy [a] *(ppm)*
$C_{48}H_{38}N_5O_2$	$[M]^+$	*716.3026*	*716.3064*	716.3021	716.3059	5.3

[a] *Mass spectrum is calibrated by the use of the MS lockspray system (LeuEnk calibration solution).*

**** MassLynx software does not take into account the mass of the electron for ionic species, therefore the shift of m/z 0.000459.*

<u>Zoomed mass spectrum – Isotopic distribution</u>.

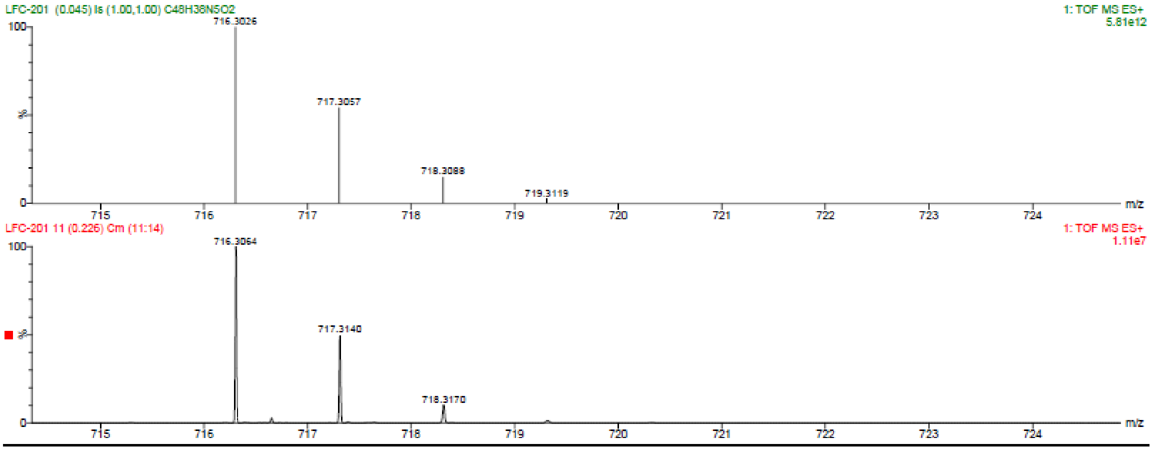

Figure S25. HRMS analysis (ESI, MeOH) report of **6d**.

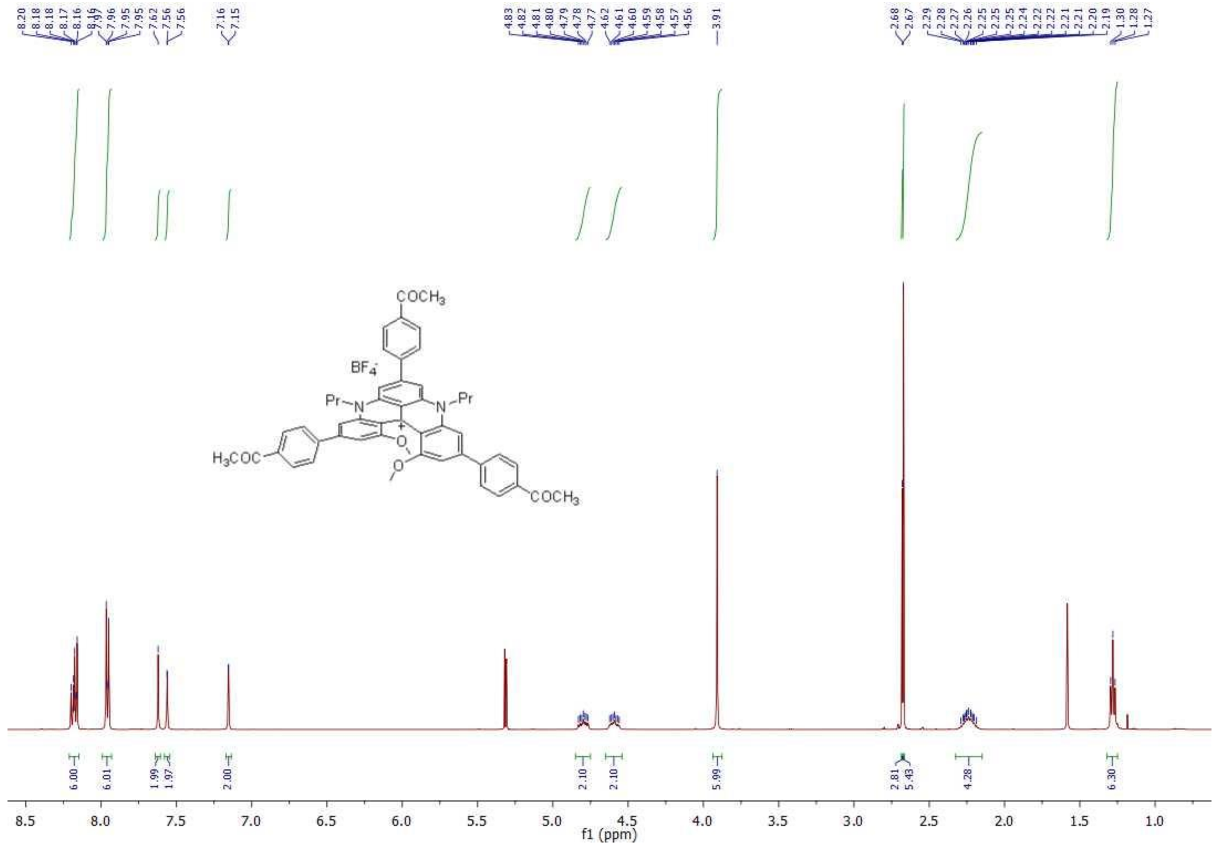

Figure S26. ^{1}H-NMR spectra of **6e** in CD_2Cl_2, 500 MHz.

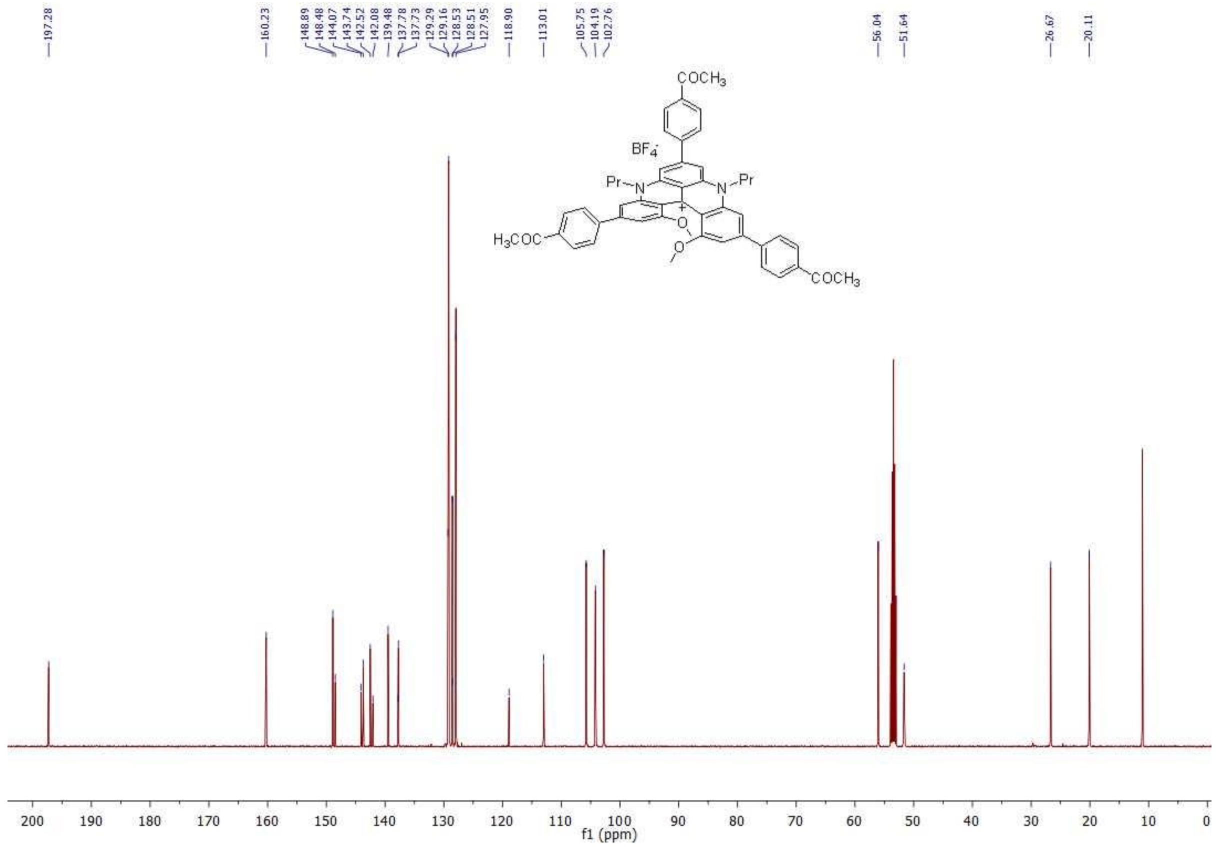

Figure S27. ^{13}C-NMR spectra of **6e** in CD_2Cl_2, 125 MHz.

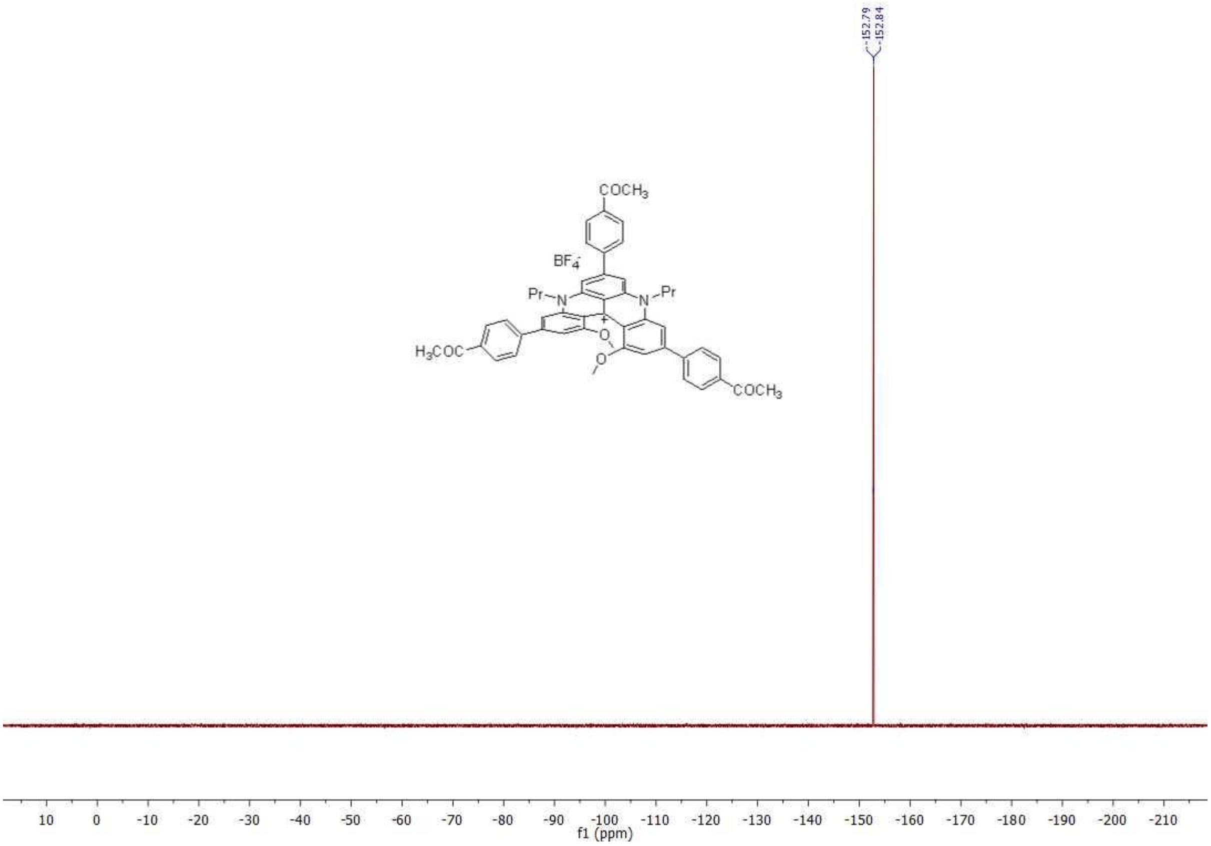

Figure S28. ^{19}F-NMR spectra of **6e** in CD_2Cl_2, 282 MHz.

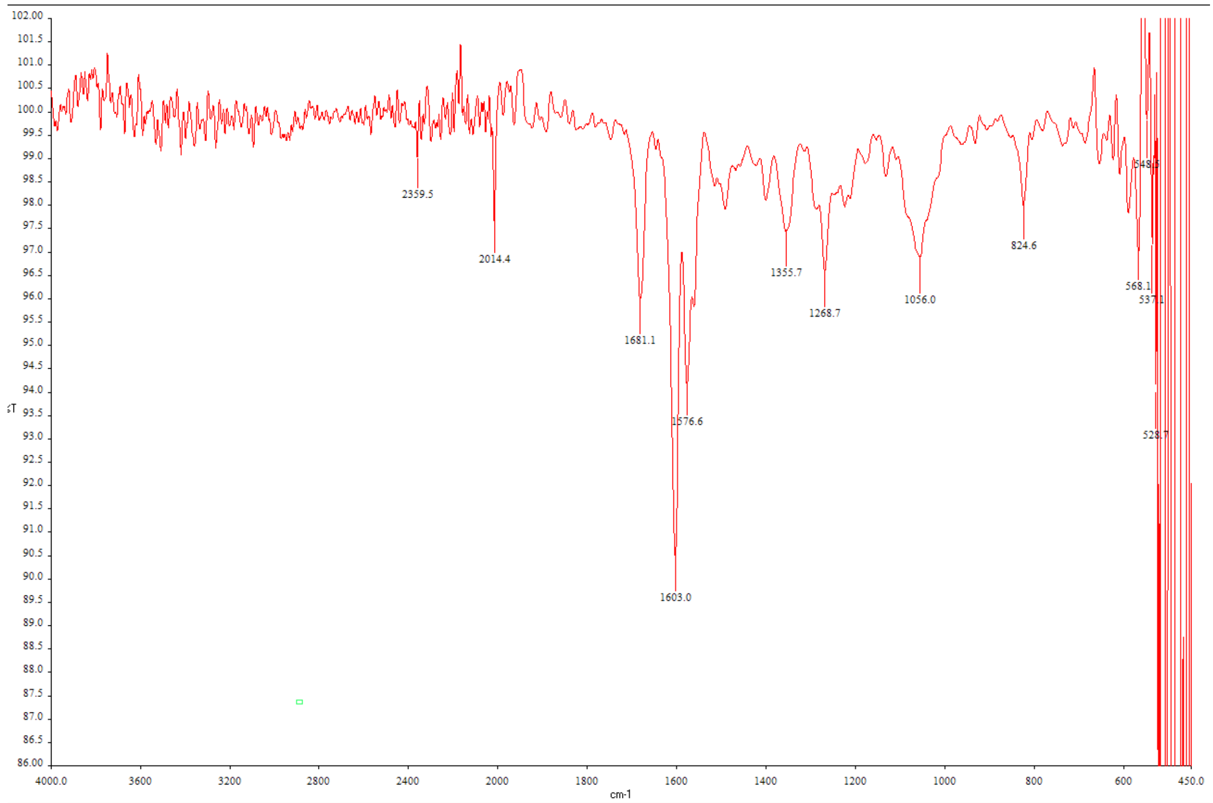

Figure S29. IR spectrum (neat) of **6e**.

Mass Spectrometry Core Facility

Lacour Group – University of Geneva

UNIVERSITÉ DE GENÈVE
FACULTÉ DES SCIENCES

ESI-HRMS – Certificate of Analysis

Applicant:	Lucas Frederic	Date of certificate:	06/09/21
Sample name:	LFC-209	Instrument:	Xevo G2 Tof (TOF)
Folder:	060921.PRO	Mobile phase:	MeOH (100 µl/min)
Analyst:	Stéphane Grass	Ionisation mode:	ESI (positive polarity)

Elemental Formula	Ion type	Masslynx values *** calc. *m/z*	Masslynx values *** meas. *m/z*	Calc. *m/z*	Meas. *m/z*	Accuracy [a] (ppm)
$C_{51}H_{47}N_2O_5$	$[M]^+$	767.3485	767.3481	767.3480	767.3476	-0.5

[a] *Mass spectrum is calibrated by the use of the MS lockspray system (LeuEnk calibration solution).*

**** MassLynx software does not take into account the mass of the electron for ionic species, therefore the shift of m/z 0.000459.*

Zoomed mass spectrum – Isotopic distribution.

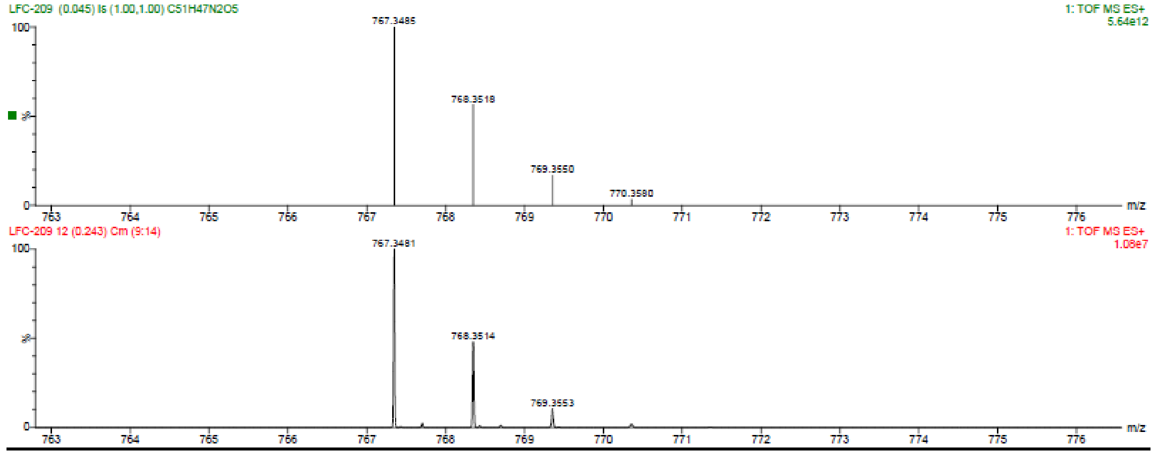

Figure S30. HRMS analysis (ESI, MeOH) report of **6e**.

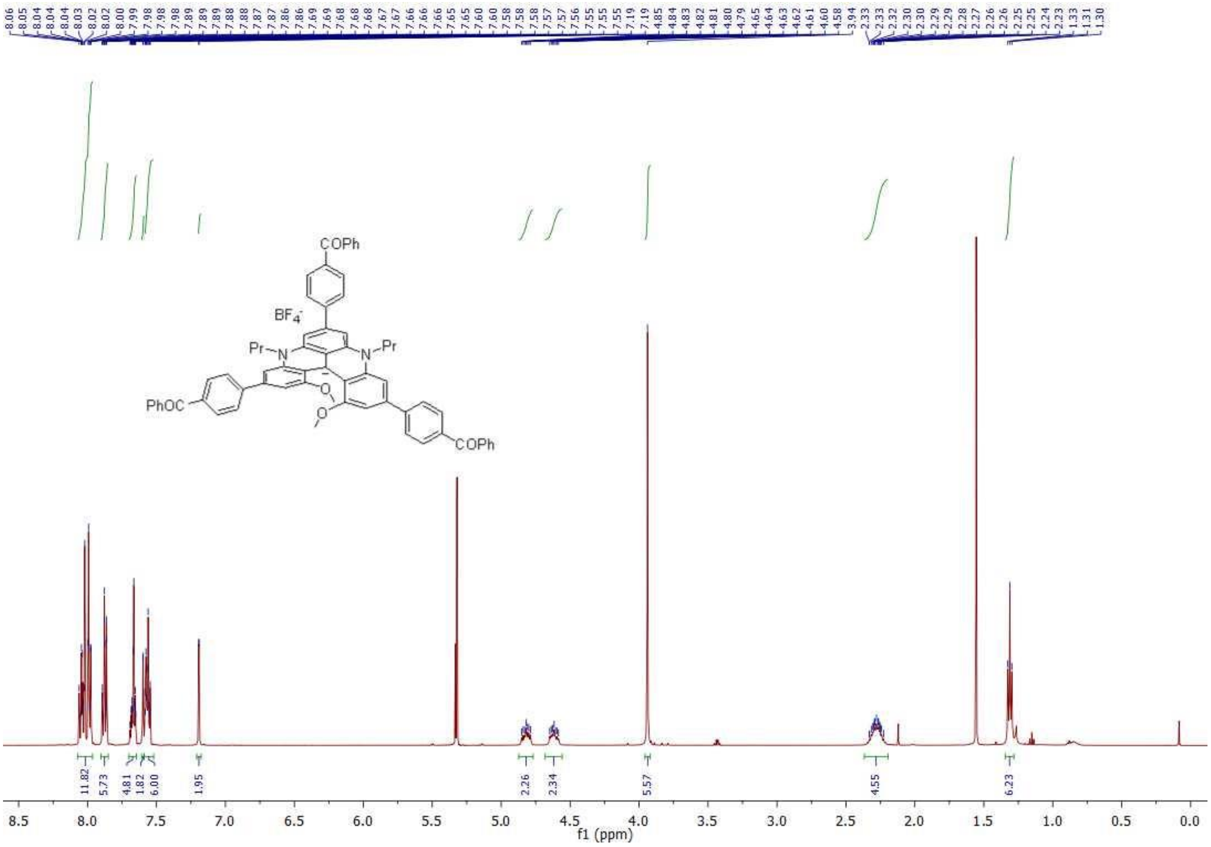

Figure S31. ^{1}H-NMR spectra of **6f** in CD_2Cl_2, 500 MHz.

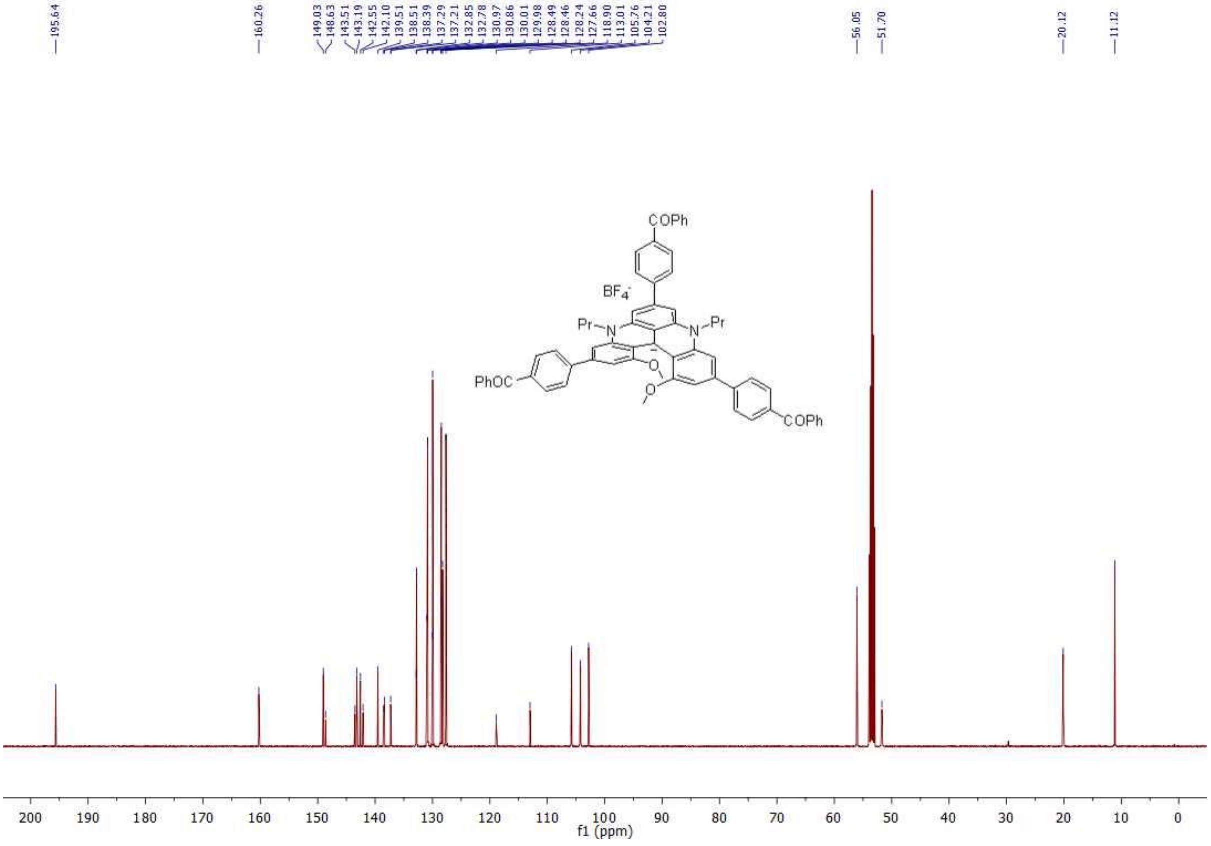

Figure S32. ^{13}C-NMR spectra of **6f** in CD_2Cl_2, 125 MHz.

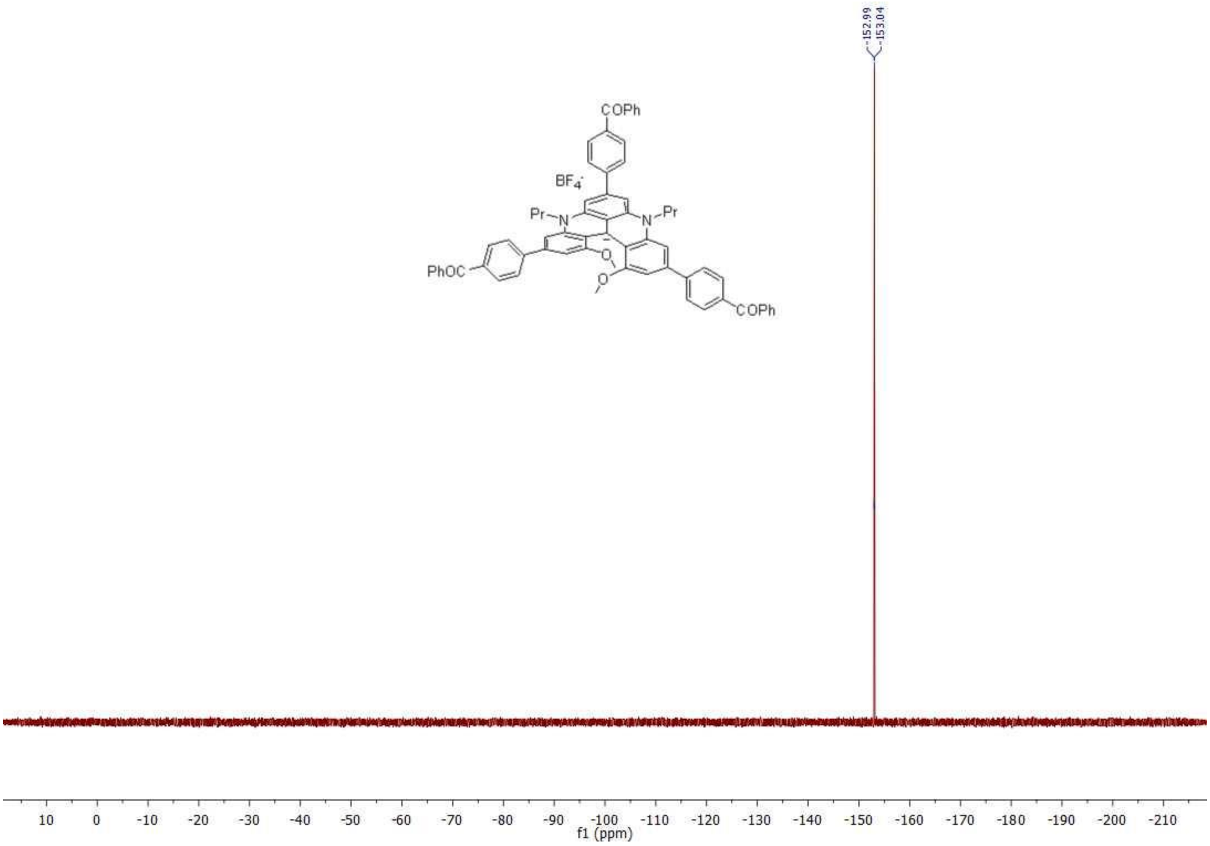

Figure S33. ^{19}F-NMR spectra of **6f** in CD_2Cl_2, 282 MHz.

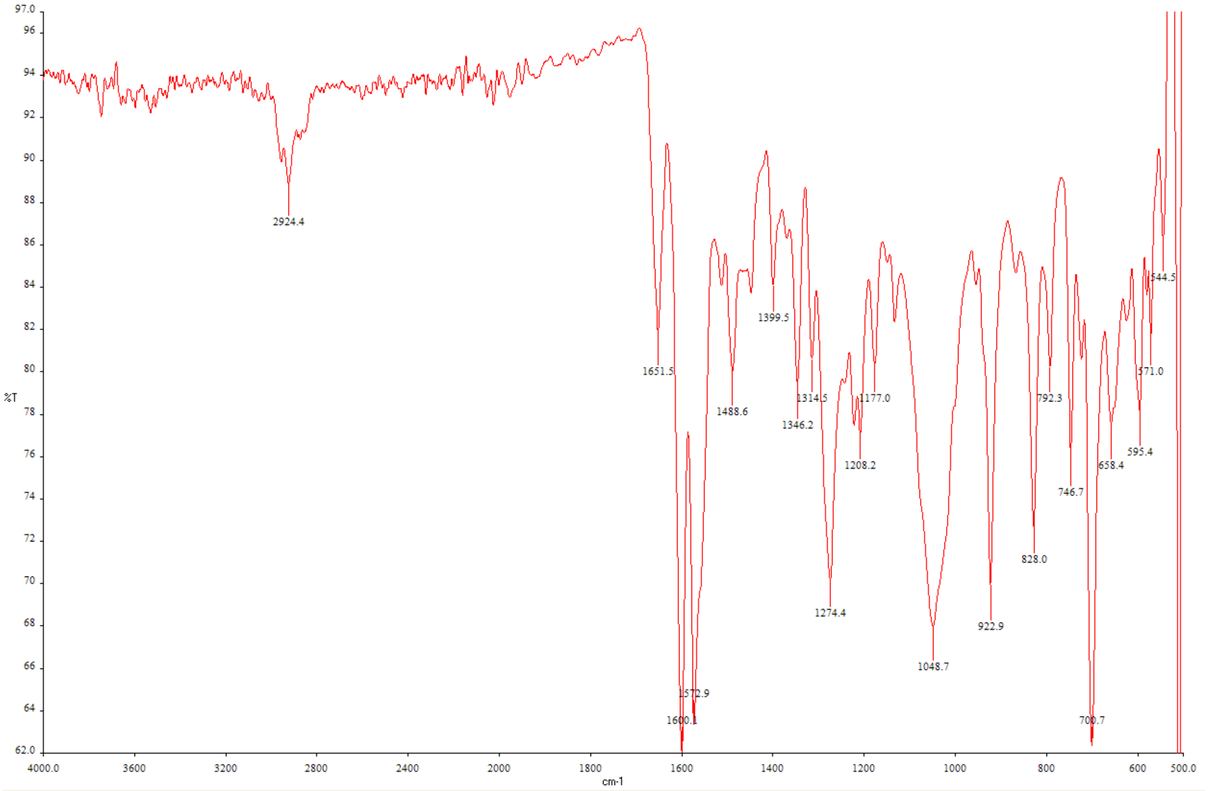

Figure S34. IR spectrum (neat) of **6f**.

Mass Spectrometry Core Facility

Lacour Group – University of Geneva

UNIVERSITÉ DE GENÈVE
FACULTÉ DES SCIENCES

ESI-HRMS – Certificate of Analysis

Applicant:	Lucas Frederic	Date of certificate:	06/09/21
Sample name:	LFC-212	Instrument:	Xevo G2 Tof (TOF)
Folder:	060921.PRO	Mobile phase:	MeOH (100 µl/min)
Analyst:	Stéphane Grass	Ionisation mode:	ESI (positive polarity)

Elemental Formula	Ion type	Masslynx values *** calc. m/z	Masslynx values *** meas. m/z	Calc. *m/z*	Meas. *m/z*	Accuracy [a] (ppm)
$C_{66}H_{53}N_2O_5$	$[M]^+$	953.3954	953.3956	953.3949	953.3951	0.2

[a] *Mass spectrum is calibrated by the use of the MS lockspray system (LeuEnk calibration solution).*

**** MassLynx software does not take into account the mass of the electron for ionic species, therefore the shift of m/z 0.000459.*

Zoomed mass spectrum – Isotopic distribution.

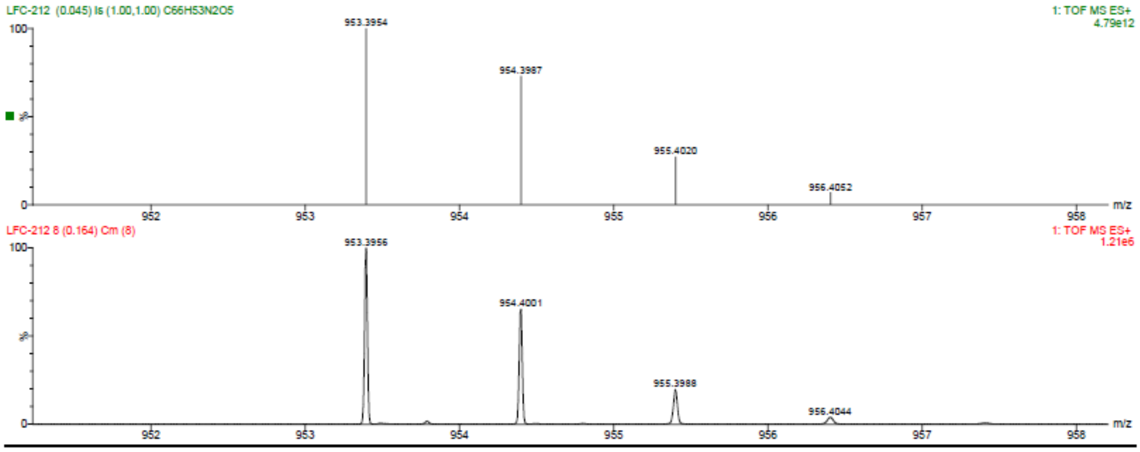

Figure S35. HRMS analysis (ESI, MeOH) report of **6f**.

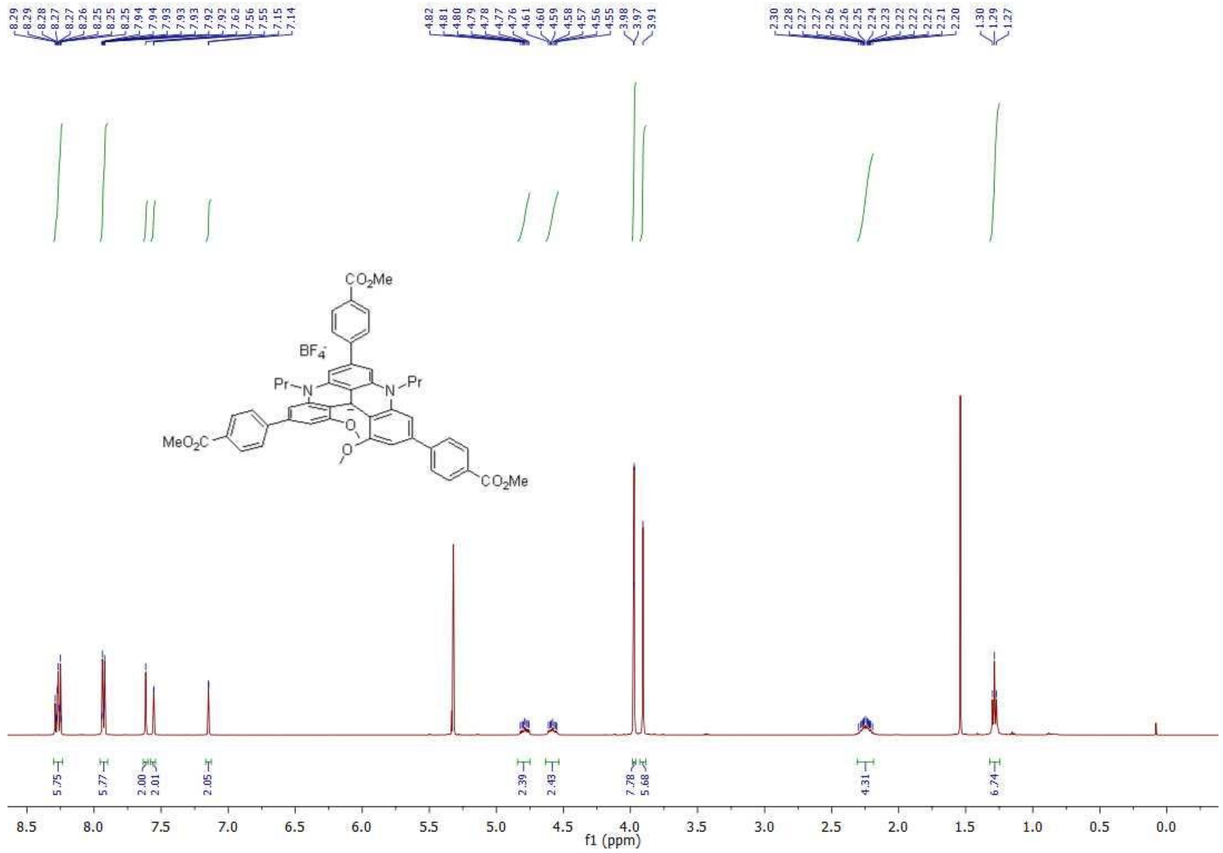

Figure S36. ^{1}H-NMR spectra of **6g** in CD_2Cl_2, 500 MHz.

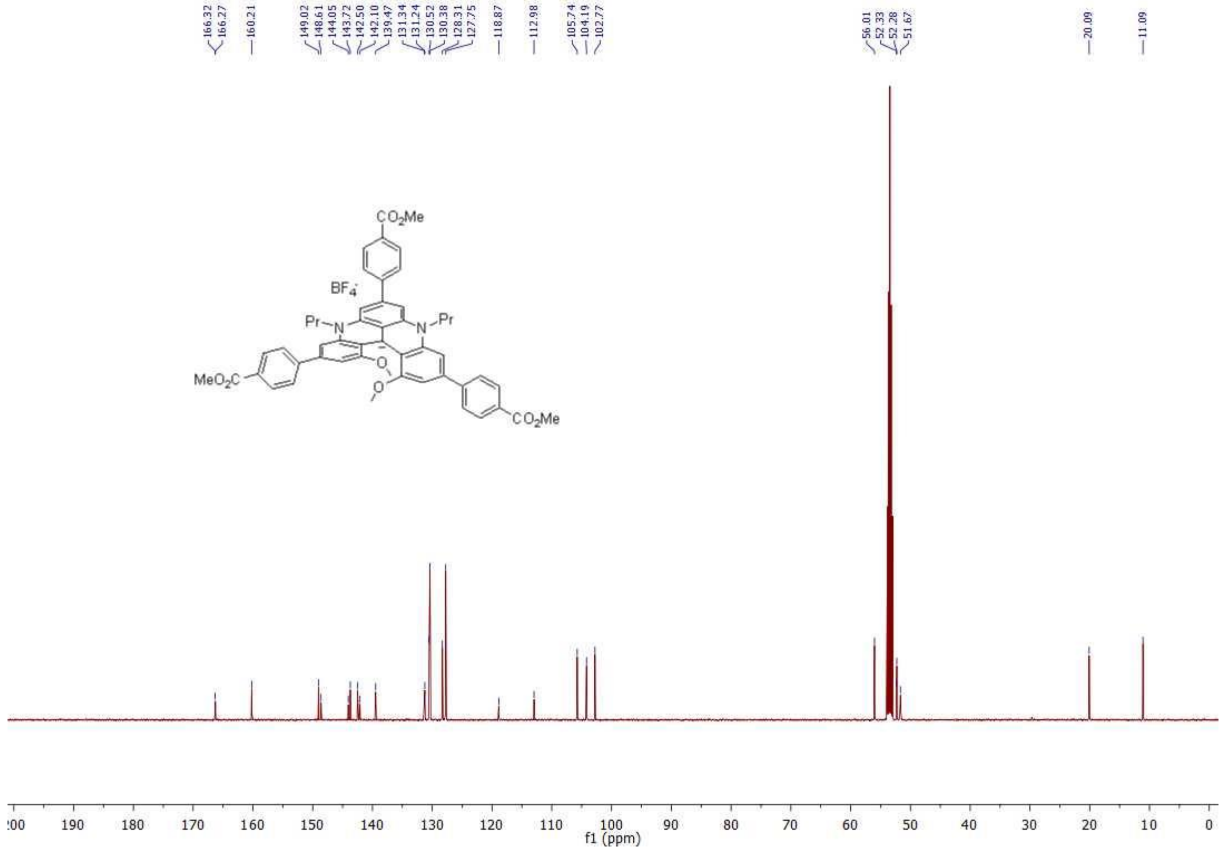

Figure S37. ^{13}C-NMR spectra of **6g** in CD_2Cl_2, 125 MHz.

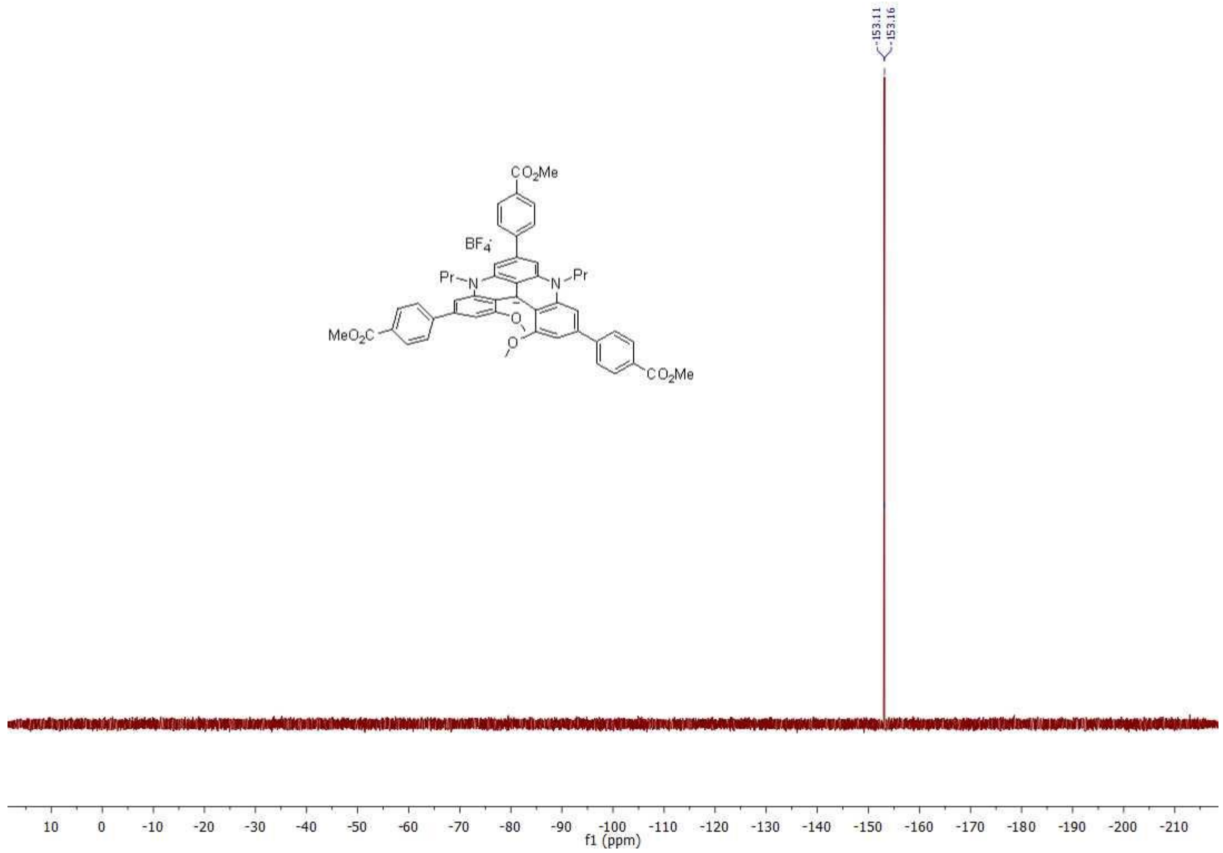

Figure S38. ^{19}F-NMR spectra of **6g** in CD_2Cl_2, 282 MHz.

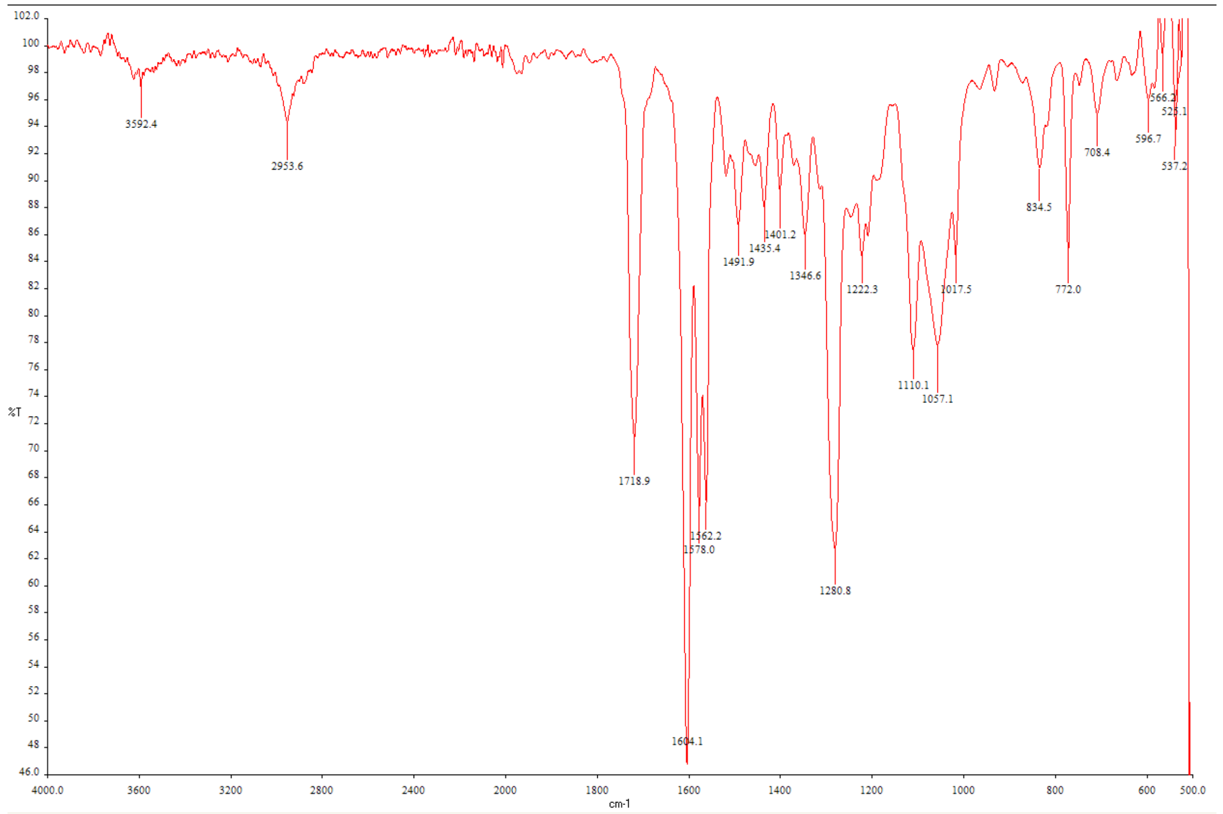

Figure S39. IR spectrum (neat) of **6g**.

Mass Spectrometry Core Facility

Lacour Group – University of Geneva

UNIVERSITÉ DE GENÈVE
FACULTÉ DES SCIENCES

ESI-HRMS – Certificate of Analysis

Applicant:	Lucas Frederic	Date of certificate:	29/07/21
Sample name:	LFC-206	Instrument:	Xevo G2 Tof (TOF)
Folder:	290721.PRO	Mobile phase:	MeOH (100 µl/min)
Analyst:	Stéphane Grass	Ionisation mode:	ESI (positive polarity)

Elemental Formula	Ion type	Masslynx values *** calc. *m/z*	Masslynx values *** meas. *m/z*	Calc. *m/z*	Meas. *m/z*	Accuracy [a] (ppm)
$C_{51}H_{47}N_2O_8$	$[M]^+$	815.3333	815.3323	815.3328	815.3318	-1.2

[a] *Mass spectrum is calibrated by the use of the MS lockspray system (LeuEnk calibration solution).*

**** MassLynx software does not take into account the mass of the electron for ionic species, therefore the shift of m/z 0.000459.*

Zoomed mass spectrum – Isotopic distribution.

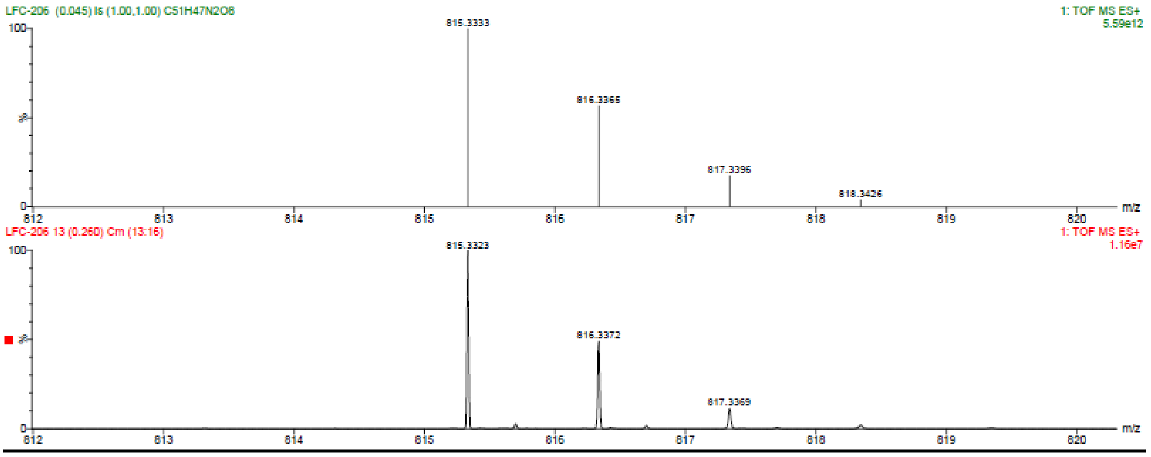

Figure S40. HRMS analysis (ESI, MeOH) report of **6g**.

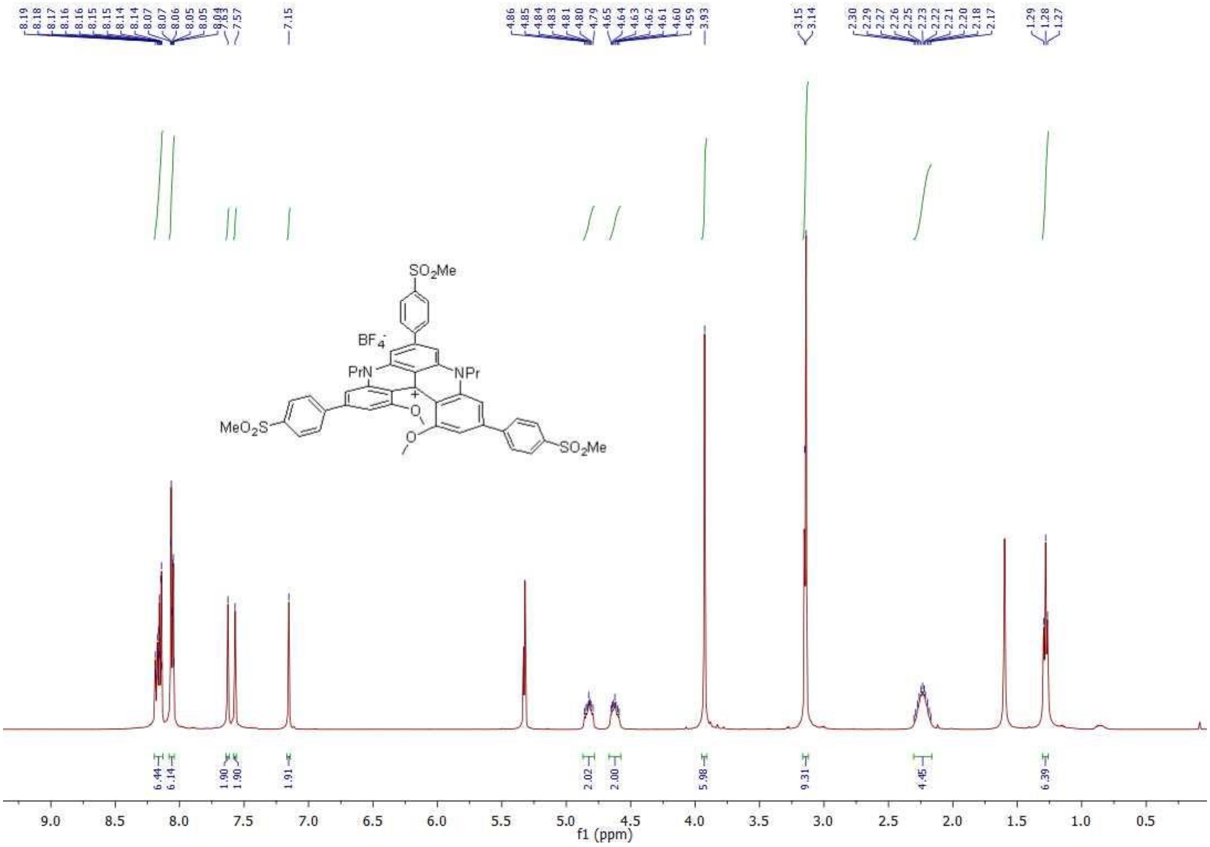

Figure S41. ^{1}H-NMR spectra of **6h** in CD_2Cl_2, 500 MHz.

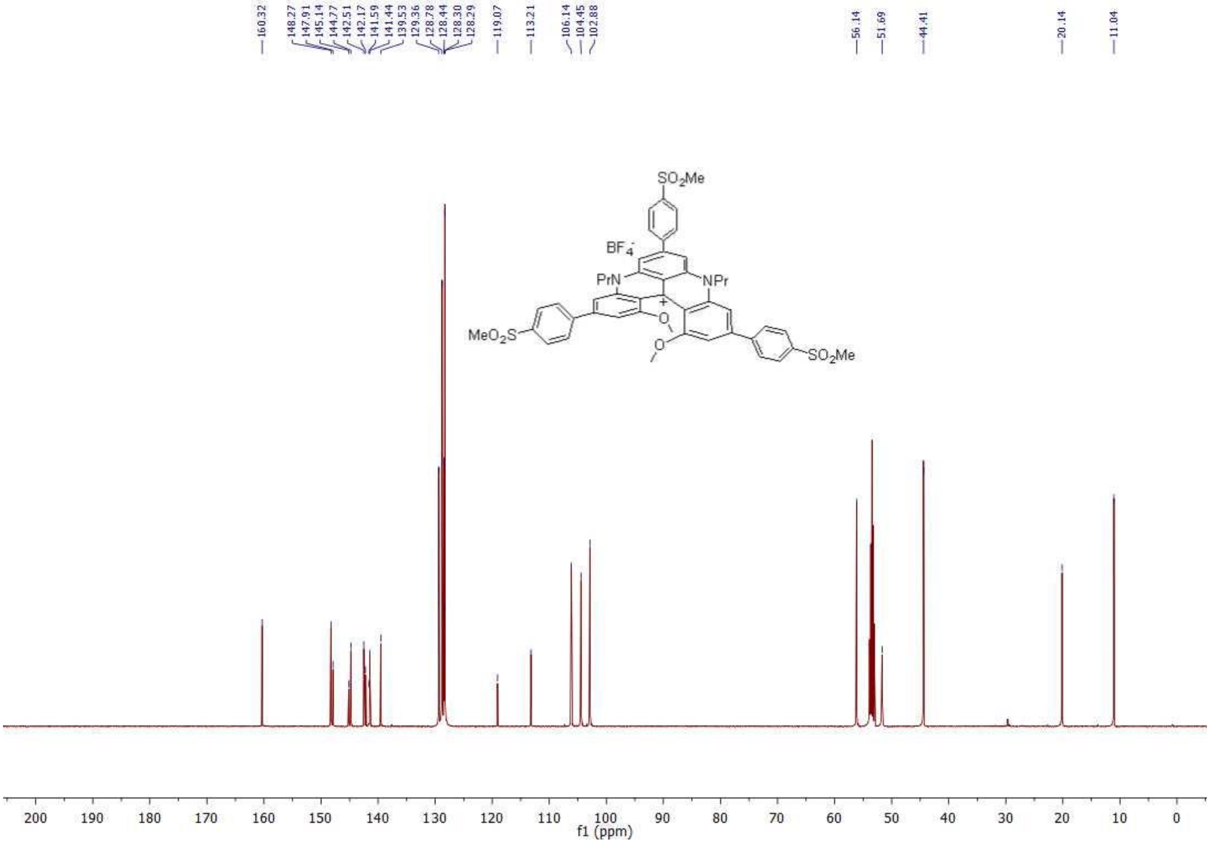

Figure S42. ^{13}C-NMR spectra of **6h** in CD_2Cl_2, 125 MHz.

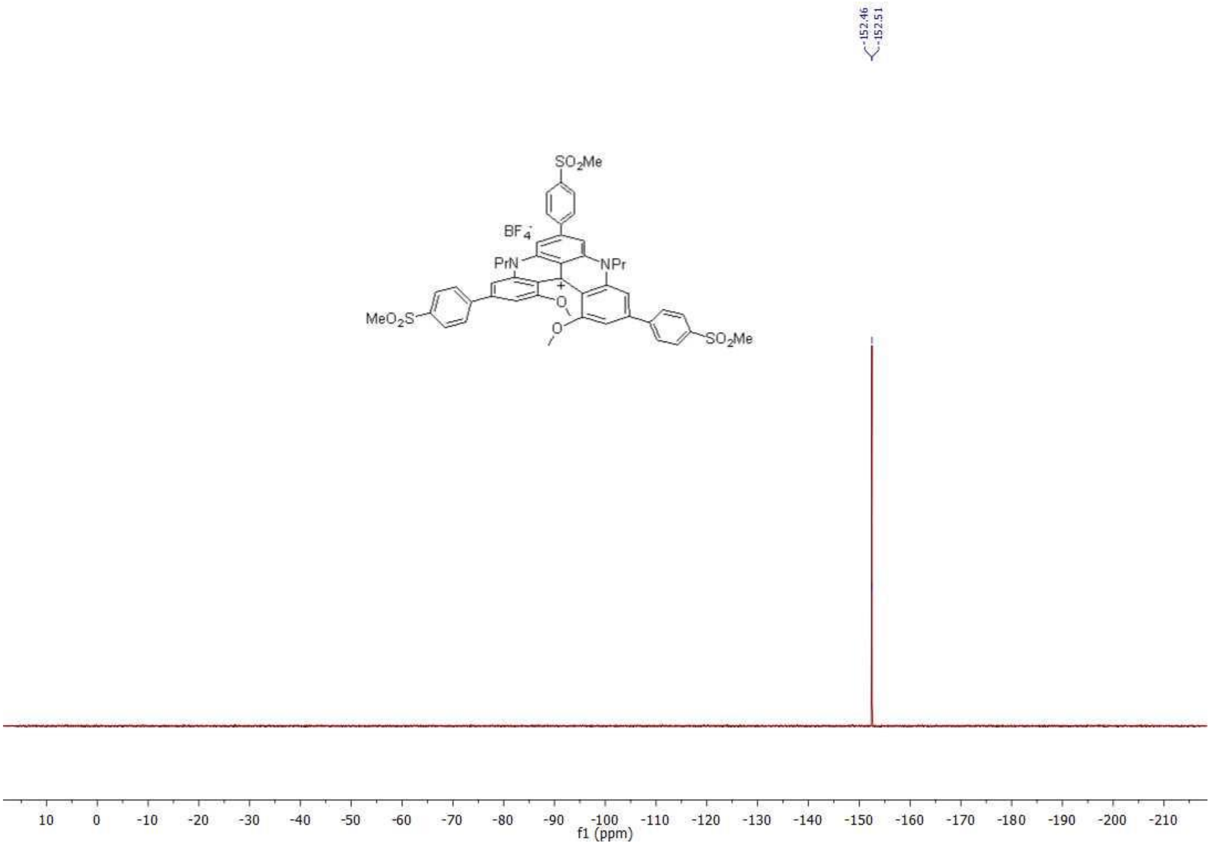

Figure S43. ^{19}F-NMR spectra of **6h** in CD_2Cl_2, 282 MHz.

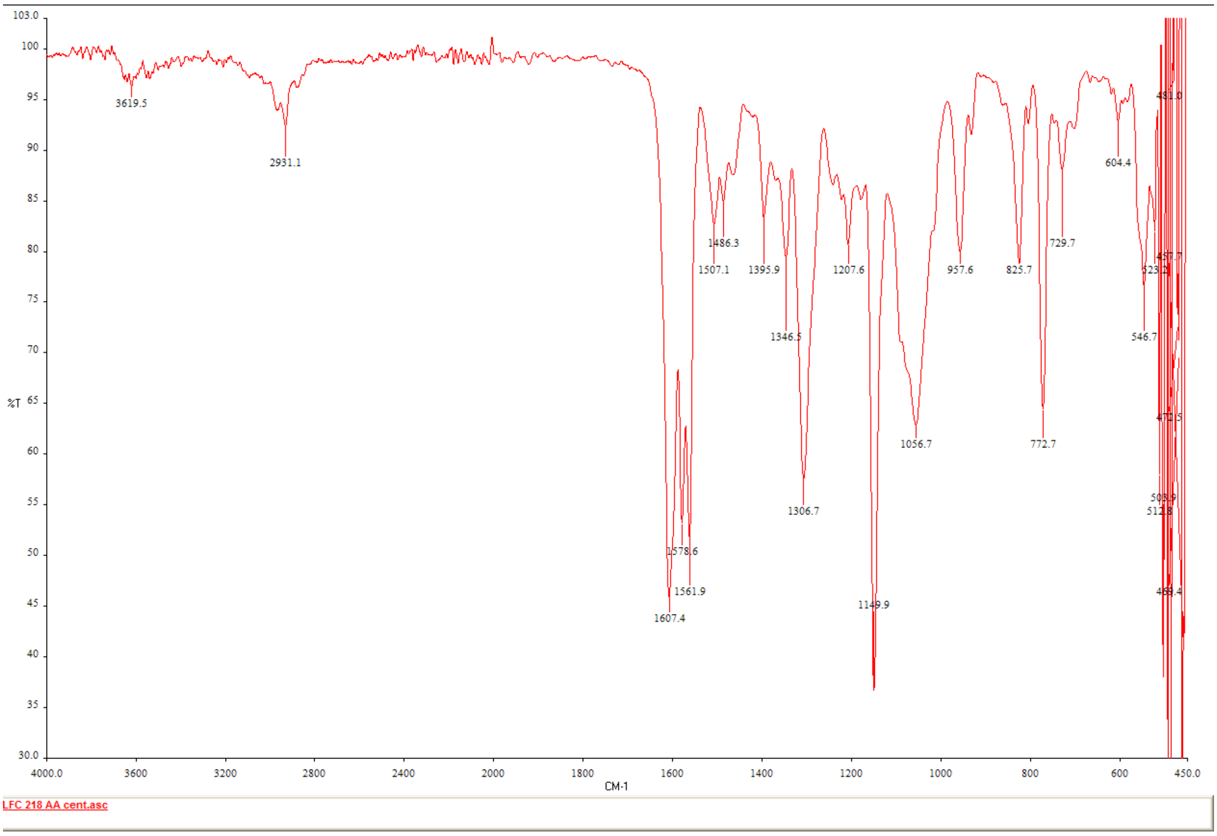

Figure S44. IR spectrum (neat) of **6h**.

Mass Spectrometry Core Facility

Lacour Group – University of Geneva

UNIVERSITÉ DE GENÈVE
FACULTÉ DES SCIENCES

ESI-HRMS – Certificate of Analysis

Applicant:	Lucas Frederic	Date of certificate:	06/09/21
Sample name:	LFC-219	Instrument:	Xevo G2 Tof (TOF)
Folder:	060921.PRO	Mobile phase:	MeOH (100 µl/min)
Analyst:	Stéphane Grass	Ionisation mode:	ESI (positive polarity)

Elemental Formula	Ion type	Masslynx values *** calc. *m/z*	Masslynx values *** meas. *m/z*	Calc. *m/z*	Meas. *m/z*	Accuracy [a] *(ppm)*
$C_{48}H_{47}N_2O_8S_3$	$[M]^+$	875.2489	875.2516	875.2484	875.2511	3.1

[a] *Mass spectrum is calibrated by the use of the MS lockspray system (LeuEnk calibration solution).*

**** MassLynx software does not take into account the mass of the electron for ionic species, therefore the shift of m/z 0.000459.*

Zoomed mass spectrum – Isotopic distribution.

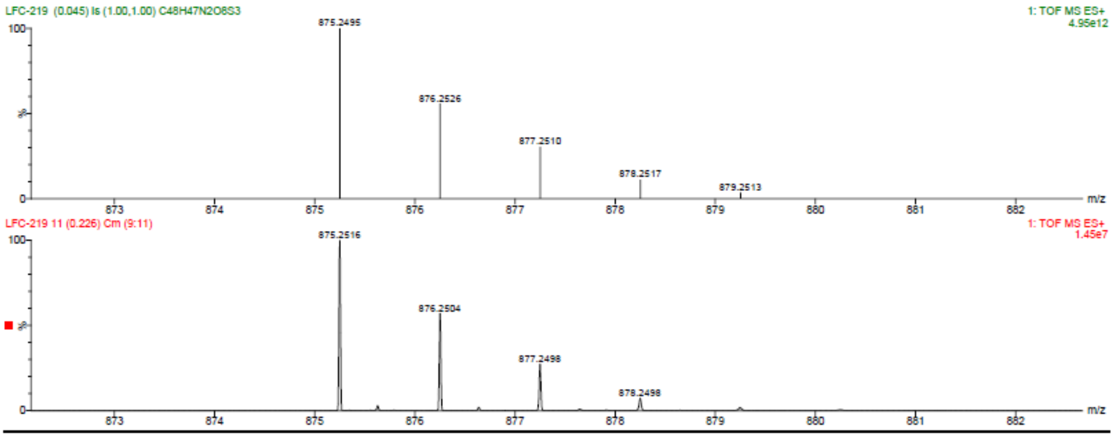

Figure S45. HRMS analysis (ESI, MeOH) report of **6h**.

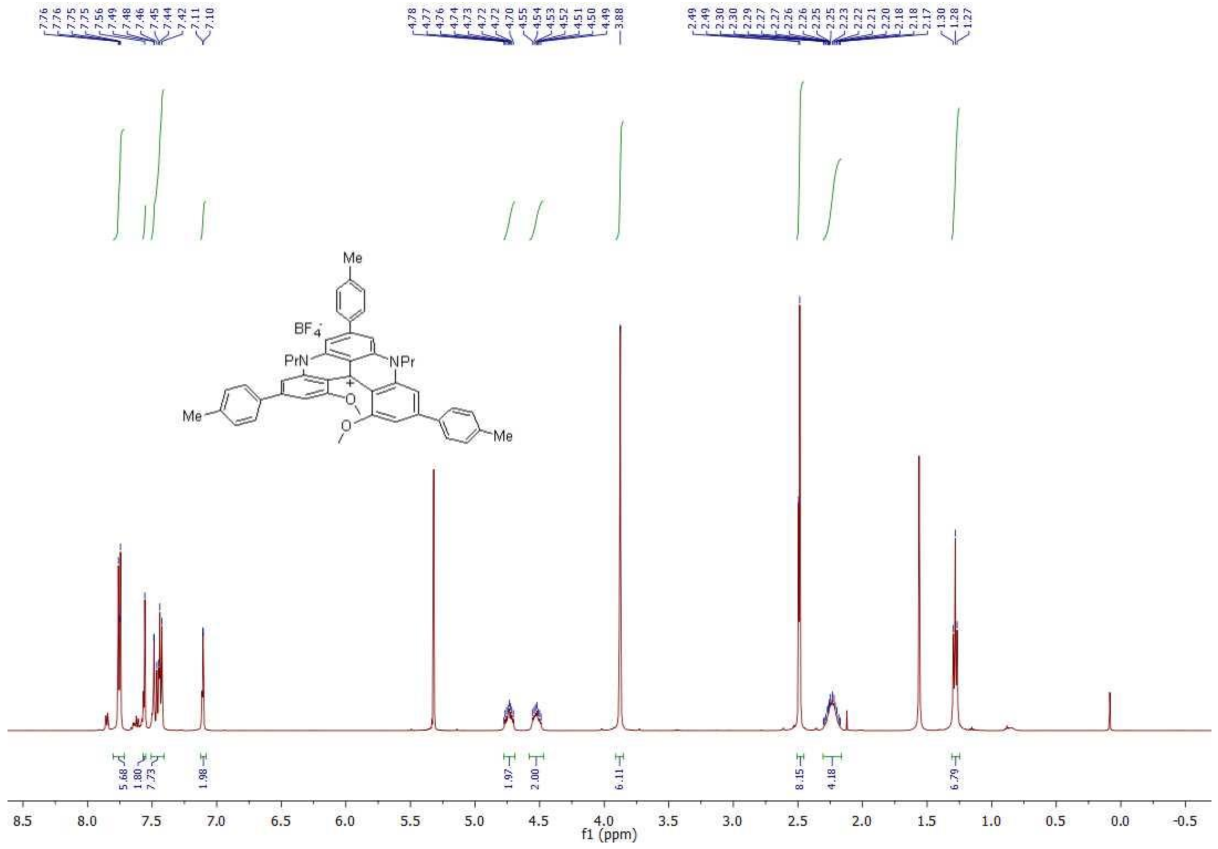

Figure S46. ^{1}H-NMR spectra of **6i** in CD_2Cl_2, 500 MHz.

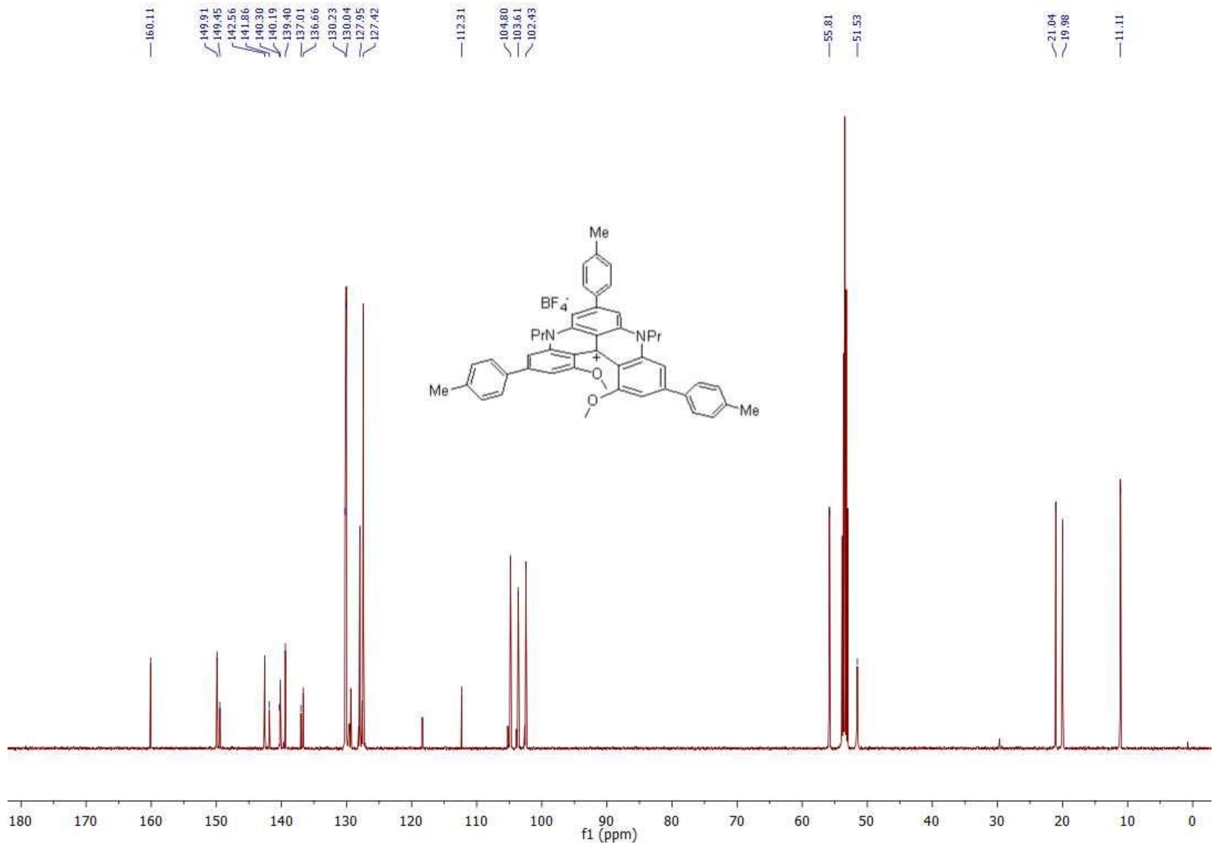

Figure S47. ^{13}C-NMR spectra of **6i** in CD_2Cl_2, 125 MHz.

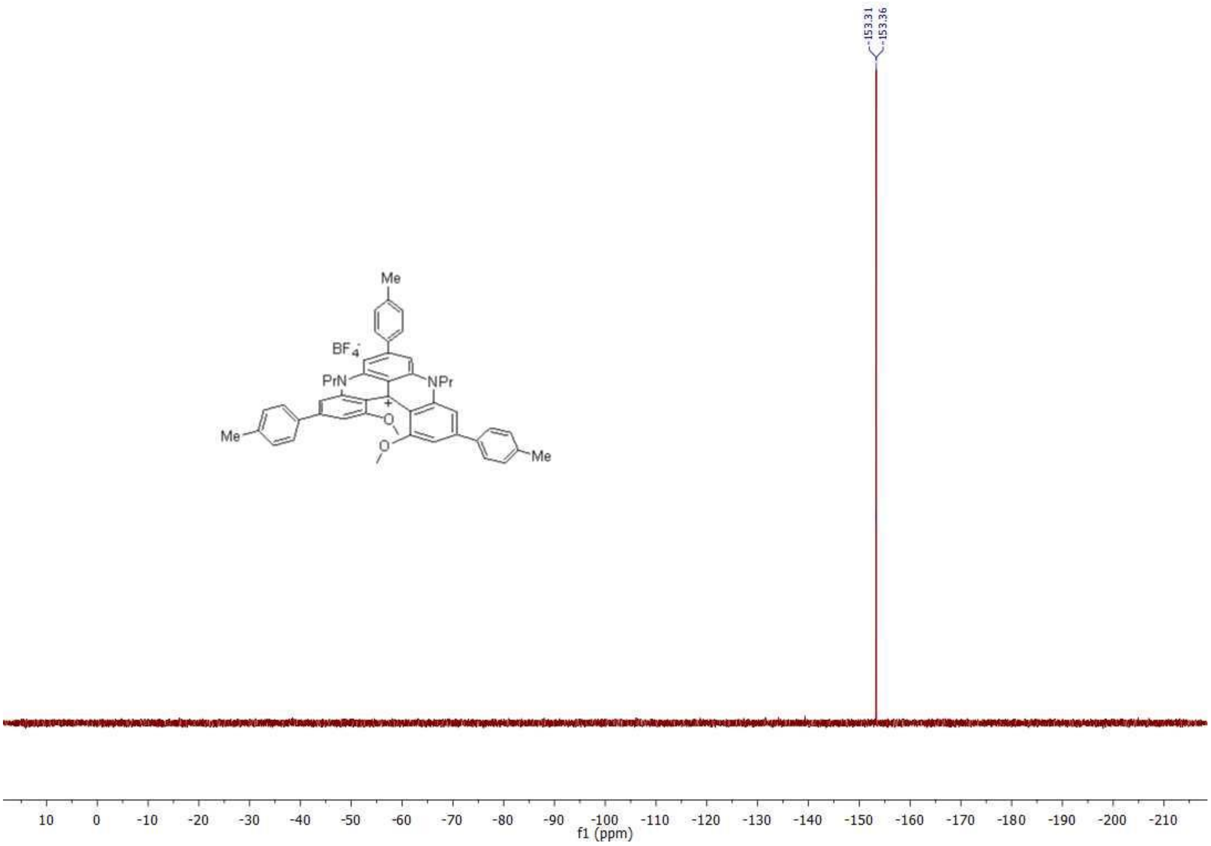

Figure S48. ^{19}F-NMR spectra of **6i** in CD_2Cl_2, 282 MHz.

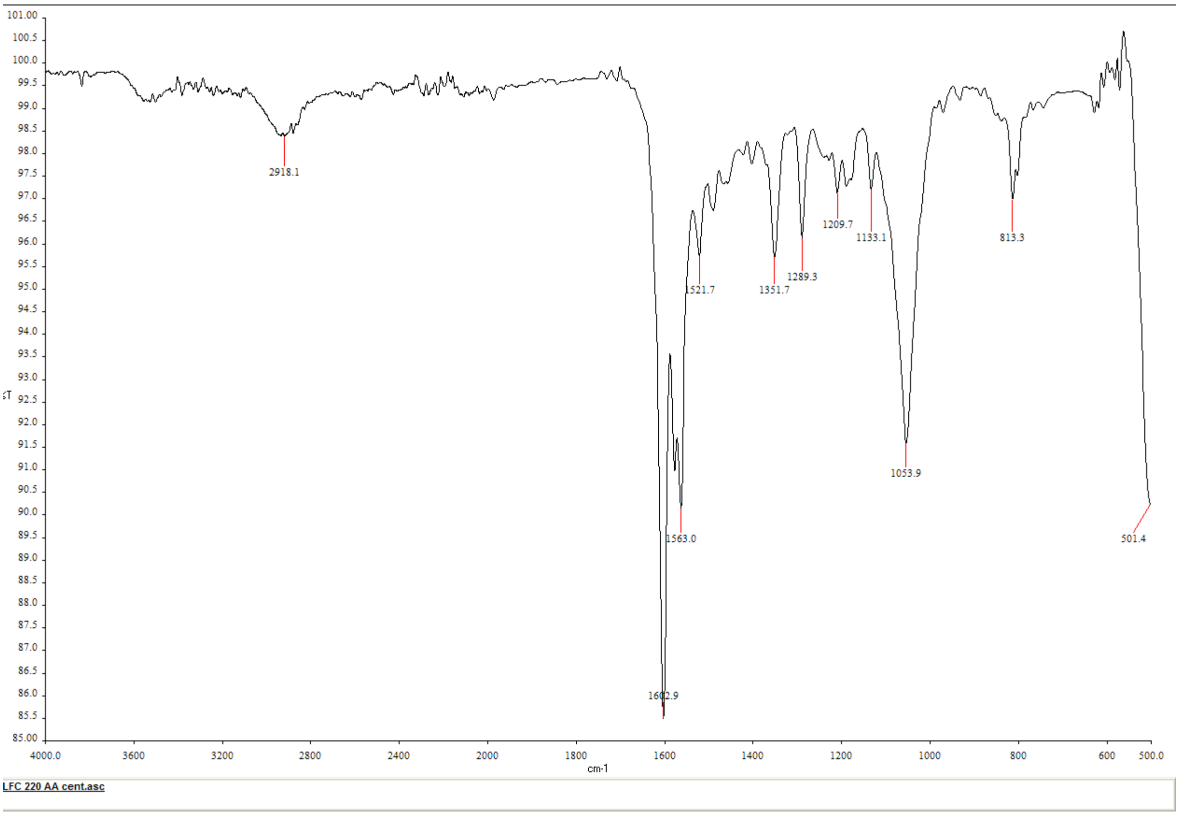

Figure S49. IR spectrum (neat) of **6i**.

Mass Spectrometry Core Facility

Lacour Group – University of Geneva

UNIVERSITÉ DE GENÈVE
FACULTÉ DES SCIENCES

ESI-HRMS – Certificate of Analysis

Applicant:	Lucas Frederic	Date of certificate:	06/09/21
Sample name:	LFC-220	Instrument:	Xevo G2 Tof (TOF)
Folder:	060921.PRO	Mobile phase:	MeOH (100 µl/min)
Analyst:	Stéphane Grass	Ionisation mode:	ESI (positive polarity)

Elemental Formula	Ion type	Masslynx values *** calc. m/z	Masslynx values *** meas. m/z	Calc. *m/z*	Meas. *m/z*	Accuracy [a] (ppm)
$C_{48}H_{47}N_2O_2$	$[M]^+$	683.3638	683.3649	683.3633	683.3644	1.6

[a] *Mass spectrum is calibrated by the use of the MS lockspray system (LeuEnk calibration solution).*

**** MassLynx software does not take into account the mass of the electron for ionic species, therefore the shift of m/z 0.000459.*

Zoomed mass spectrum – Isotopic distribution.

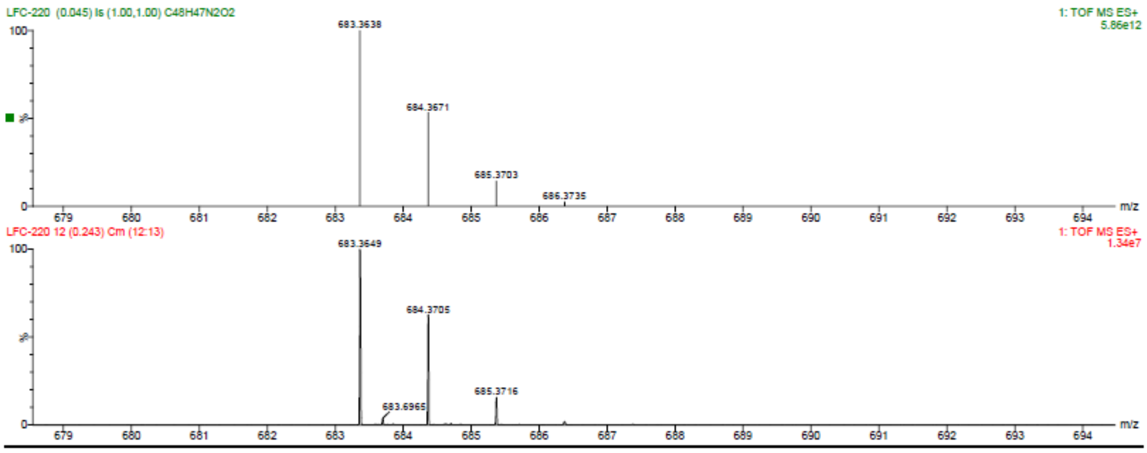

Figure S50. HRMS analysis (ESI, MeOH) report of **6i**.

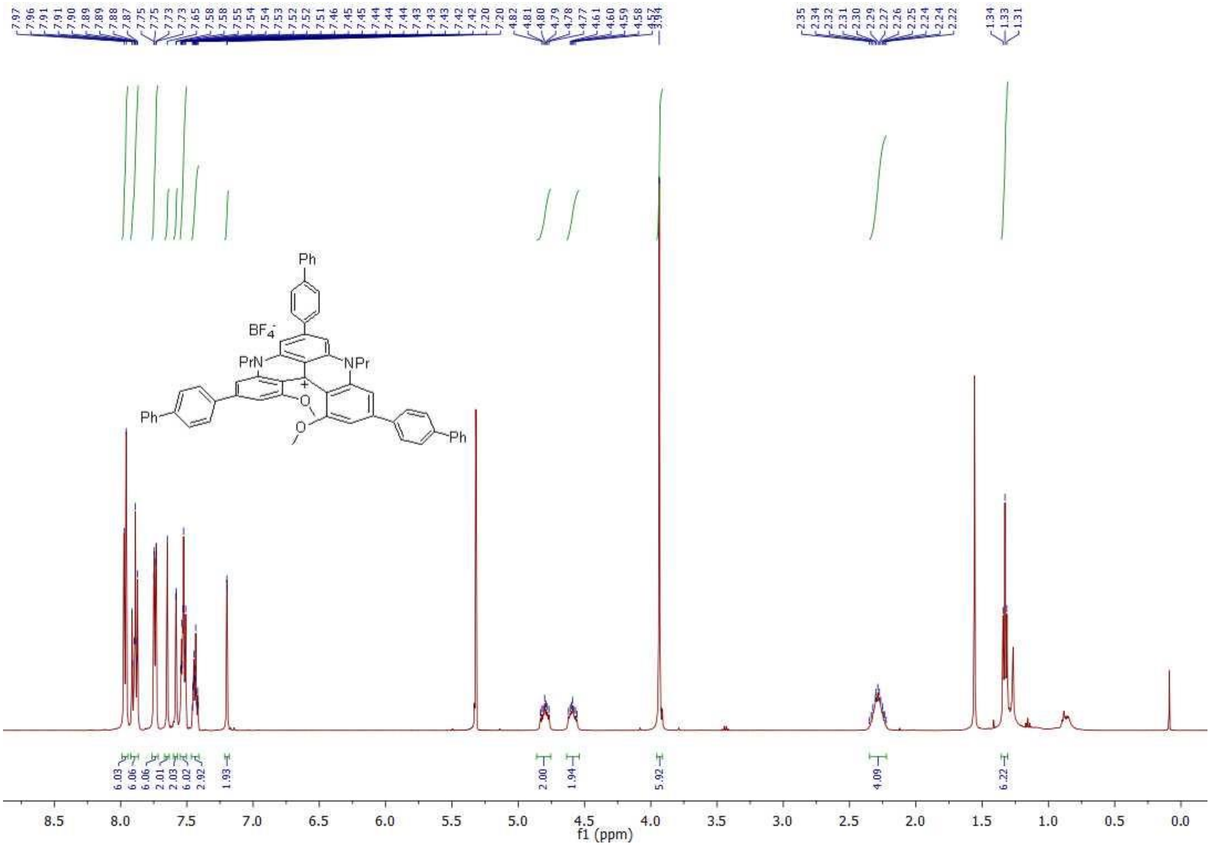

Figure S51. ^{1}H-NMR spectra of **6j** in CD_2Cl_2, 500 MHz.

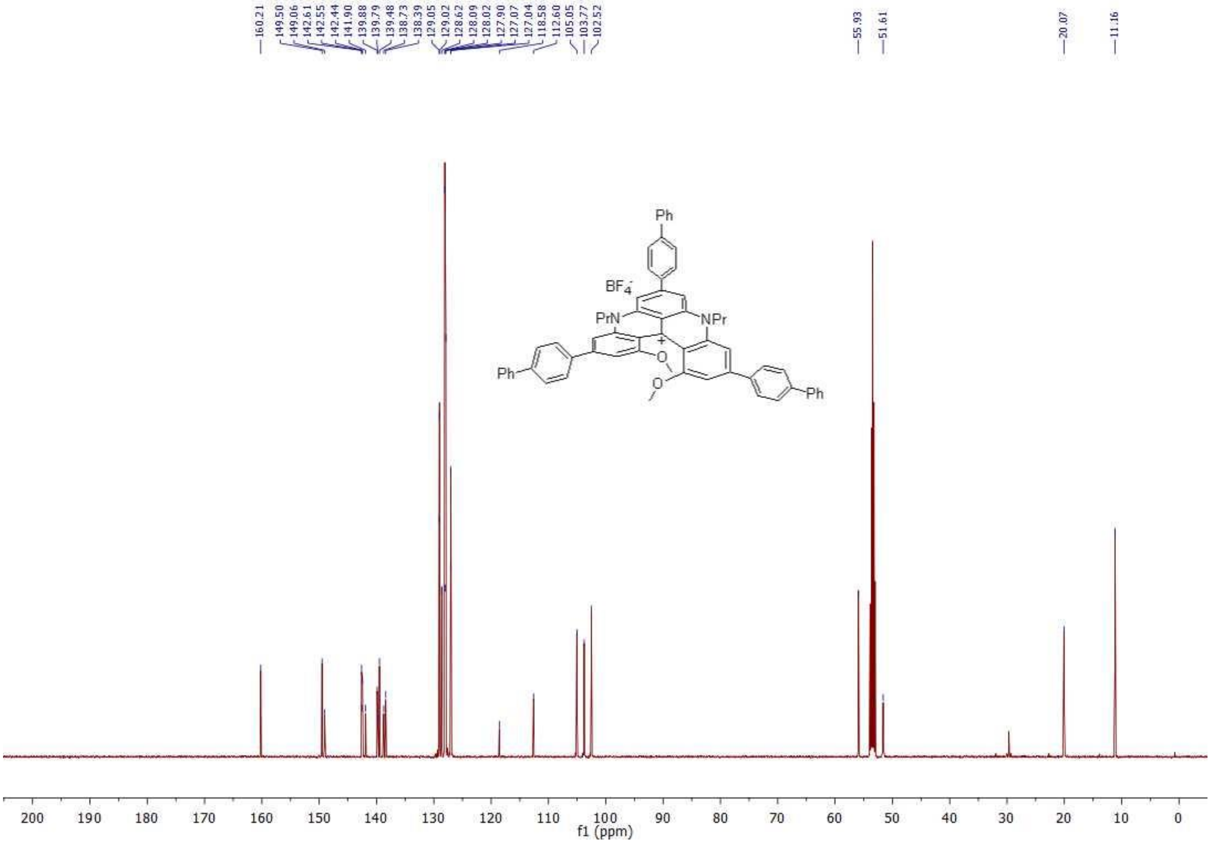

Figure S52. ^{13}C-NMR spectra of **6j** in CD_2Cl_2, 125 MHz.

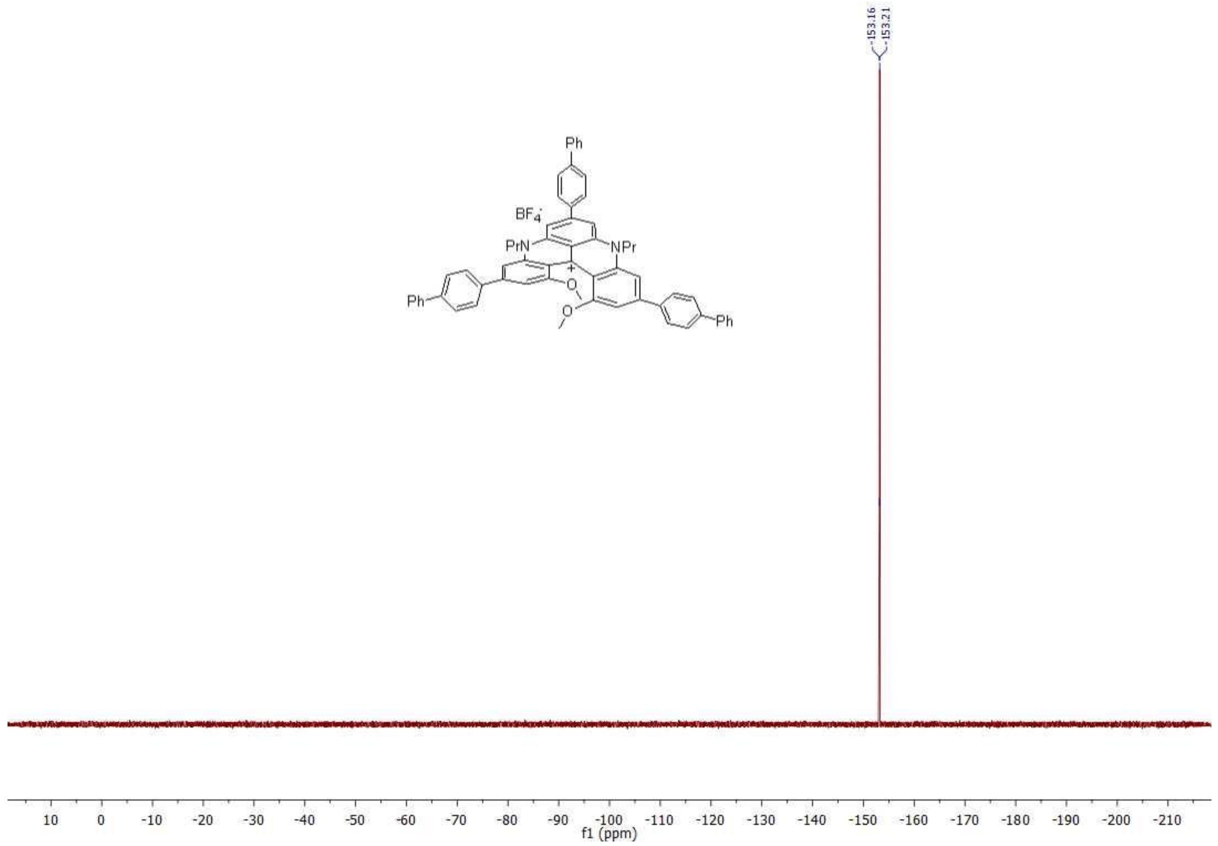

Figure S53. ^{19}F-NMR spectra of **6j** in CD_2Cl_2, 282 MHz.

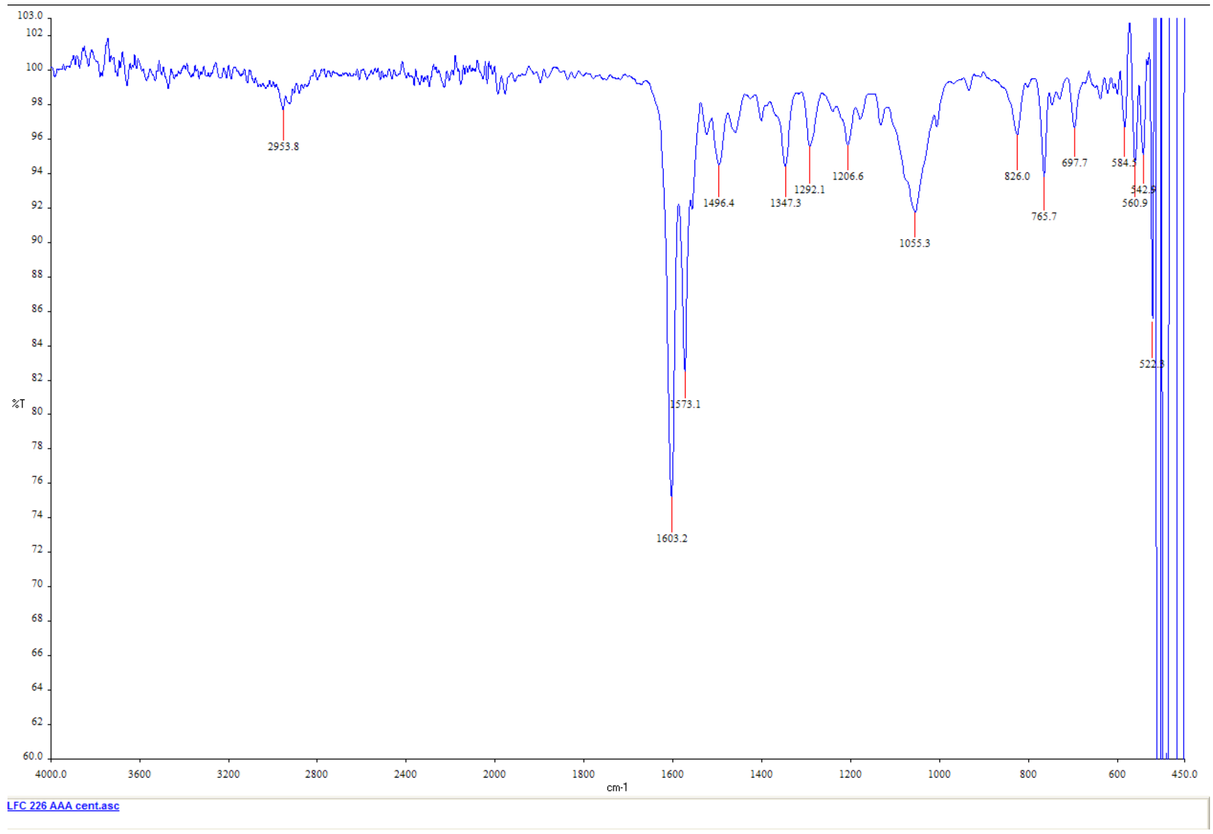

Figure S54. IR spectrum (neat) of **6j**.

Mass Spectrometry Core Facility

Lacour Group – University of Geneva

UNIVERSITÉ DE GENÈVE
FACULTÉ DES SCIENCES

ESI-HRMS – Certificate of Analysis

Applicant:	Lucas Frederic	Date of certificate:	06/09/21
Sample name:	LFC-226	Instrument:	Xevo G2 Tof (TOF)
Folder:	060921.PRO	Mobile phase:	MeOH (100 µl/min)
Analyst:	Stéphane Grass	Ionisation mode:	ESI (positive polarity)

Elemental Formula	Ion type	*Masslynx values* *** *calc. m/z*	*meas. m/z*	Calc. *m/z*	Meas. *m/z*	Accuracy [a] *(ppm)*
$C_{63}H_{53}N_2O_2$	$[M]^+$	*869.4107*	*869.4095*	869.4102	869.4090	-1.4

[a] *Mass spectrum is calibrated by the use of the MS lockspray system (LeuEnk calibration solution).*

**** MassLynx software does not take into account the mass of the electron for ionic species, therefore the shift of m/z 0.000459.*

Zoomed mass spectrum – Isotopic distribution.

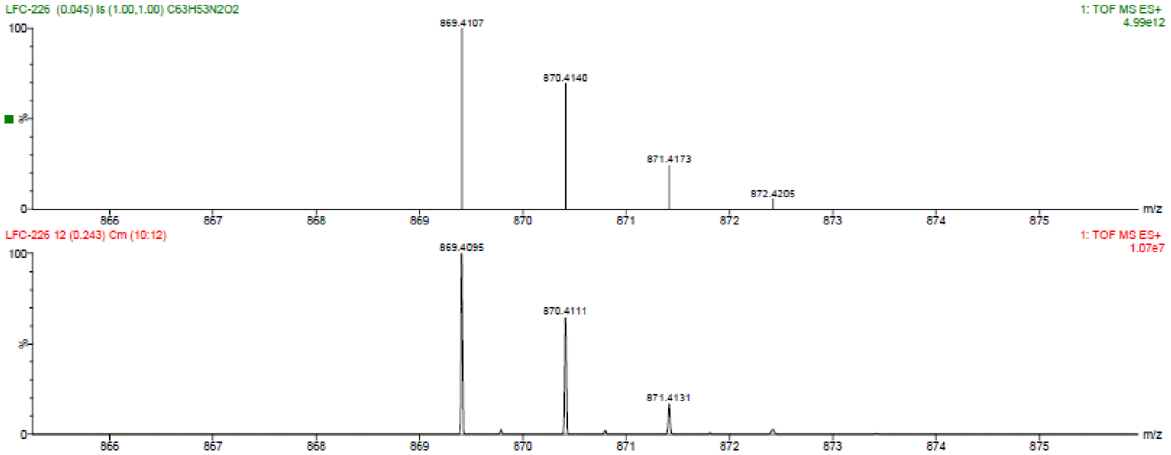

Figure S55. HRMS analysis (ESI, MeOH) report of **6j**.

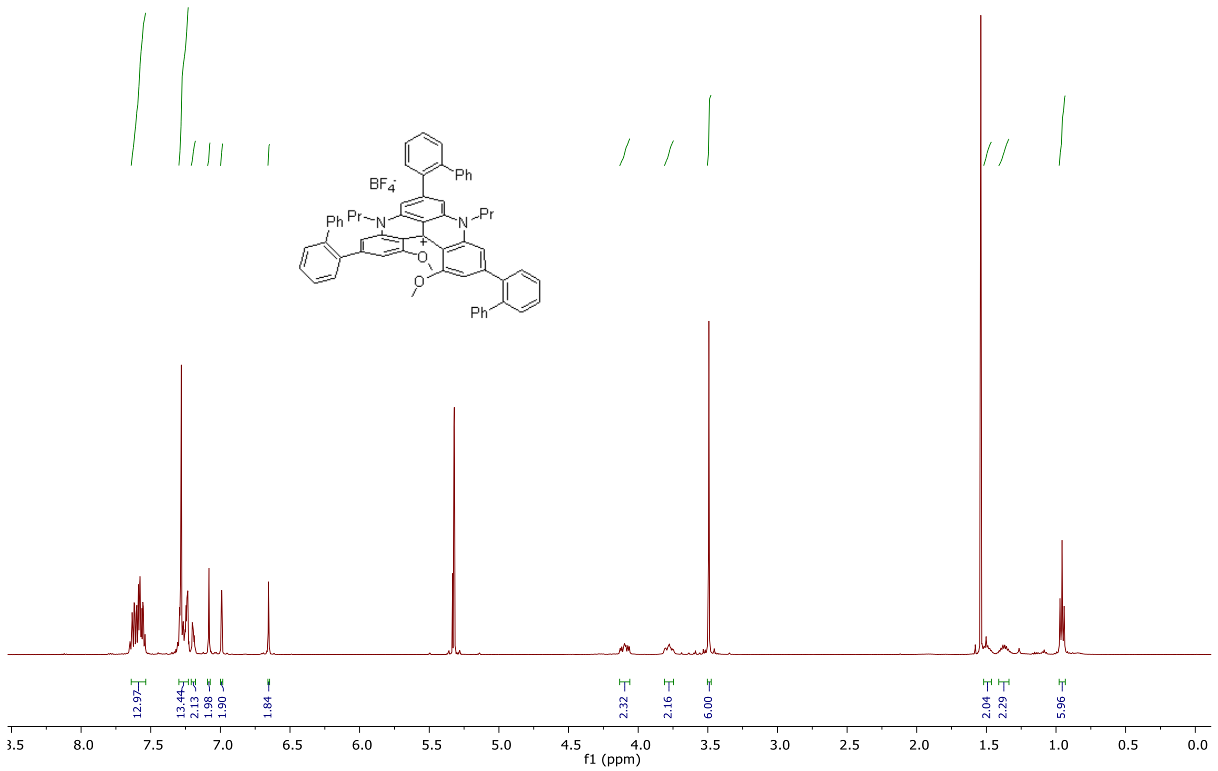

Figure S56. ^{1}H-NMR spectra of **6k** in CD_2Cl_2, 500 MHz.

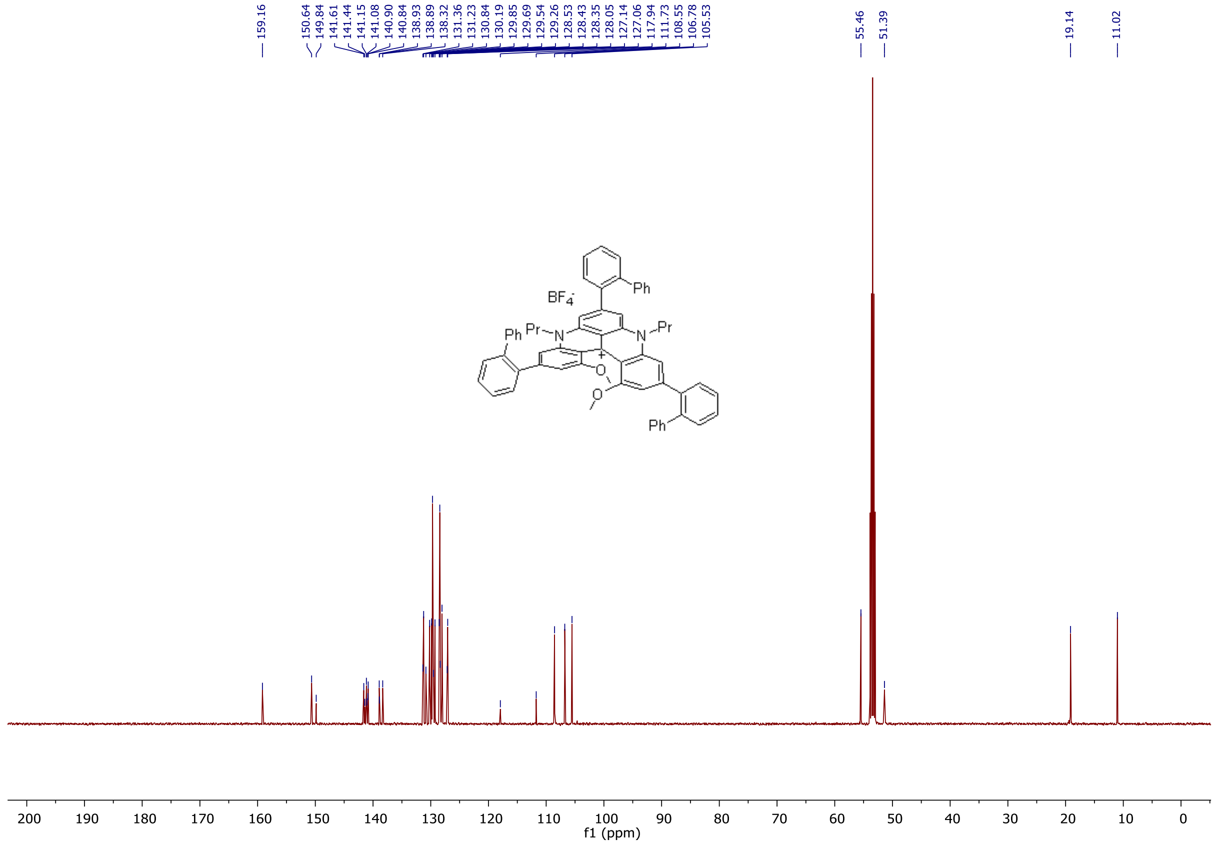

Figure S57. ^{13}C-NMR spectra of **6k** in CD_2Cl_2, 125 MHz.

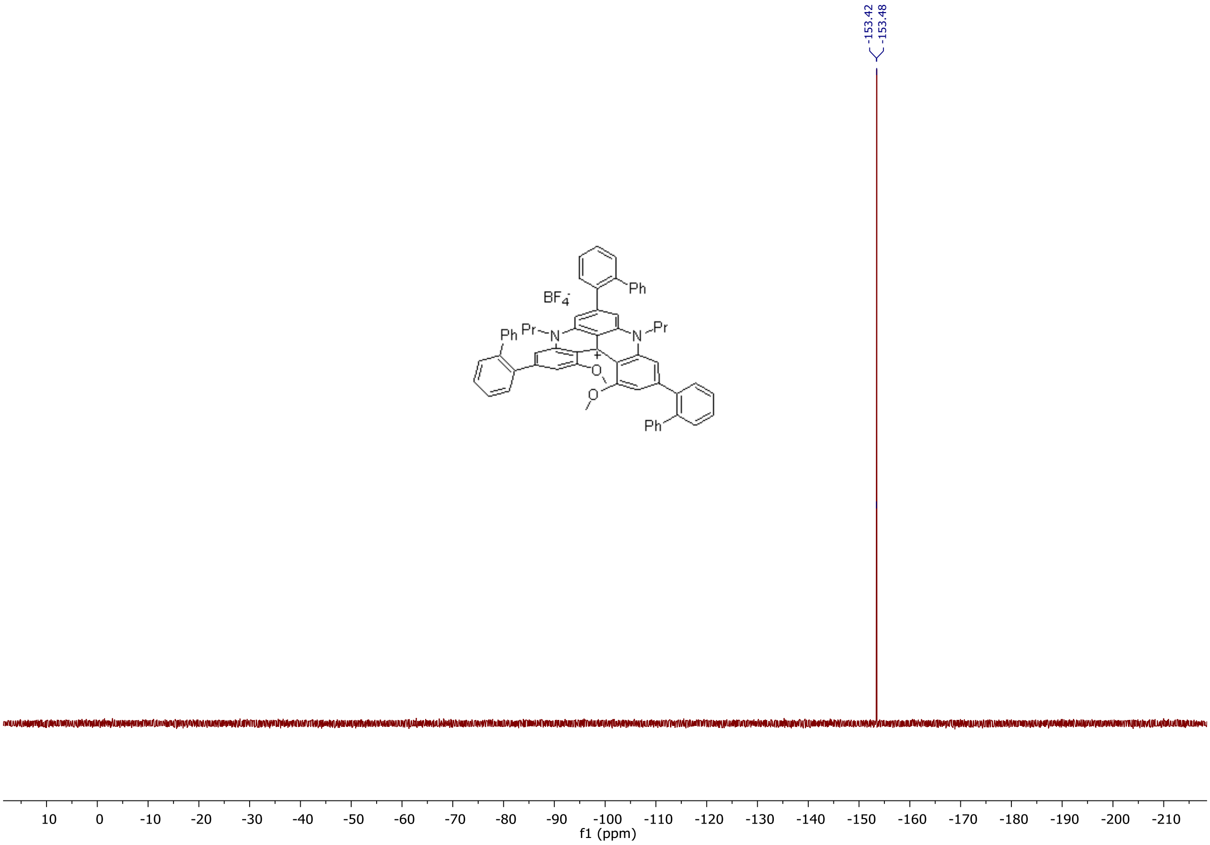

Figure S58. ^{19}F-NMR spectra of **6k** in CD_2Cl_2, 282 MHz.

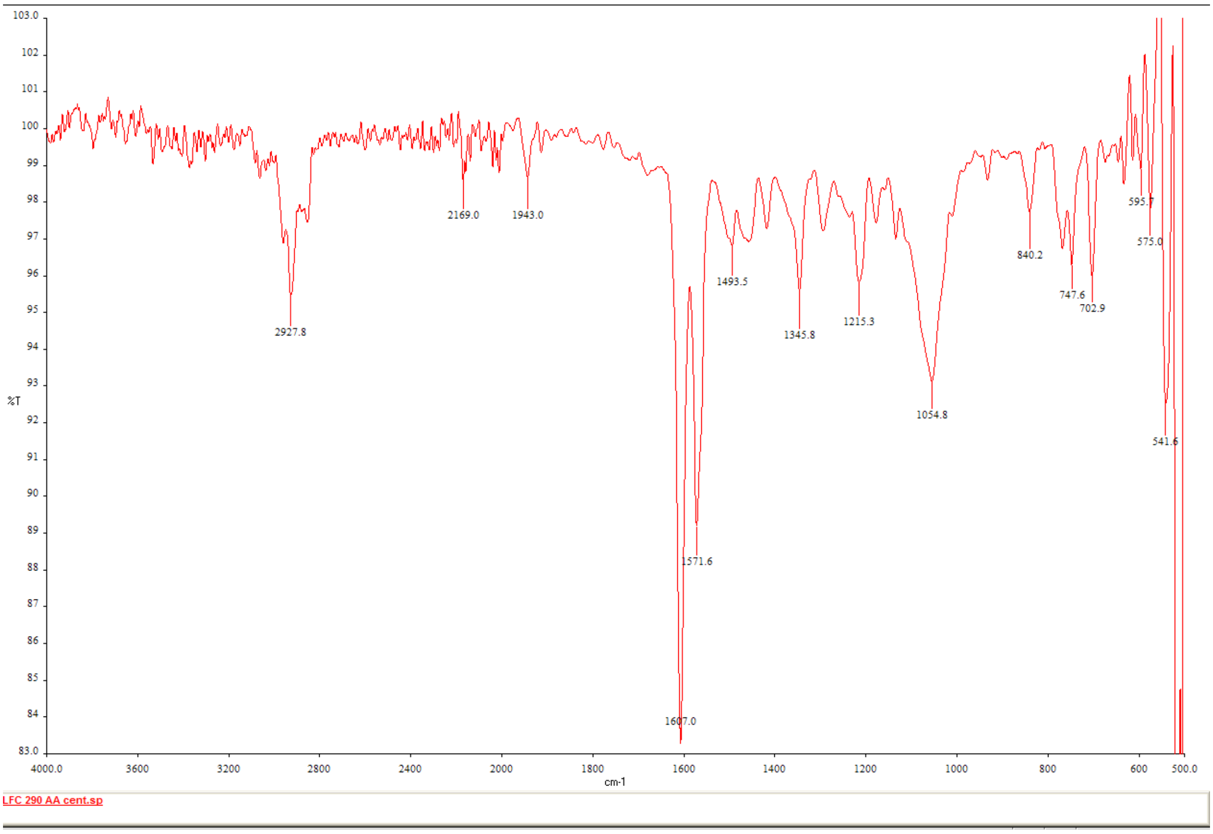

Figure S59. IR spectrum (neat) of **6k**.

Mass Spectrometry Core Facility

Lacour Group – University of Geneva

UNIVERSITÉ DE GENÈVE
FACULTÉ DES SCIENCES

ESI-HRMS – Certificate of Analysis

Applicant:	Lucas Frederic	Date of certificate:	06/12/21
Sample name:	LFC-290 AA	Instrument:	Xevo G2 Tof (TOF)
Folder:	061221.PRO	Mobile phase:	MeOH (100 µl/min)
Analyst:	Stéphane Grass	Ionisation mode:	ESI (positive polarity)

Elemental Formula	Ion type	Masslynx values *** calc. m/z	Masslynx values *** meas. m/z	Calc. *m/z*	Meas. *m/z*	Accuracy [a)] (ppm)
$C_{63}H_{53}N_2O_2$	$[M]^+$	869.4107	869.4095	869.4102	869.4090	-1.4

[a)] *Mass spectrum is calibrated by the use of the MS lockspray system (LeuEnk calibration solution).*

**** MassLynx software does not take into account the mass of the electron for ionic species, therefore the shift of m/z 0.000459.*

Zoomed mass spectrum – Isotopic distribution.

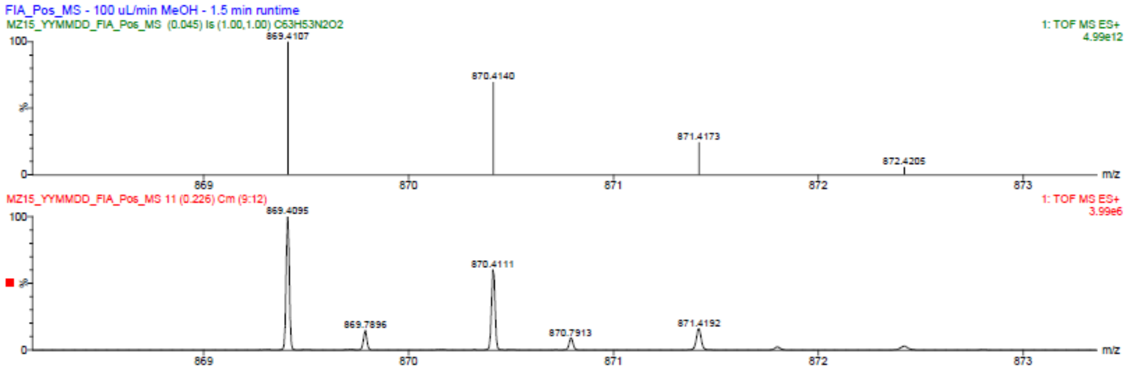

Figure S60. HRMS analysis (ESI, MeOH) report of **6k**.

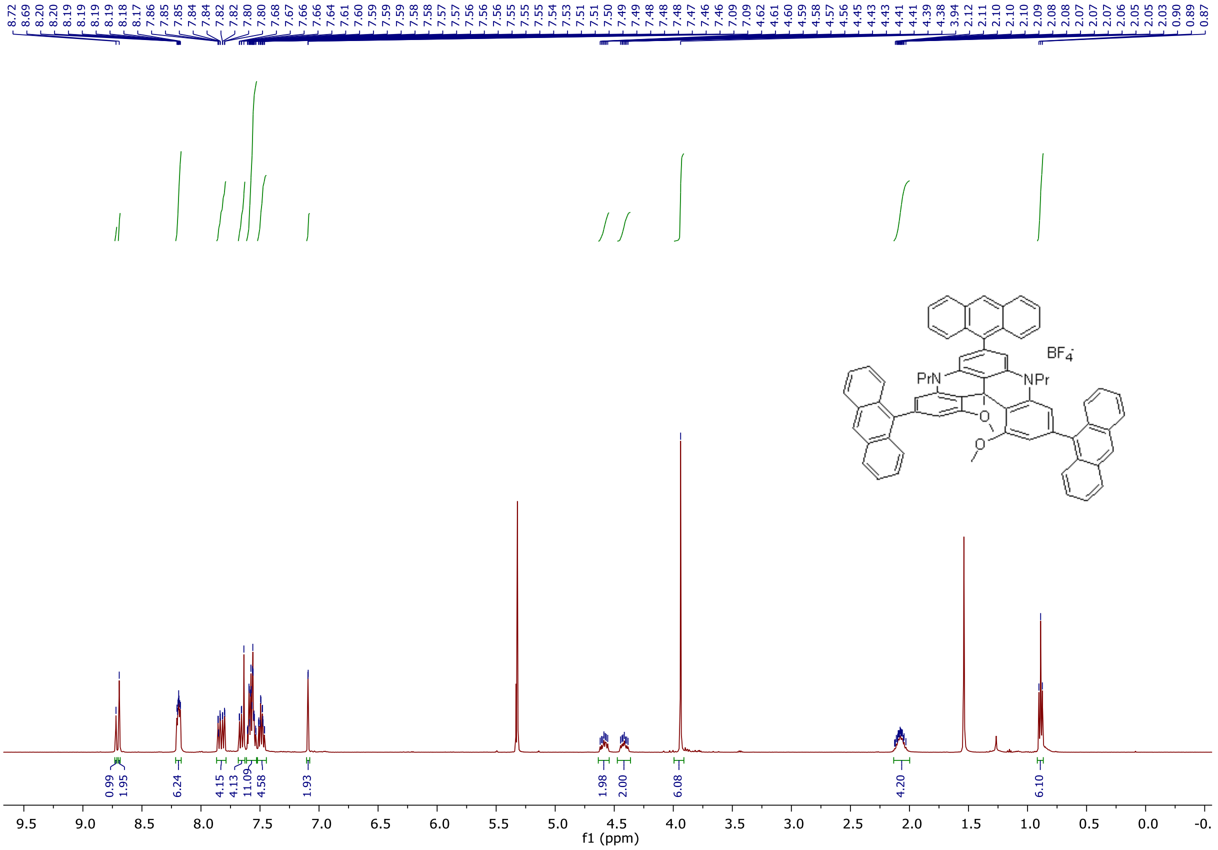

Figure S61. ^{1}H-NMR spectra of **6l** in CD_2Cl_2, 500 MHz.

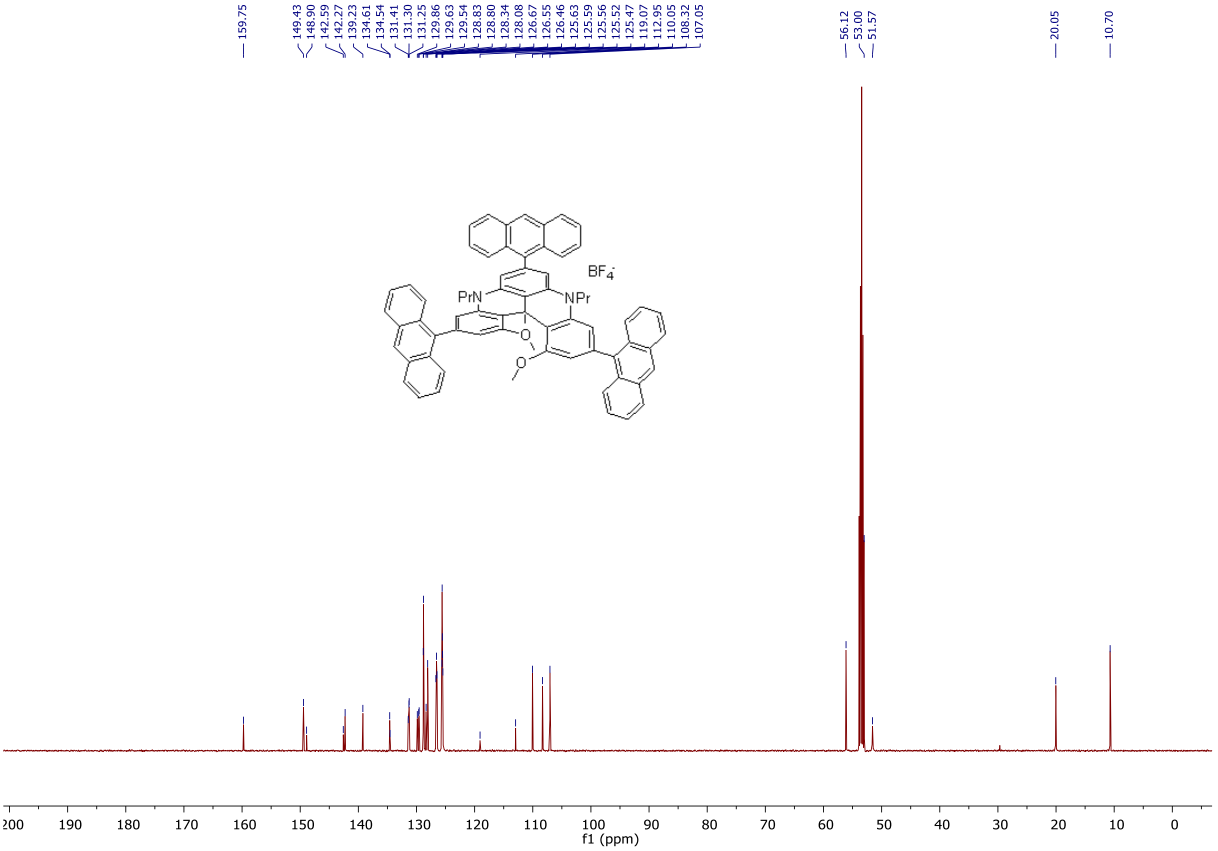

Figure S62. ^{13}C-NMR spectra of **6l** in CD_2Cl_2, 125 MHz.

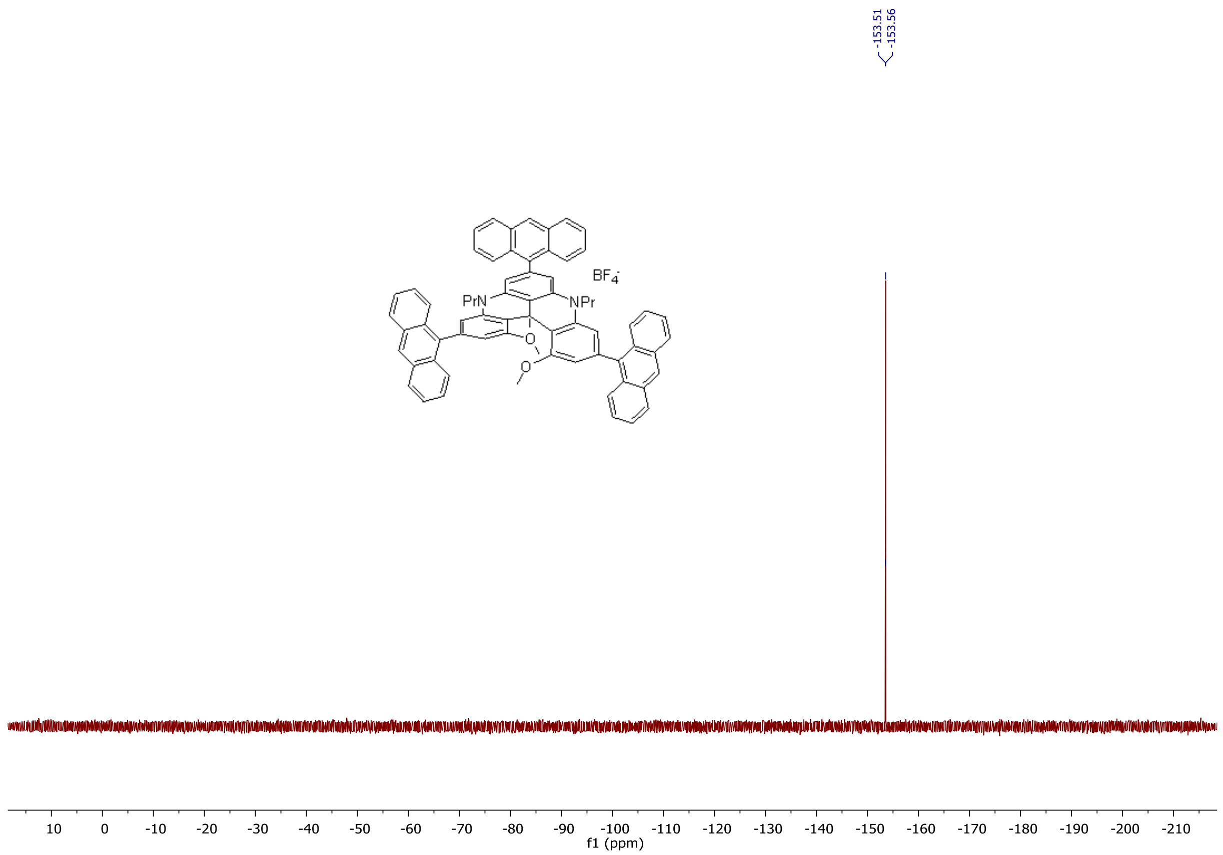

Figure S63. ^{19}F-NMR spectra of **6l** in CD_2Cl_2, 282 MHz.

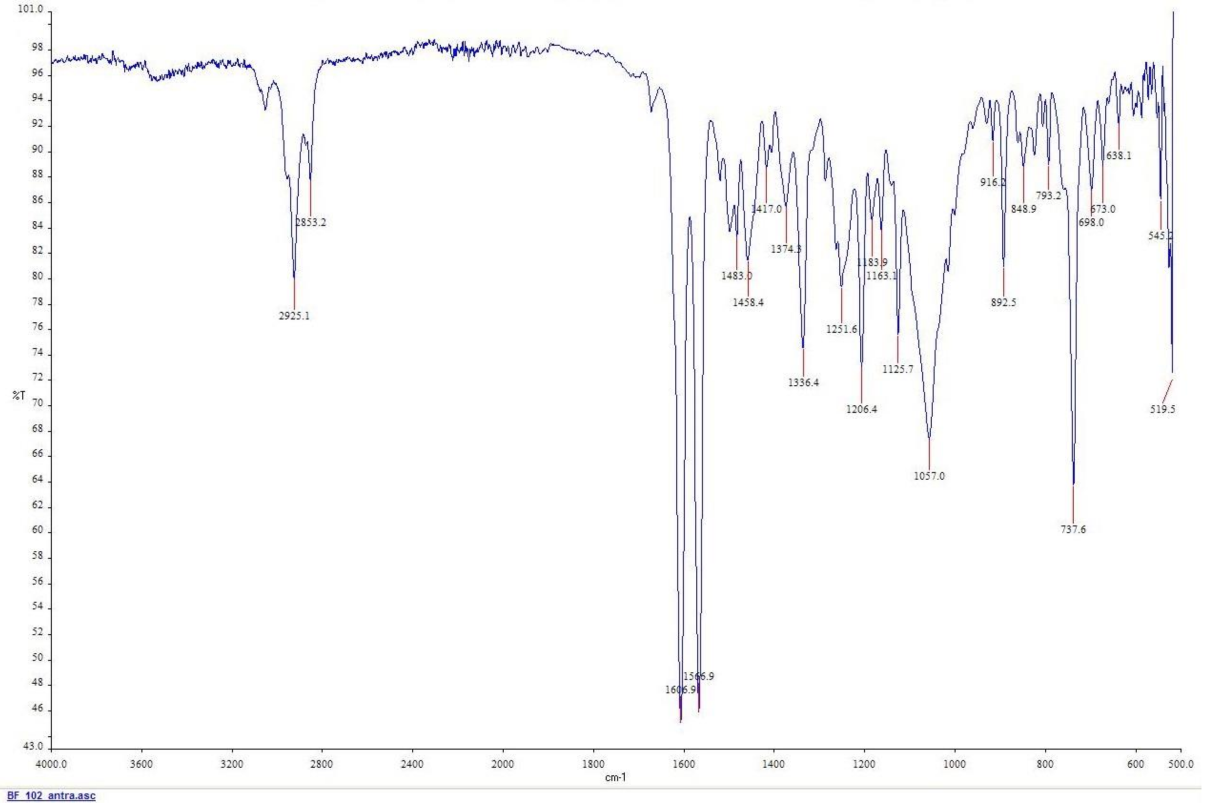

Figure S64. IR spectrum (neat) of **6l**.

Mass Spectrometry Core Facility

Lacour Group – University of Geneva

UNIVERSITÉ DE GENÈVE
FACULTÉ DES SCIENCES

ESI-HRMS – Certificate of Analysis

Applicant:	Bibiana Fabri	Date of certificate:	01/03/22
Sample name:	BF-antra	Instrument:	Xevo G2 Tof (TOF)
Folder:	010322.PRO	Mobile phase:	MeOH (100 µl/min)
Analyst:	Stéphane Grass	Ionisation mode:	ESI (positive polarity)

Elemental Formula	Ion type	Masslynx values *** calc. *m/z*	Masslynx values *** meas. *m/z*	Calc. *m/z*	Meas. *m/z*	Accuracy [a] (ppm)
$C_{69}H_{53}N_2O_2$	[M+]	*941.4107*	*941.4080*	941.4102	941.4075	-2.9

[a] *Mass spectrum is calibrated by the use of the MS lockspray system (LeuEnk calibration solution).*

******* *MassLynx software does not take into account the mass of the electron for ionic species, therefore the shift of m/z 0.000459.*

Zoomed mass spectrum – Isotopic distribution.

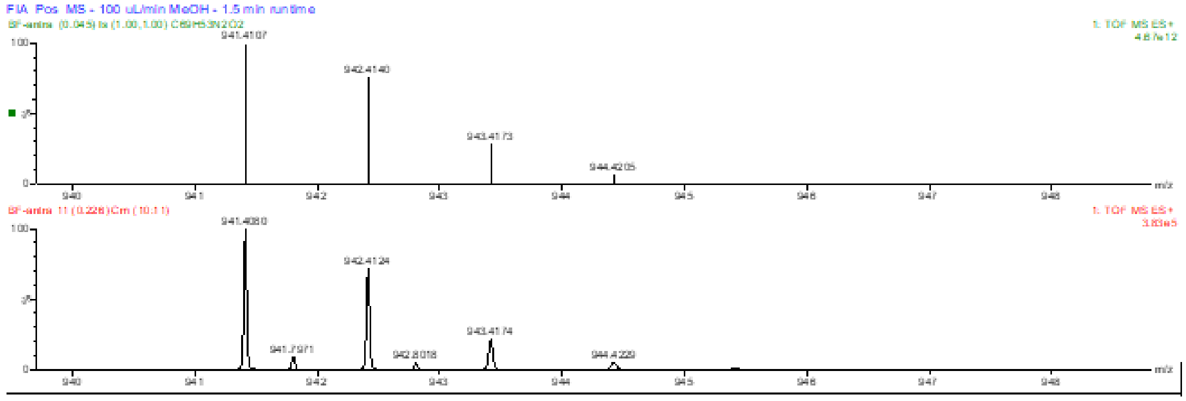

Figure S65. HRMS analysis (ESI, MeOH) report of **6l**.

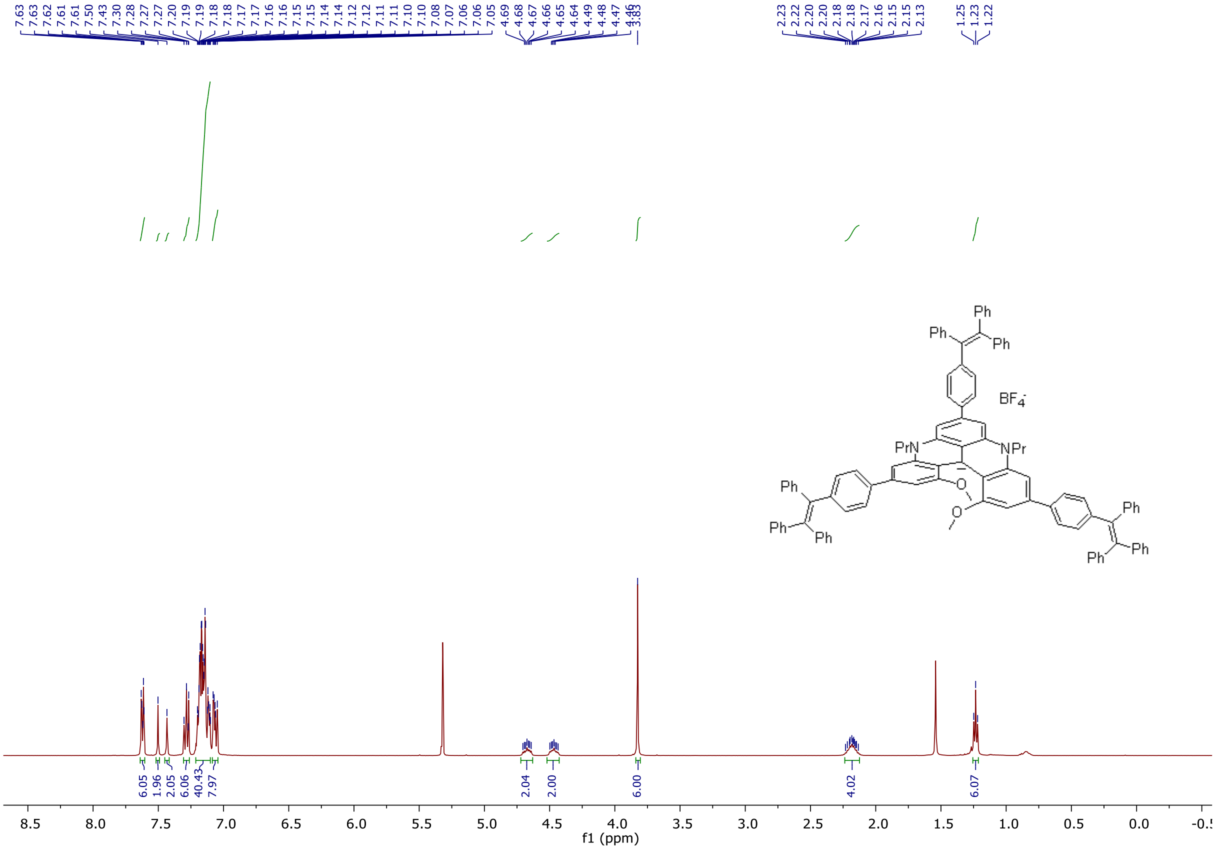

Figure S66. ^{1}H-NMR spectra of **6m** in CD_2Cl_2, 500 MHz.

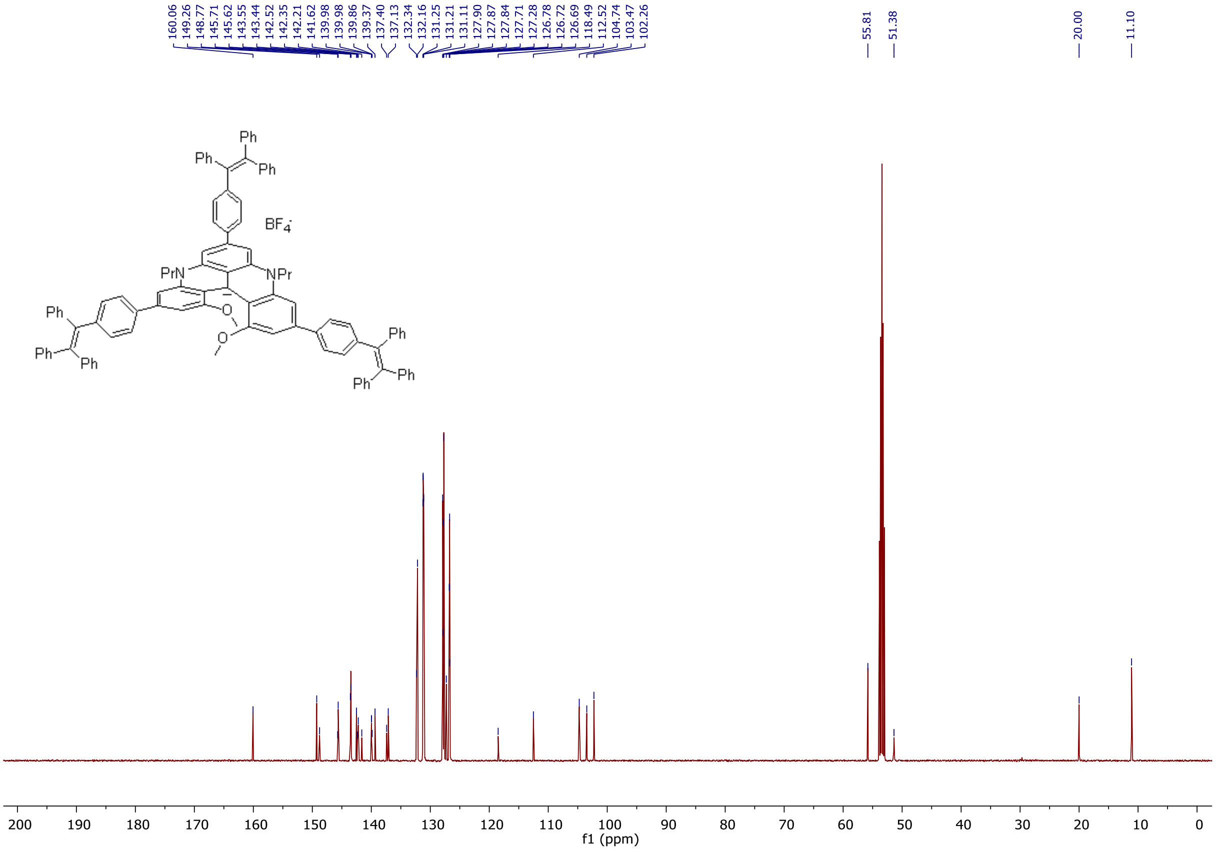

Figure S67. ^{13}C-NMR spectra of **6m** in CD_2Cl_2, 125 MHz.

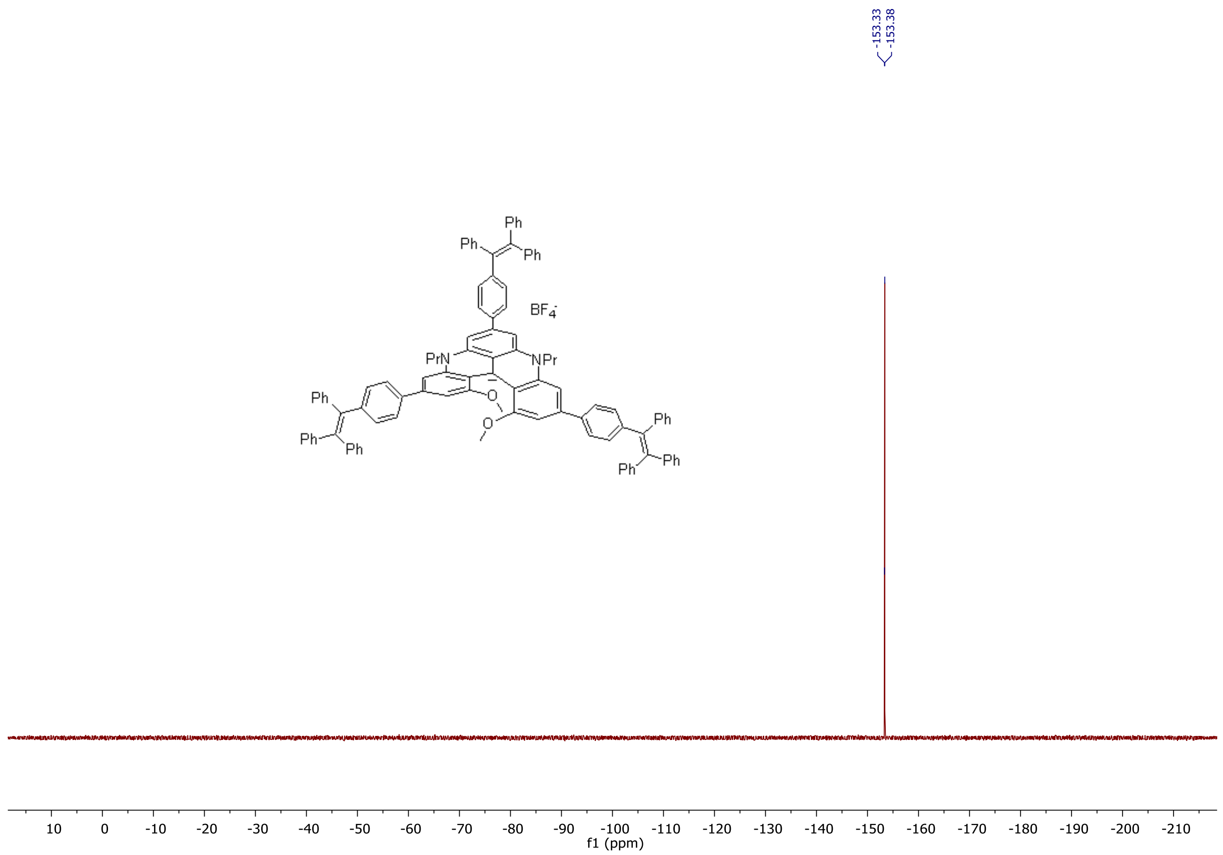

Figure S68. ^{19}F-NMR spectra of **6m** in CD_2Cl_2, 282 MHz.

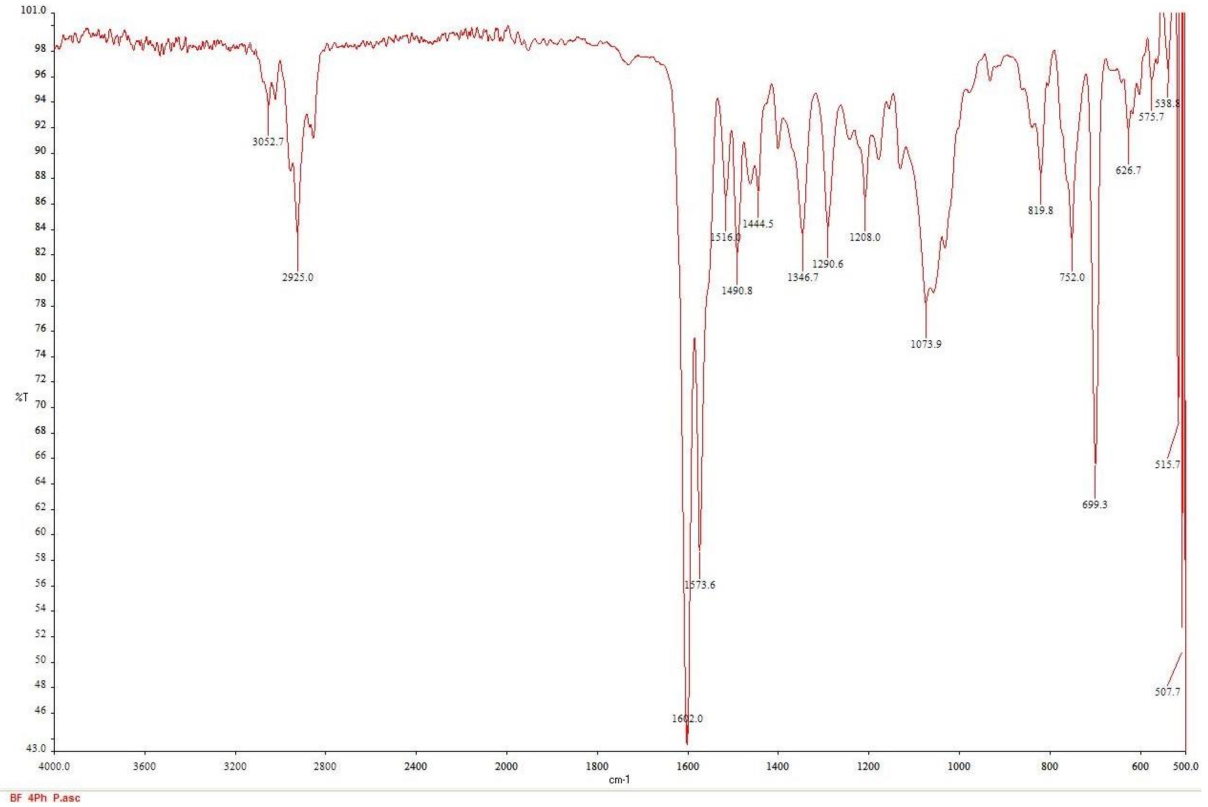

Figure S69. IR spectrum (neat) of **6m**.

Mass Spectrometry Core Facility

Lacour Group – University of Geneva

UNIVERSITÉ DE GENÈVE
FACULTÉ DES SCIENCES

ESI-HRMS – Certificate of Analysis

Applicant:	Bibiana Fabri	Date of certificate:	01/03/22
Sample name:	BF-4Ph	Instrument:	Xevo G2 Tof (TOF)
Folder:	010322.PRO	Mobile phase:	MeOH (100 µl/min)
Analyst:	Stéphane Grass	Ionisation mode:	ESI (positive polarity)

Elemental Formula	Ion type	*Masslynx values* *** *calc. m/z*	*meas. m/z*	Calc. *m/z*	Meas. *m/z*	Accuracy [a] *(ppm)*
$C_{105}H_{83}N_2O_2$	[M+]	*1403.6455*	*1403.6462*	1,403.6450	1,403.6457	0.5

[a] *Mass spectrum is calibrated by the use of the MS lockspray system (LeuEnk calibration solution).*

**** MassLynx software does not take into account the mass of the electron for ionic species, therefore the shift of m/z 0.000459.*

Zoomed mass spectrum – Isotopic distribution.

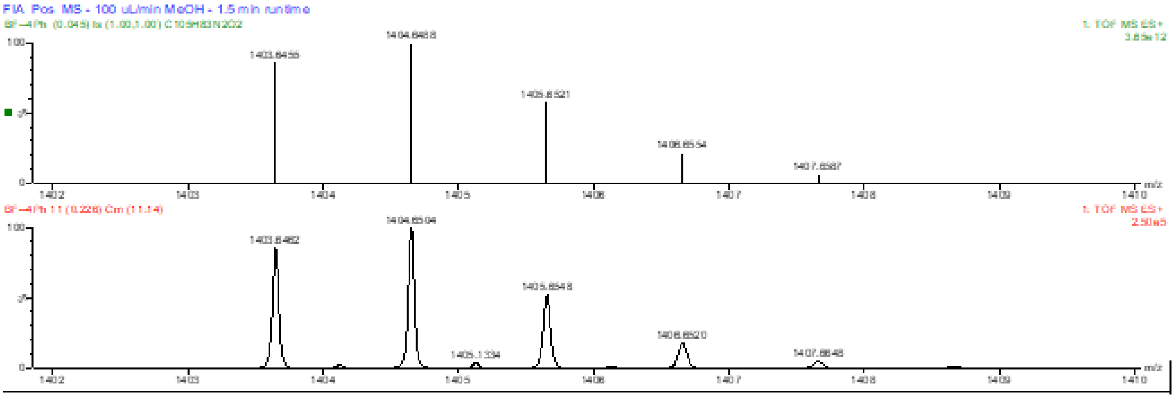

Figure S70. HRMS analysis (ESI, MeOH) report of **6m**.

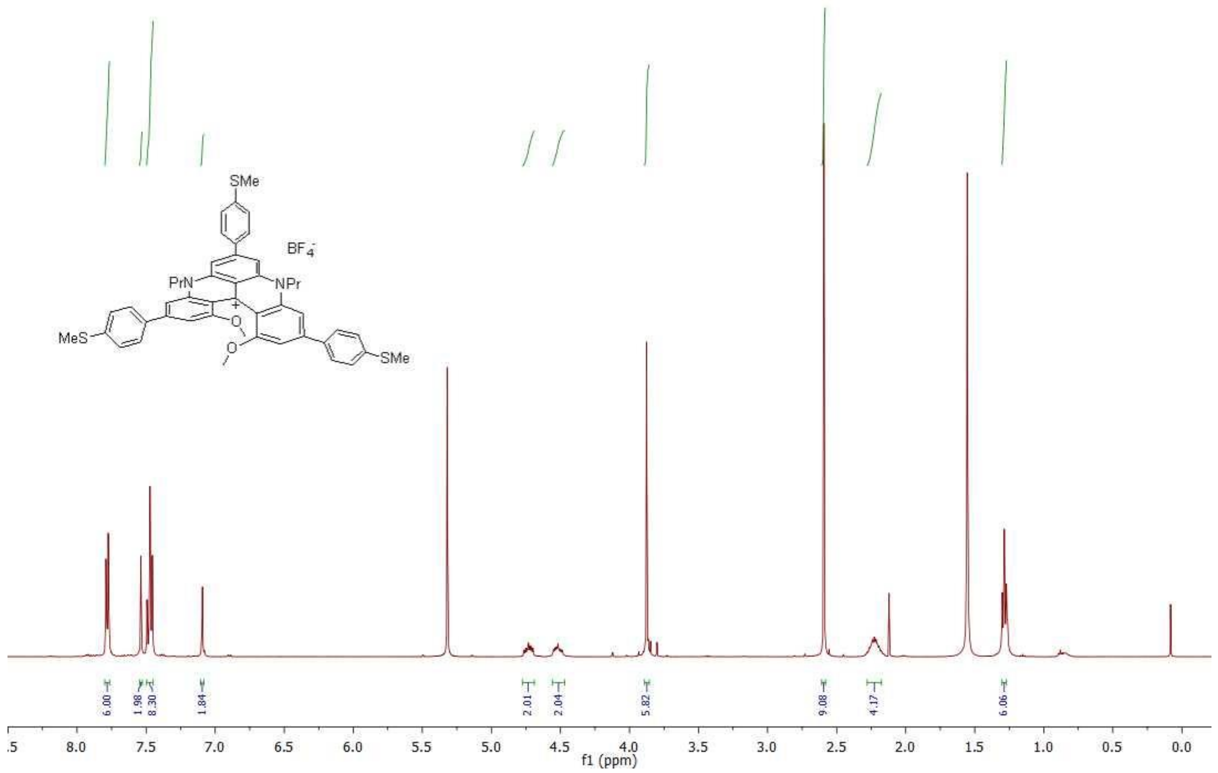

Figure S71. ^{1}H-NMR spectra of **6n** in CD_2Cl_2, 500 MHz.

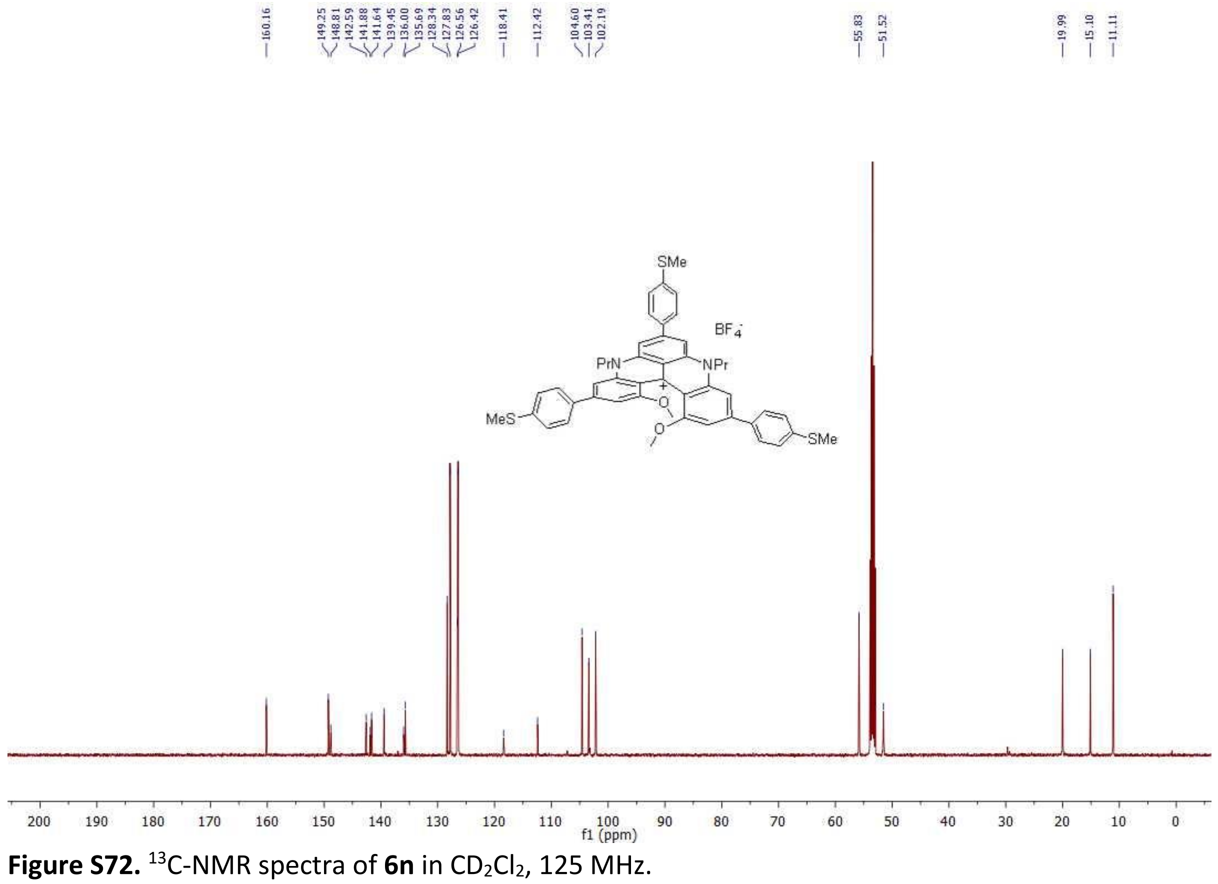

Figure S72. ^{13}C-NMR spectra of **6n** in CD_2Cl_2, 125 MHz.

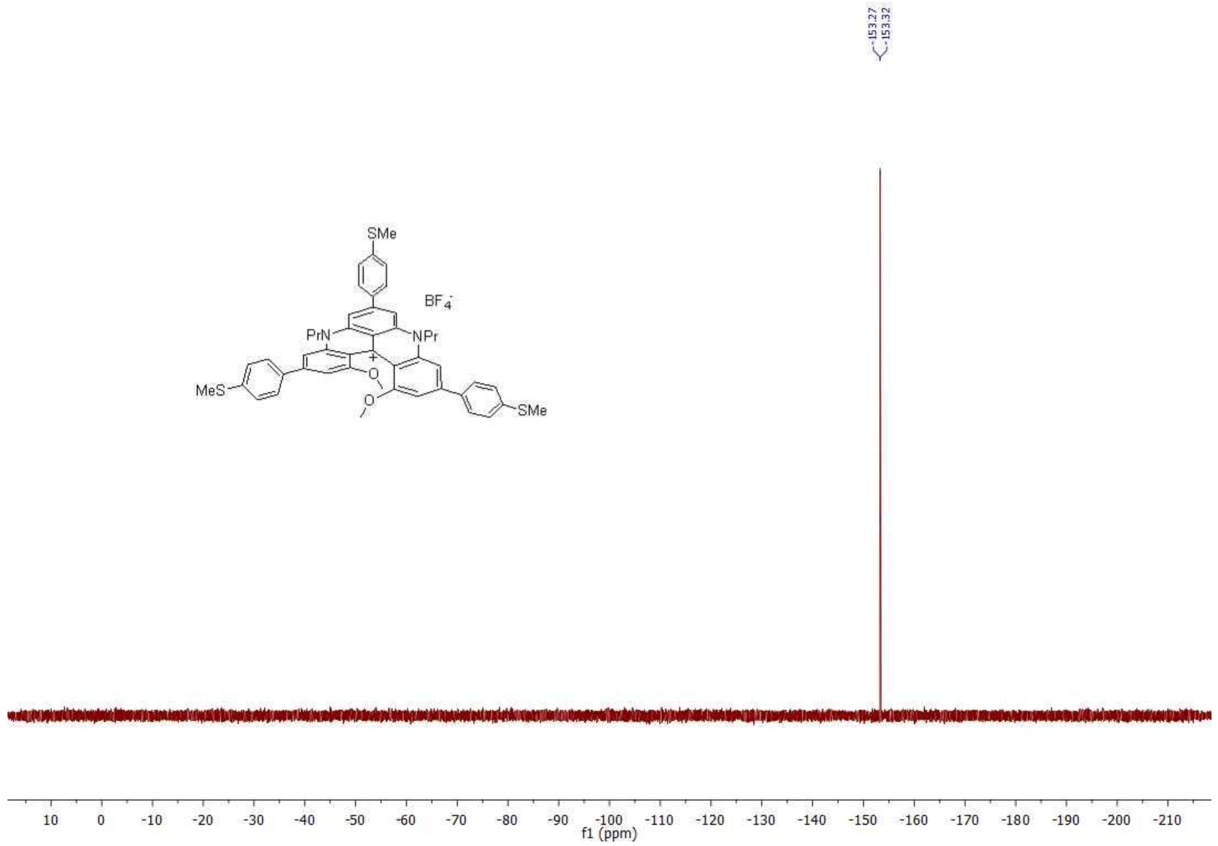

Figure S73. ^{19}F-NMR spectra of **6n** in CD_2Cl_2, 282 MHz.

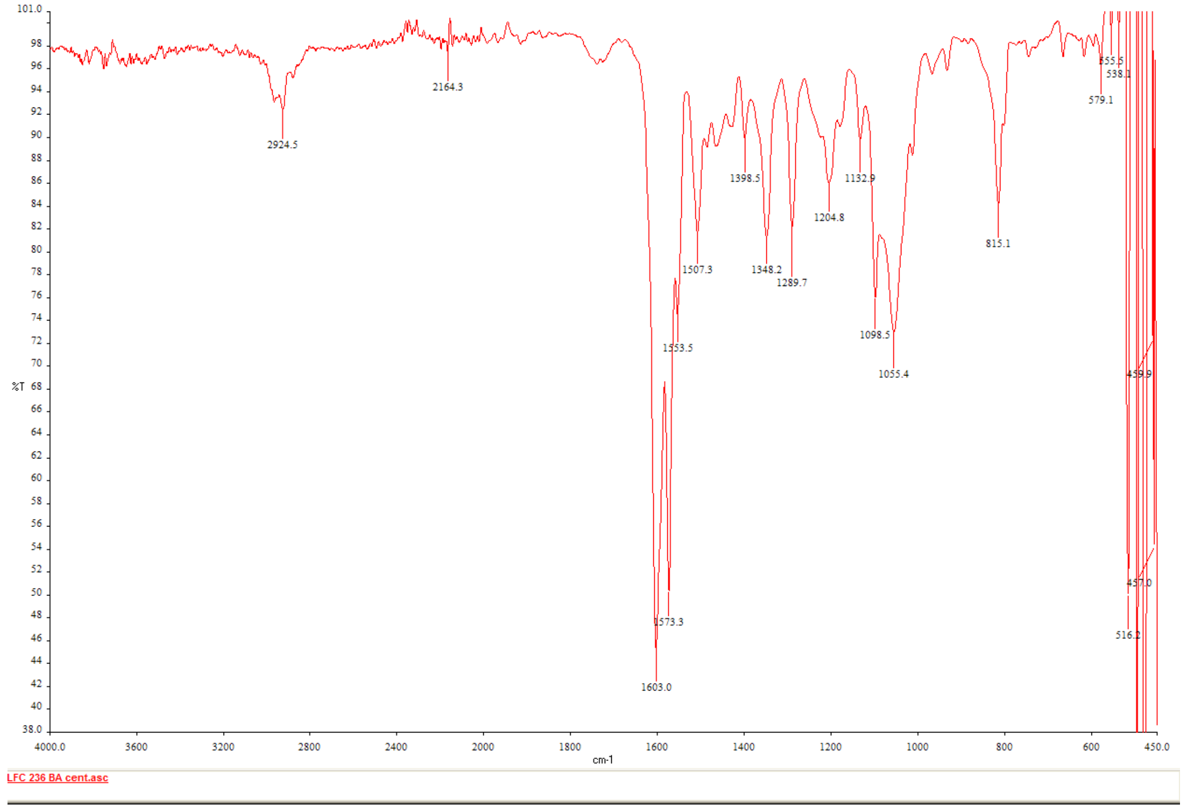

Figure S74. IR spectrum (neat) of **6n**.

Mass Spectrometry Core Facility

Lacour Group – University of Geneva

UNIVERSITÉ DE GENÈVE
FACULTÉ DES SCIENCES

ESI-HRMS – Certificate of Analysis

Applicant:	Lucas Frederic	Date of certificate:	06/09/21
Sample name:	LFC-236	Instrument:	Xevo G2 Tof (TOF)
Folder:	060921.PRO	Mobile phase:	MeOH (100 µl/min)
Analyst:	Stéphane Grass	Ionisation mode:	ESI (positive polarity)

Elemental Formula	Ion type	Masslynx values *** calc. *m/z*	Masslynx values *** meas. *m/z*	Calc. *m/z*	Meas. *m/z*	Accuracy [a] (ppm)
$C_{48}H_{47}N_2O_2S_3$	$[M]^+$	779.28	779.2820	779.2795	779.2815	2.6

[a] Mass spectrum is calibrated by the use of the MS lockspray system (LeuEnk calibration solution).

**** MassLynx software does not take into account the mass of the electron for ionic species, therefore the shift of m/z 0.000459.*

Zoomed mass spectrum – Isotopic distribution.

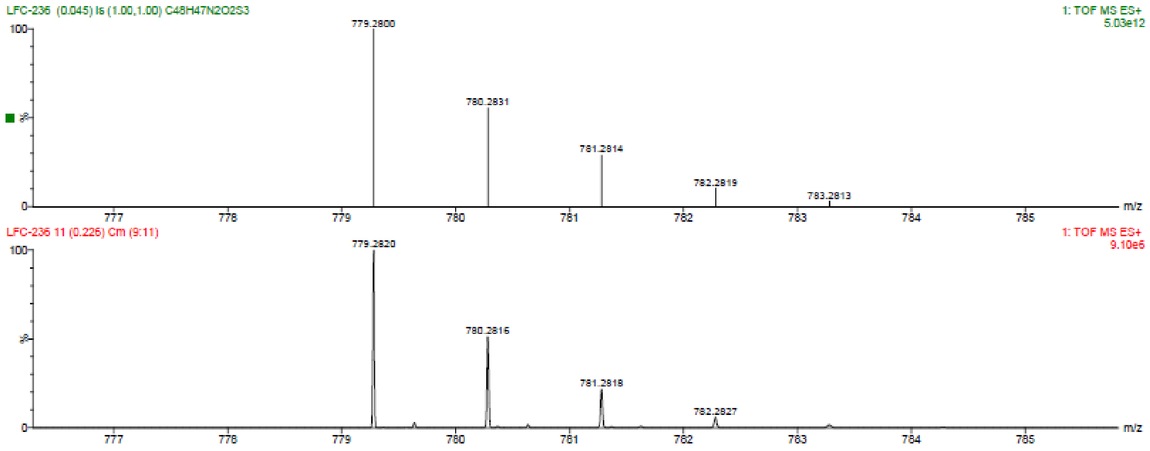

Figure S75. HRMS analysis (ESI, MeOH) report of **6n**.

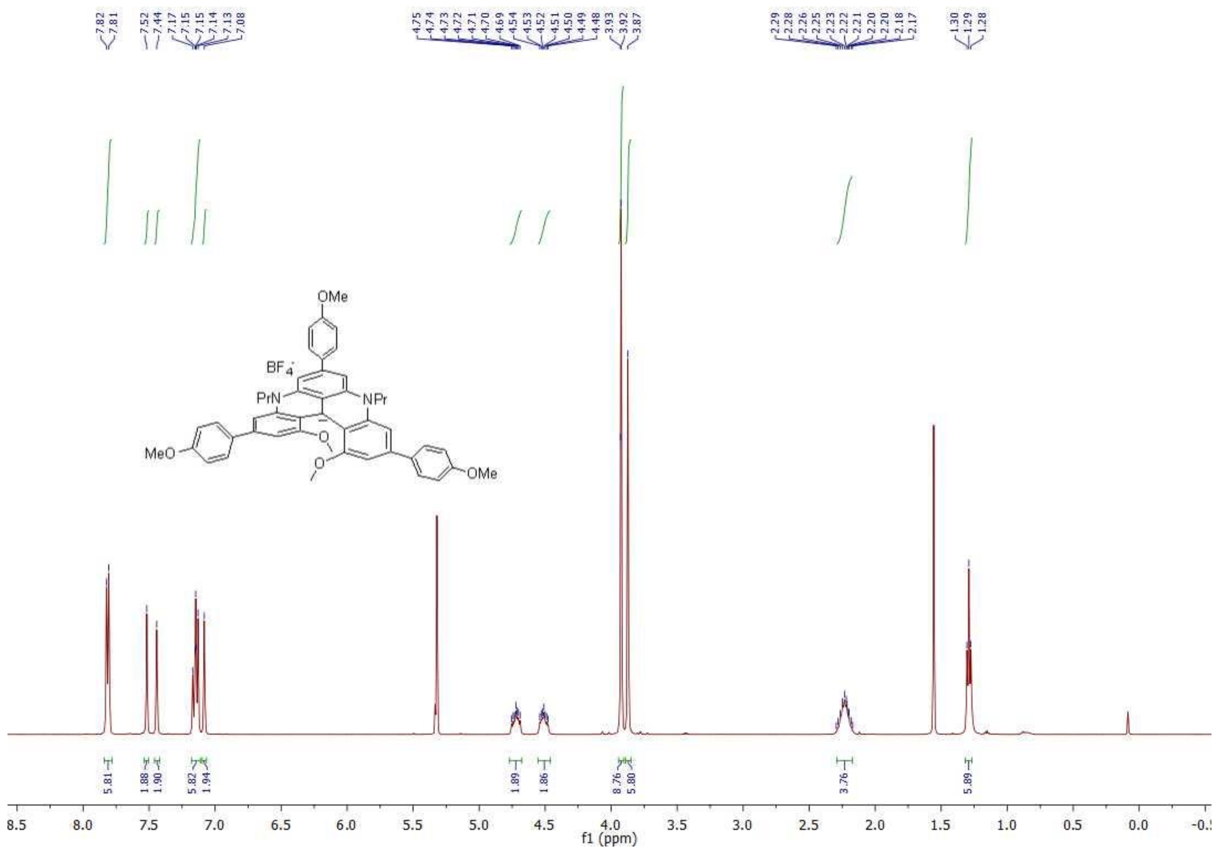

Figure S76. ^{1}H-NMR spectra of **6o** in CD_2Cl_2, 500 MHz.

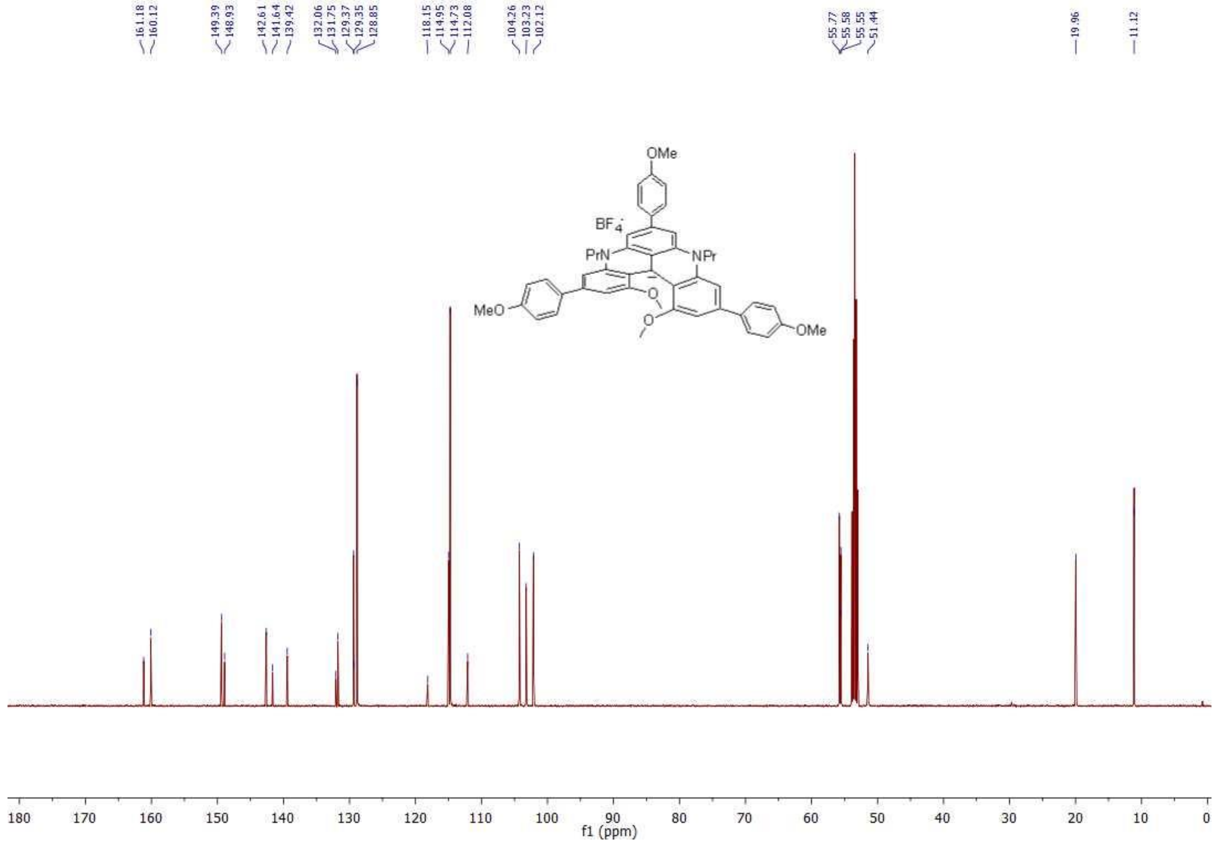

Figure S77. ^{13}C-NMR spectra of **6o** in CD_2Cl_2, 125 MHz.

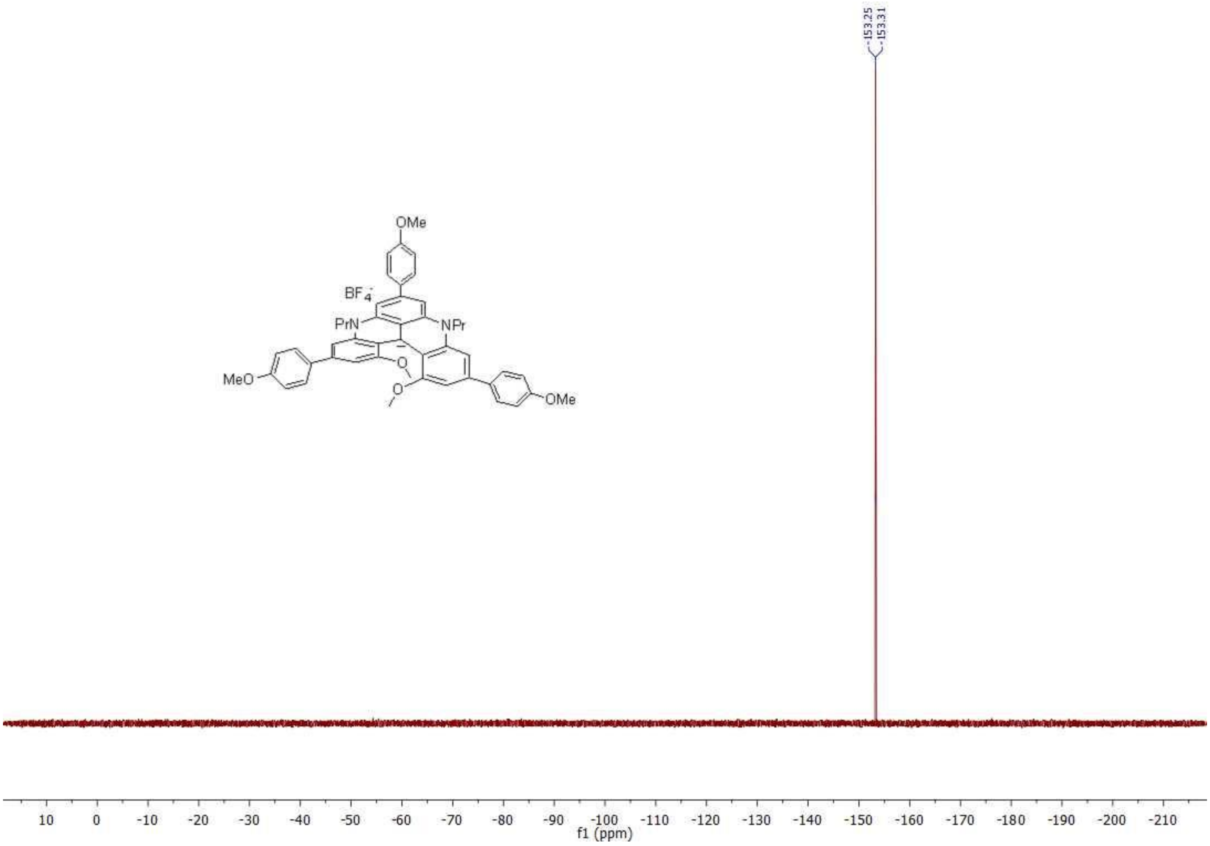

Figure S78. ^{19}F-NMR spectra of **6o** in CD_2Cl_2, 282 MHz.

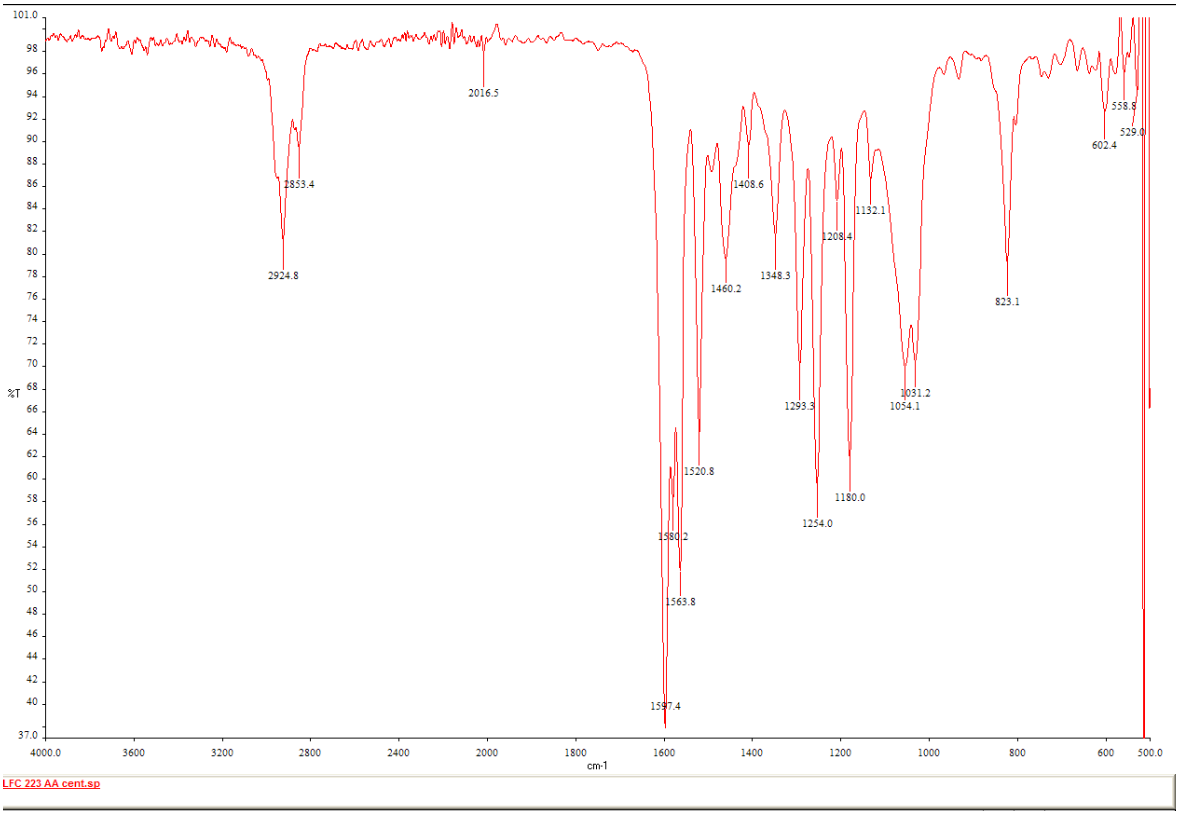

Figure S79. IR spectrum (neat) of **6o**.

Mass Spectrometry Core Facility

Lacour Group – University of Geneva

UNIVERSITÉ DE GENÈVE
FACULTÉ DES SCIENCES

ESI-HRMS – Certificate of Analysis

Applicant:	Lucas Frederic	Date of certificate:	06/09/21
Sample name:	LFC-224	Instrument:	Xevo G2 Tof (TOF)
Folder:	060921.PRO	Mobile phase:	MeOH (100 µl/min)
Analyst:	Stéphane Grass	Ionisation mode:	ESI (positive polarity)

Elemental Formula	Ion type	*Masslynx values* ***		Calc. *m/z*	Meas. *m/z*	Accuracy [a] *(ppm)*
		calc. m/z	*meas. m/z*			
$C_{48}H_{47}N_2O_5$	$[M]^+$	*731.3485*	*731.3465*	731.3480	731.3460	-2.7

[a] *Mass spectrum is calibrated by the use of the MS lockspray system (LeuEnk calibration solution).*

**** MassLynx software does not take into account the mass of the electron for ionic species, therefore the shift of m/z 0.000459.*

Zoomed mass spectrum – Isotopic distribution.

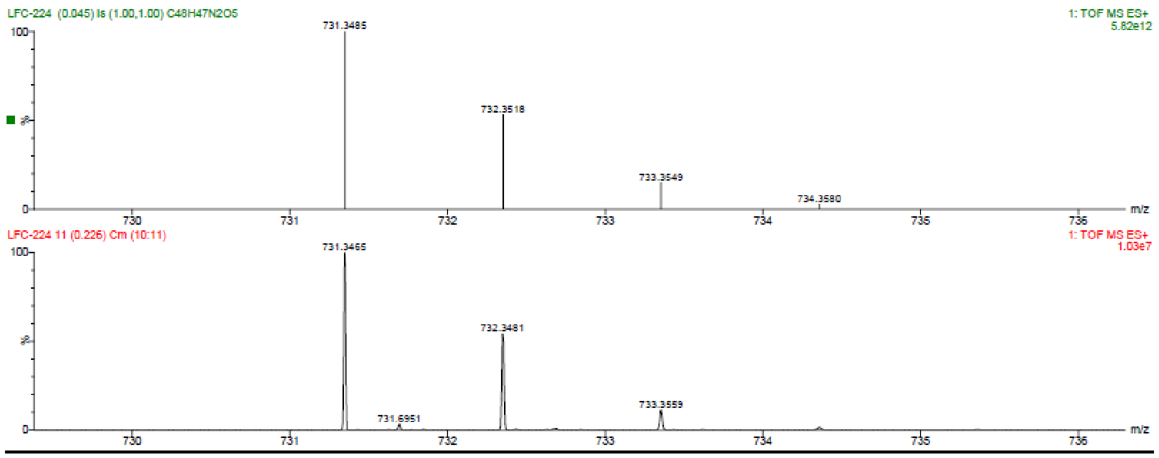

Figure S80. HRMS analysis (ESI, MeOH) report of **6o**.

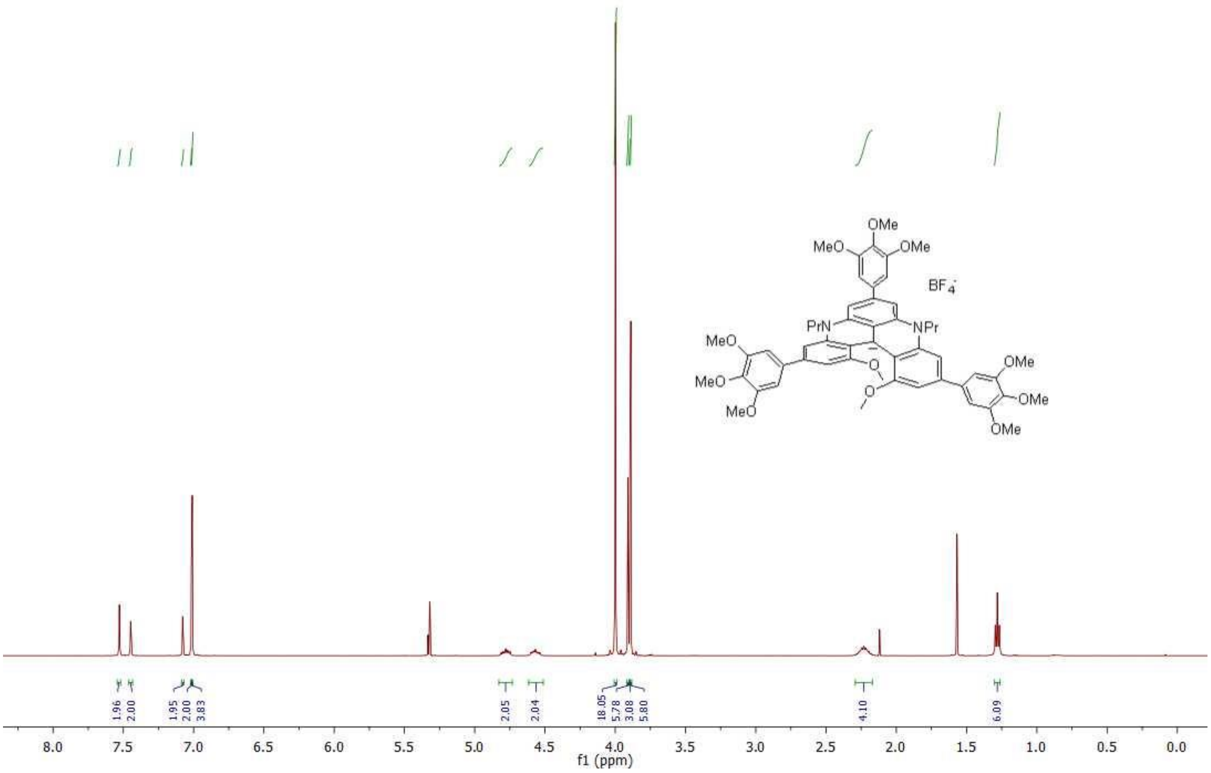

Figure S81. ^{1}H-NMR spectra of **6p** in CD_2Cl_2, 500 MHz.

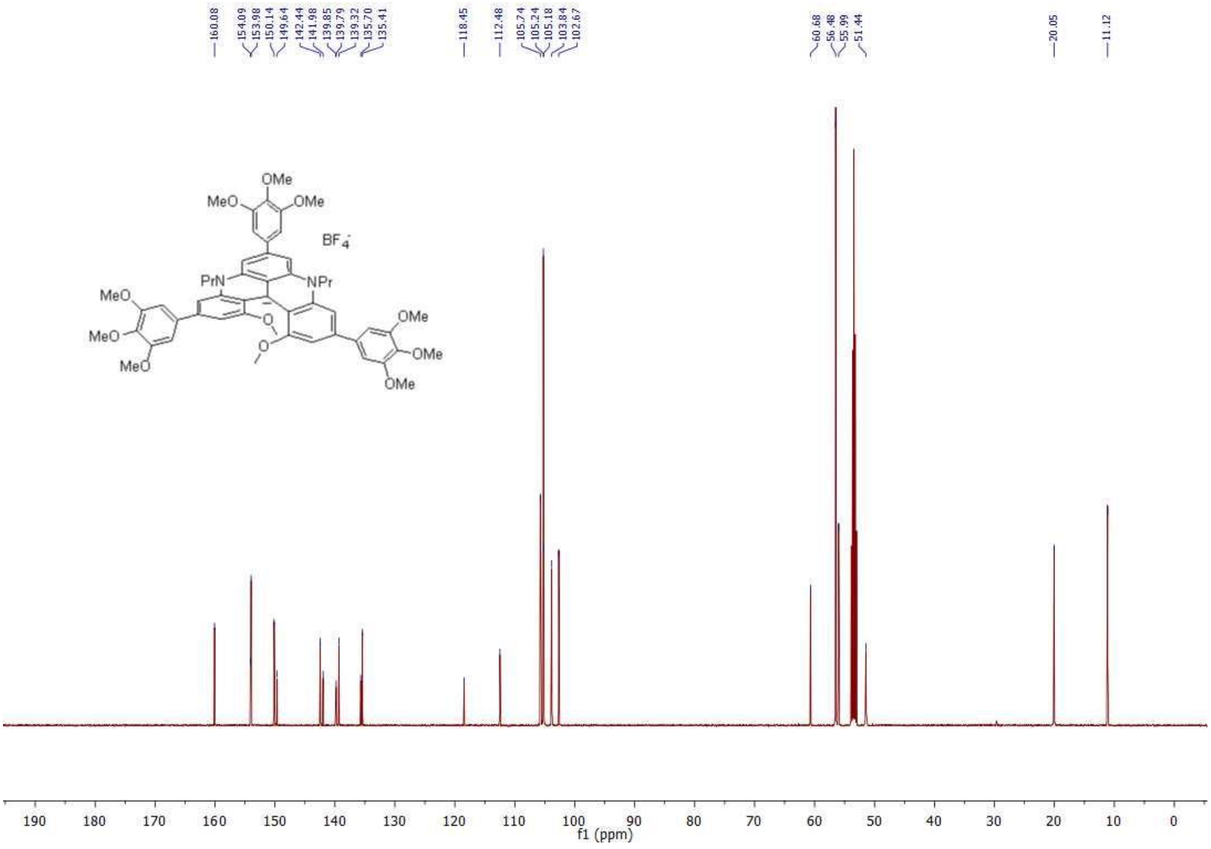

Figure S82. ^{13}C-NMR spectra of **6p** in CD_2Cl_2, 125 MHz.

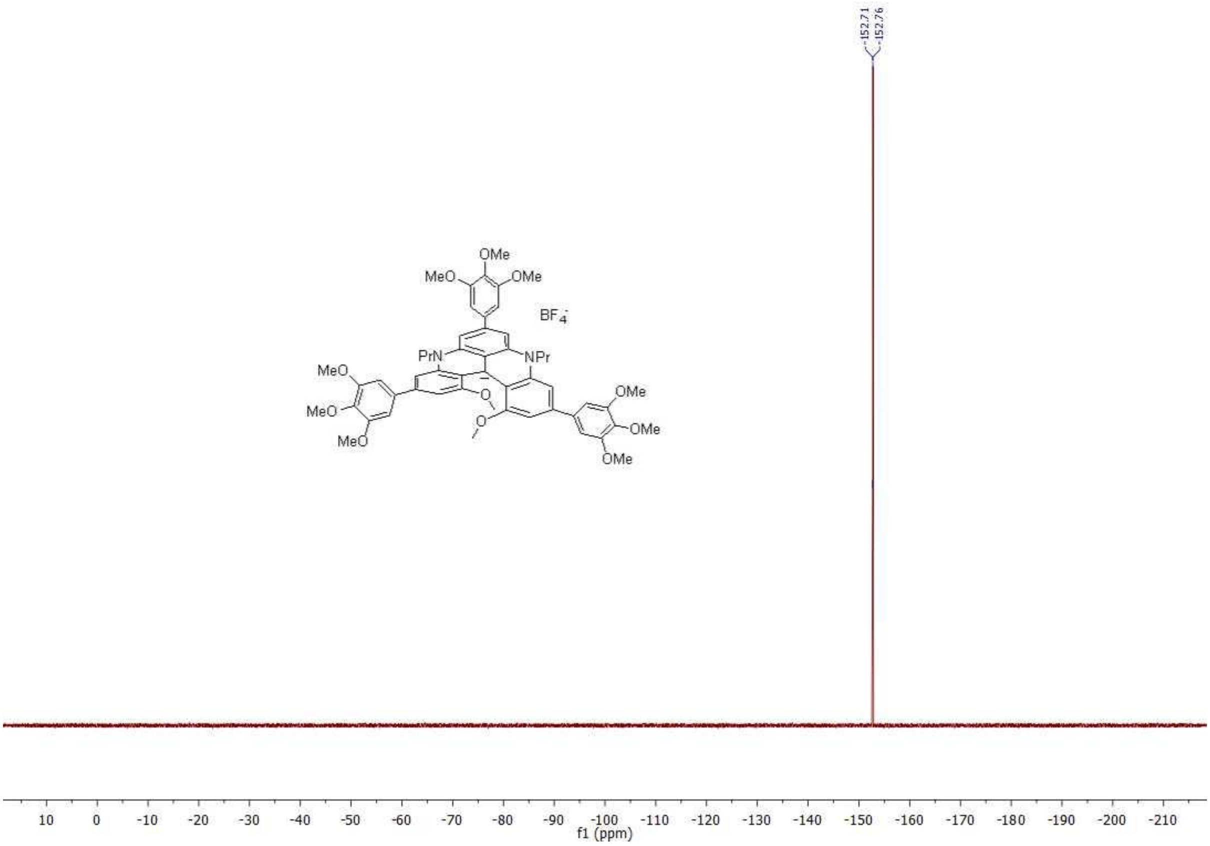

Figure S83. ^{19}F-NMR spectra of **6p** in CD_2Cl_2, 282 MHz.

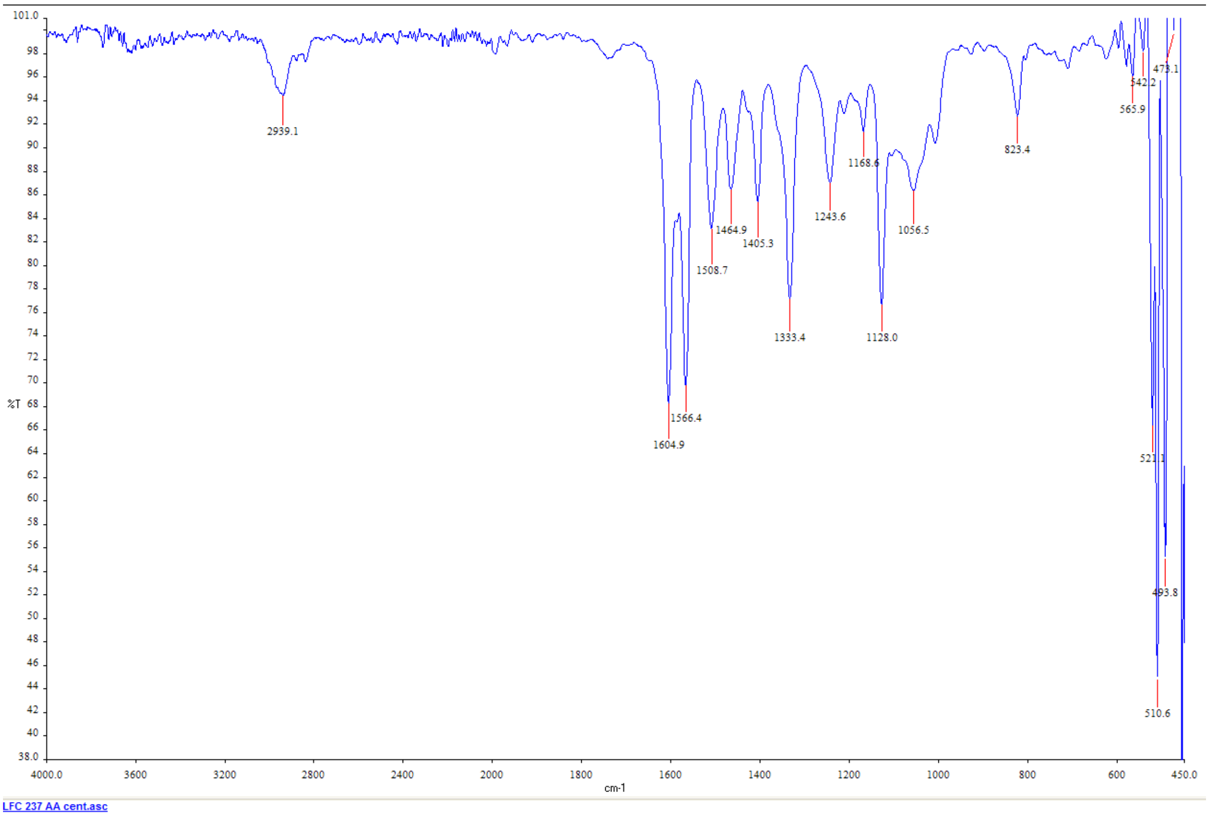

Figure S84. IR spectrum (neat) of **6p**.

Mass Spectrometry Core Facility

Lacour Group – University of Geneva

UNIVERSITÉ DE GENÈVE
FACULTÉ DES SCIENCES

ESI-HRMS – Certificate of Analysis

Applicant:	Lucas Frederic	Date of certificate:	06/09/21
Sample name:	LFC-234	Instrument:	Xevo G2 Tof (TOF)
Folder:	060921.PRO	Mobile phase:	MeOH (100 µl/min)
Analyst:	Stéphane Grass	Ionisation mode:	ESI (positive polarity)

Elemental Formula	Ion type	Masslynx values *** calc. m/z	meas. m/z	Calc. *m/z*	Meas. *m/z*	Accuracy [a] (ppm)
$C_{54}H_{59}N_2O_{11}$	$[M]^+$	1136.4904	1136.4900	1,136.4899	1,136.4895	-0.4

[a] *Mass spectrum is calibrated by the use of the MS lockspray system (LeuEnk calibration solution).*

**** MassLynx software does not take into account the mass of the electron for ionic species, therefore the shift of m/z 0.000459.*

Zoomed mass spectrum – Isotopic distribution.

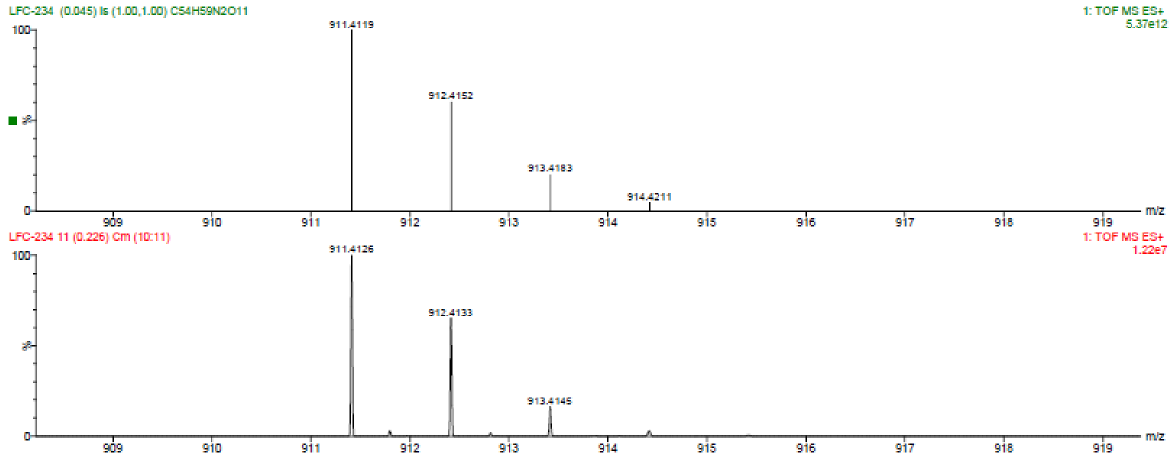

Figure S85. HRMS analysis (ESI, MeOH) report of **6p**.

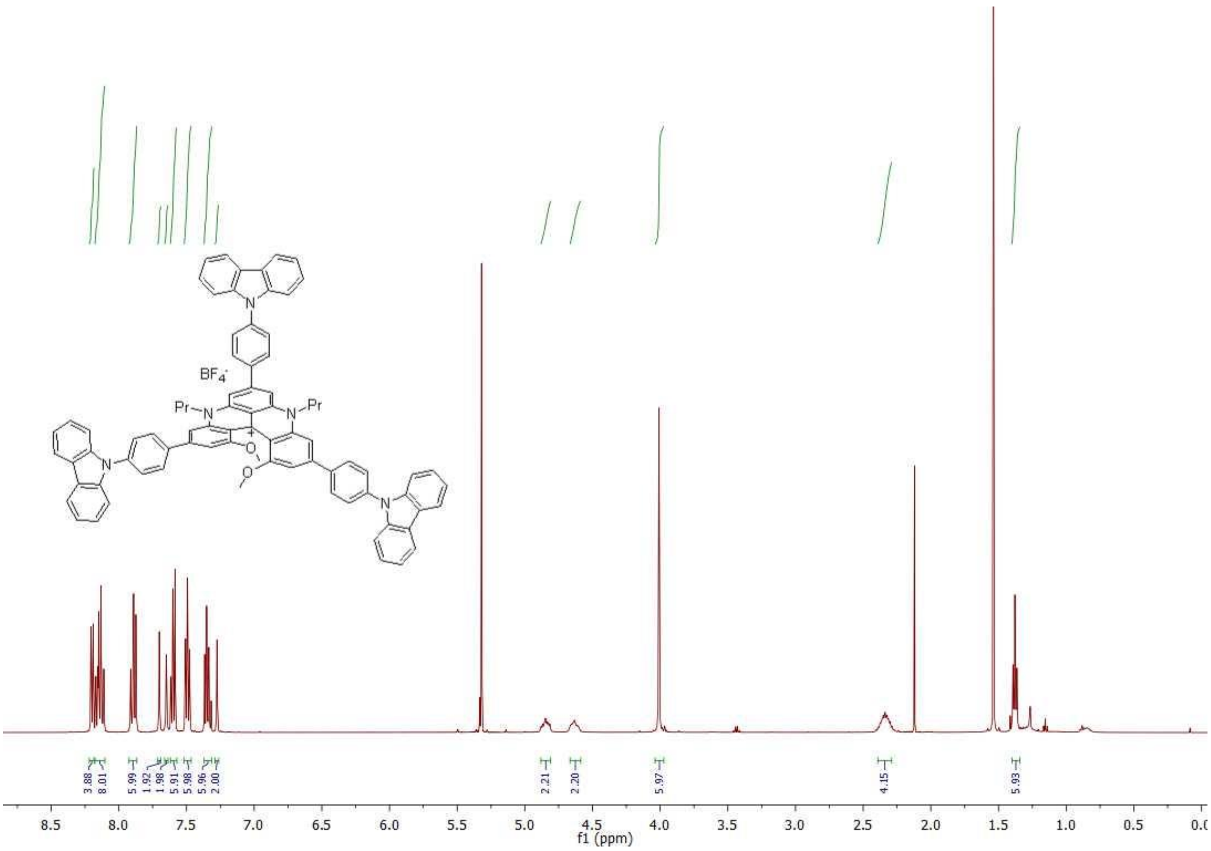

Figure S86. ^{1}H-NMR spectra of **6q** in CD_2Cl_2, 500 MHz.

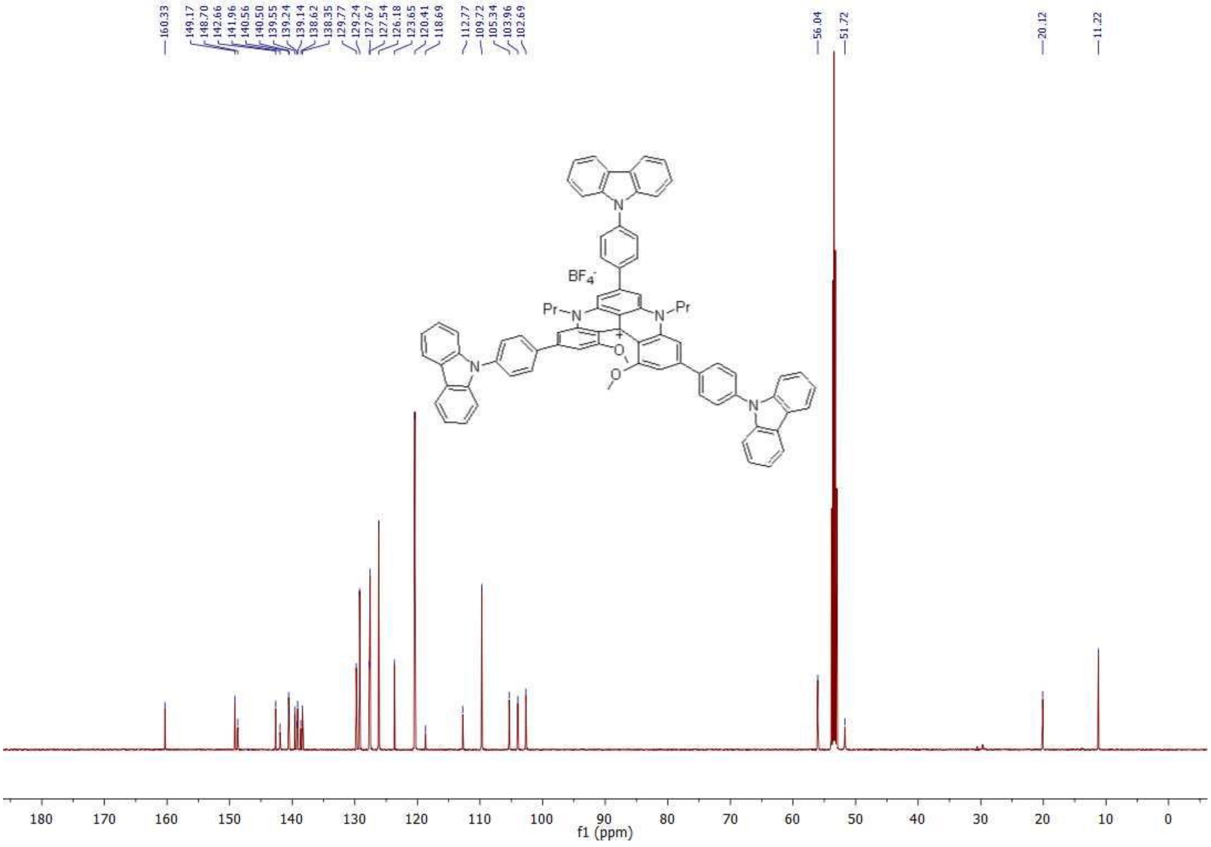

Figure S87. ^{13}C-NMR spectra of **6q** in CD_2Cl_2, 125 MHz.

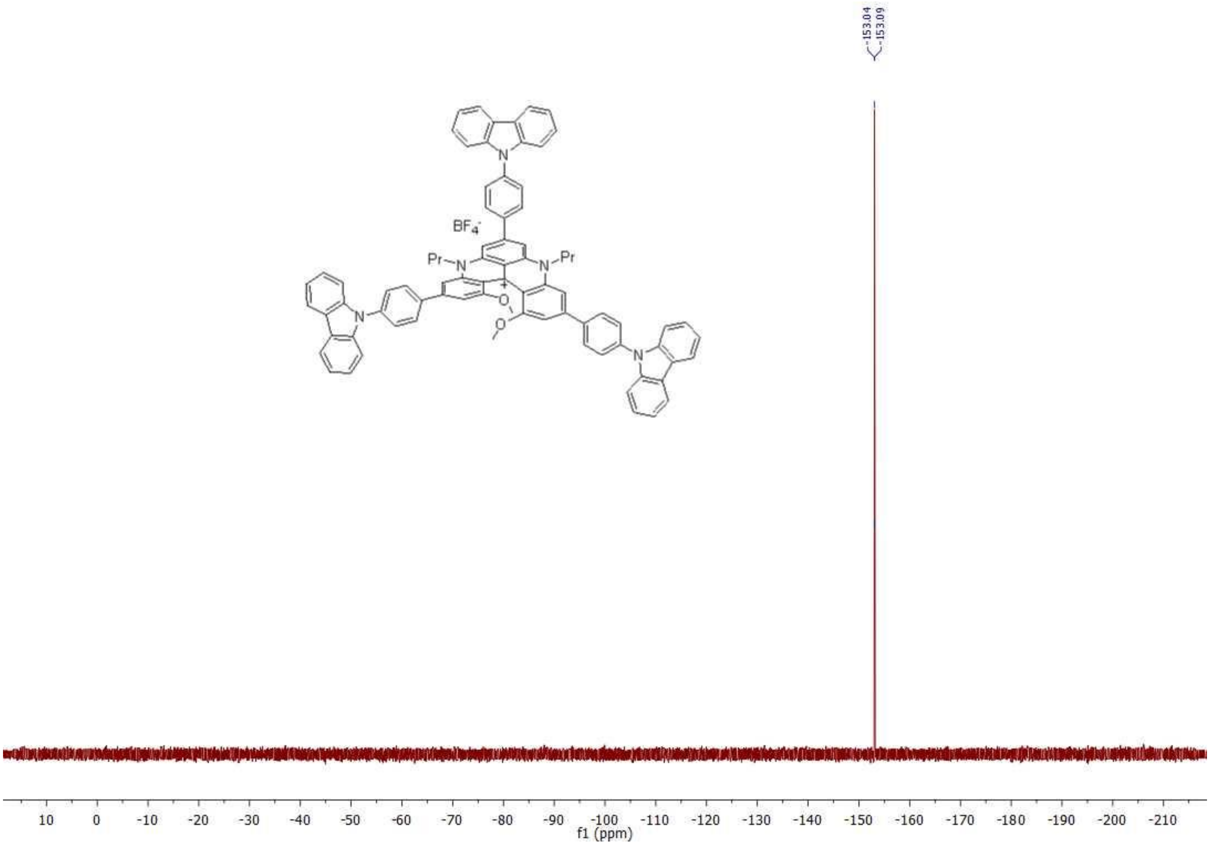

Figure S88. ^{19}F-NMR spectra of **6q** in CD_2Cl_2, 282 MHz.

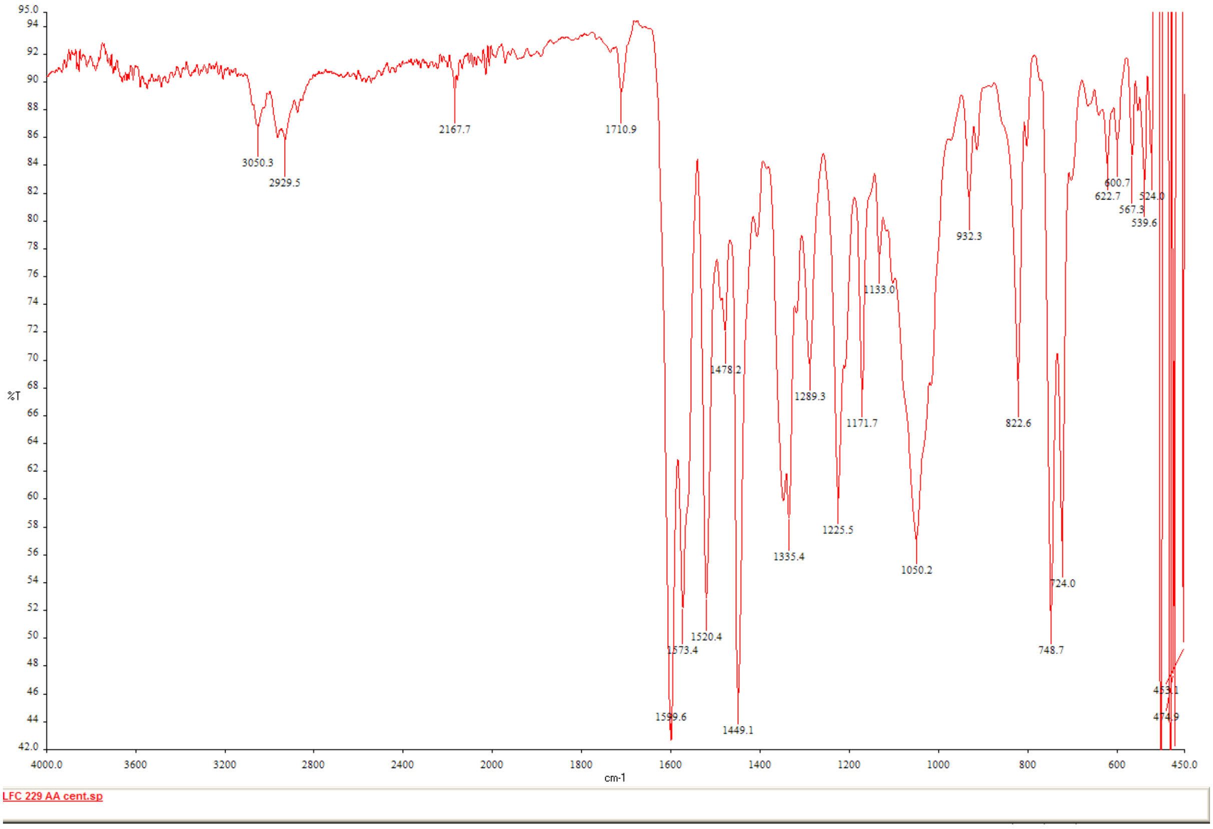

Figure S89. IR spectrum (neat) of **6q**.

Mass Spectrometry Core Facility

Lacour Group – University of Geneva

UNIVERSITÉ DE GENÈVE
FACULTÉ DES SCIENCES

ESI-HRMS – Certificate of Analysis

Applicant:	Lucas Frederic	Date of certificate:	06/09/21
Sample name:	LFC-229	Instrument:	Xevo G2 Tof (TOF)
Folder:	060921.PRO	Mobile phase:	MeOH (100 µl/min)
Analyst:	Stéphane Grass	Ionisation mode:	ESI (positive polarity)

Elemental Formula	Ion type	Masslynx values *** calc. *m/z*	Masslynx values *** meas. *m/z*	Calc. *m/z*	Meas. *m/z*	Accuracy [a] (ppm)
$C_{81}H_{62}N_5O_2$	$[M]^+$	1136.4904	1136.4900	1,136.4899	1,136.4895	-0.4

[a] *Mass spectrum is calibrated by the use of the MS lockspray system (LeuEnk calibration solution).*

**** MassLynx software does not take into account the mass of the electron for ionic species, therefore the shift of m/z 0.000459.*

Zoomed mass spectrum – Isotopic distribution.

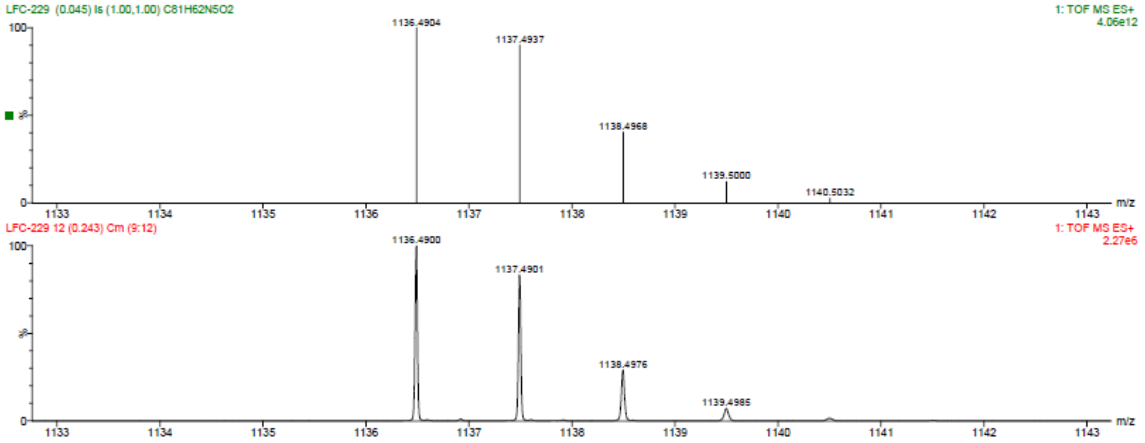

Figure S90. HRMS analysis (ESI, MeOH) report of **6q**.

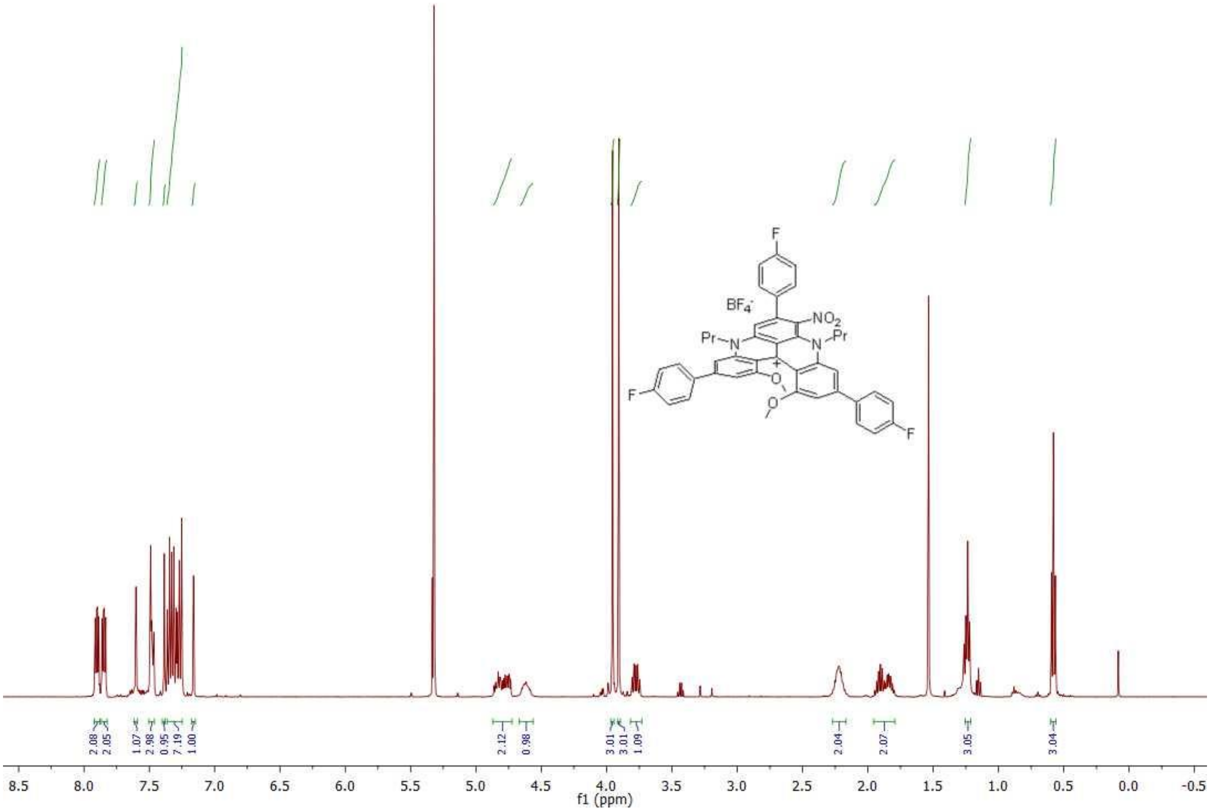

Figure S91. ^{1}H-NMR spectra of **9** in CD_2Cl_2, 500 MHz.

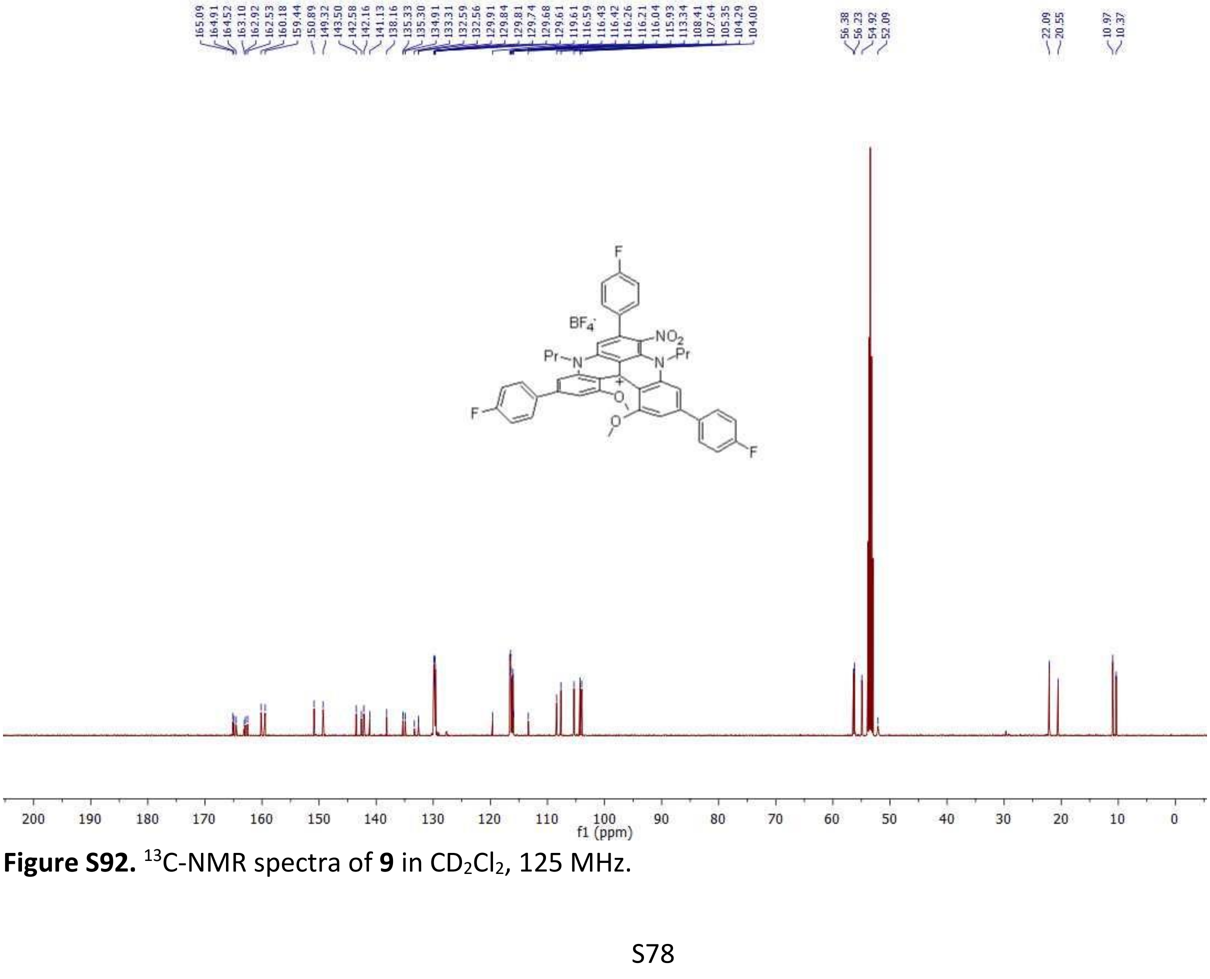

Figure S92. ^{13}C-NMR spectra of **9** in CD_2Cl_2, 125 MHz.

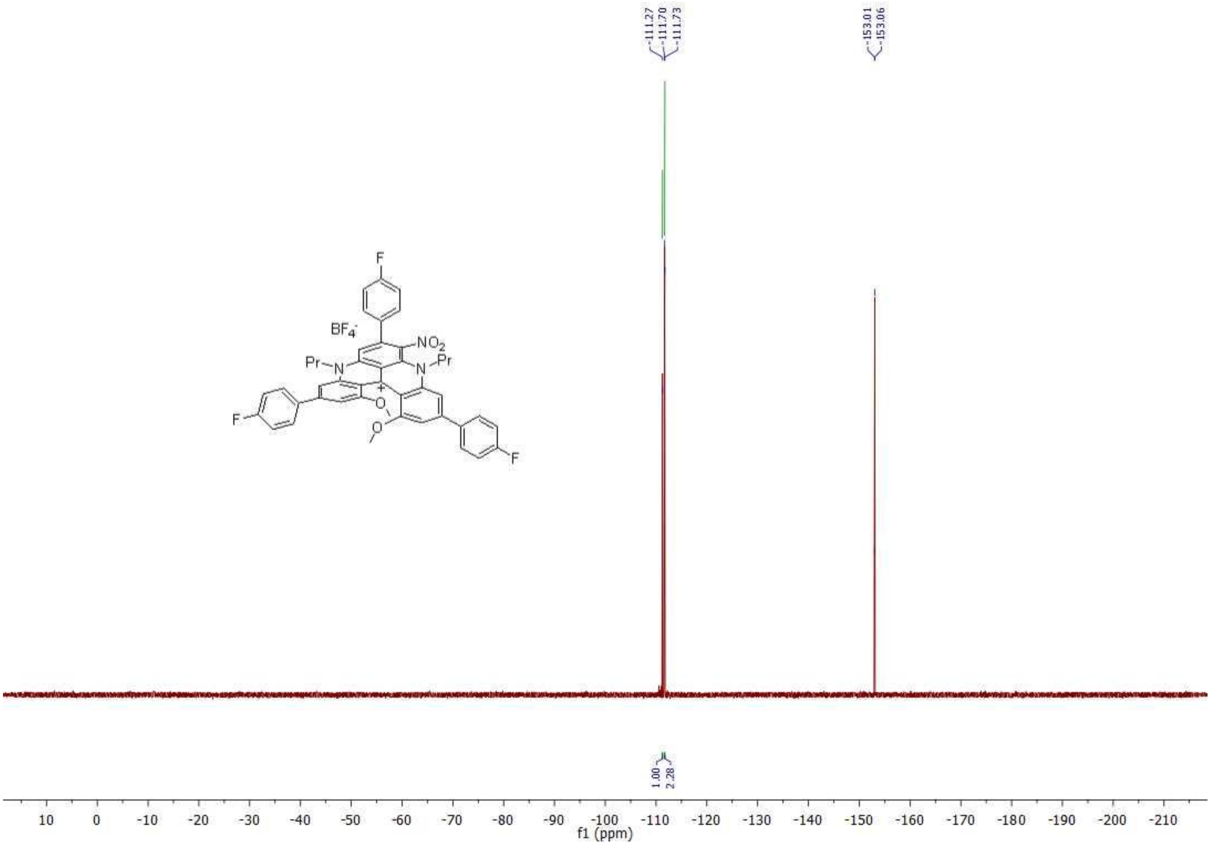

Figure S93. ^{19}F-NMR spectra of **9** in CD_2Cl_2, 282 MHz.

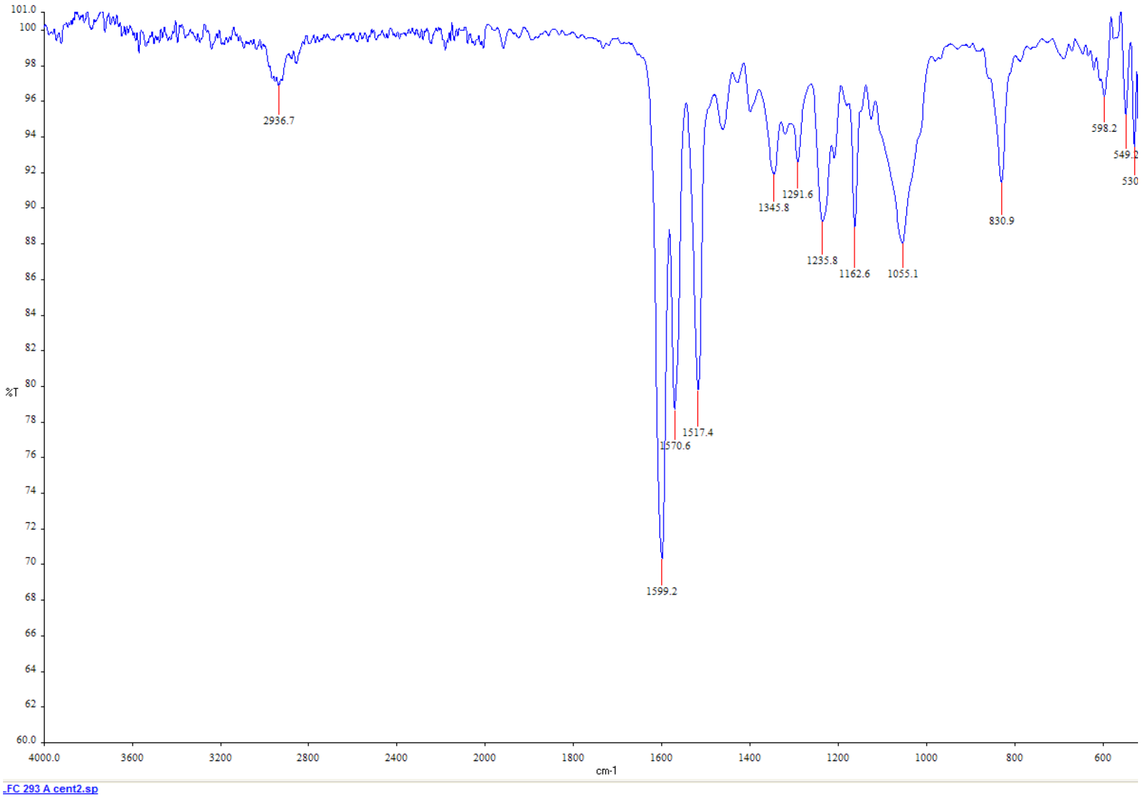

Figure S94. IR spectrum (neat) of **9**.

Mass Spectrometry Core Facility

Lacour Group – University of Geneva

UNIVERSITÉ DE GENÈVE
FACULTÉ DES SCIENCES

ESI-HRMS – Certificate of Analysis

Applicant:	Lucas Frederic	Date of certificate:	03/12/21
Sample name:	LFC-293	Instrument:	Xevo G2 Tof (TOF)
Folder:	031221.PRO	Mobile phase:	MeOH (100 µl/min)
Analyst:	Stéphane Grass	Ionisation mode:	ESI (positive polarity)

Elemental Formula	Ion type	*Masslynx values **** calc. m/z	meas. m/z	Calc. *m/z*	Meas. *m/z*	Accuracy [a] (ppm)
$C_{45}H_{37}F_3N_3O_4$	$[M]^+$	*740.2736*	*740.2742*	740.2731	740.2737	0.8

[a] *Mass spectrum is calibrated by the use of the MS lockspray system (LeuEnk calibration solution).*

**** MassLynx software does not take into account the mass of the electron for ionic species, therefore the shift of m/z 0.000459.*

Zoomed mass spectrum – Isotopic distribution.

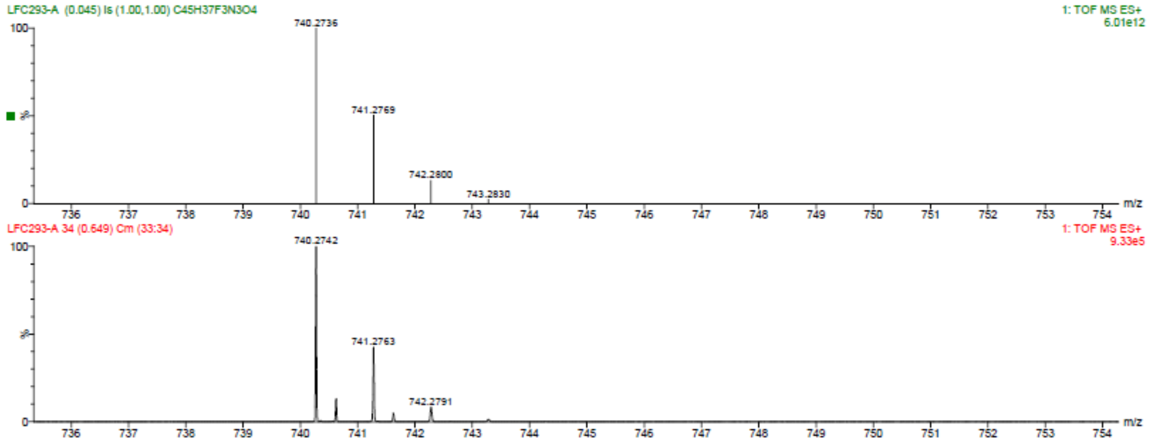

Figure S95. HRMS analysis (ESI, MeOH) report of **9**

Additional NMR and MS spectra for optimization or mechanistic studies

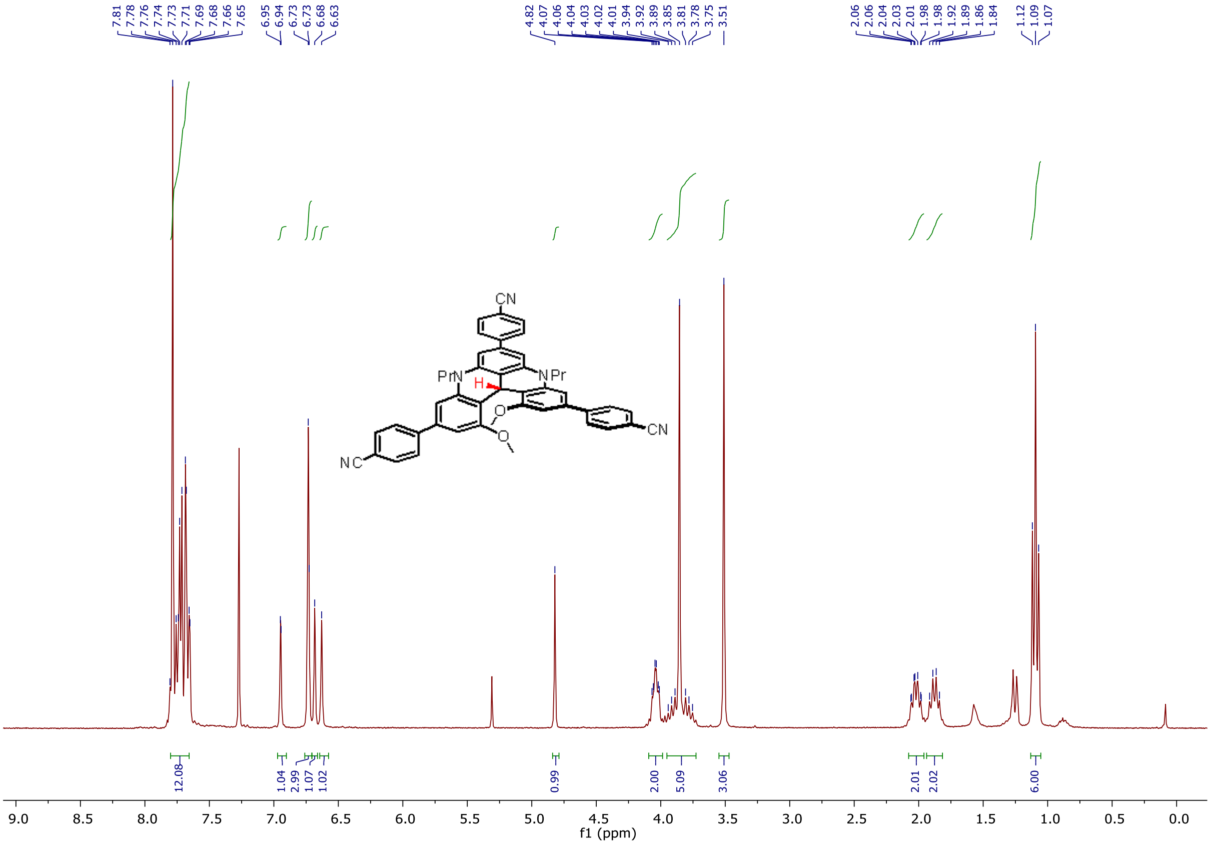

Figure S96. ^{1}H-NMR spectra of 6d-H in CDCl3, 300 MHz.

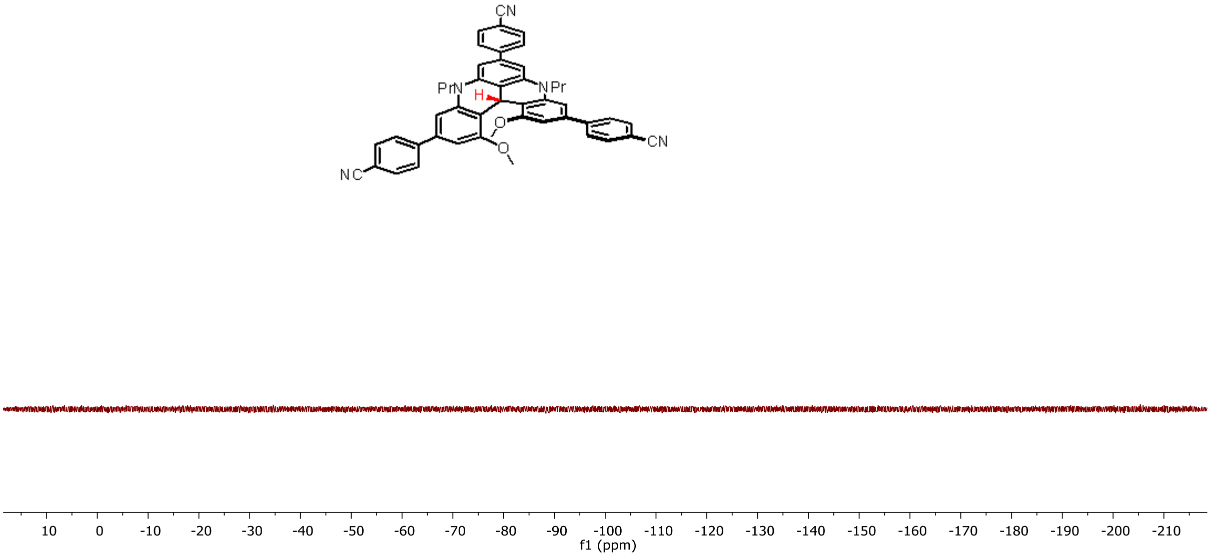

Figure S97. ^{19}F-NMR spectra of **6d**-H in $CDCl_3$, 282 MHz

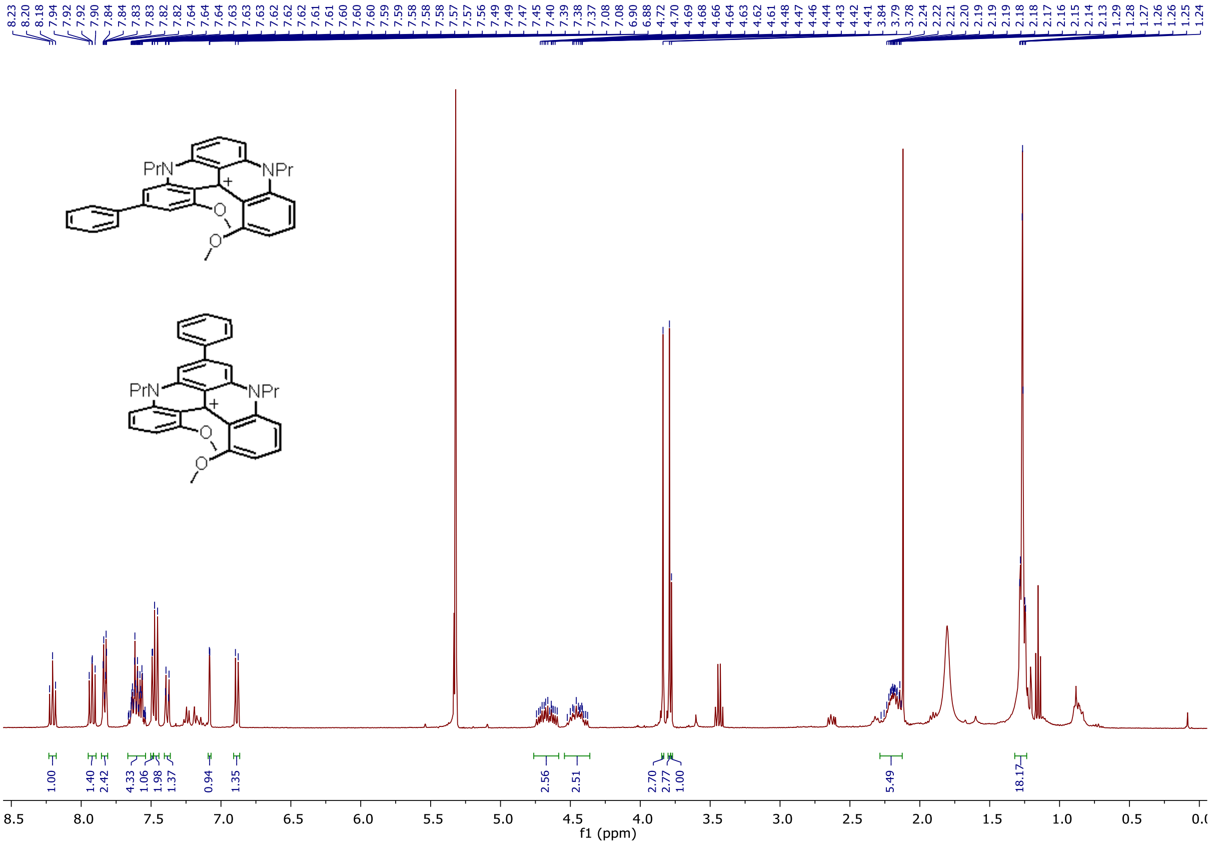

Figure S98. ^{1}H-NMR spectra of **7a** in CD_2Cl_2, 400 MHz, resulting from attempt of isolation.

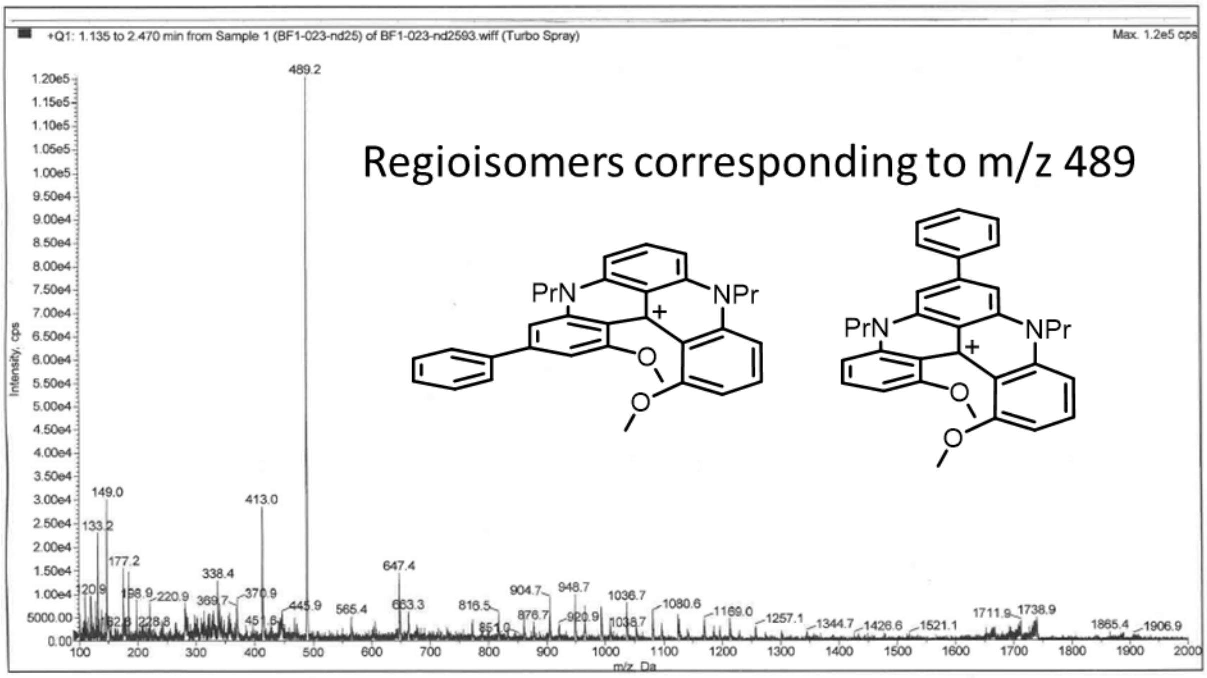

Figure S99. Low-resolution mass spectrometry spectrum corresponding to attempt of isolation of **7a**.

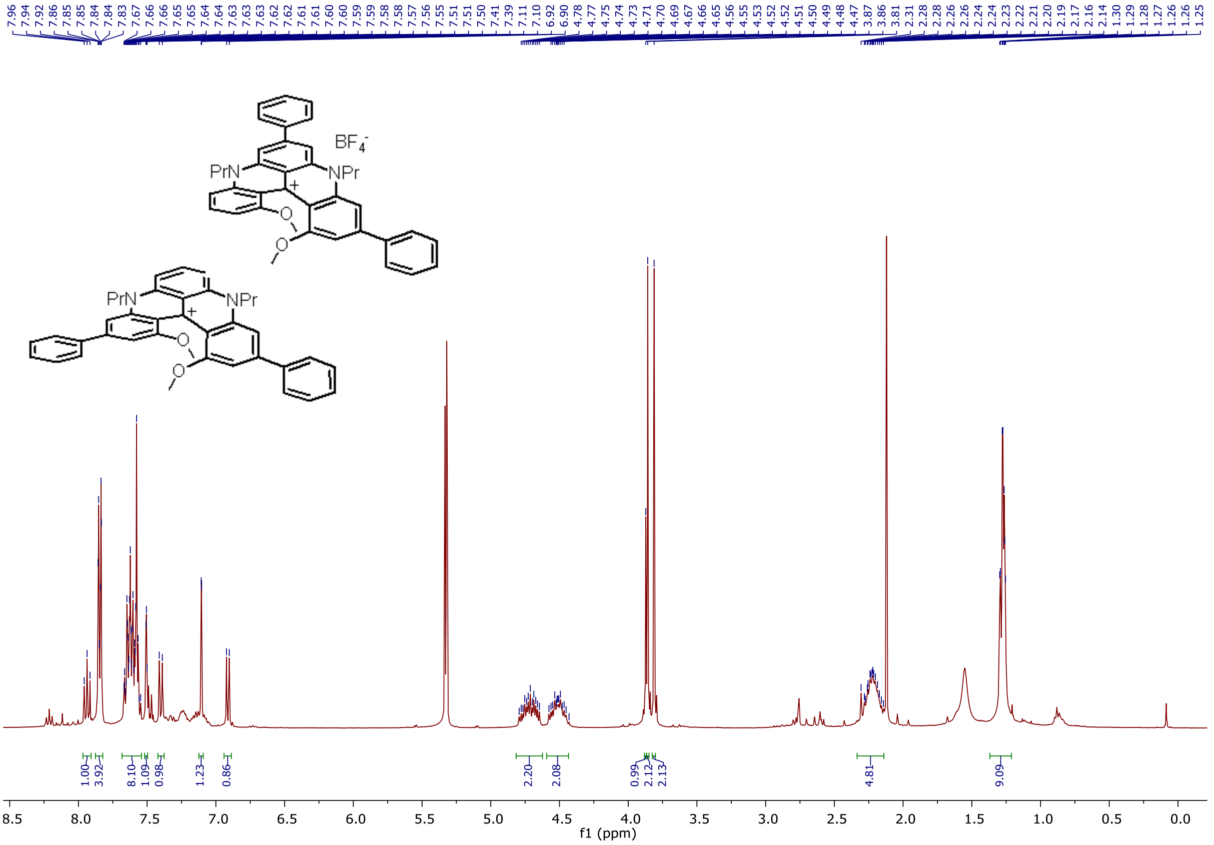

Figure S100. ^{1}H-NMR spectra of **8a** in CD_2Cl_2, 400 MHz, resulting from attempt of isolation.

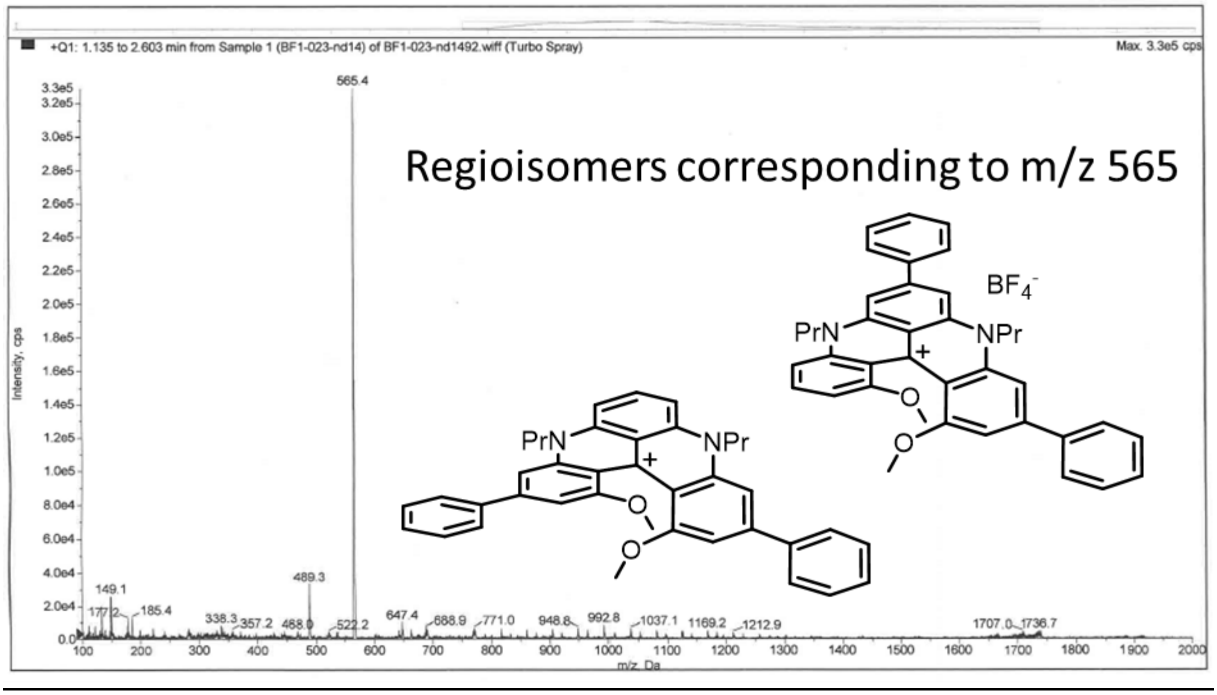

Figure S101. Low-resolution mass spectrometry spectrum corresponding to attempt of isolation of **8a**.

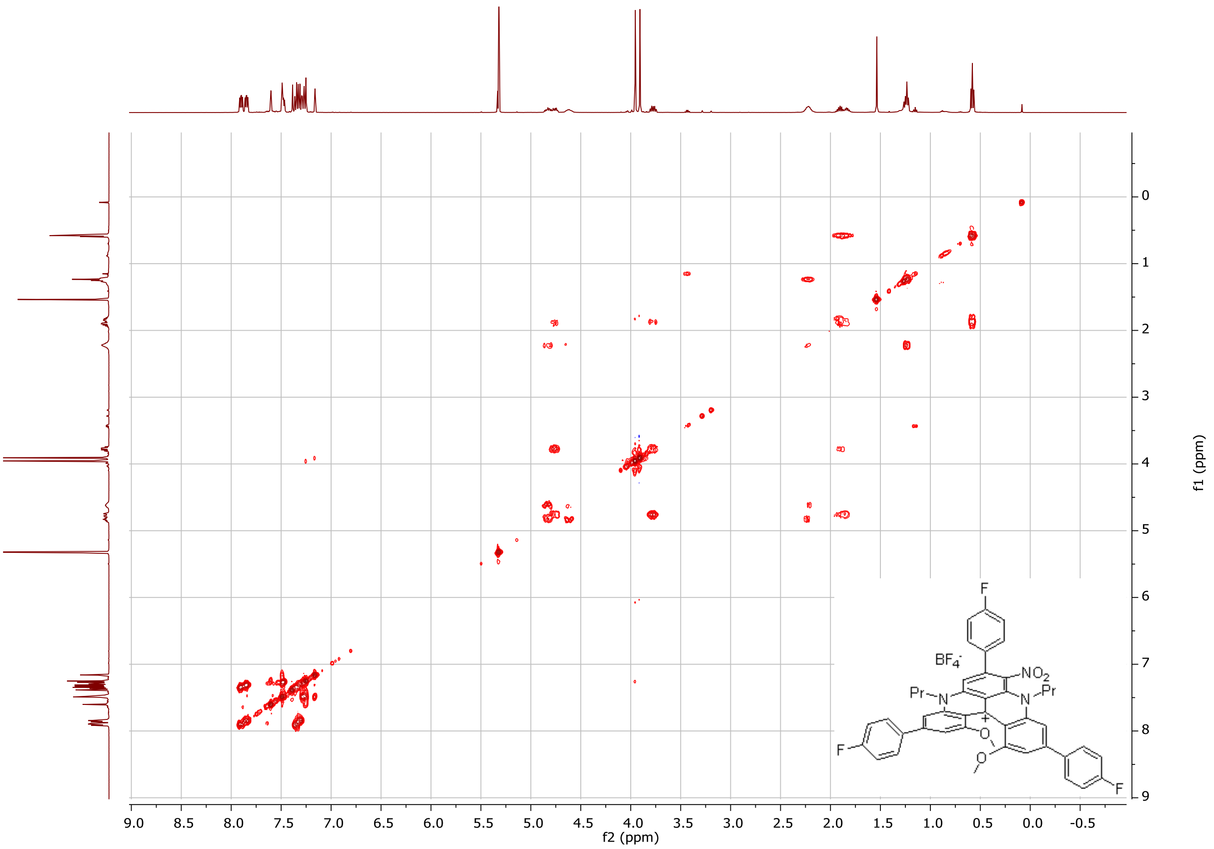

Figure S102. COSY (500 MHz, CD_2Cl_2) analysis of **9**.

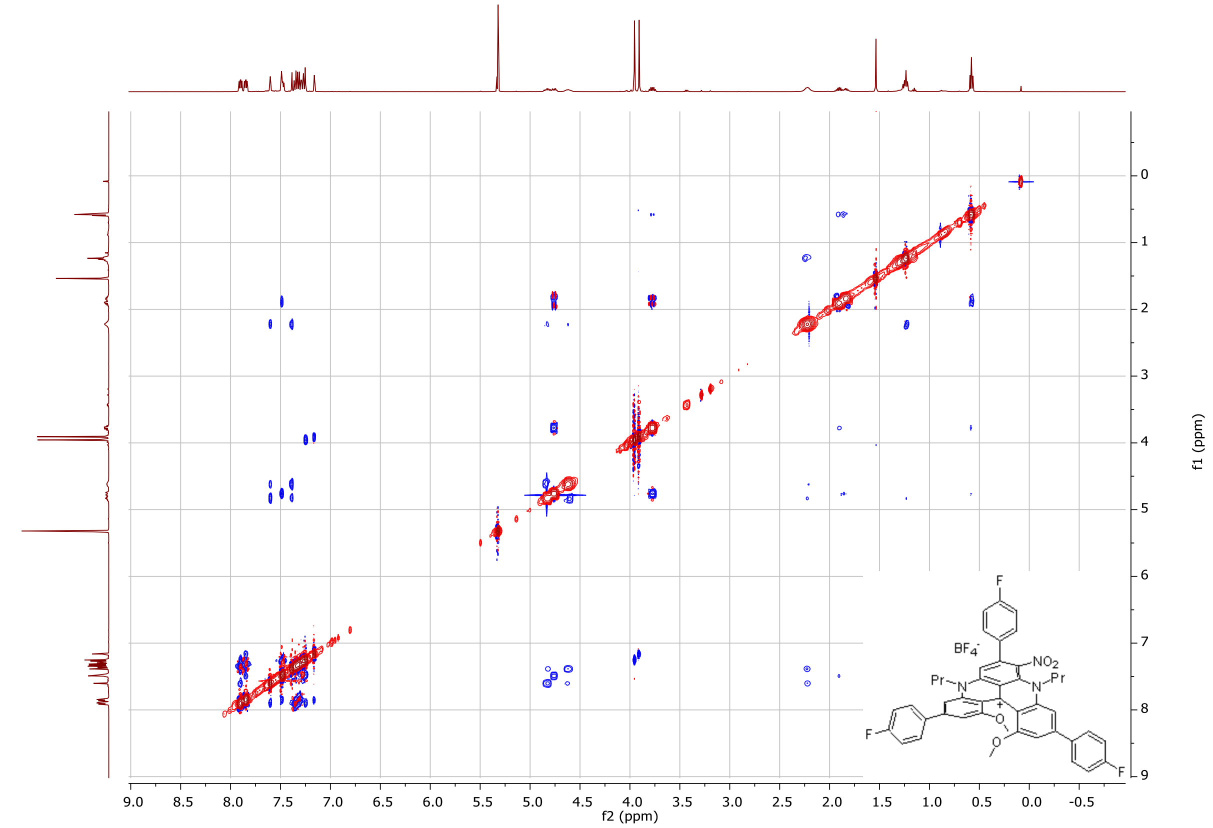

Figure S103. NOESY (500 MHz, CD_2Cl_2) analysis of **9**.

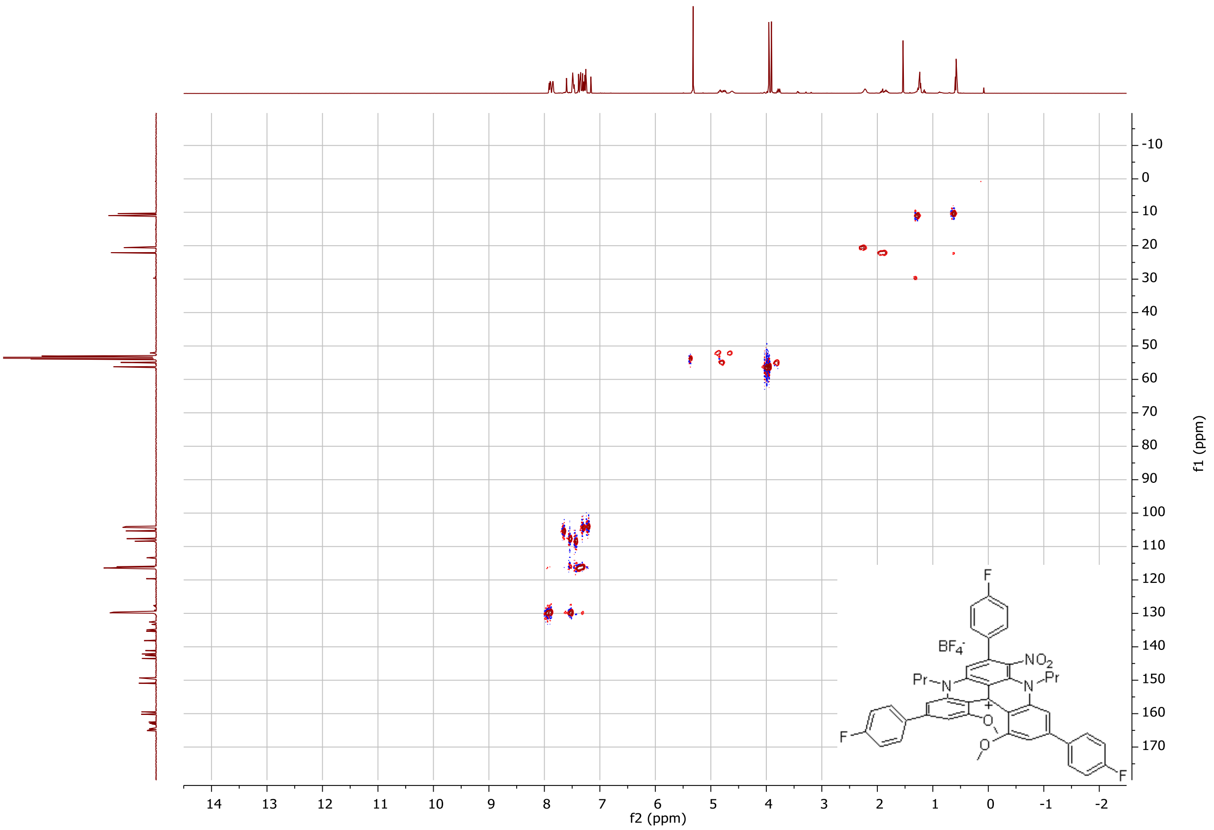

Figure S104. HSQC (^{1}H 500 MHz, ^{13}C 126 MHz, CD_2Cl_2) analysis of **9**.

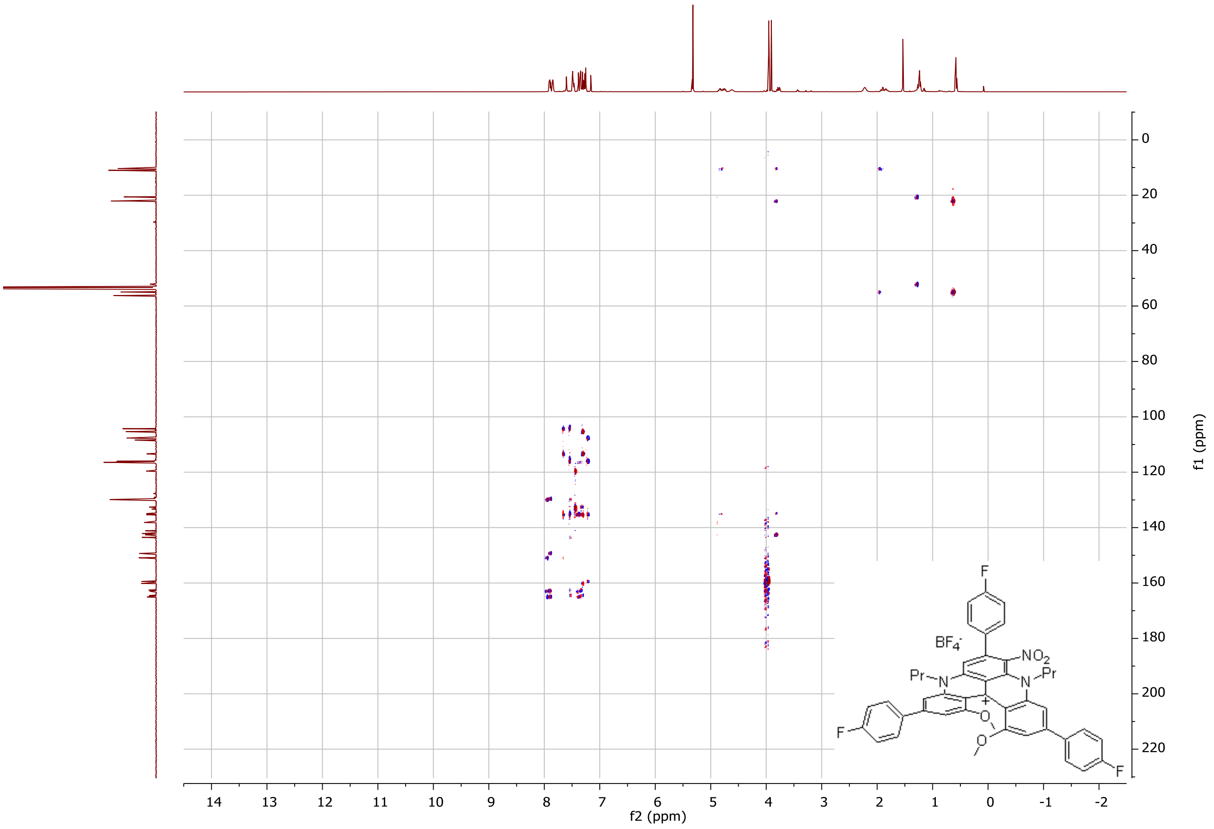

Figure S105. HMBC (^{1}H 500 MHz, ^{13}C 126 MHz, CD_2Cl_2) analysis of **9**.

Solid state analysis (X-Ray diffraction)

Table S1. Crystal data and structure refinement for **6e.**

CCDC number	2171649	
Empirical formula	C52 H49 B Cl2 F4 N2 O5	
Formula weight	939.64	
Temperature	150.01(10) K	
Wavelength	1.54184 Å	
Crystal system	Orthorhombic	
Space group	$P\ 2_12_12_1$	
Unit cell dimensions	a = 7.89434(10) Å	α = 90°.
	b = 24.1199(3) Å	β = 90°.
	c = 24.2806(3) Å	γ = 90°.
Volume	4623.30(10) $Å^3$	
Z	4	
Density (calculated)	1.350 Mg/m^3	
Absorption coefficient	1.824 mm^{-1}	
F(000)	1960	
Crystal size	0.33 x 0.05 x 0.03 mm^3	
Theta range for data collection	2.582 to 68.994°.	
Index ranges	$-6\leq h \leq 9$, $-27\leq k \leq 28$, $-28\leq l \leq 29$	
Reflections collected	19287	
Independent reflections	8416 [R(int) = 0.0292]	
Completeness to theta = 67.684°	99.8 %	
Absorption correction	Analytical	
Max. and min. transmission	0.948 and 0.731	
Refinement method	Full-matrix least-squares on F^2	
Data / restraints / parameters	8416 / 6 / 622	
Goodness-of-fit on F^2	1.055	
Final R indices [I>2sigma(I)]	R_1 = 0.0410, wR_2 = 0.1066	
R indices (all data)	R_1 = 0.0487, wR_2 = 0.1120	
Absolute structure parameter	0.00(2)	
Extinction coefficient	n/a	
Largest diff. peak and hole	0.528 and -0.404 e.$Å^{-3}$	

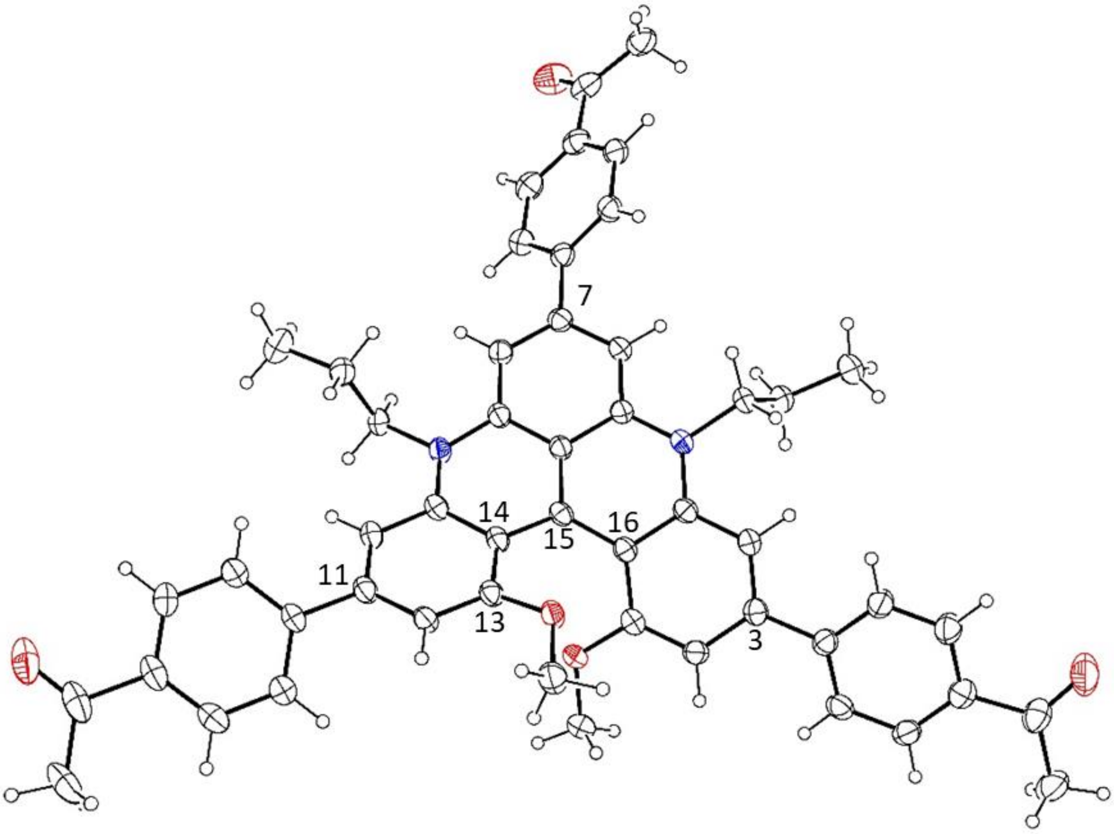

Figure S106. Ortep View of molecular **6e** (thermal ellipsoids are drawn at 50% probability level). BF_4 counter ion and CH_2Cl_2 dichloromethane solvent molecule are omitted for clarity purpose.

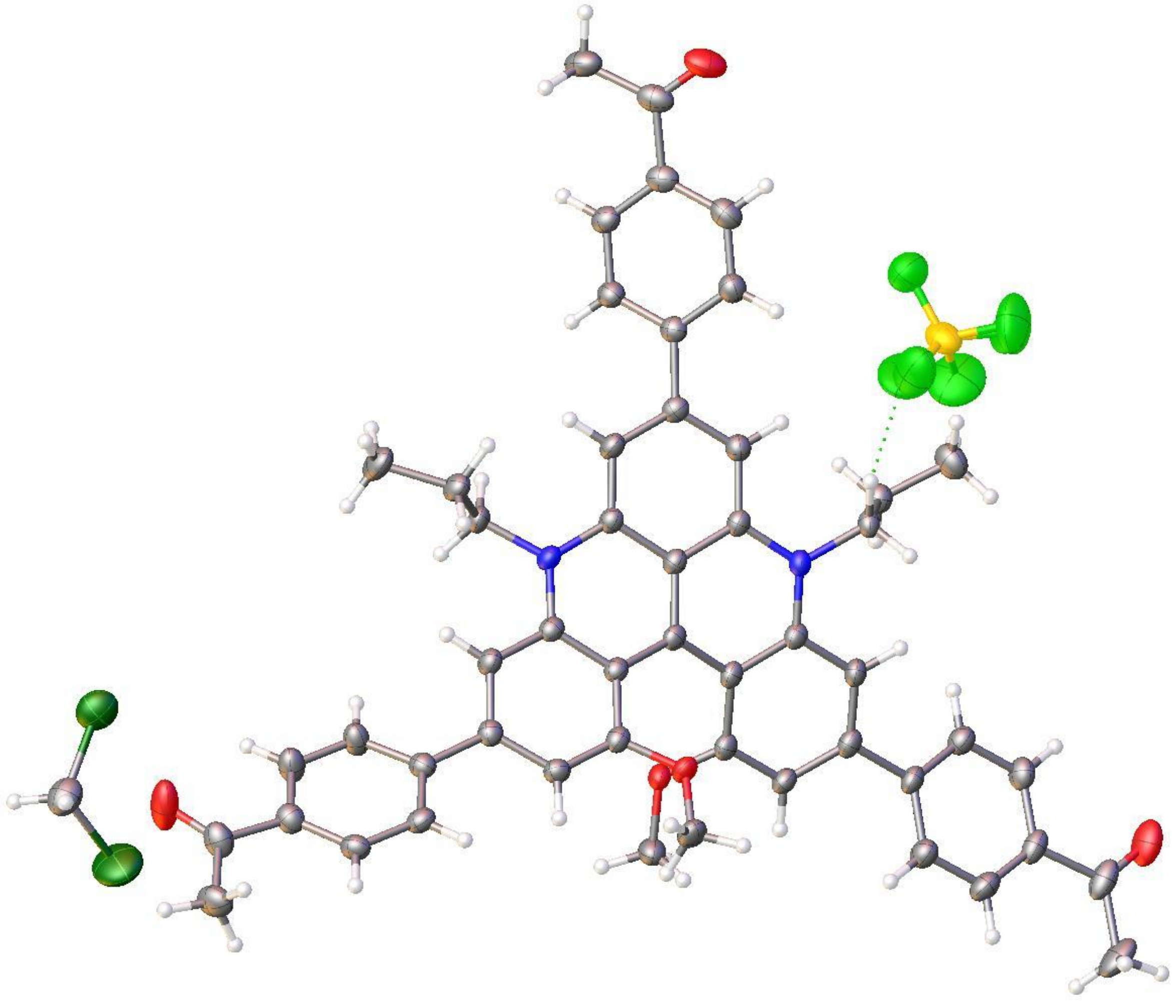

Figure S107. View of the asymmetric unit of **6e**

Remark: Counter ion BF4- is disordered and was refined by splitting 2 Fluorine atoms in 2 components (refined occupancies 0.54/0.46) and with restrained ADPs for F3A (ISOR).

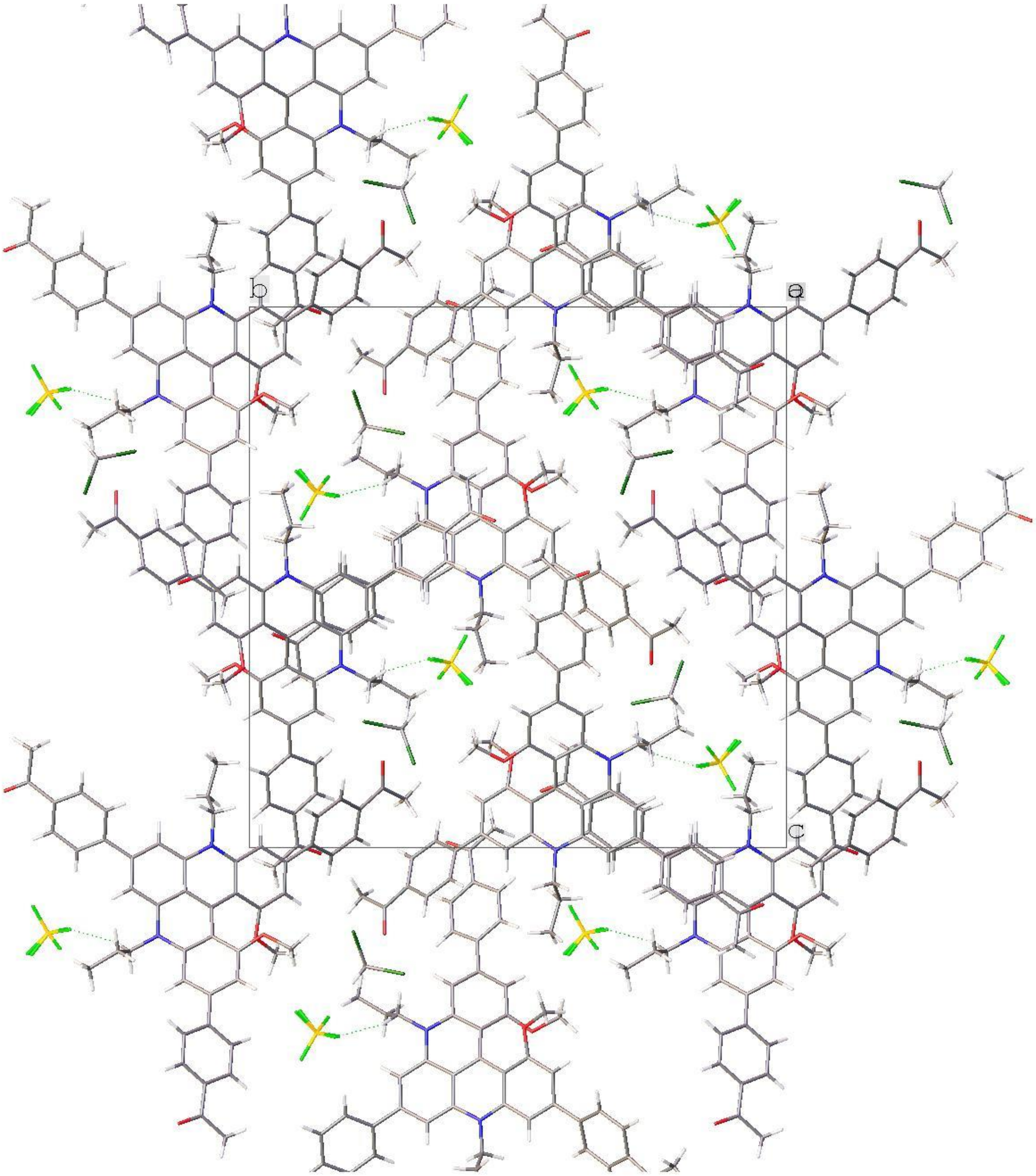

Figure S108. Packing of the molecules viewed along the *a* direction in **6e**.

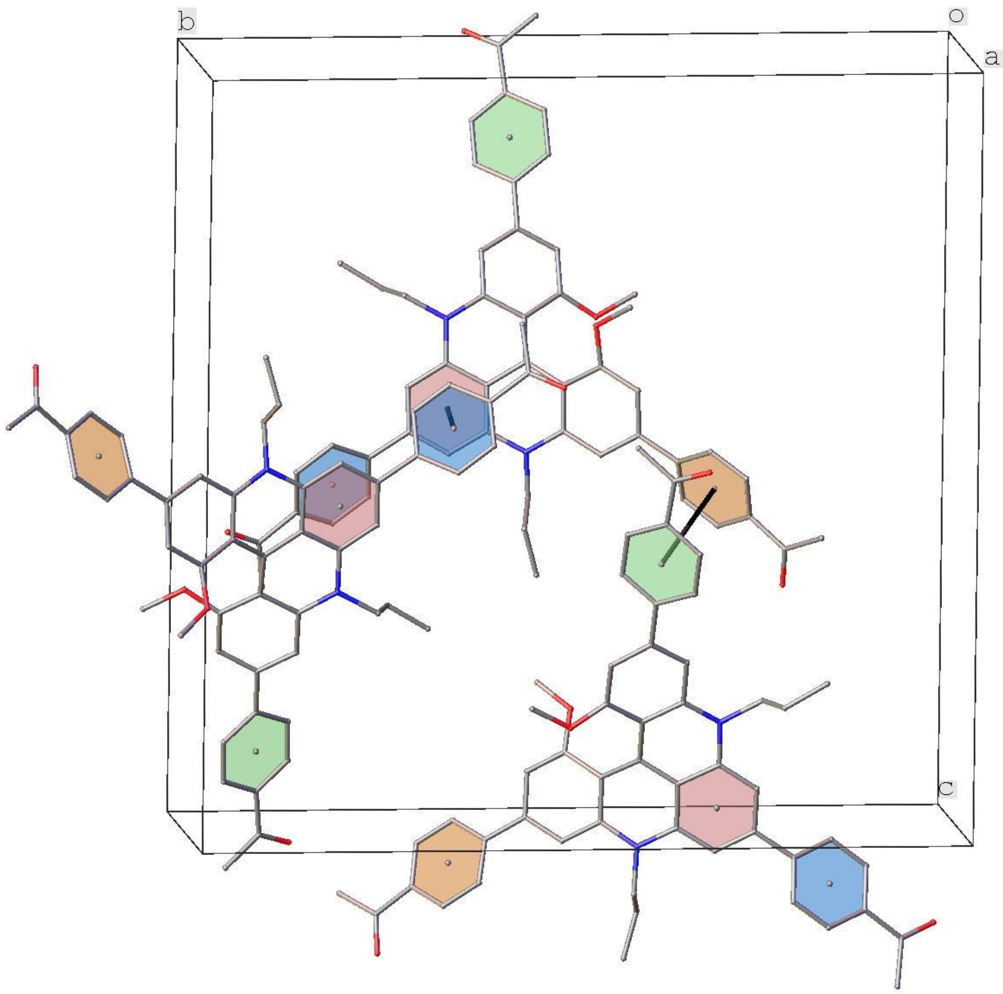

Figure S109. Detailed π-π stacking interactions found in **6e** (highlighted with black line). Aryl groups in green (position 3); orange (position 11) and blue (position 7) and ring of DMQA core (pink).

Table S2. Intermolecular interactions analysis in **6e**.

Planes	Label (color)	Sym op ‘	Sym op’’	
C3 C8 C7 C6 C5 C4	#1 (orange)			
C20 C33 C32 C31 C22 C21	#3 (pink)			
C23 C28 C27 C26 C25 C24	#5 (blue)	1/2+x,3/2-y,1-z		
C46 C51 C50 C49 C48 C47	#6 (green)		3/2-*x*,1-*y*,1/2+z	
Stacking	Interplan angle (°)	Distance plane to centroid (Å)	Dcentr-centr (Å)	Plane to plane shift distance (Å)
#1 #6’’	14.122	3.730(2)	3.759(2)	0.466(5)
#3 #5’	11.618	3.740(2)	3.798(2)	0.661(5)

Table S3. Selected Least-squared planes for **6e**

planes	label	Max deviation (Å) (at.)
C23 C24 C25 C26 C27 C28	Ar_7	0.020 (C26)
C21 C22 C31 C32 C33 C20	Hel_7	0.009 (C32)
C6 C7 C8 C3 C4 C5	Ar_3	0.019 (C7)
C9 C15 C14 C13 C11 C10	Hel_3	0.075 (C13)
C46 C47 C48 C49 C50 C51	Ar_11	0.018 (C46)
C35 C36 C41 C42 C43 C44	Hel_11	0.056 (C35)

Table S4. Selected Inter-plane angles (°) for **6e**

planes	Hel_7	Hel_3	Hel_11
Ar_7	37.8(1)		
Ar_3		26.4(1)	
Ar_11			32.2(1)

Table S5. Crystal data and structure refinement for **6o**.

CCDC number	2171650	
Empirical formula	C53 H59.33 B Cl2 F4 N2 O6.17	
Chemical formula moiety	$C_{48}H_{47}N_2O_5$, $C_4H_{10}O$, BF_4, CH_2Cl_2, 0.167(H_2O)	
Formula weight	980.73	
Temperature	120.00(10) K	
Wavelength	1.54184 Å	
Crystal system	Hexagonal	
Space group	$P6_5$	
Unit cell dimensions	a = 33.49797(18) Å	α = 90°.
	b = 33.49797(18) Å	β = 90°.
	c = 7.78400(4) Å	γ = 120°.
Volume	7564.33(9) $Å^3$	
Z	6	
Density (calculated)	1.292 Mg/m^3	
Absorption coefficient	1.708 mm^{-1}	
F(000)	3094	
Crystal size	0.815 x 0.041 x 0.034 mm^3	
Theta range for data collection	2.638 to 74.352°.	
Index ranges	$-41 \le h \le 40$, $-41 \le k \le 41$, $-9 \le l \le 7$	
Reflections collected	82879	
Independent reflections	9629 [R(int) = 0.0330]	
Completeness to theta = 67.684°	99.9 %	
Absorption correction	Analytical	
Max. and min. transmission	0.947 and 0.535	
Refinement method	Full-matrix least-squares on F^2	
Data / restraints / parameters	9629 / 2 / 618	
Goodness-of-fit on F^2	1.076	
Final R indices [I>2sigma(I)]	R_1 = 0.0826, wR_2 = 0.2290	
R indices (all data)	R_1 = 0.0865, wR_2 = 0.2330	
Absolute structure parameter	0.05(5)	
Extinction coefficient	n/a	
Largest diff. peak and hole	0.611 and -0.580 e.$Å^{-3}$	

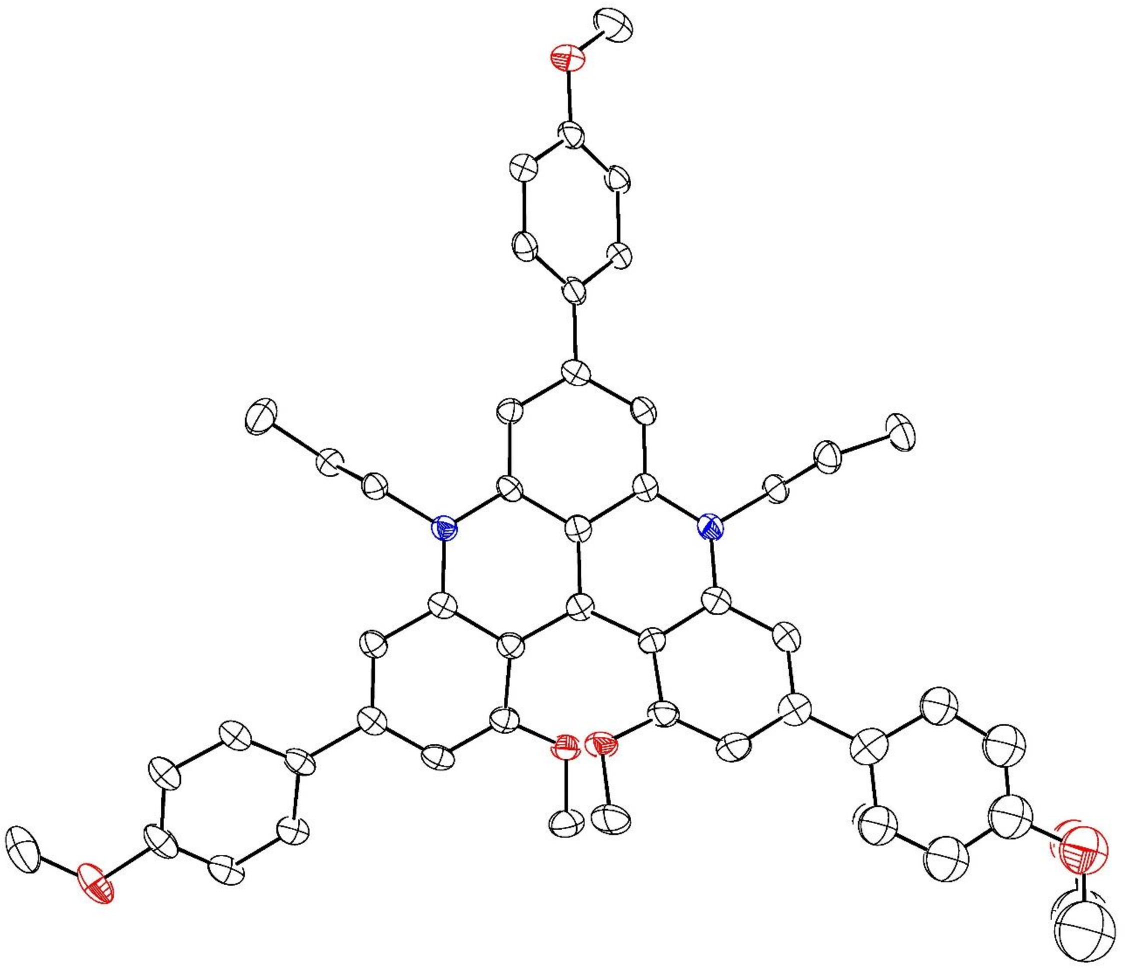

Figure S110. Ortep View of molecular **6o** (thermal ellipsoids are drawn at 50% probability level). Counter ion and solvent molecules are omitted for clarity purpose.

Remark: One ArOMe part is slightly disordered and refined in two components with atomic occupancies 0.68/0.32 and with isotropic atomic displacement parameters constrained to be equals.

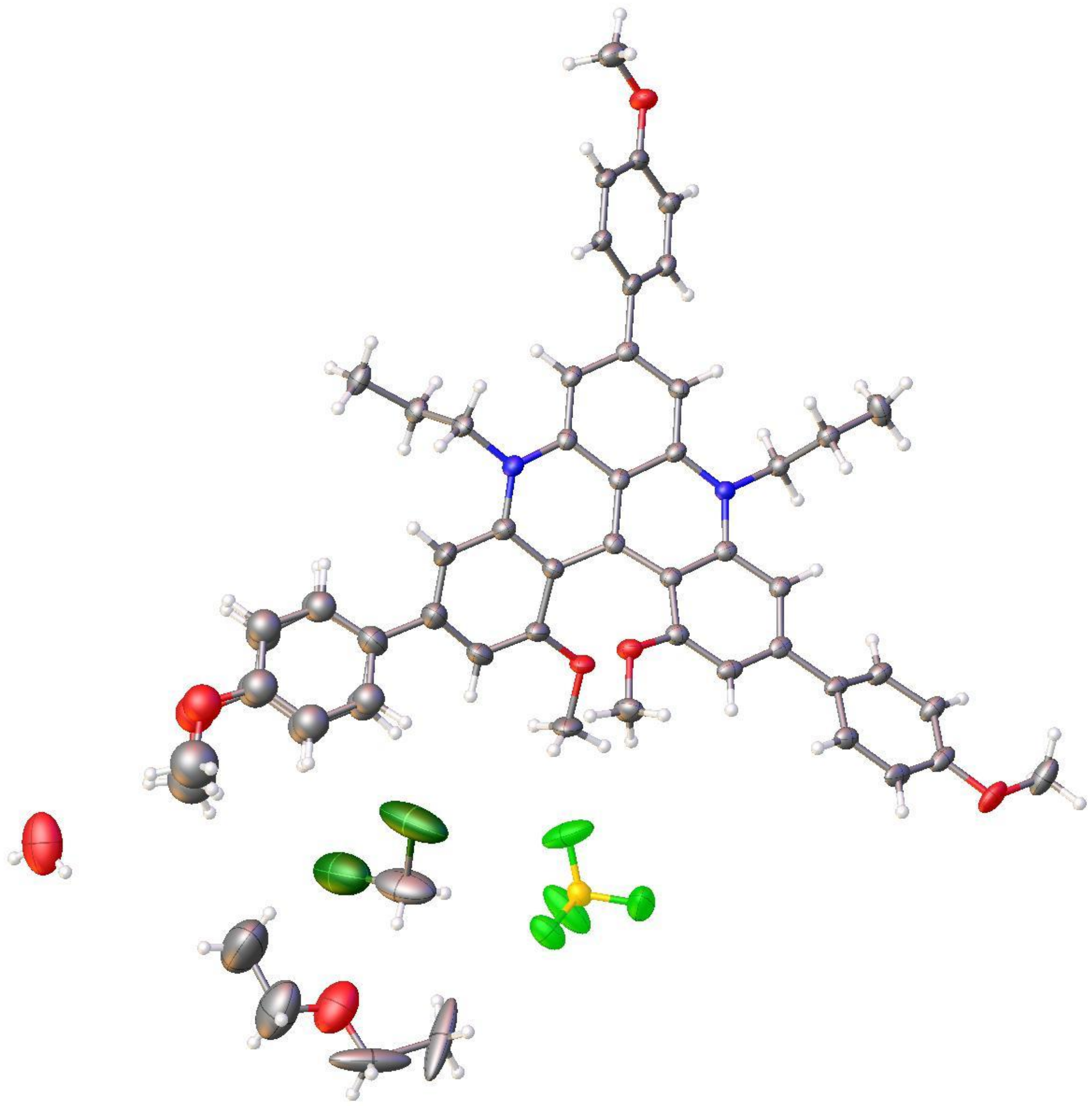

Figure S111. View of the asymmetric unit of **6o**

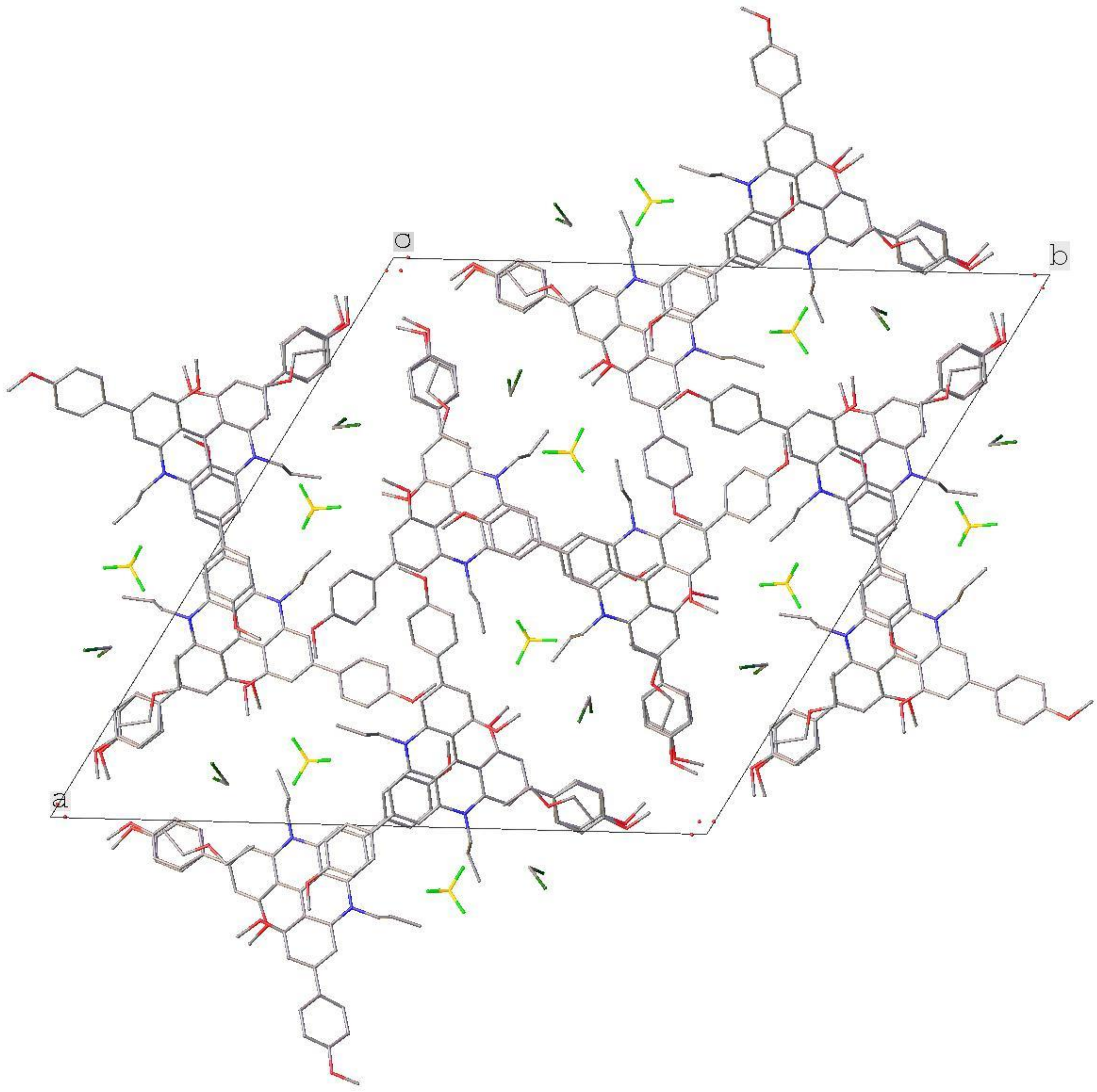

Figure S112. View along the *c* direction showing the packing of the molecules in **6o** (π-π stacking along *c* direction).

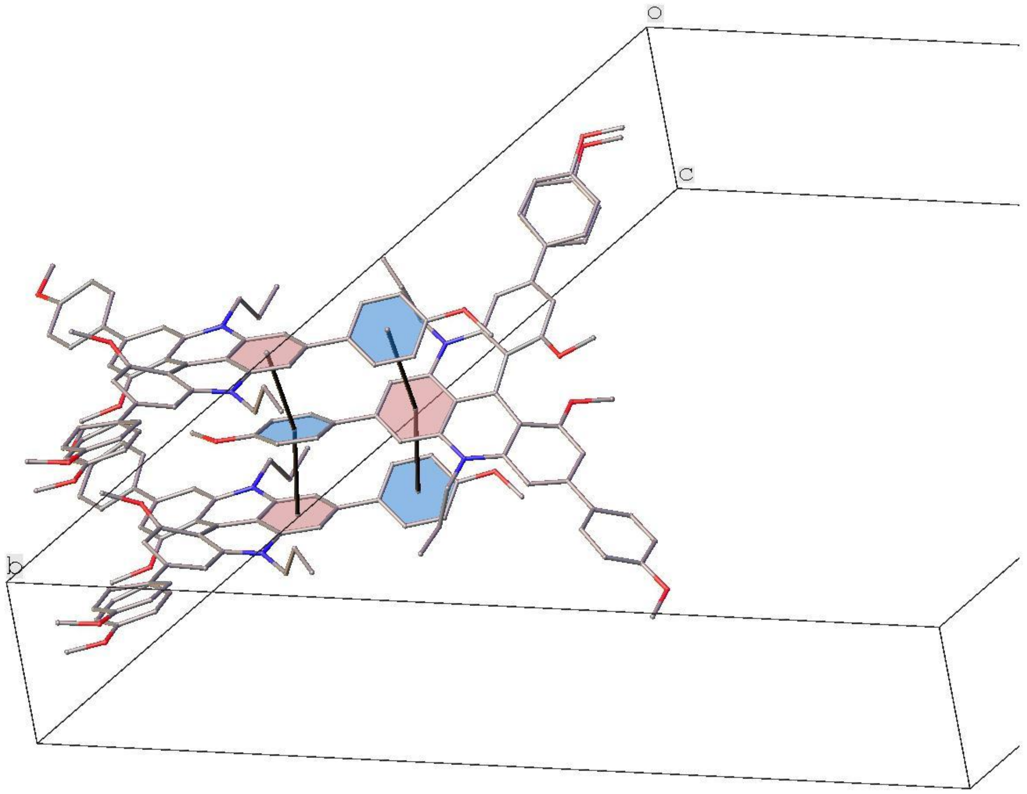

Figure S113. Detailed π-π stacking interactions found in **6o** (highlighted with black line). Aryl groups in blue (position 7) and ring of DMQA core (pink).

Table S6. Intermolecular interactions analysis in **6o**.

Planes	Label	Sym op ‘	Sym op’’	
C2-C3-C4-C5-C6-C7	#1			
C8-C9-C10-C11-C12-C13	#2	1-*x*, 1-*y*, -1/2+*z*	1-*x*, 1-*y*, 1/2+*z*	
Stacking	Interplan angle (°)	Distance plane to centroid (Å)	Dcentr-centr (Å)	Plane to plane shift distance (Å)
#1 #2’	7.36(2)	3.679(4)	3.864	1.635(3)
#1 #2’’	7.36(2)	3.663(5)	3.971(3)	1.534(9)

Table S7. Selected Least-squared plane deviation for **6o**

planes	label	Max deviation (Å) (at.)
C5 C4 C3 C2 C7 C6	Ar_7	0.013 (C6)
C9 C8 C13 C12 C11 C10	Hel_7	0.006 (C8)
C26 C25 C24 C29 C28 C27	Ar_3	0.032 (C28)
C21 C20 C17 C18 C23 C22	Hel_3	0.090 (C18)
C41 C46A C45A C44A C43A C42A	Ar_11a	0.066 (C41)
C46B C45B C44B C43B C42B C41	Ar_11b	0.118 (C42B)
C32 C37 C36 C35 C34 C33	Hel_11	0.083 (C32)

Table S8. Selected Inter-plane angles (°) for **6o**

planes	Hel_7	Hel_3	Hel_11
Ar_7	38.7(2)		
Ar_3		18.9(2)	
Ar_11a			32.2(4)
Ar_11b			30.1(8)

Position_3 and position_11 mean angle (°) with standard deviation= 28.0(5.6)
Position_7 mean angle (°) with standard deviation= 38.3(0.6)

Optical Properties

Absorbance and luminescence

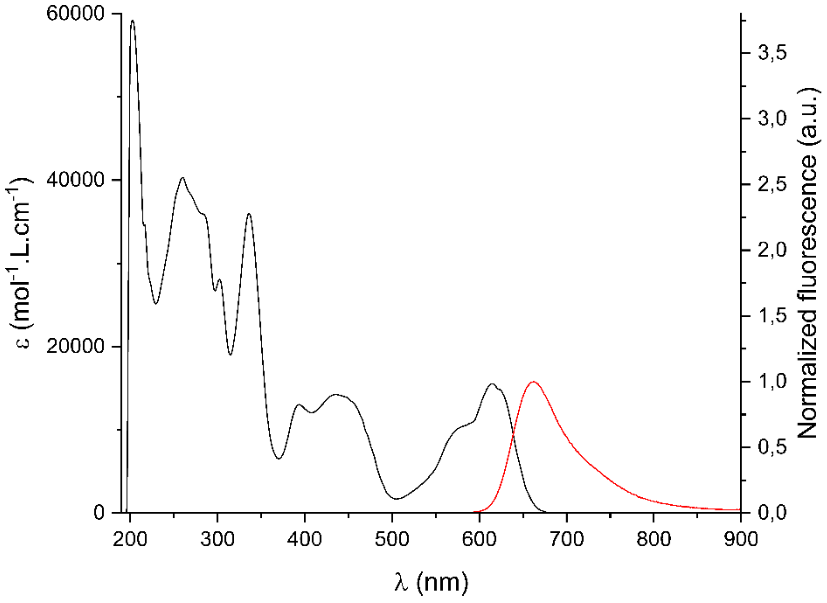

Figure S114. Absorption (black) and emission (red) spectra of compounds **6a** in acetonitrile.

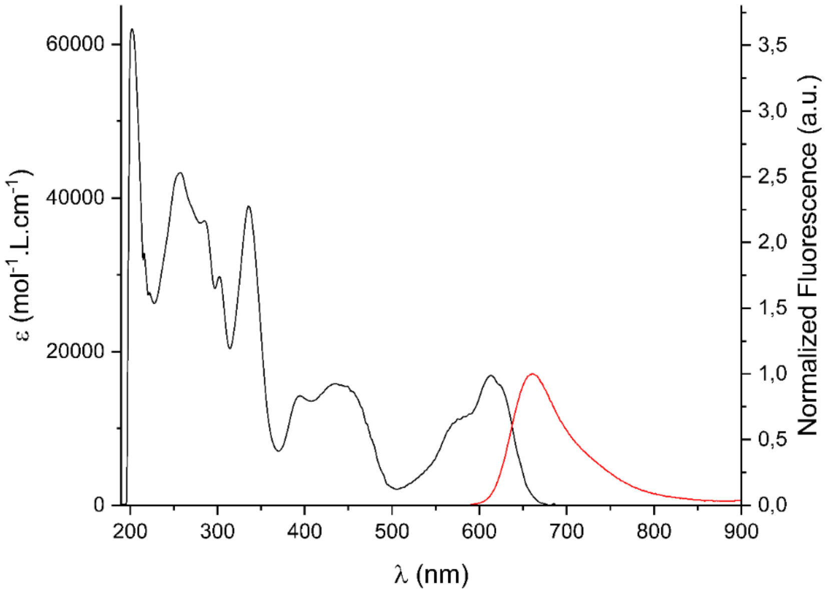

Figure S115. Absorption (black) and emission (red) spectra of compounds **6b** in acetonitrile.

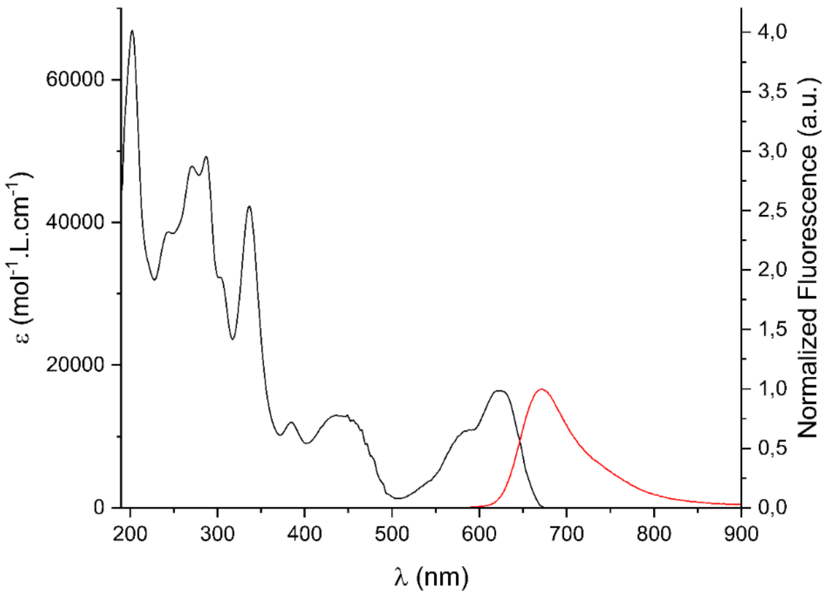

Figure S116. Absorption (black) and emission (red) spectra of compounds **6c** in acetonitrile.

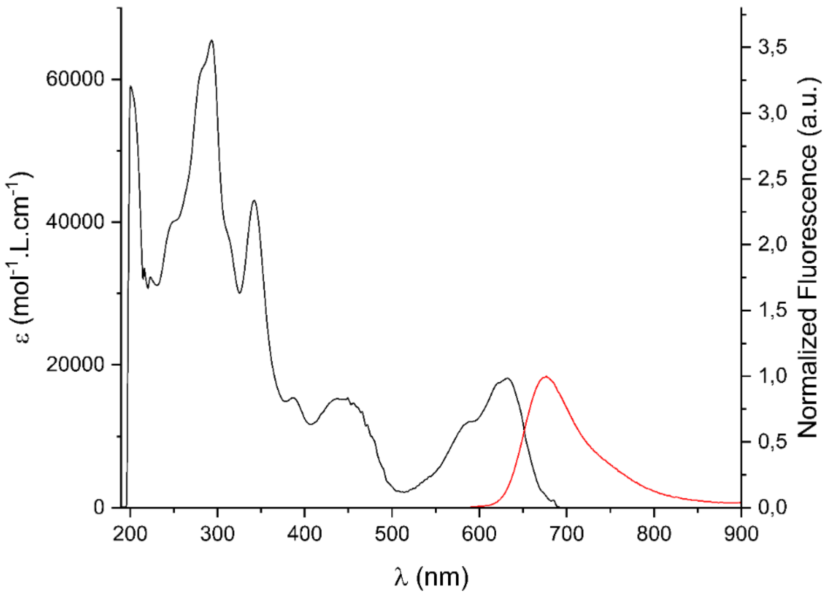

Figure S117. Absorption (black) and emission (red) spectra of compounds **6d** in acetonitrile.

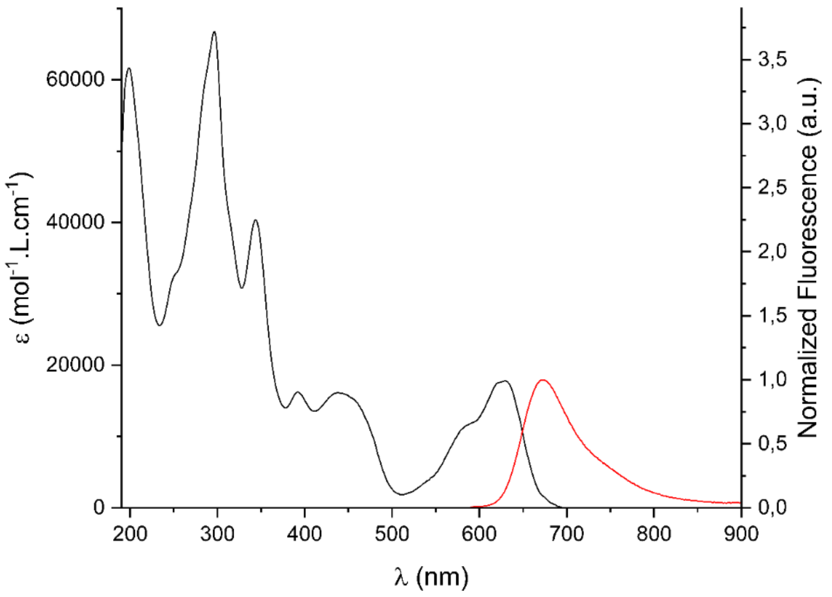

Figure S118. Absorption (black) and emission (red) spectra of compounds **6e** in acetonitrile.

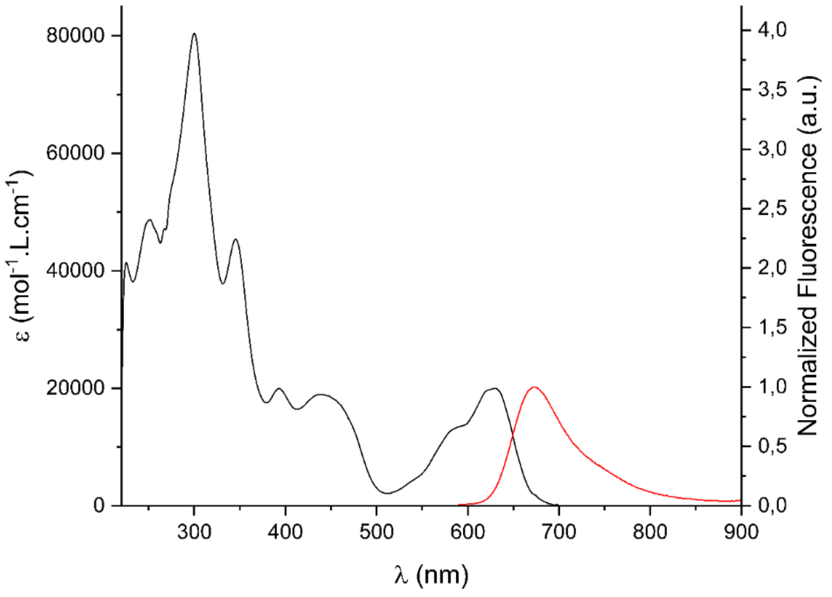

Figure S119. Absorption (black) and emission (red) spectra of compounds **6f** in acetonitrile.

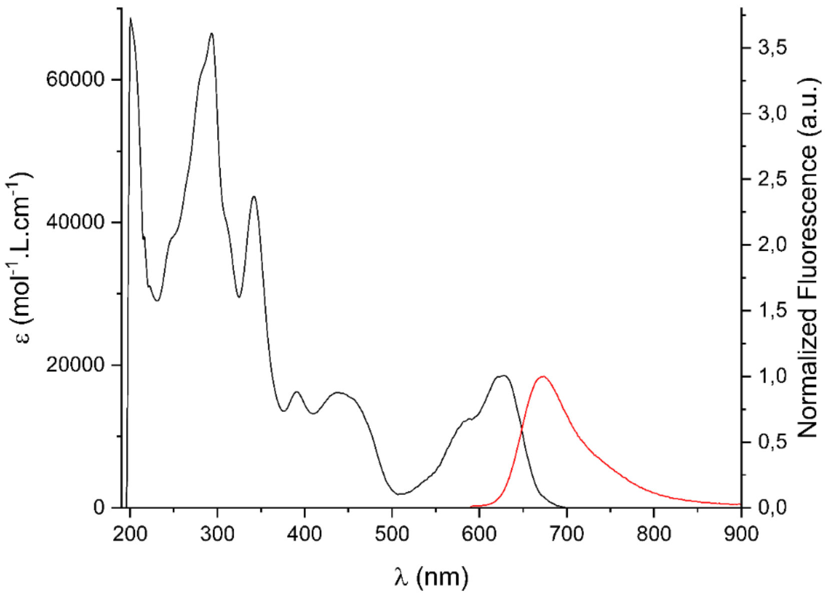

Figure S120. Absorption (black) and emission (red) spectra of compounds **6g** in acetonitrile.

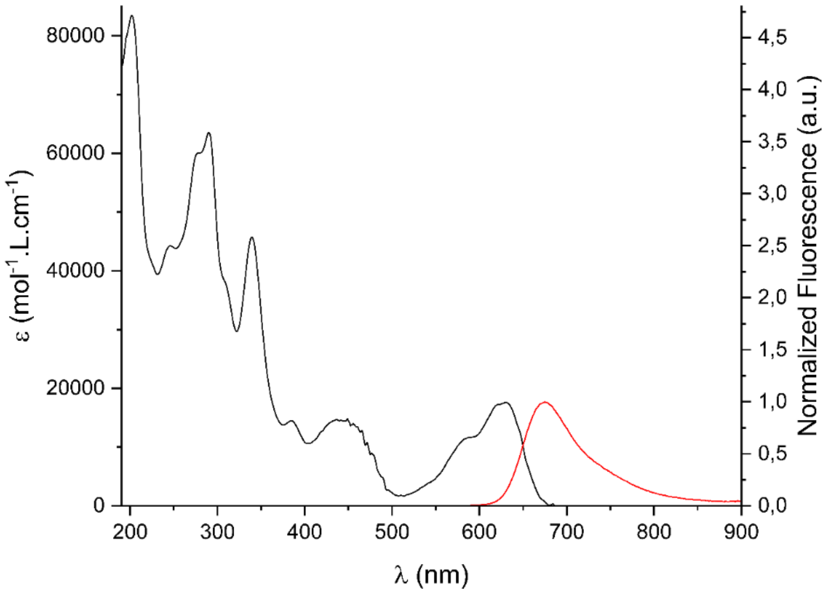

Figure S121. Absorption (black) and emission (red) spectra of compounds **6h** in acetonitrile.

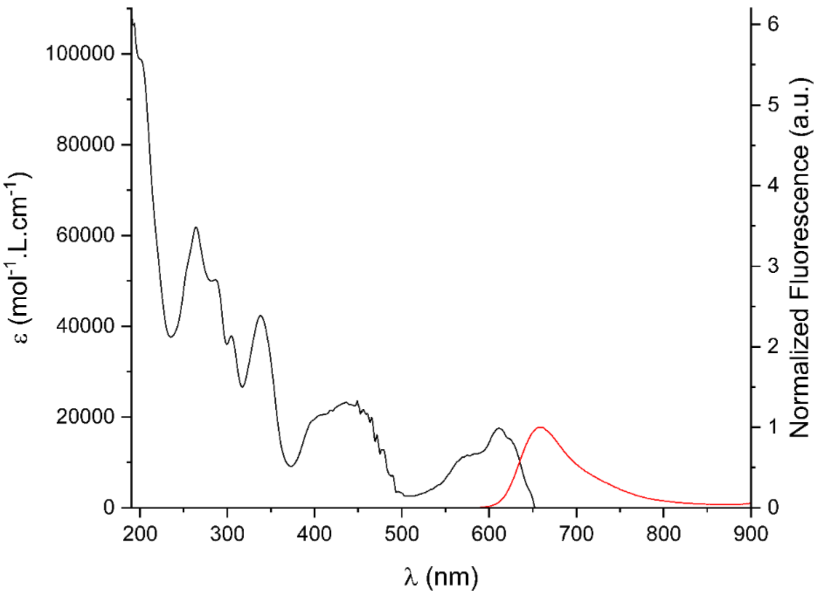

Figure S122. Absorption (black) and emission (red) spectra of compounds **6i** in acetonitrile.

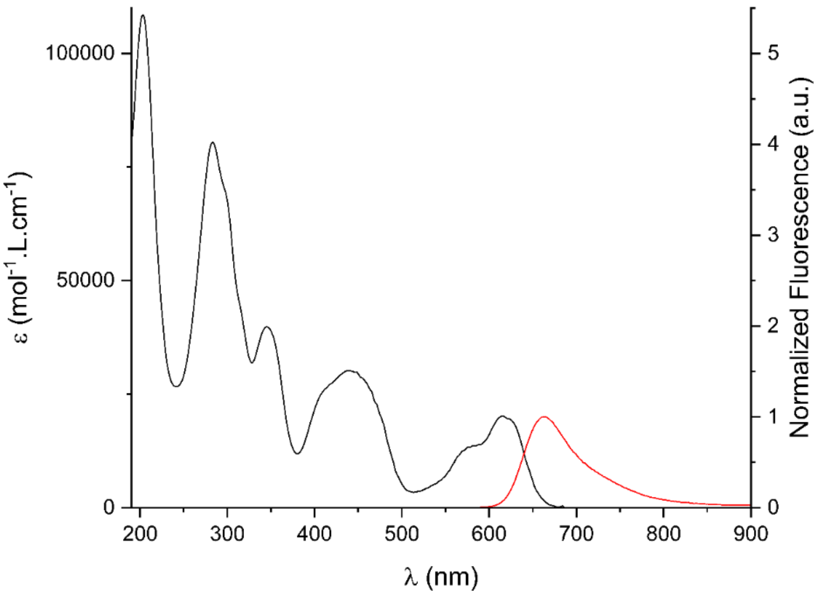

Figure S123. Absorption (black) and emission (red) spectra of compounds **6j** in acetonitrile.

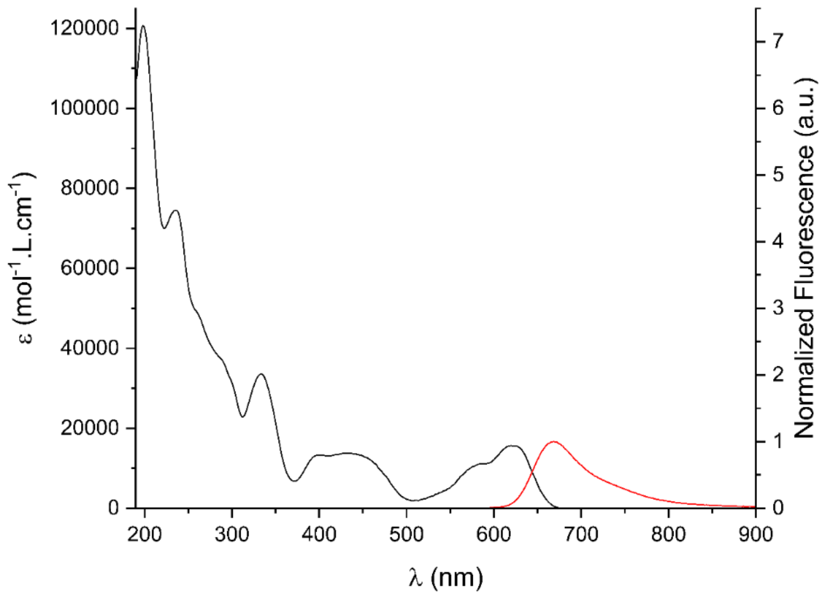

Figure S124. Absorption (black) and emission (red) spectra of compounds **6k** in acetonitrile.

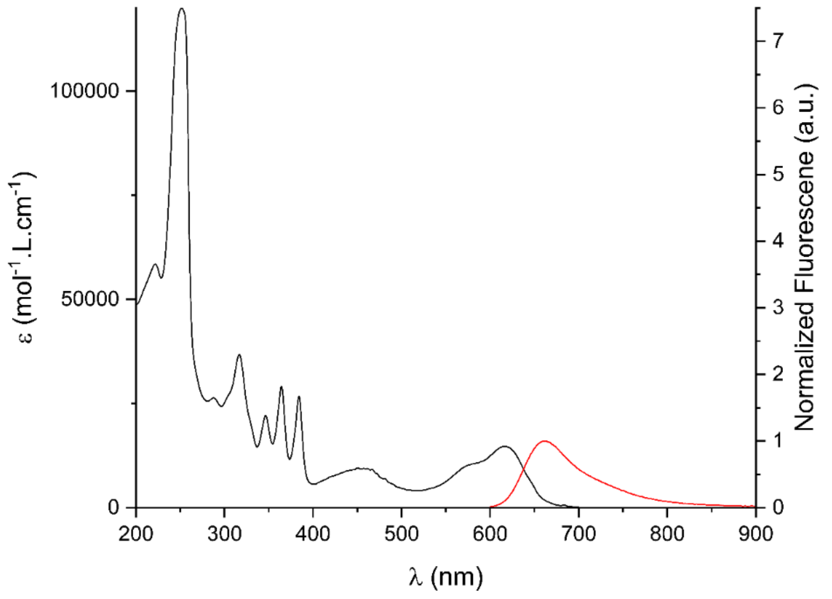

Figure S125. Absorption (black) and emission (red) spectra of compounds **6l** in acetonitrile.

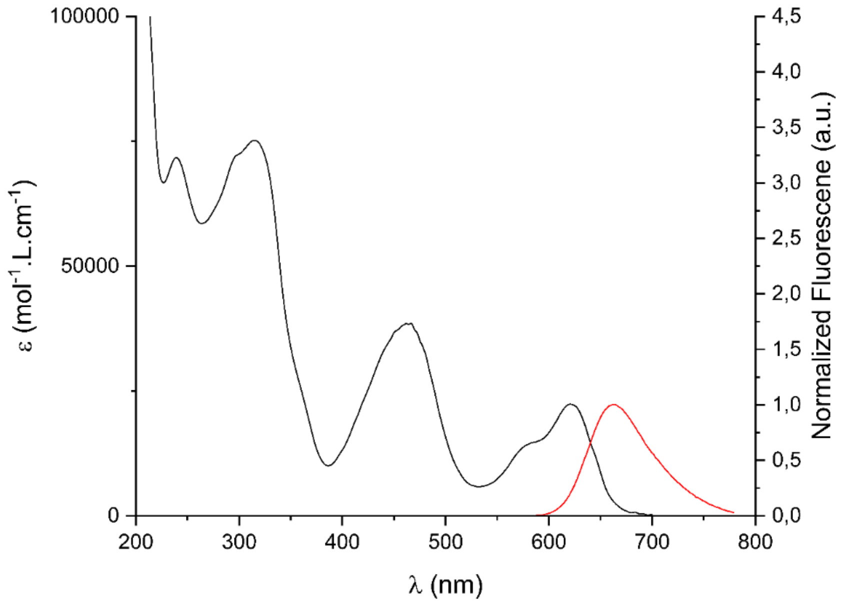

Figure S126. Absorption (black) and emission (red) spectra of compounds **6m** in acetonitrile.

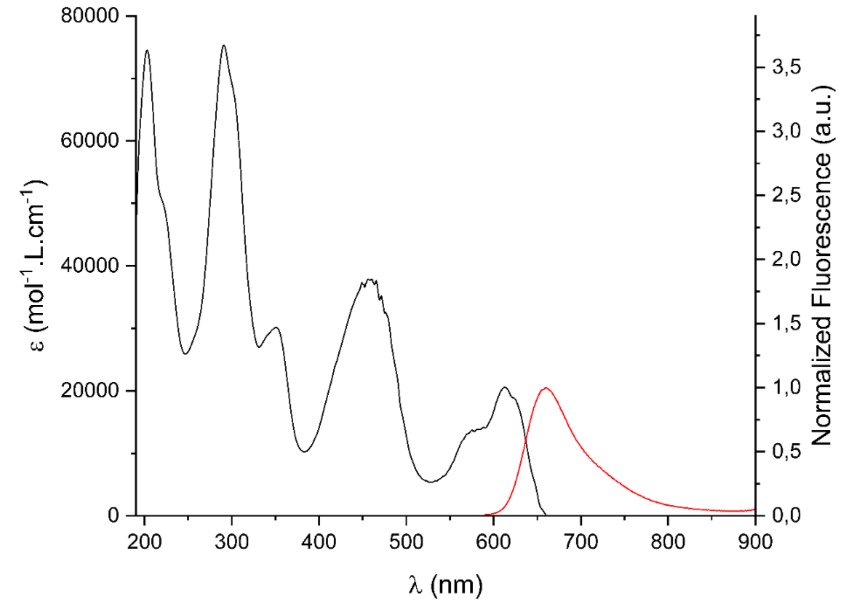

Figure S127. Absorption (black) and emission (red) spectra of compounds **6n** in acetonitrile.

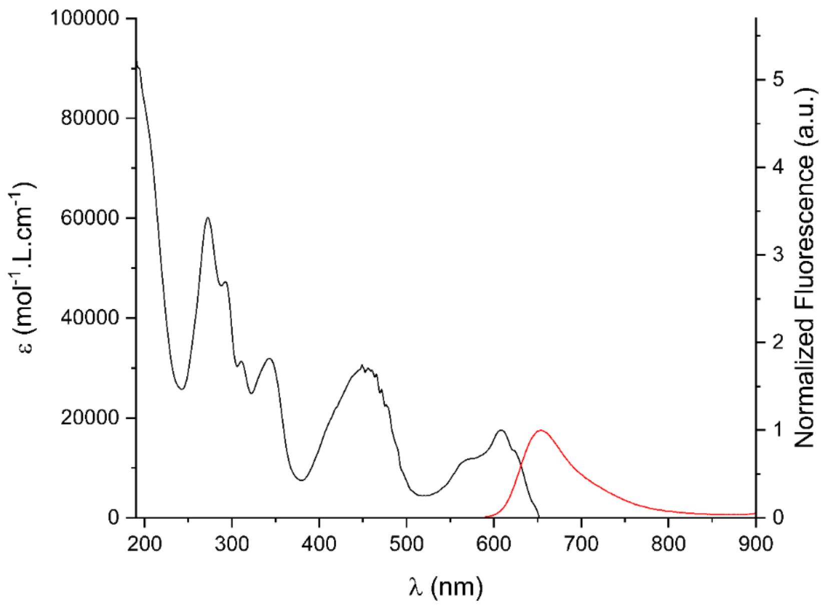

Figure S128. Absorption (black) and emission (red) spectra of compounds **6o** in acetonitrile.

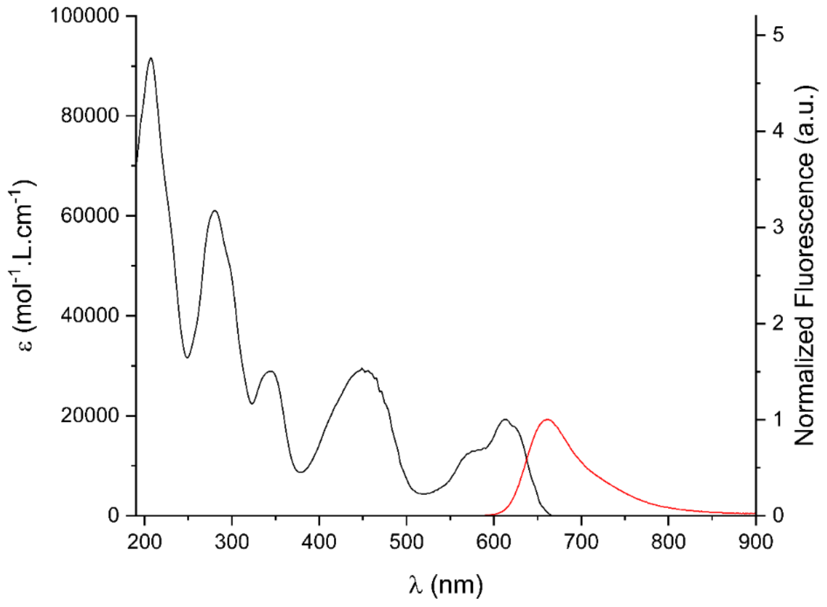

Figure S129. Absorption (black) and emission (red) spectra of compounds **6p** in acetonitrile.

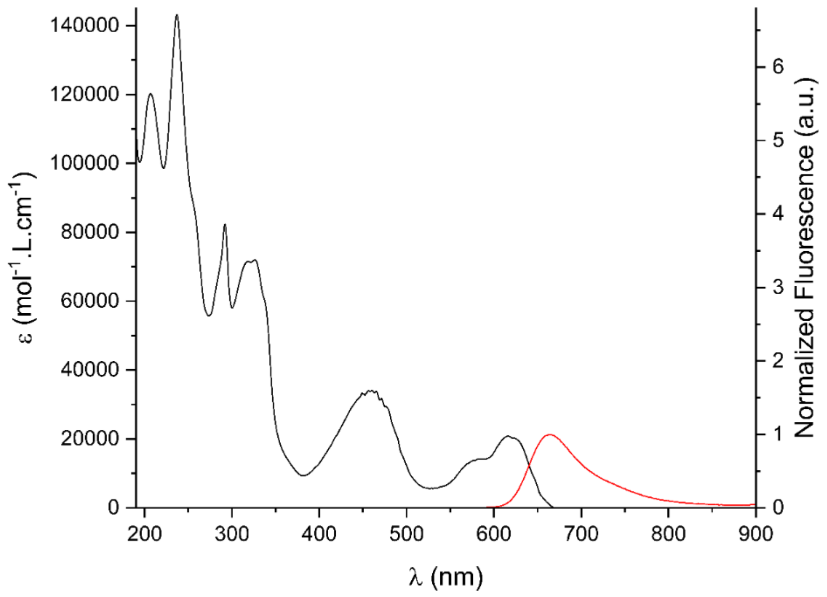

Figure S130. Absorption (black) and emission (red) spectra of compounds **6q** in acetonitrile.

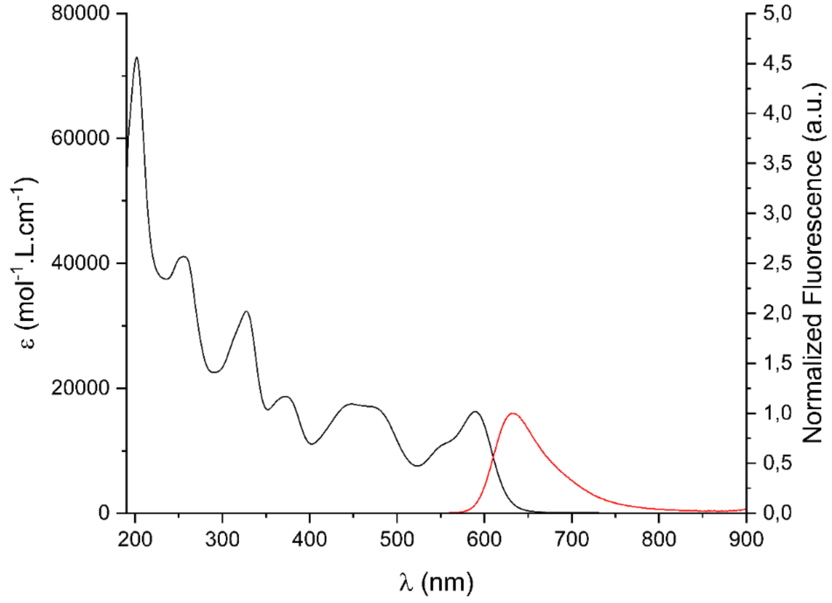

Figure S131. Absorption (black) and emission (red) spectra of compounds **9** in acetonitrile.

Additional spectrum

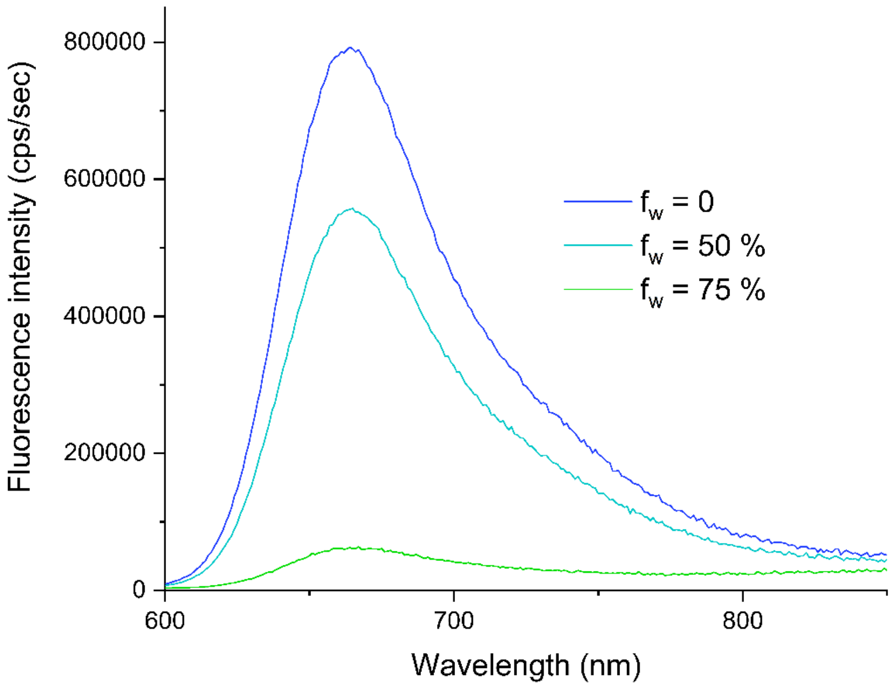

Figure S132. Luminescence spectra of compounds **6m** in acetonitrile/water mixture (f_w: fraction of water in volume). Excitation wavelength: 488 nm

Chiroptical Properties

Electronic Circular Dichroism and g_{abs}

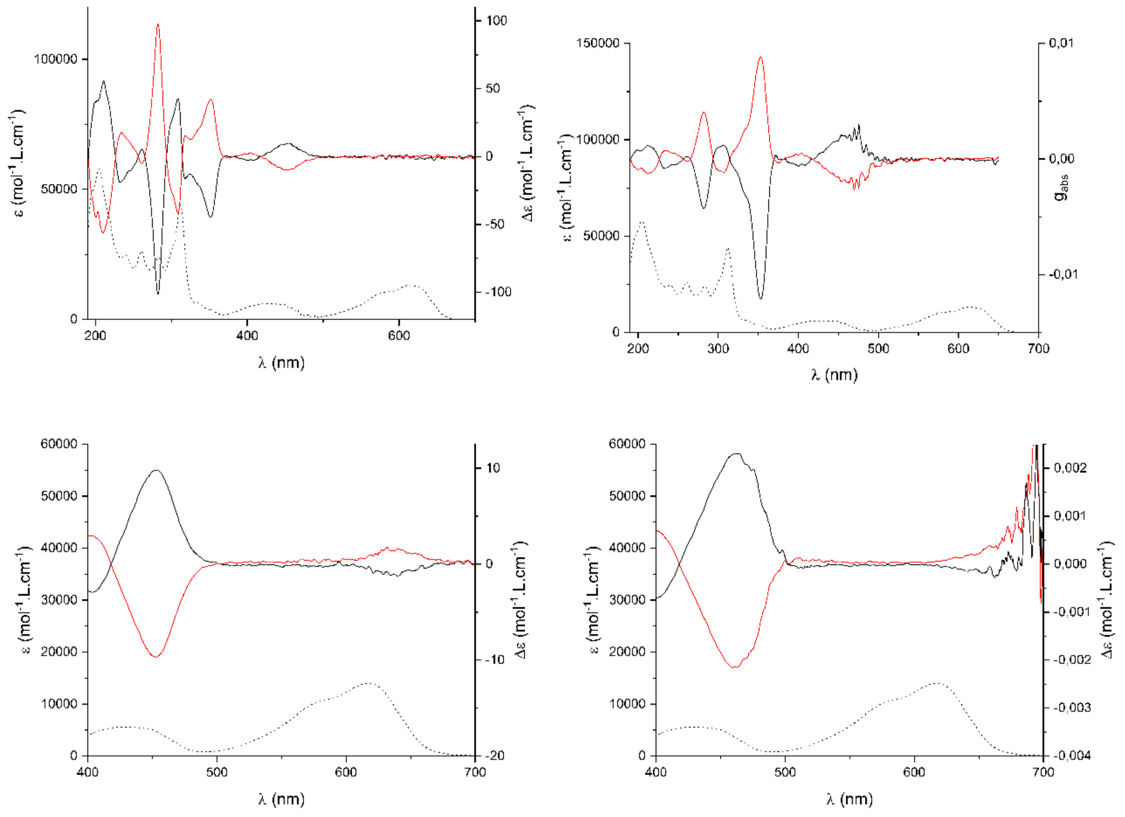

Figure S133. Absorption (dotted black), circular dichroism (left, (*M*)- enantiomer in black, (*P*)- enantiomer in red) and g_{abs} (right, (*M*)- enantiomer in black, (*P*)- enantiomer in red) spectra of compounds **1** in acetonitrile. Top: entire spectra, bottom: zoom between 400-700 nm.

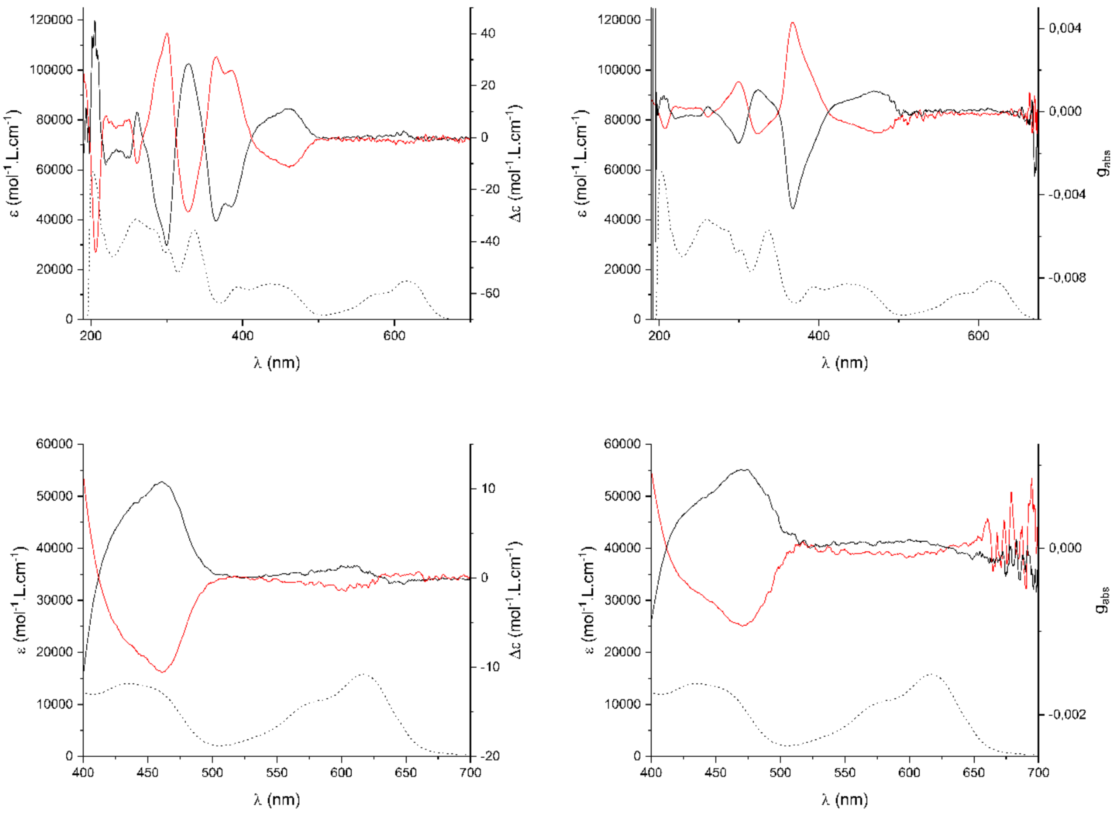

Figure S134. Absorption (dotted black), circular dichroism (left, (*M*)- enantiomer in black, (*P*)- enantiomer in red) and g_{abs} (right, (*M*)- enantiomer in black, (*P*)- enantiomer in red) spectra of compounds **6a** in acetonitrile. Top: entire spectra, bottom: zoom between 400-700 nm.

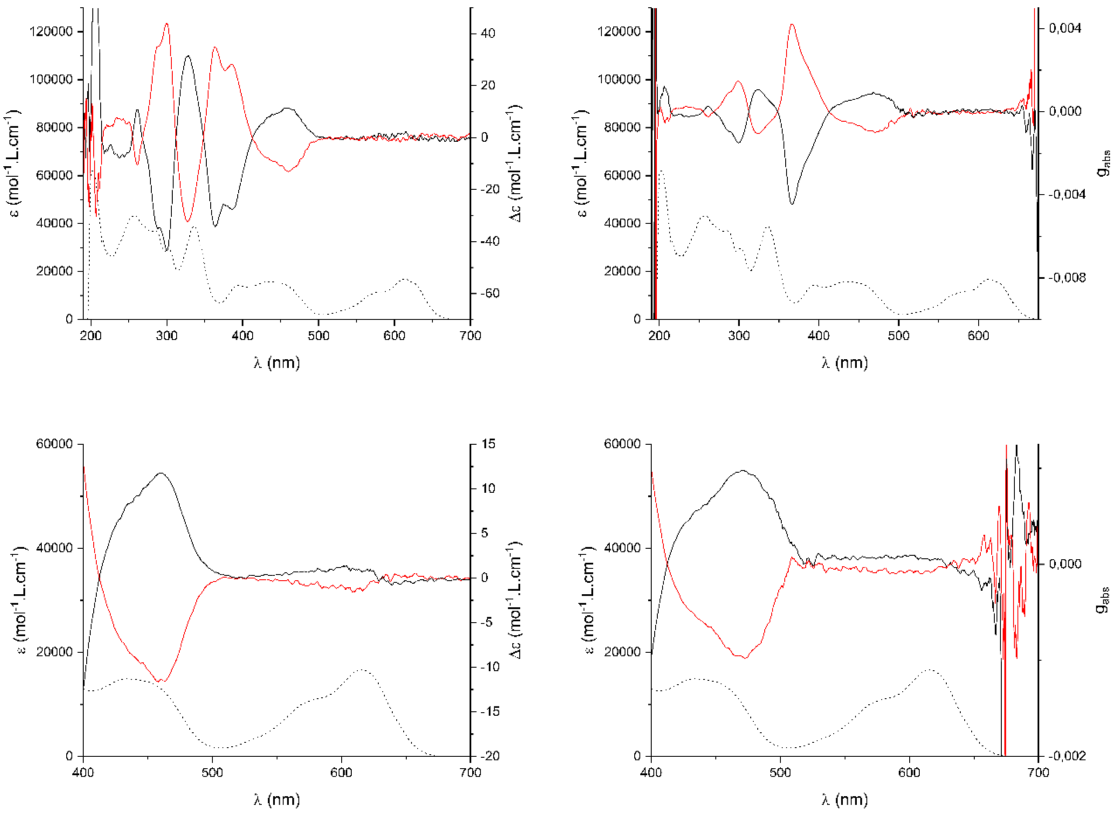

Figure S135. Absorption (dotted black), circular dichroism (left, (*M*)- enantiomer in black, (*P*)- enantiomer in red) and g_{abs} (right, (*M*)- enantiomer in black, (*P*)- enantiomer in red) spectra of compounds **6b** in acetonitrile. Top: entire spectra, bottom: zoom between 400-700 nm.

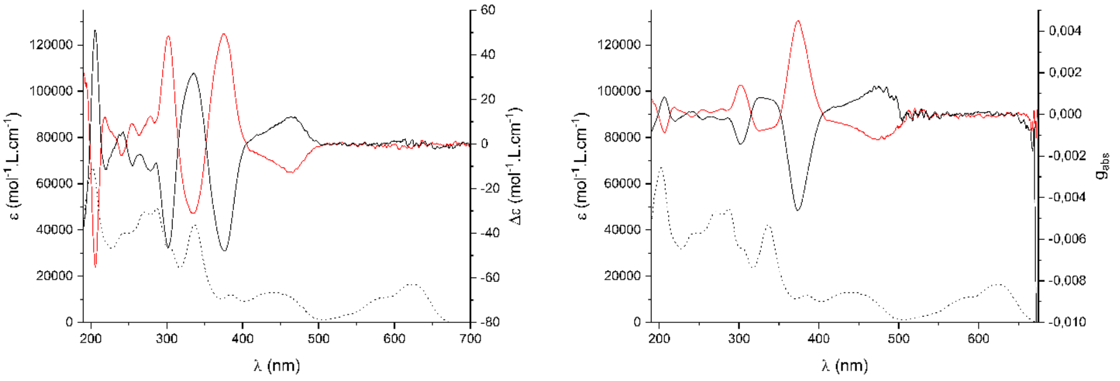

Figure S136. Absorption (dotted black), circular dichroism (left, (*M*)- enantiomer in black, (*P*)- enantiomer in red) and g_{abs} (right, (*M*)- enantiomer in black, (*P*)- enantiomer in red) spectra of compounds **6c** in acetonitrile.

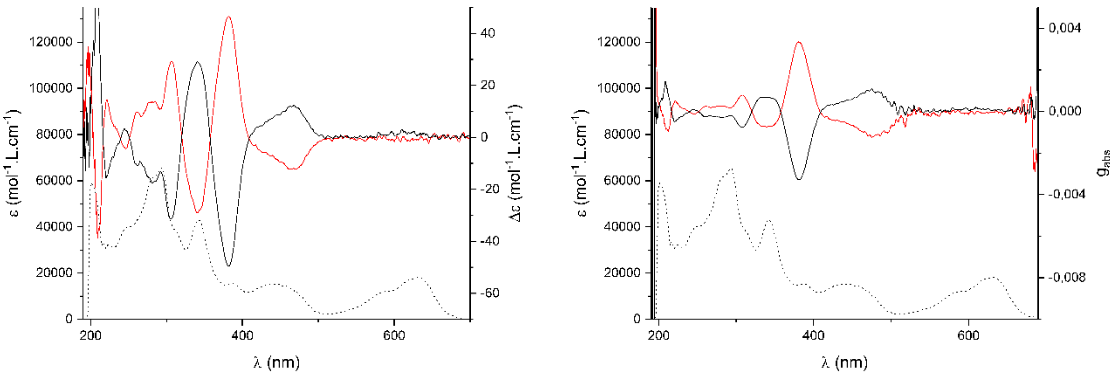

Figure S137. Absorption (dotted black), circular dichroism (left, (*M*)- enantiomer in black, (*P*)- enantiomer in red) and g_{abs} (right, (*M*)- enantiomer in black, (*P*)- enantiomer in red) spectra of compounds **6d** in acetonitrile.

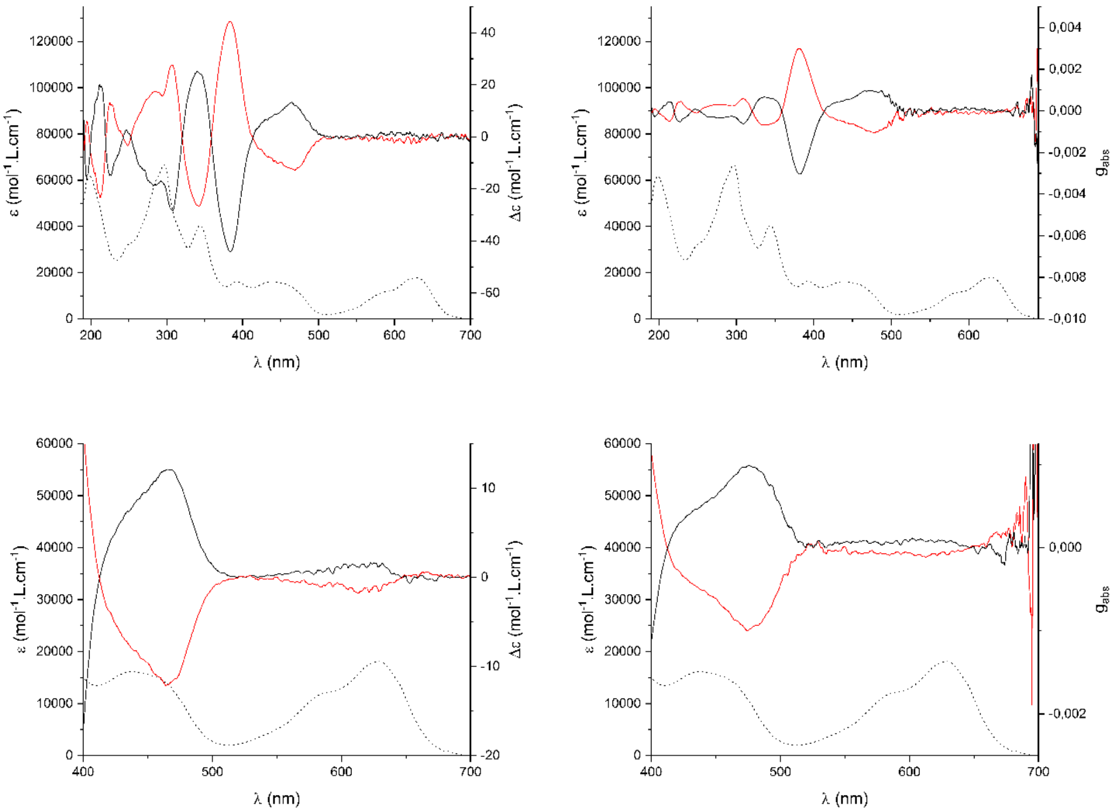

Figure S138. Absorption (dotted black), circular dichroism (left, (*M*)- enantiomer in black, (*P*)- enantiomer in red) and g_{abs} (right, (*M*)- enantiomer in black, (*P*)- enantiomer in red) spectra of compounds **6e** in acetonitrile. Top: entire spectra, bottom: zoom between 400-700 nm.

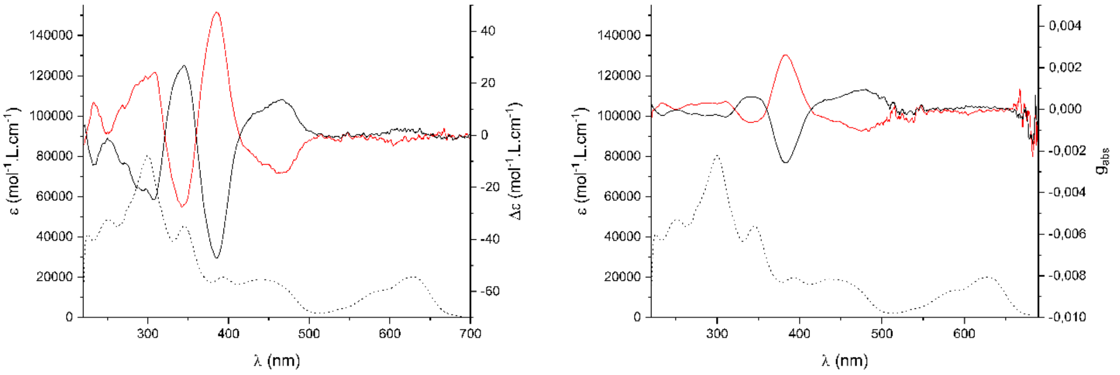

Figure S139. Absorption (dotted black), circular dichroism (left, (*M*)- enantiomer in black, (*P*)- enantiomer in red) and g_{abs} (right, (*M*)- enantiomer in black, (*P*)- enantiomer in red) spectra of compounds **6f** in acetonitrile.

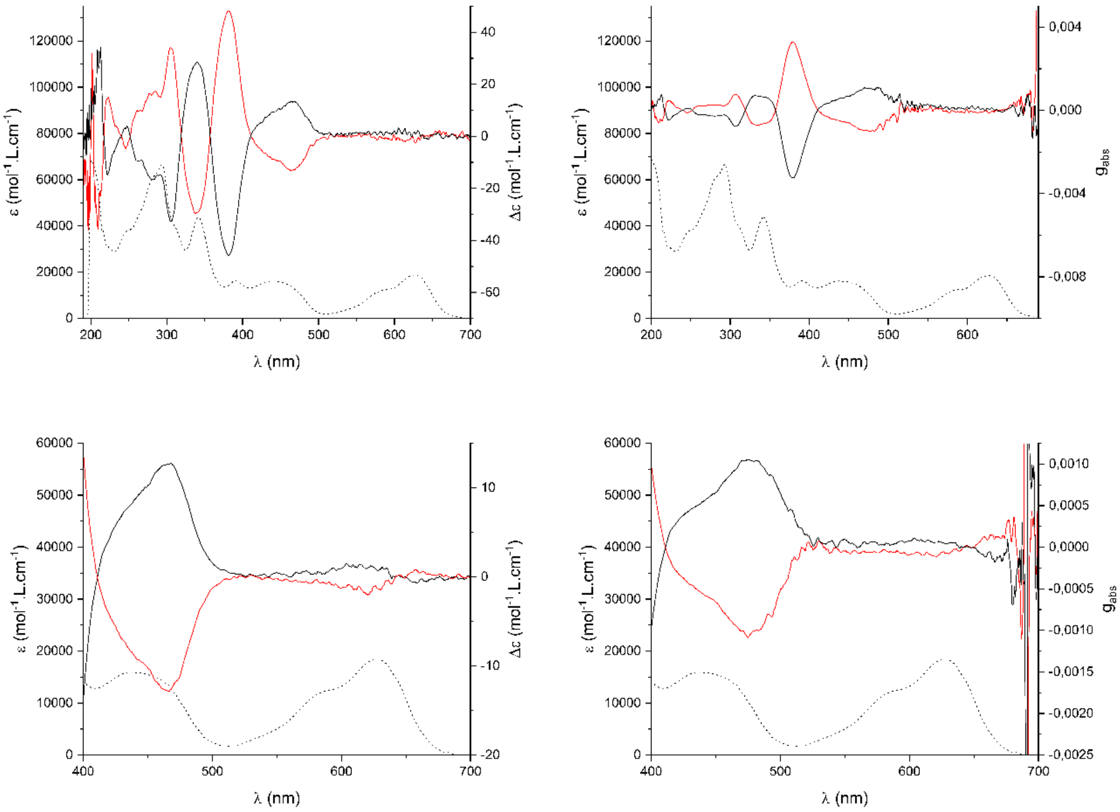

Figure S140. Absorption (dotted black), circular dichroism (left, (*M*)- enantiomer in black, (*P*)- enantiomer in red) and g_{abs} (right, (*M*)- enantiomer in black, (*P*)- enantiomer in red) spectra of compounds **6g** in acetonitrile. Top: entire spectra, bottom: zoom between 400-700 nm.

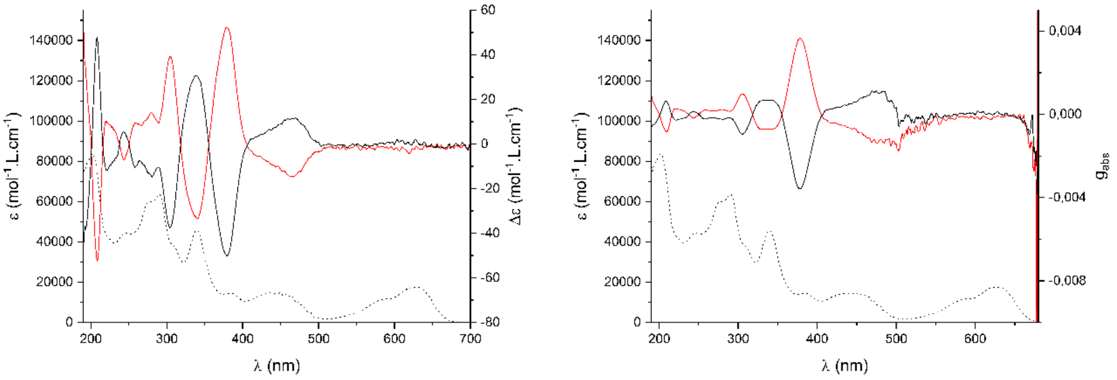

Figure S141. Absorption (dotted black), circular dichroism (left, (*M*)- enantiomer in black, (*P*)- enantiomer in red) and g_{abs} (right, (*M*)- enantiomer in black, (*P*)- enantiomer in red) spectra of compounds **6h** in acetonitrile.

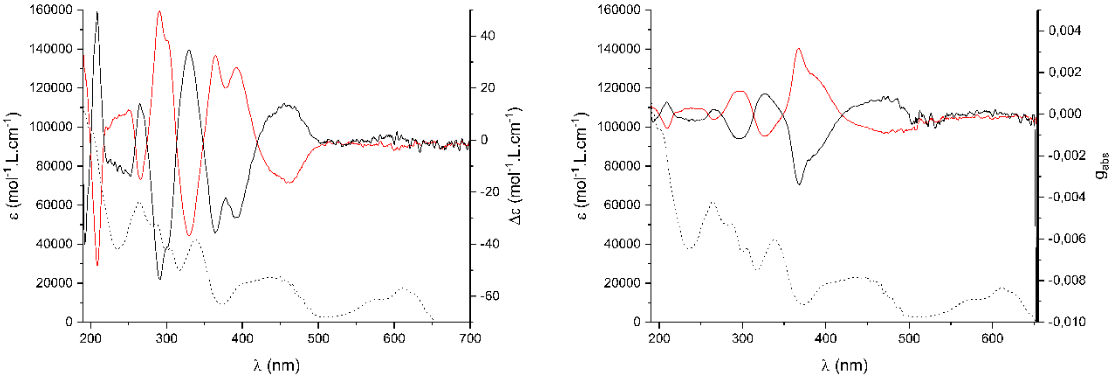

Figure S142. Absorption (dotted black), circular dichroism (left, (*M*)- enantiomer in black, (*P*)- enantiomer in red) and g_{abs} (right, (*M*)- enantiomer in black, (*P*)- enantiomer in red) spectra of compounds **6i** in acetonitrile.

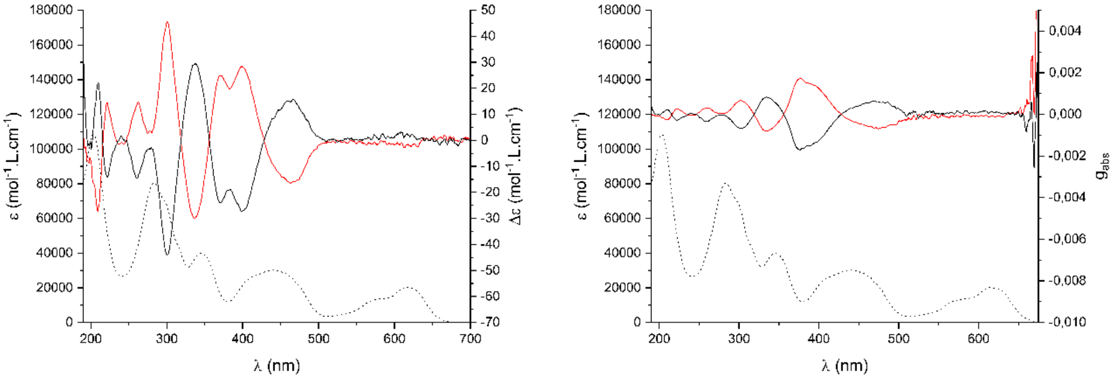

Figure S143. Absorption (dotted black), circular dichroism (left, (*M*)- enantiomer in black, (*P*)- enantiomer in red) and g_{abs} (right, (*M*)- enantiomer in black, (*P*)- enantiomer in red) spectra of compounds **6j** in acetonitrile.

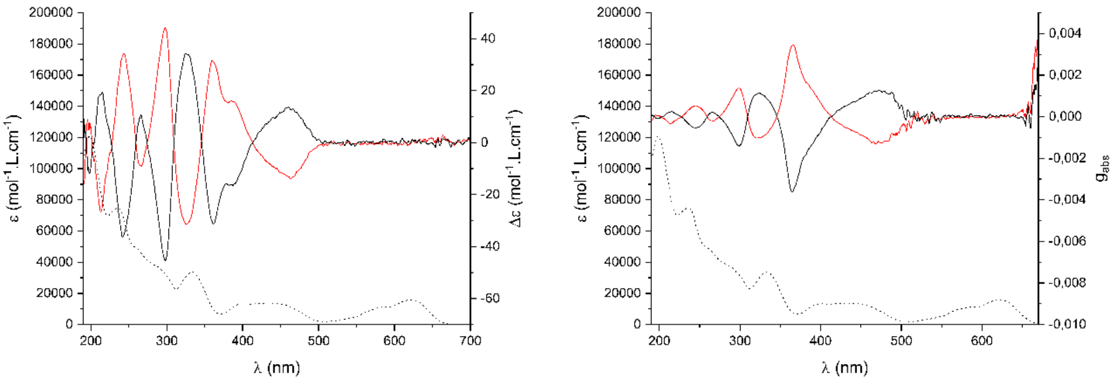

Figure S144. Absorption (dotted black), circular dichroism (left, (*M*)- enantiomer in black, (*P*)- enantiomer in red) and g_{abs} (right, (*M*)- enantiomer in black, (*P*)- enantiomer in red) spectra of compounds **6k** in acetonitrile.

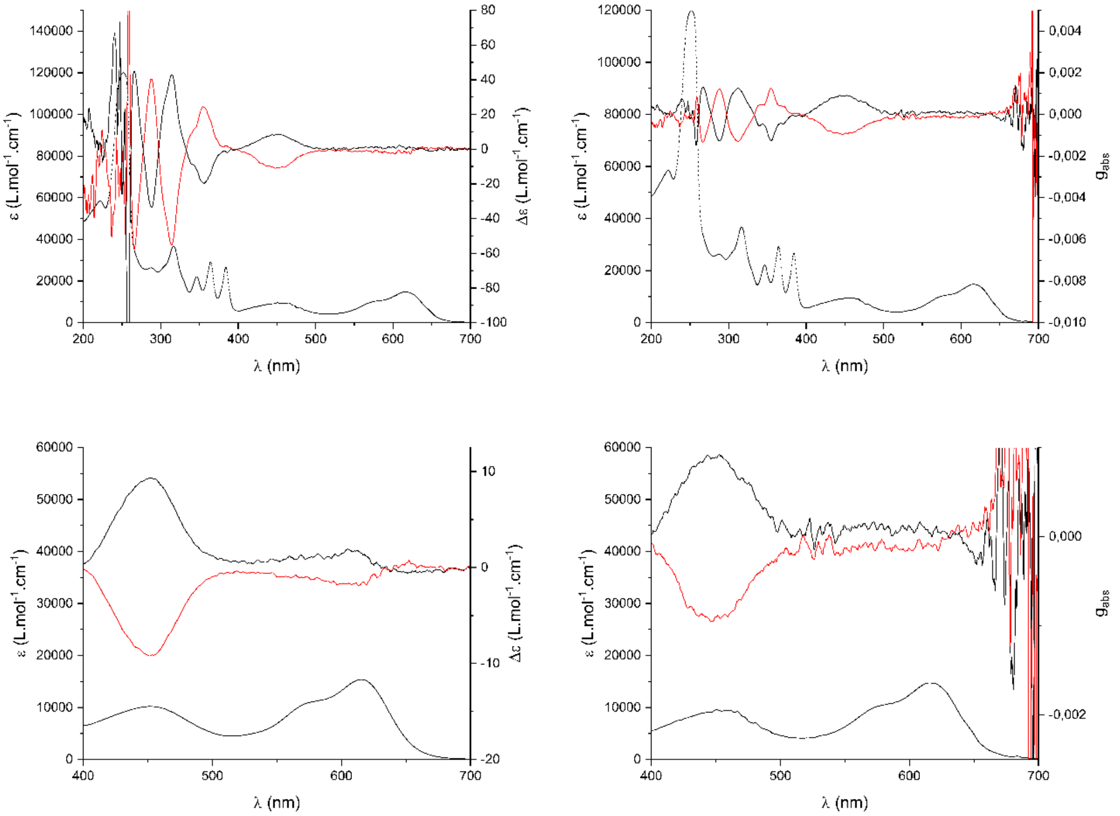

Figure S145. Absorption (dotted black), circular dichroism (left, (*M*) enantiomer in black, (*P*) enantiomer in red) and g_{abs} (right, (*M*) enantiomer in black, (*P*) enantiomer in red) spectra of compounds **6l** in acetonitrile. Top: entire spectra, bottom: zoom between 400-700 nm.

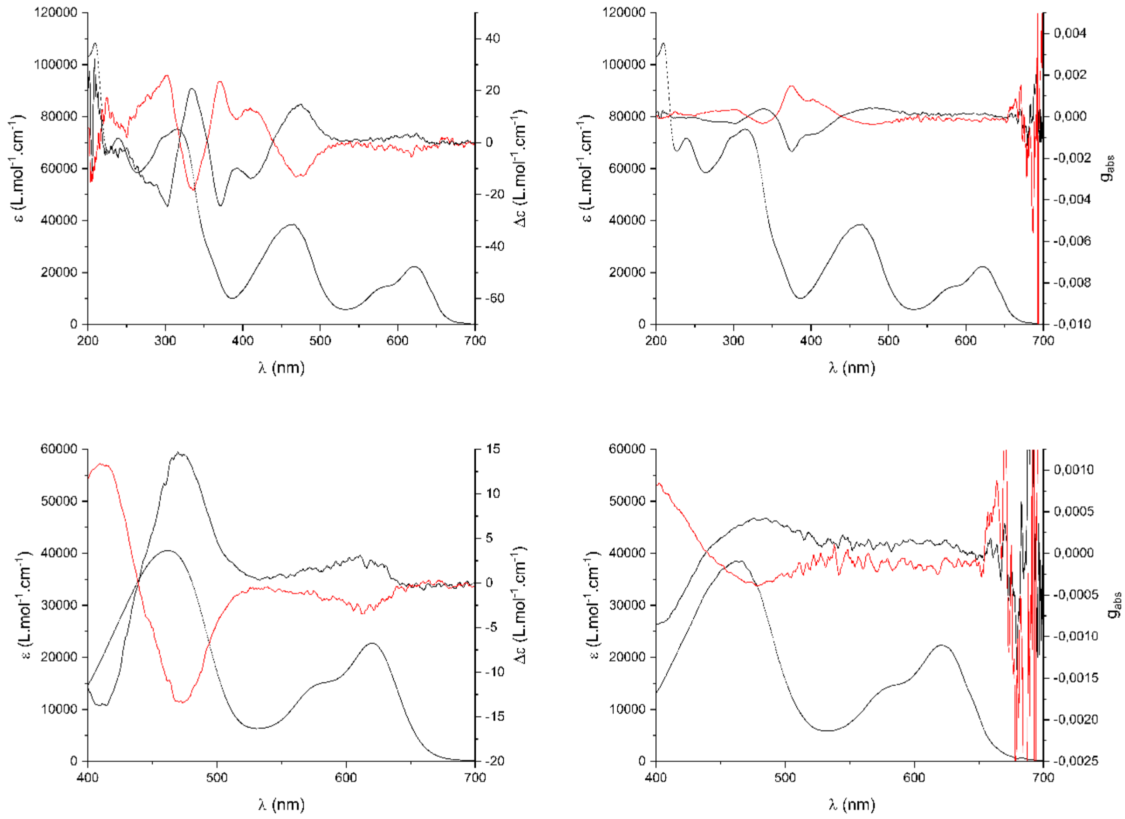

Figure S146. Absorption (dotted black), circular dichroism (left, (*M*)- enantiomer in black, (*P*)- enantiomer in red) and g_{abs} (right, (*M*)- enantiomer in black, (*P*)- enantiomer in red) spectra of compounds **6m** in acetonitrile. Top: entire spectra, bottom: zoom between 400-700 nm.

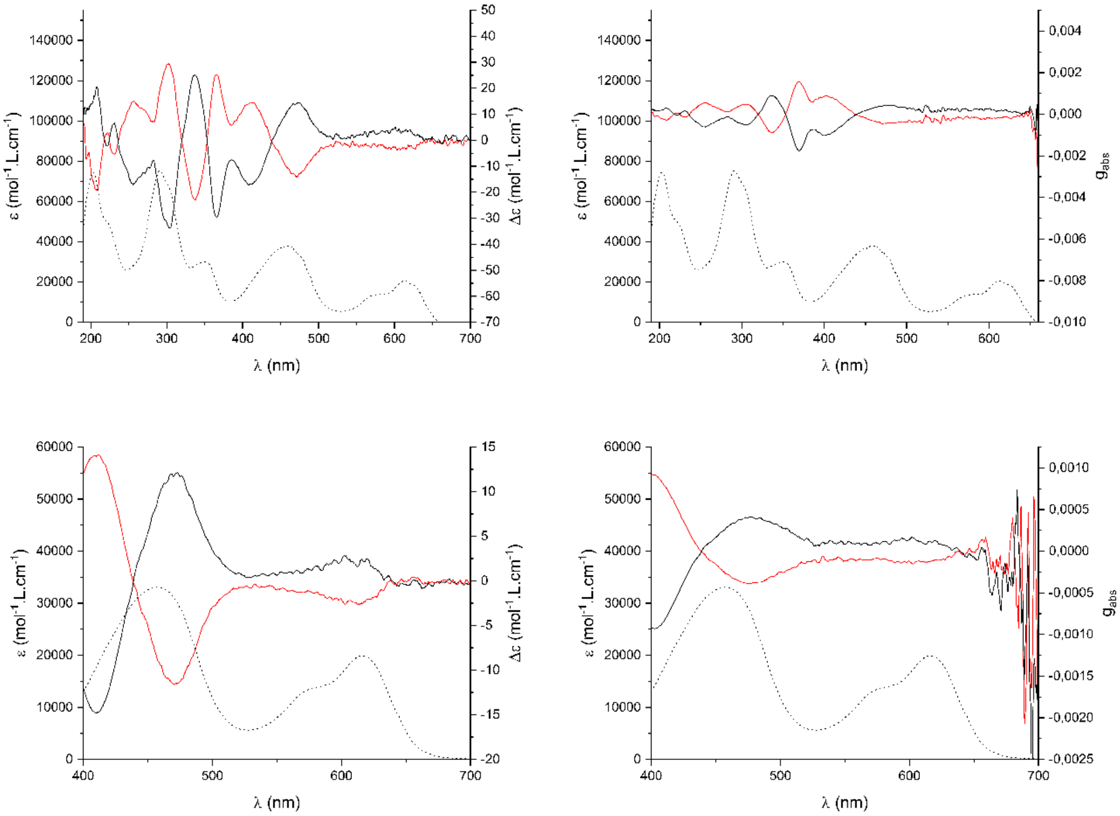

Figure S147. Absorption (dotted black), circular dichroism (left, (*M*)- enantiomer in black, (*P*)- enantiomer in red) and g_{abs} (right, (*M*)- enantiomer in black, (*P*)- enantiomer in red) spectra of compounds **6n** in acetonitrile. Top: entire spectra, bottom: zoom between 400-700 nm.

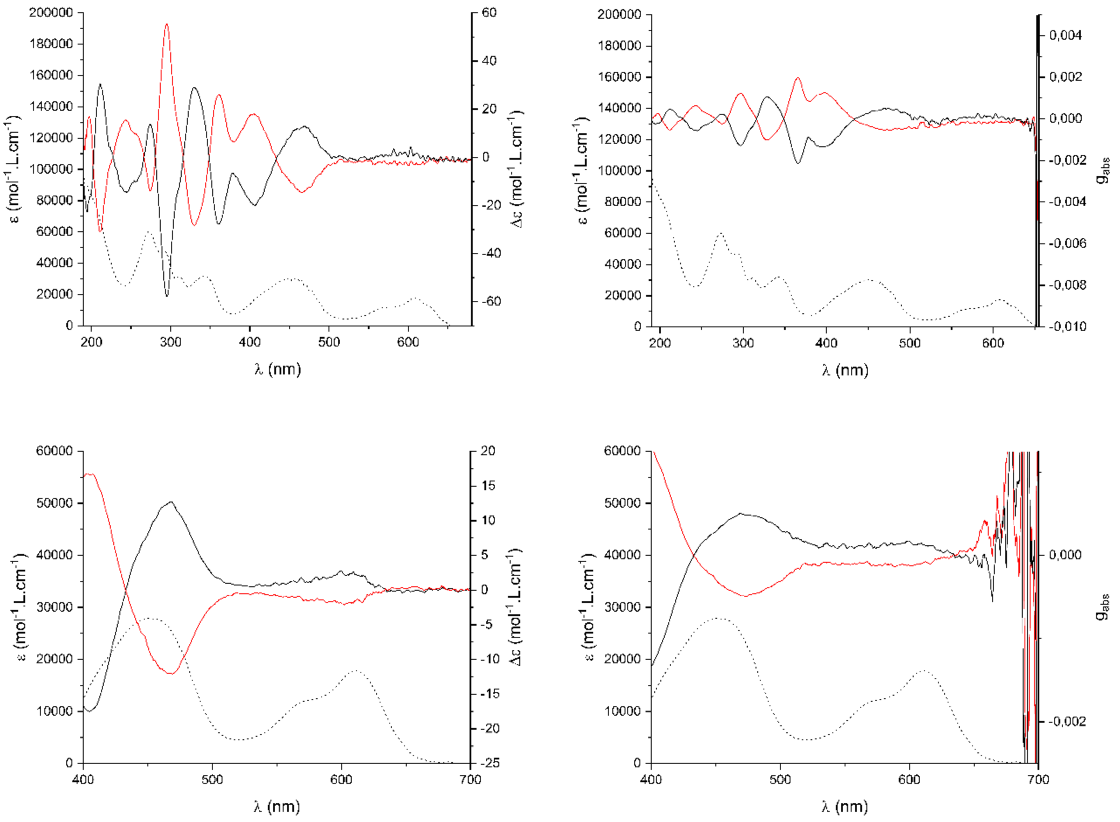

Figure S148. Absorption (dotted black), circular dichroism (left, (*M*)- enantiomer in black, (*P*)- enantiomer in red) and g_{abs} (right, (*M*)- enantiomer in black, (*P*)- enantiomer in red) spectra of compounds **6o** in acetonitrile. Top: entire spectra, bottom: zoom between 400-700 nm.

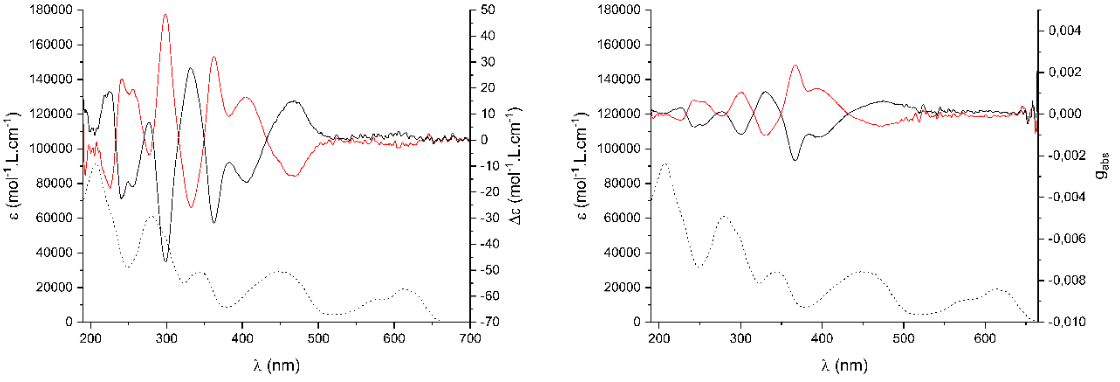

Figure S149. Absorption (dotted black), circular dichroism (left, (*M*)- enantiomer in black, (*P*)- enantiomer in red) and g_{abs} (right, (*M*)- enantiomer in black, (*P*)- enantiomer in red) spectra of compounds **6p** in acetonitrile.

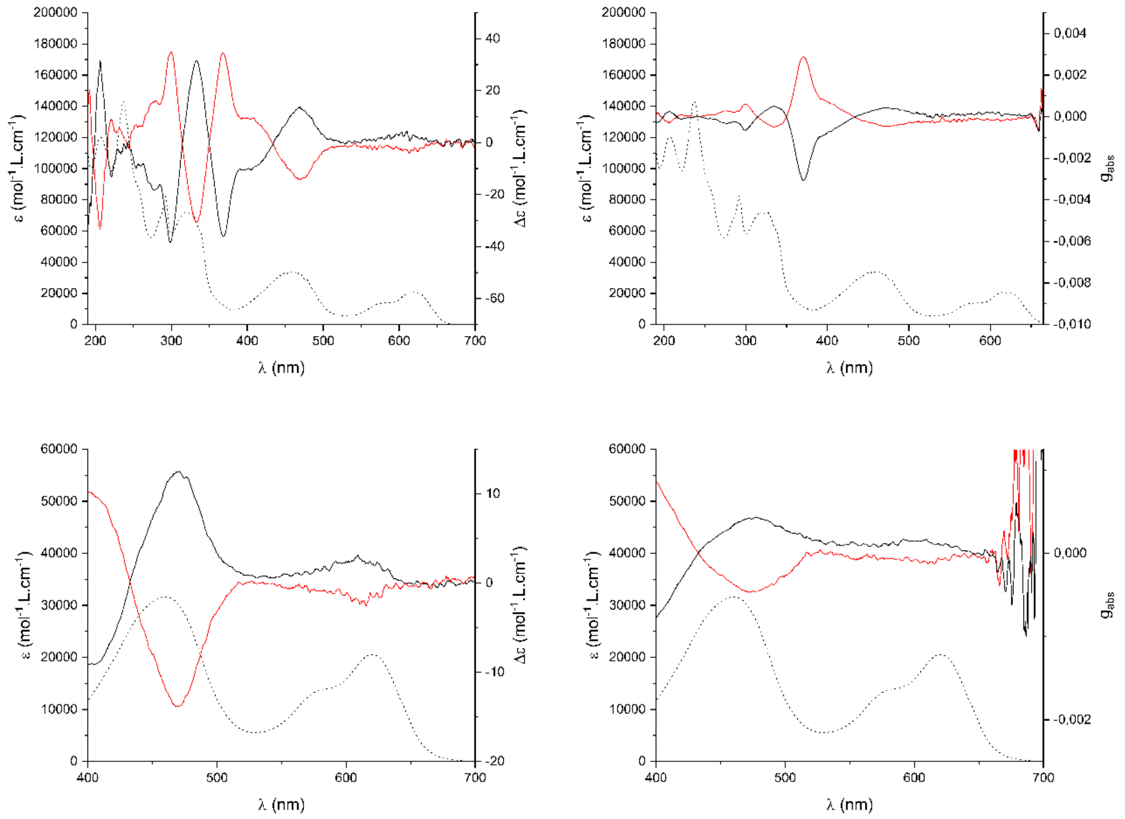

Figure S150. Absorption (dotted black), circular dichroism (left, (*M*)- enantiomer in black, (*P*)- enantiomer in red) and g_{abs} (right, (*M*)- enantiomer in black, (*P*)- enantiomer in red) spectra of compounds **6q** in acetonitrile. Top: entire spectra, bottom: zoom between 400-700 nm.

Additional spectra

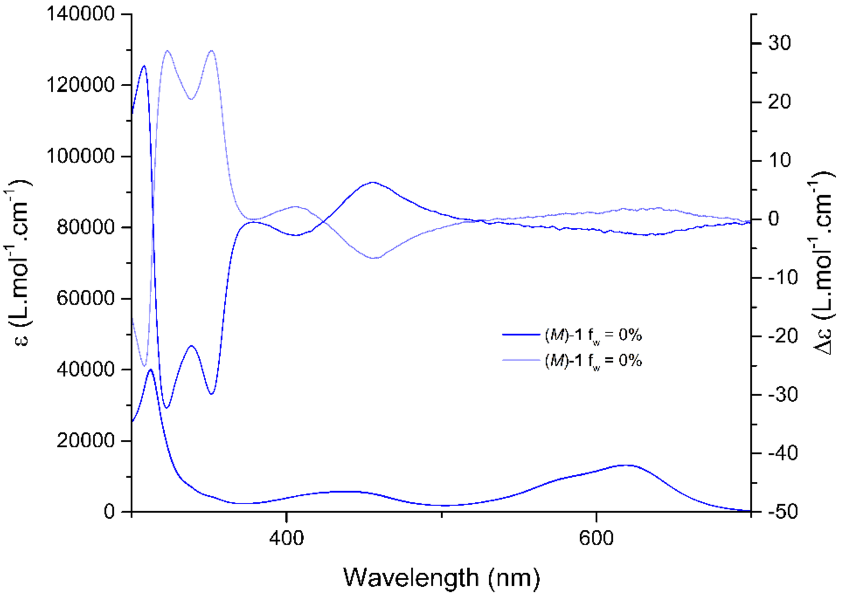

Figure S151. Absorption (bottom part of the figure) and circular dichroism (top part of the figure) of compounds **1** in acetonitrile.

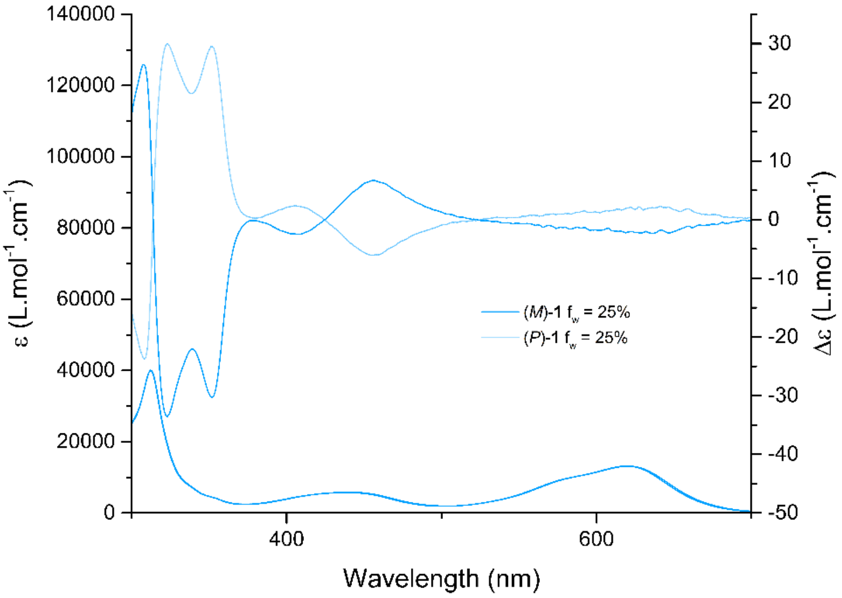

Figure S152. Absorption (bottom part of the figure) and circular dichroism (top part of the figure) of compounds **1** in 25/75 acetonitrile/water solution.

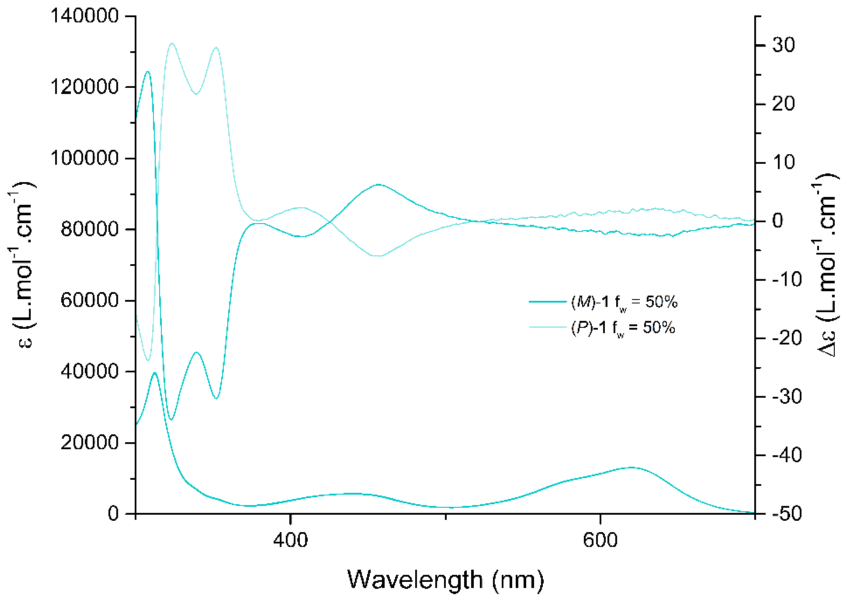

Figure S153. Absorption (bottom part of the figure) and circular dichroism (top part of the figure) of compounds **1** in 50/50 acetonitrile/water solution.

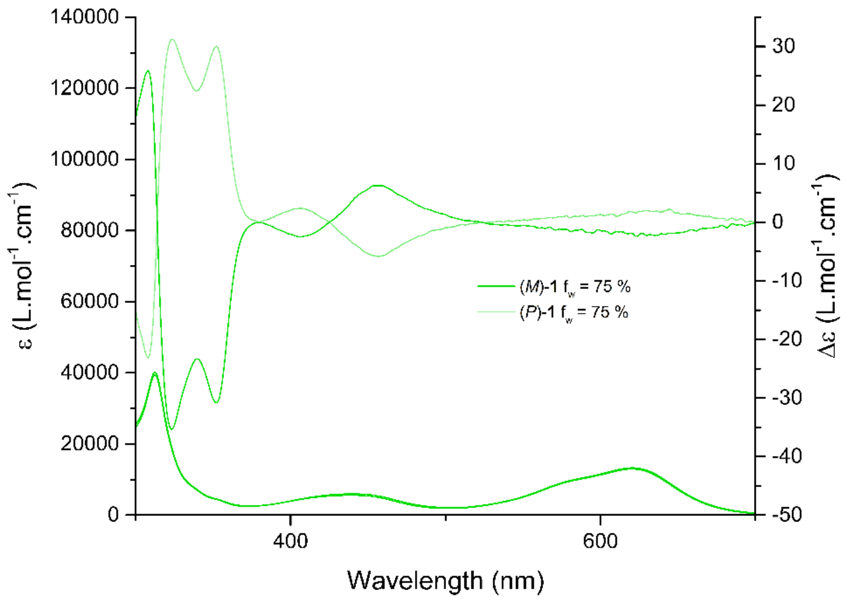

Figure S154. Absorption (bottom part of the figure) and circular dichroism (top part of the figure) of compounds **1** in 25/75 acetonitrile/water solution.

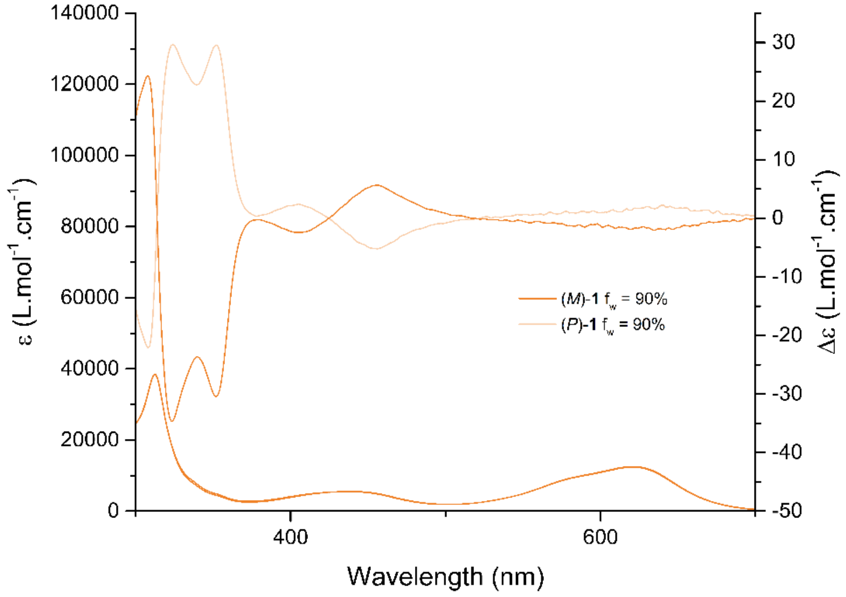

Figure S155. Absorption (bottom part of the figure) and circular dichroism (top part of the figure) of compounds **1** in 10/90 acetonitrile/water solution.

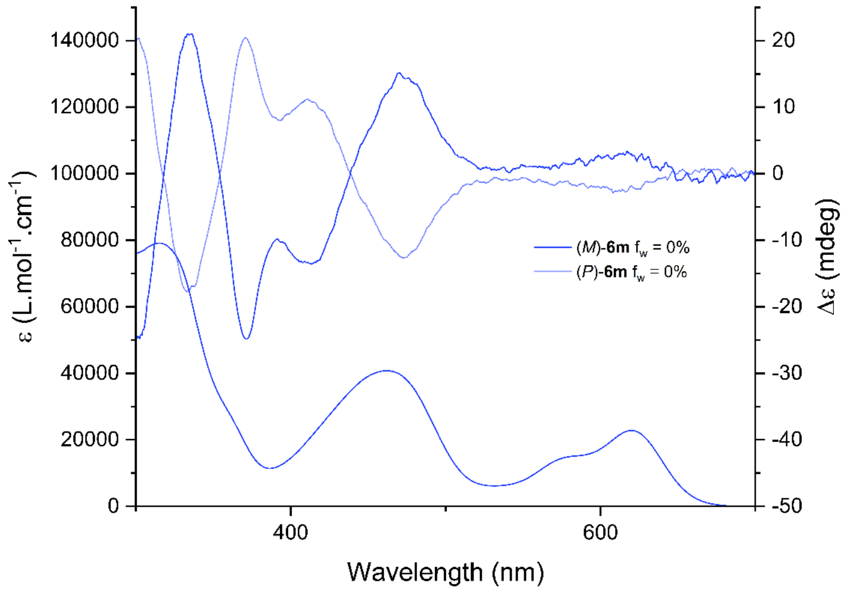

Figure S156. Absorption (bottom part of the figure) and circular dichroism (top part of the figure) of compounds **6m** acetonitrile solution.

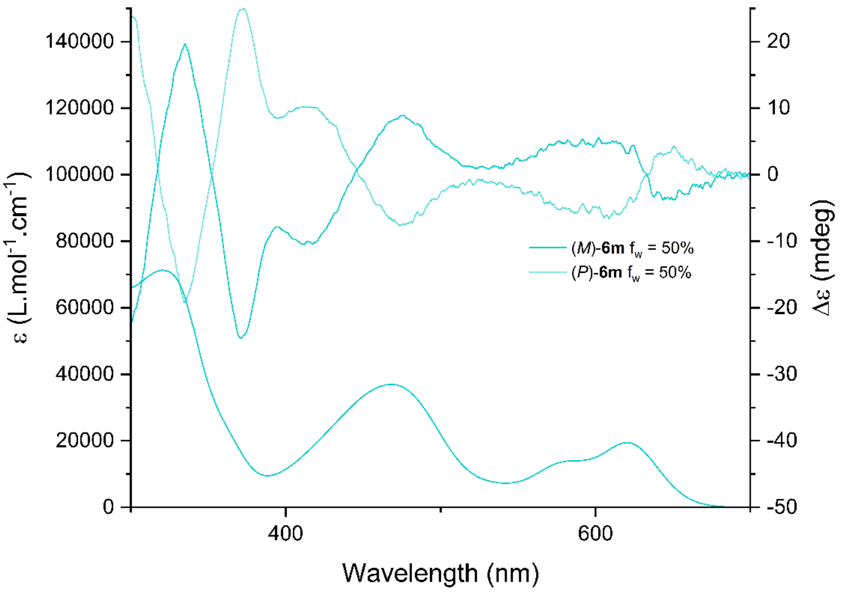

Figure S157. Absorption (bottom part of the figure) and circular dichroism (top part of the figure) of compounds **6m** in 50/50 acetonitrile/water solution.

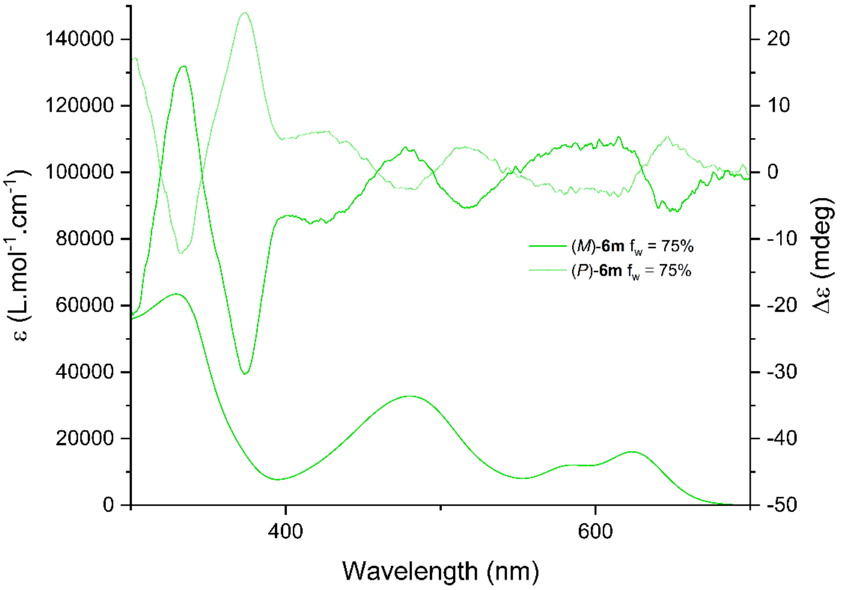

Figure S158. Absorption (bottom part of the figure) and circular dichroism (top part of the figure) of compounds **6m** in 25/75 acetonitrile/water solution.

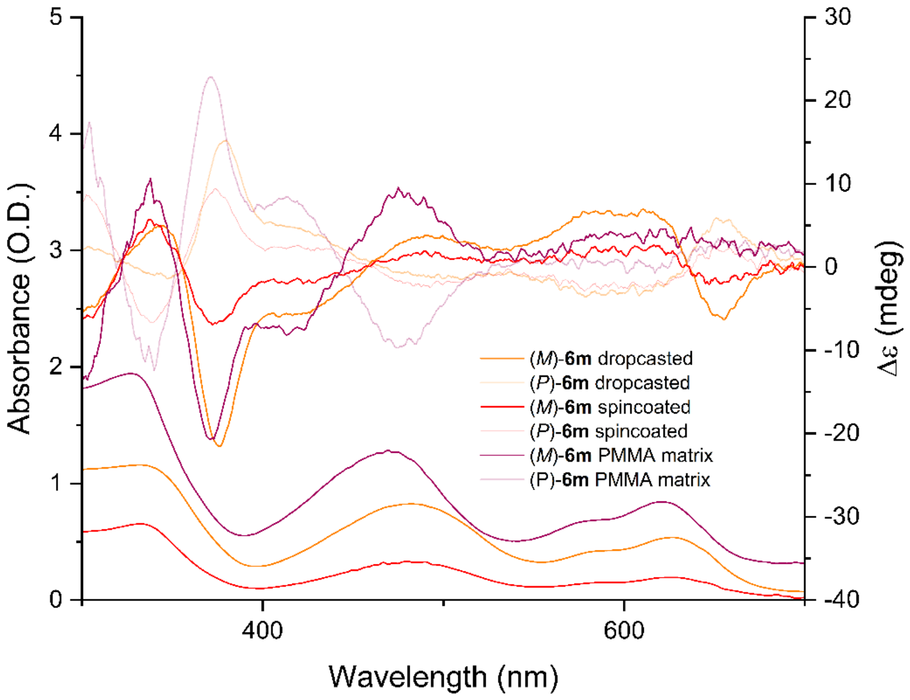

Figure S159. Absorption (bottom part of the figure) and circular dichroism (top part of the figure) of compounds **6m** in solid state on glass.

Circularly Polarized Luminescence and g_{lum}

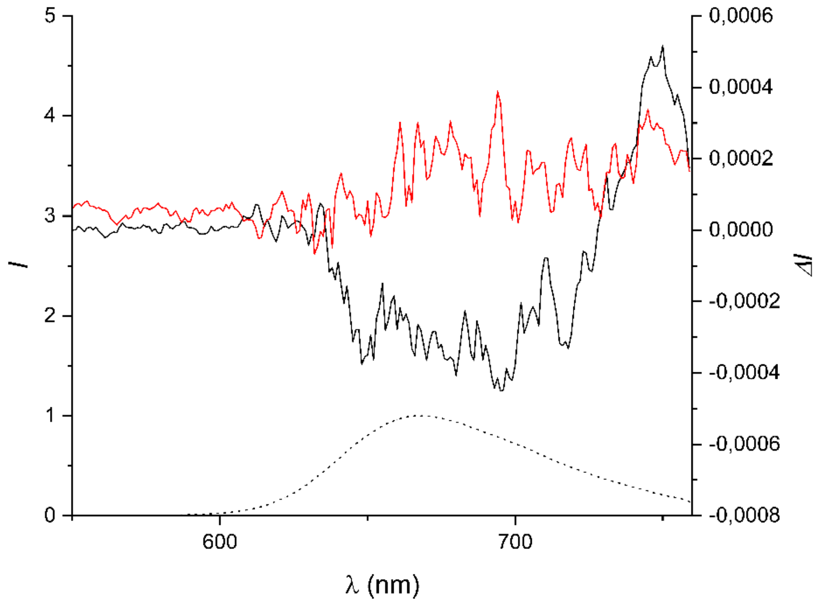

Figure S160. Fluorescence (dotted black) and CPL spectra (*M*)- enantiomer in black, (*P*)- enantiomer in red) of compounds **1** in acetonitrile.

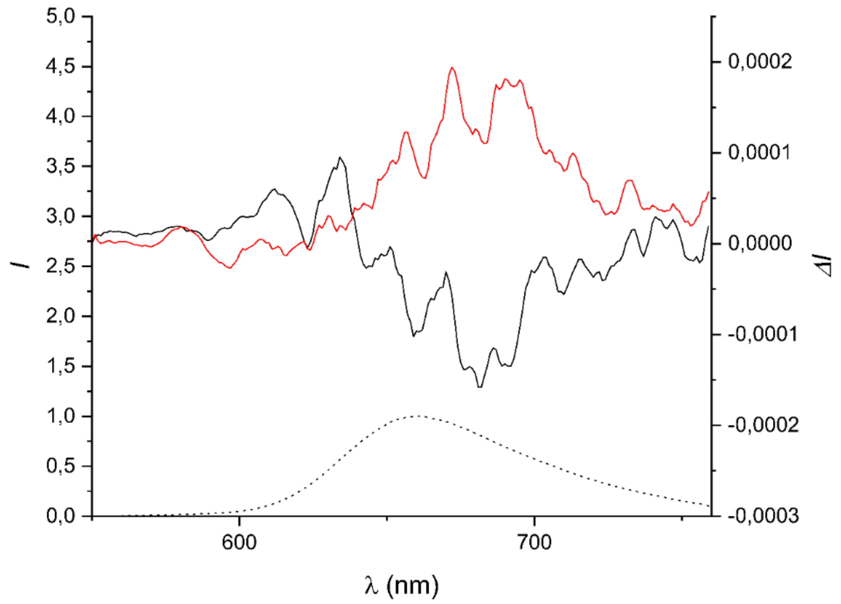

Figure S161. Fluorescence (dotted black) and CPL spectra ((*M*)- enantiomer in black, (*P*)- enantiomer in red) of compounds **6a** in acetonitrile.

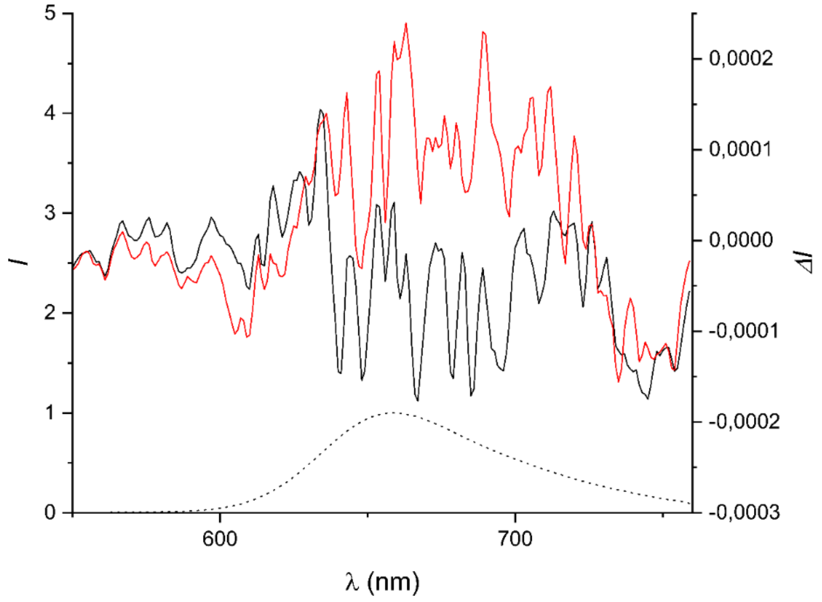

Figure S162. Fluorescence (dotted black) and CPL spectra ((*M*)- enantiomer in black, (*P*)- enantiomer in red) of compounds **6b** in acetonitrile.

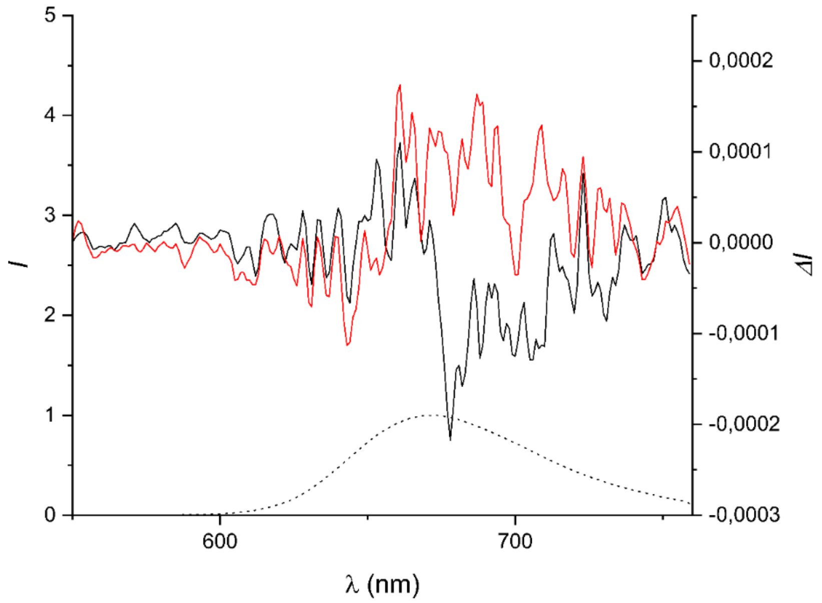

Figure S163. Fluorescence (dotted black) and CPL spectra ((*M*)- enantiomer in black, (*P*)- enantiomer in red) of compounds **6e** in acetonitrile.

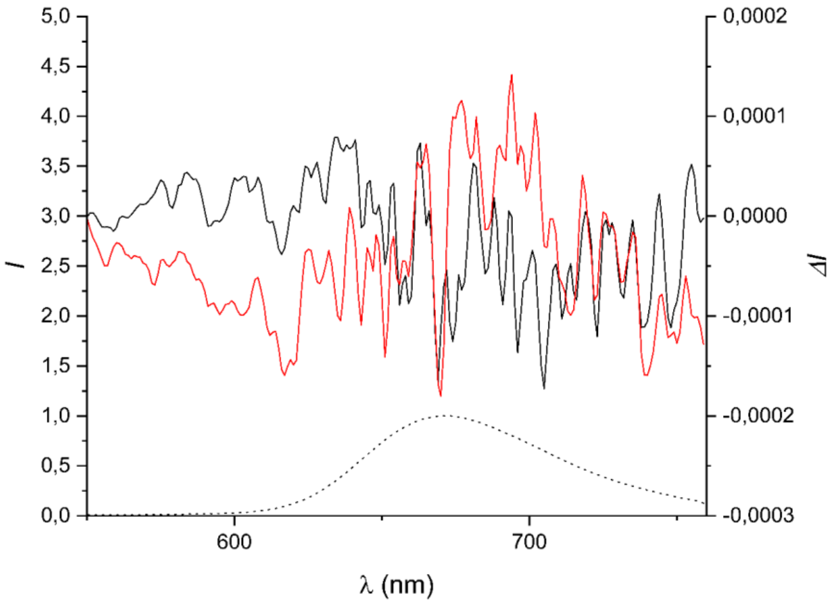

Figure S164. Fluorescence (dotted black) and CPL spectra ((*M*)- enantiomer in black, (*P*)- enantiomer in red) of compounds **6g** in acetonitrile.

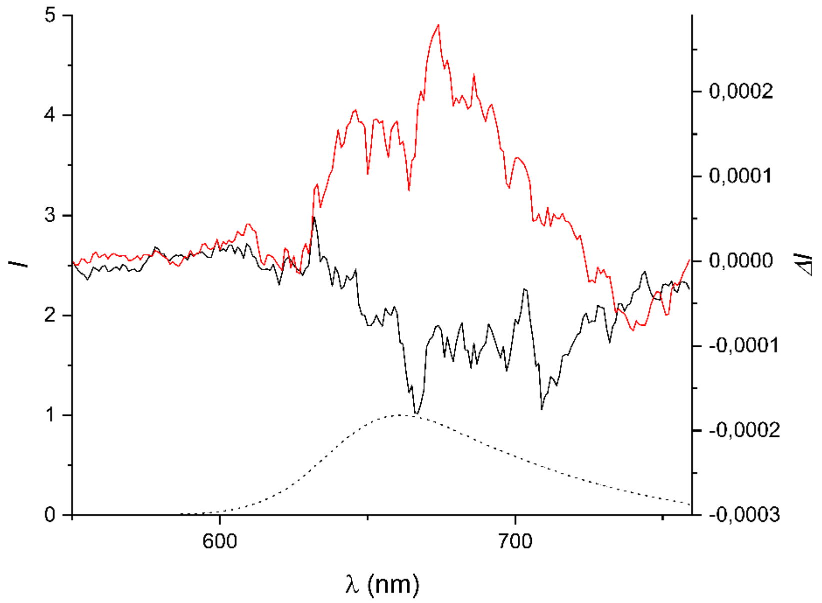

Figure S165. Fluorescence (dotted black) and CPL spectra ((*M*)- enantiomer in black, (*P*)- enantiomer in red) of compounds **6m** in acetonitrile.

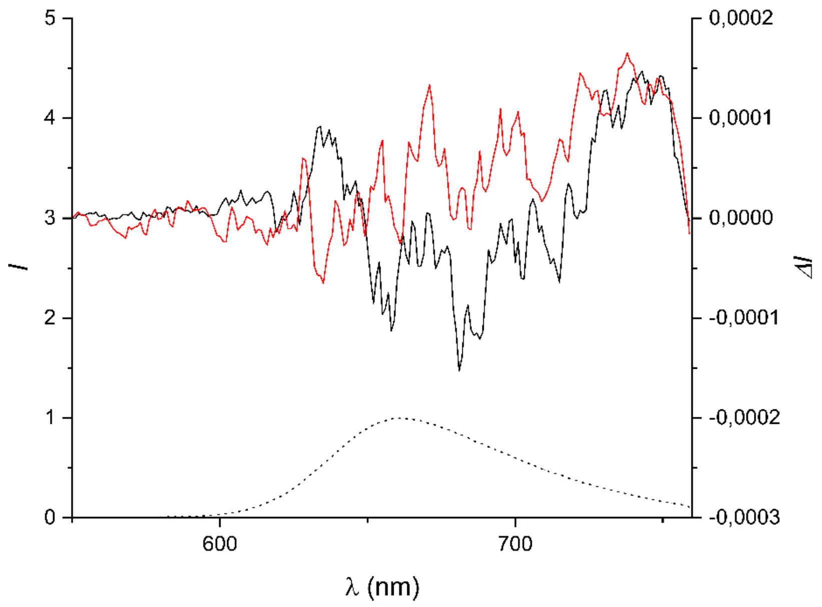

Figure S166. Fluorescence (dotted black) and CPL spectra ((*M*)- enantiomer in black, (*P*)- enantiomer in red) of compounds **6m** in acetonitrile.

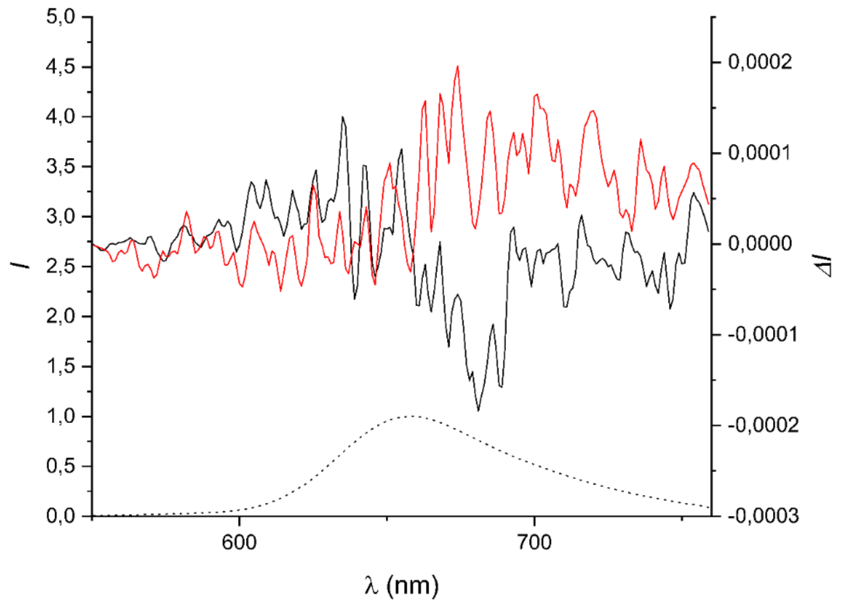

Figure S167. Fluorescence (dotted black) and CPL spectra ((*M*)- enantiomer in black, (*P*)- enantiomer in red) of compounds **6n** in acetonitrile.

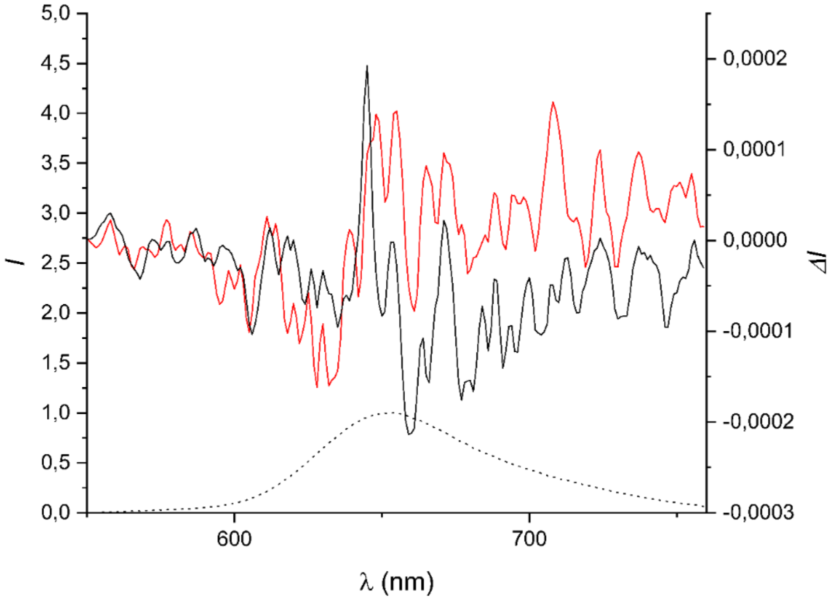

Figure S168. Fluorescence (dotted black) and CPL spectra ((*M*)- enantiomer in black, (*P*)- enantiomer in red) of compounds **6o** in acetonitrile.

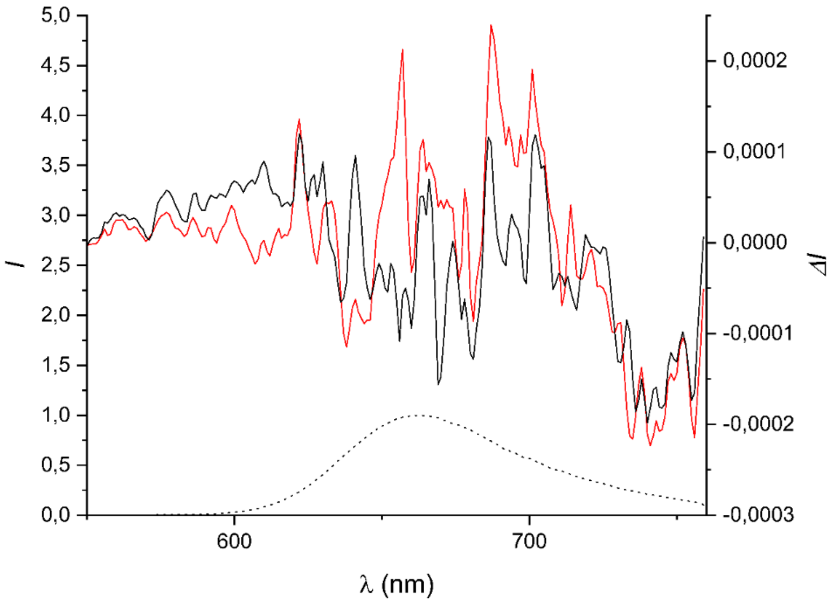

Figure S169. Fluorescence (dotted black) and CPL spectra ((*M*)- enantiomer in black, (*P*)- enantiomer in red) of compounds **6q** in acetonitrile.

Fluorescence lifetime

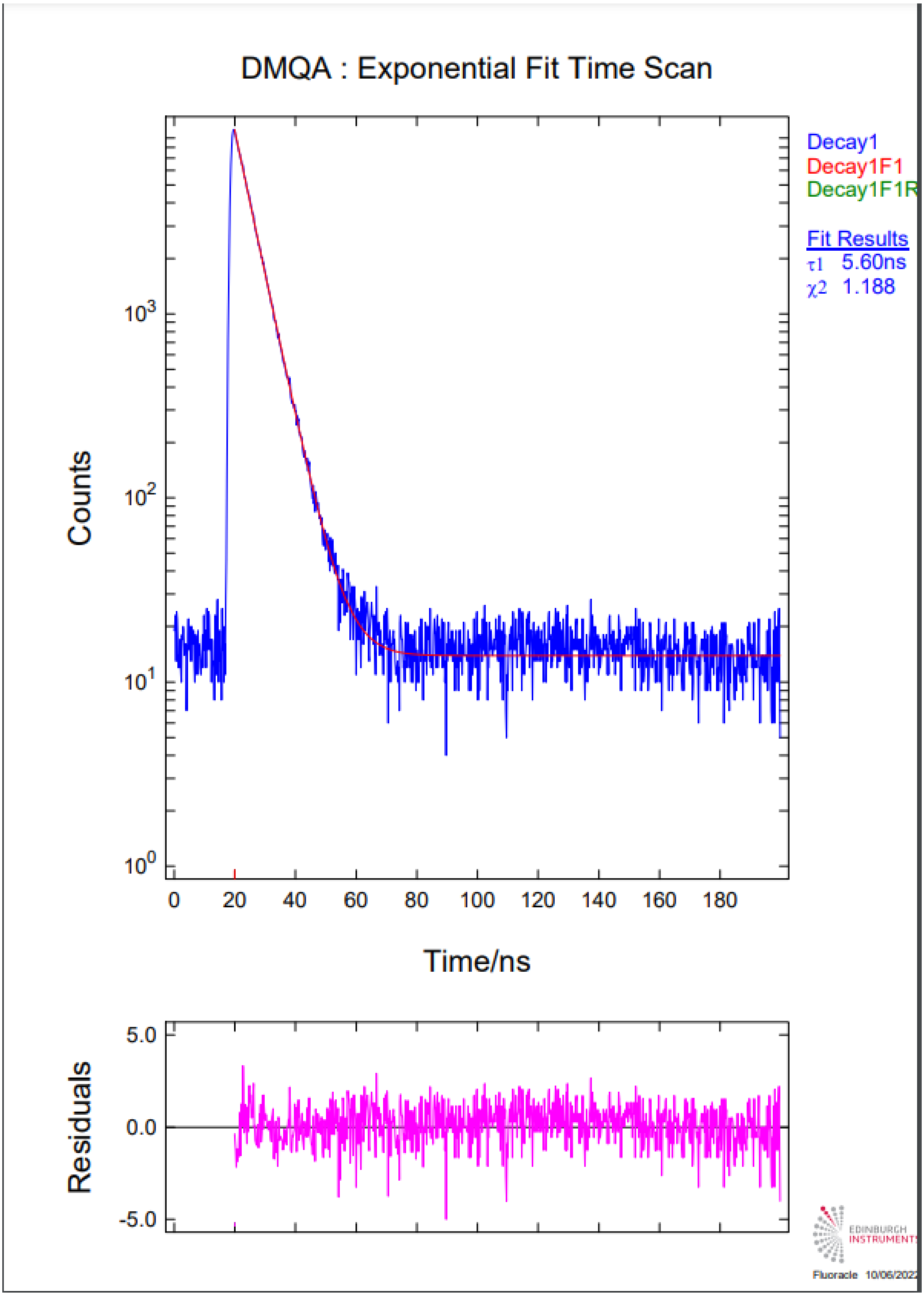

Figure S170. Fluorescence (experimental data in blue, fit in red, residue in pink) of compounds **1** in acetonitrile.

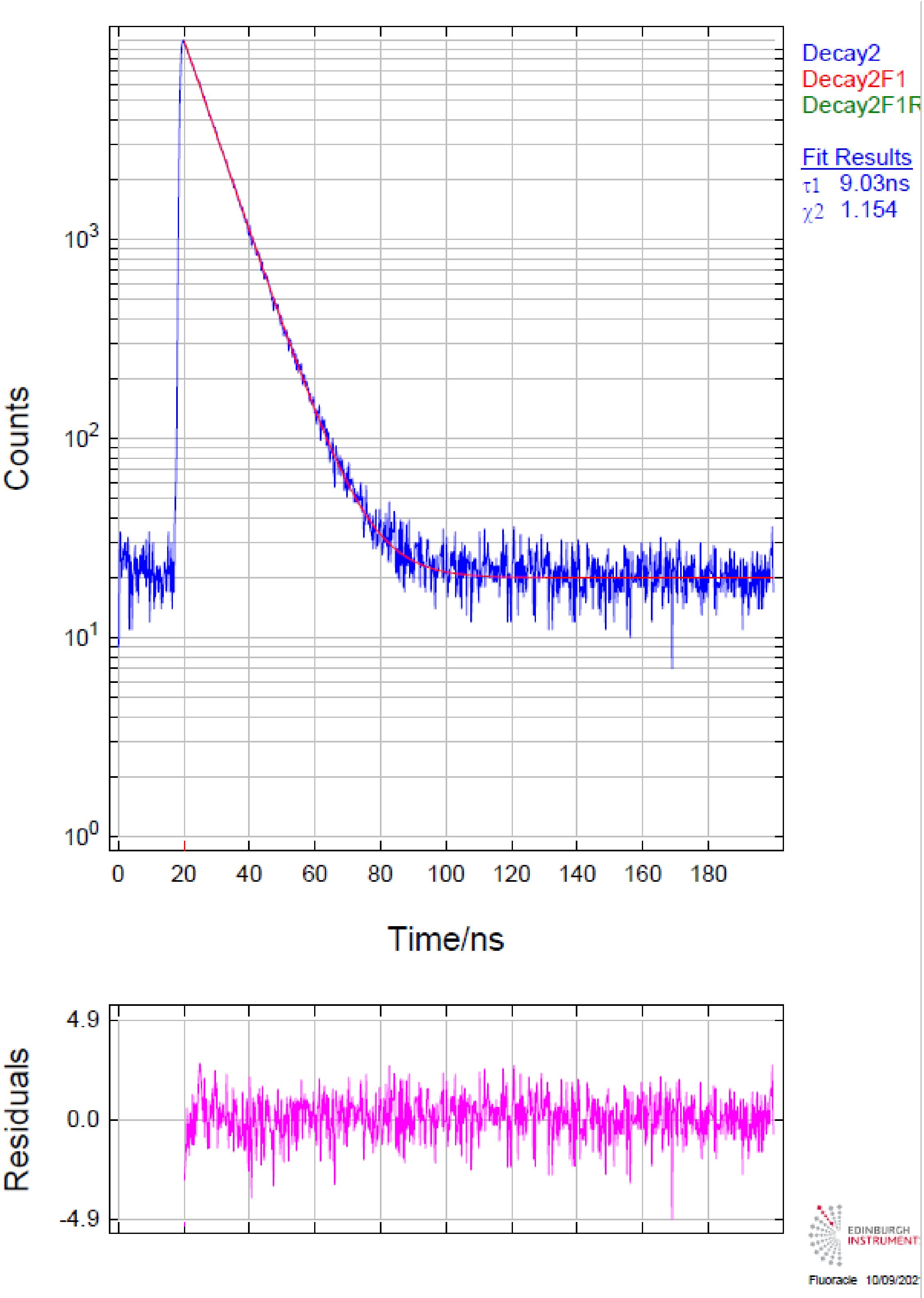

Figure S171. Fluorescence (experimental data in blue, fit in red, residue in pink) of compounds **6a** in acetonitrile.

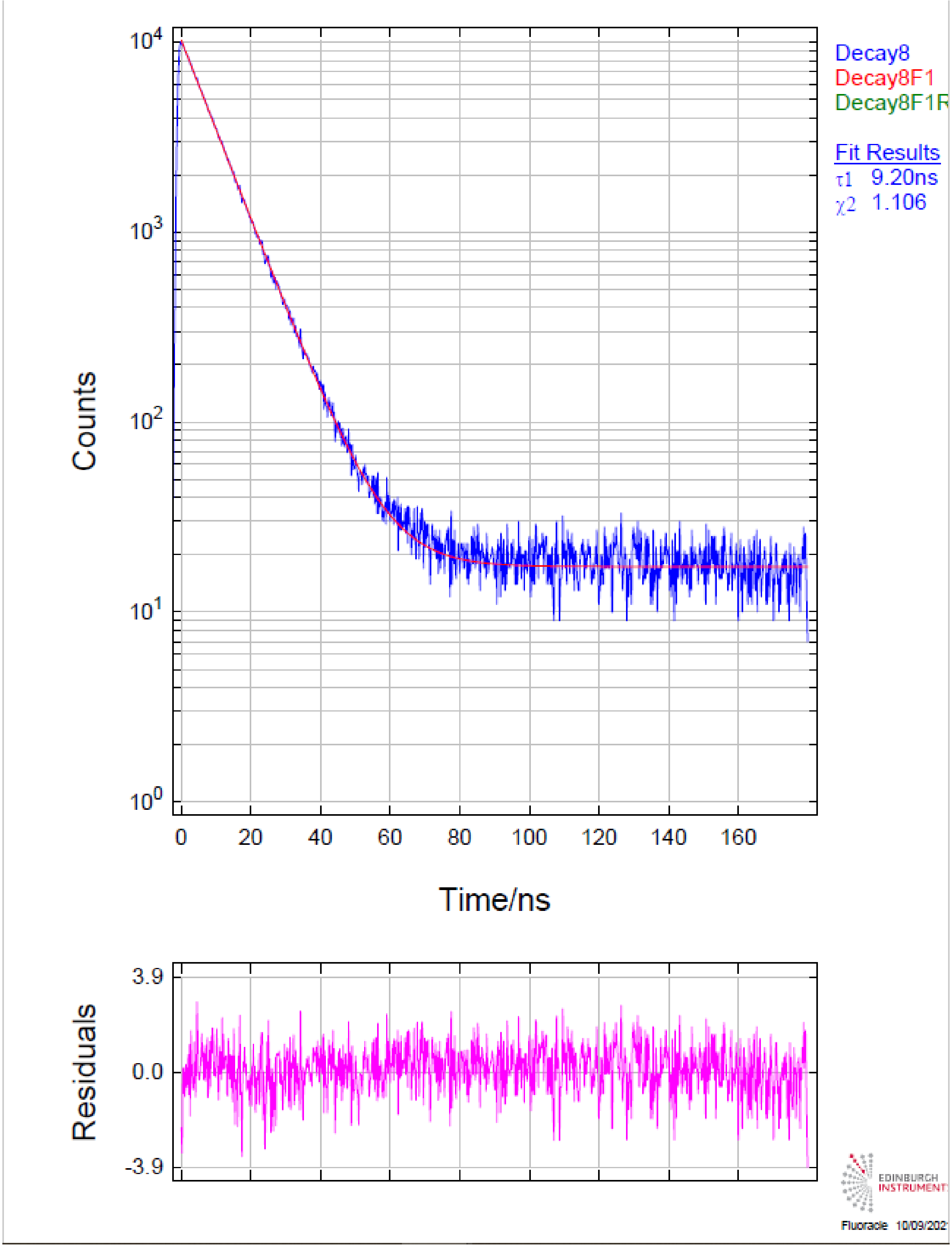

Figure S172. Fluorescence (experimental data in blue, fit in red, residue in pink) of compounds **6b** in acetonitrile.

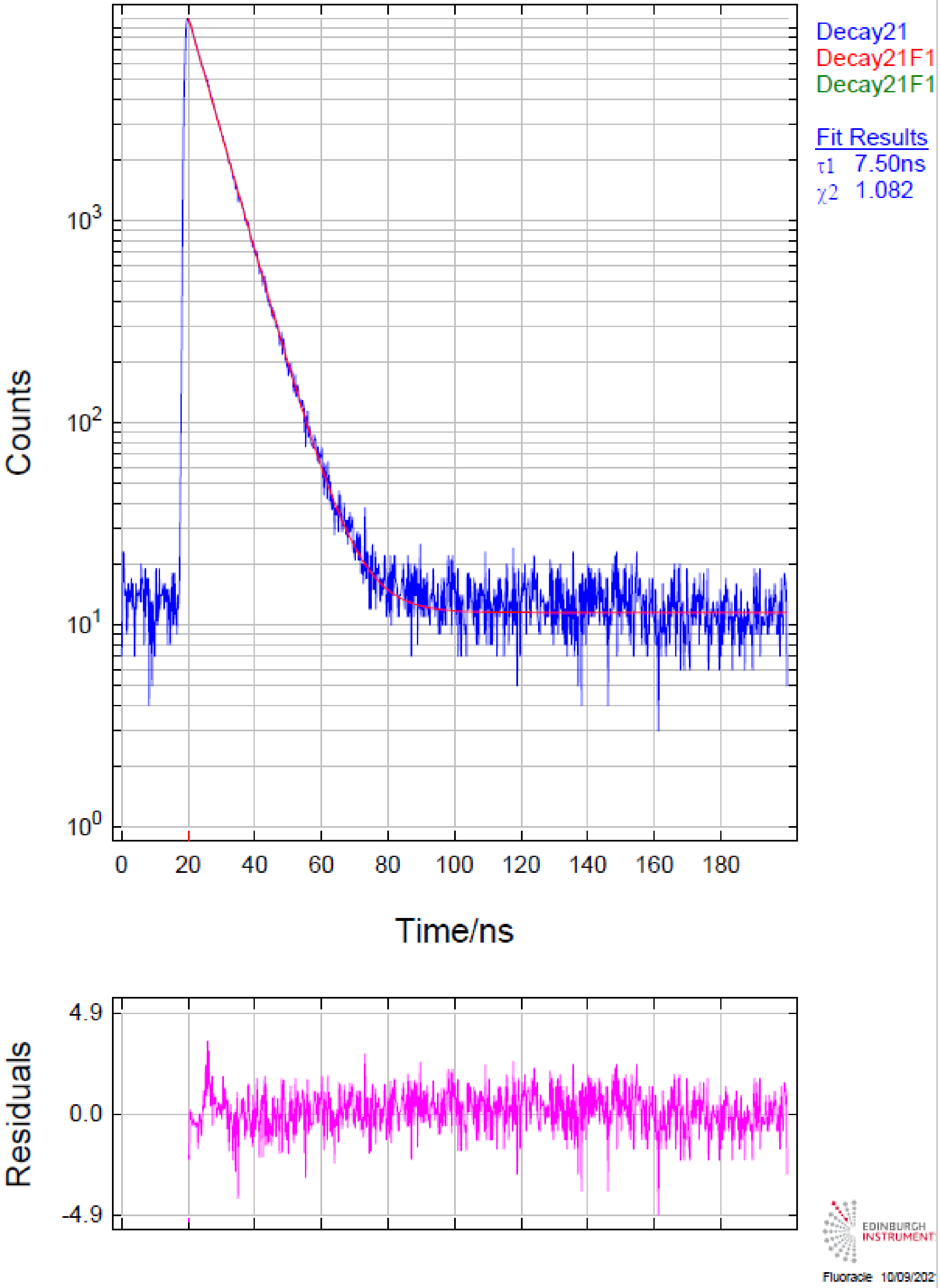

Figure S173. Fluorescence (experimental data in blue, fit in red, residue in pink) of compounds **6c** in acetonitrile.

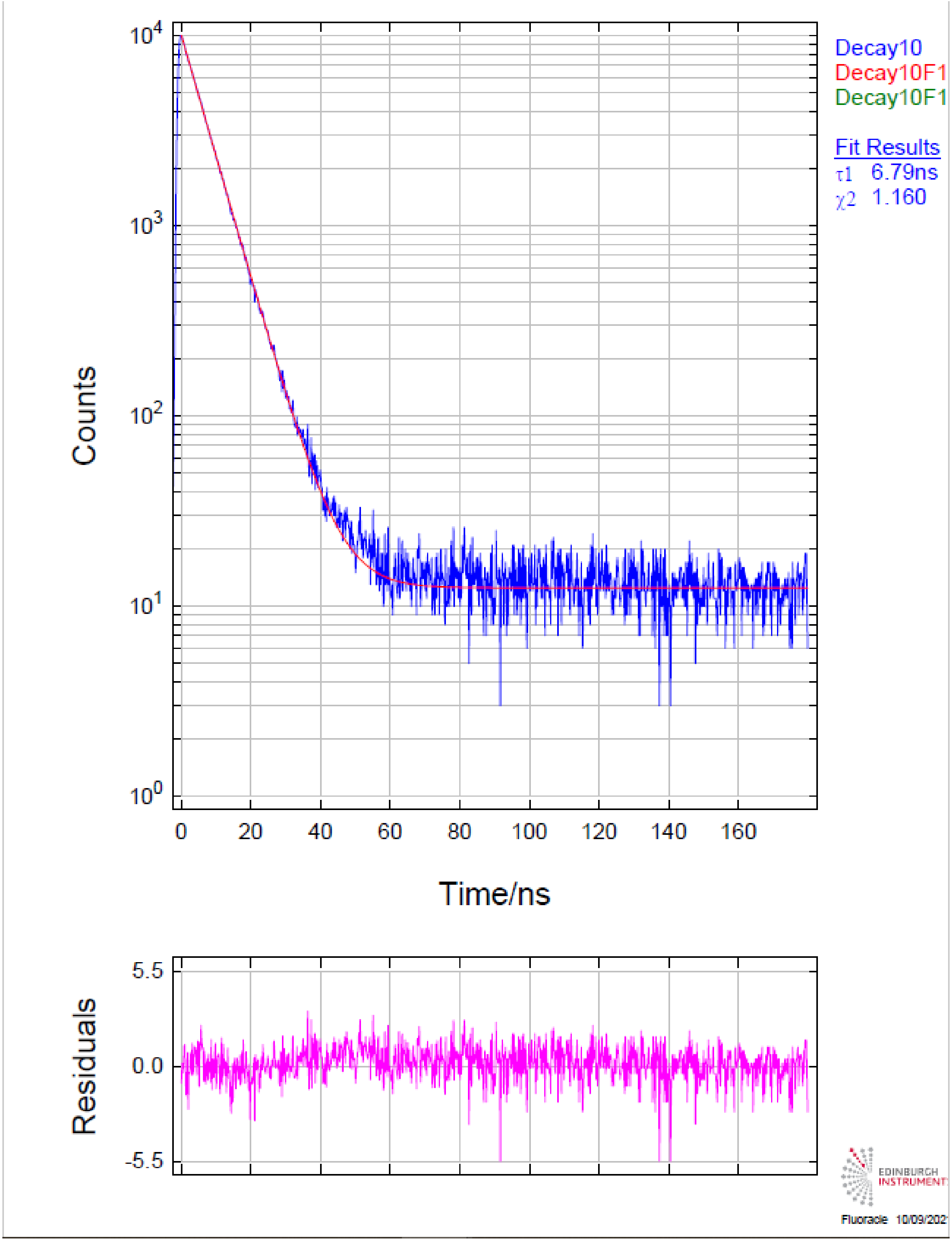

Figure S174. Fluorescence (experimental data in blue, fit in red, residue in pink) of compounds **6d** in acetonitrile.

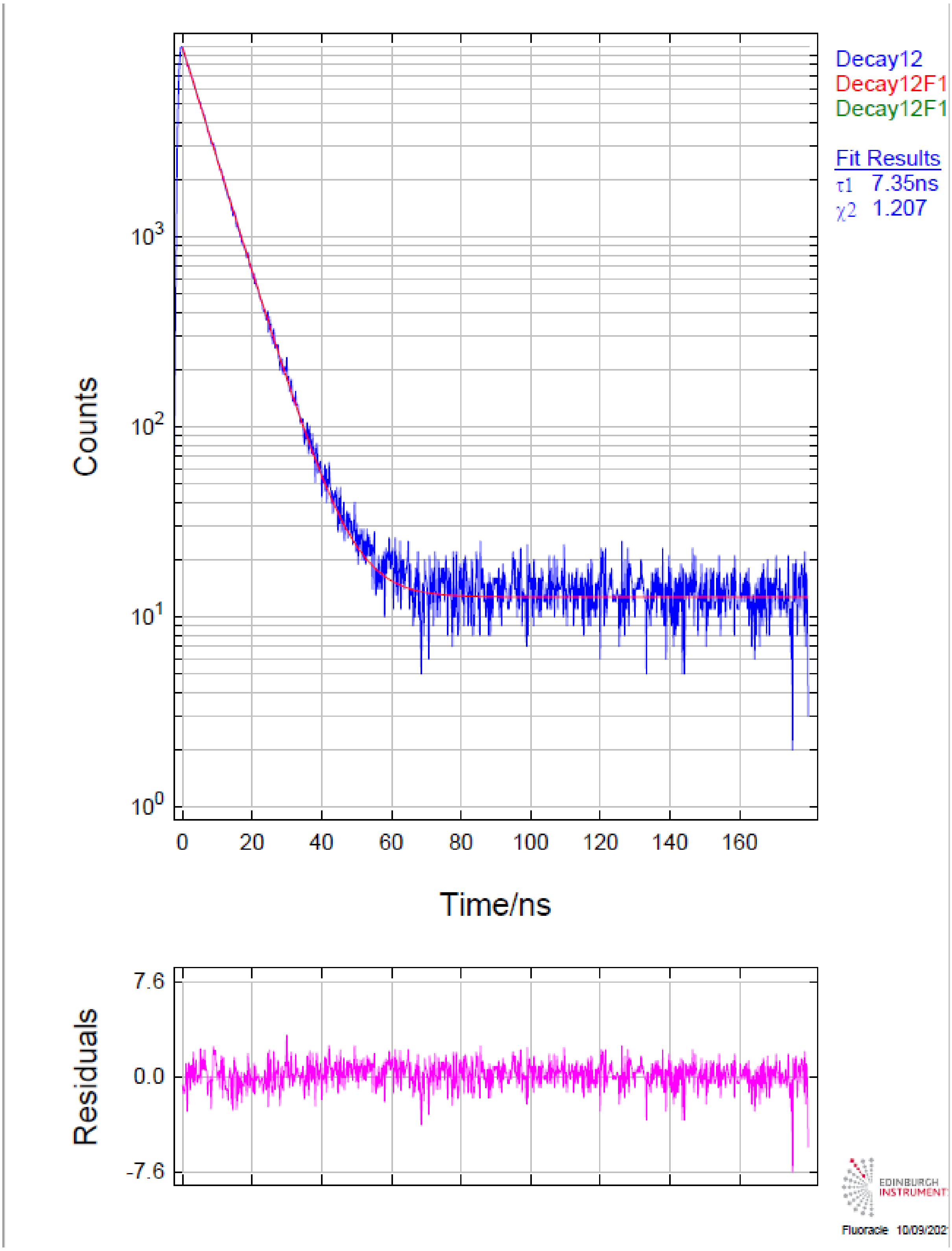

Figure S175. Fluorescence (experimental data in blue, fit in red, residue in pink) of compounds **6e** in acetonitrile.

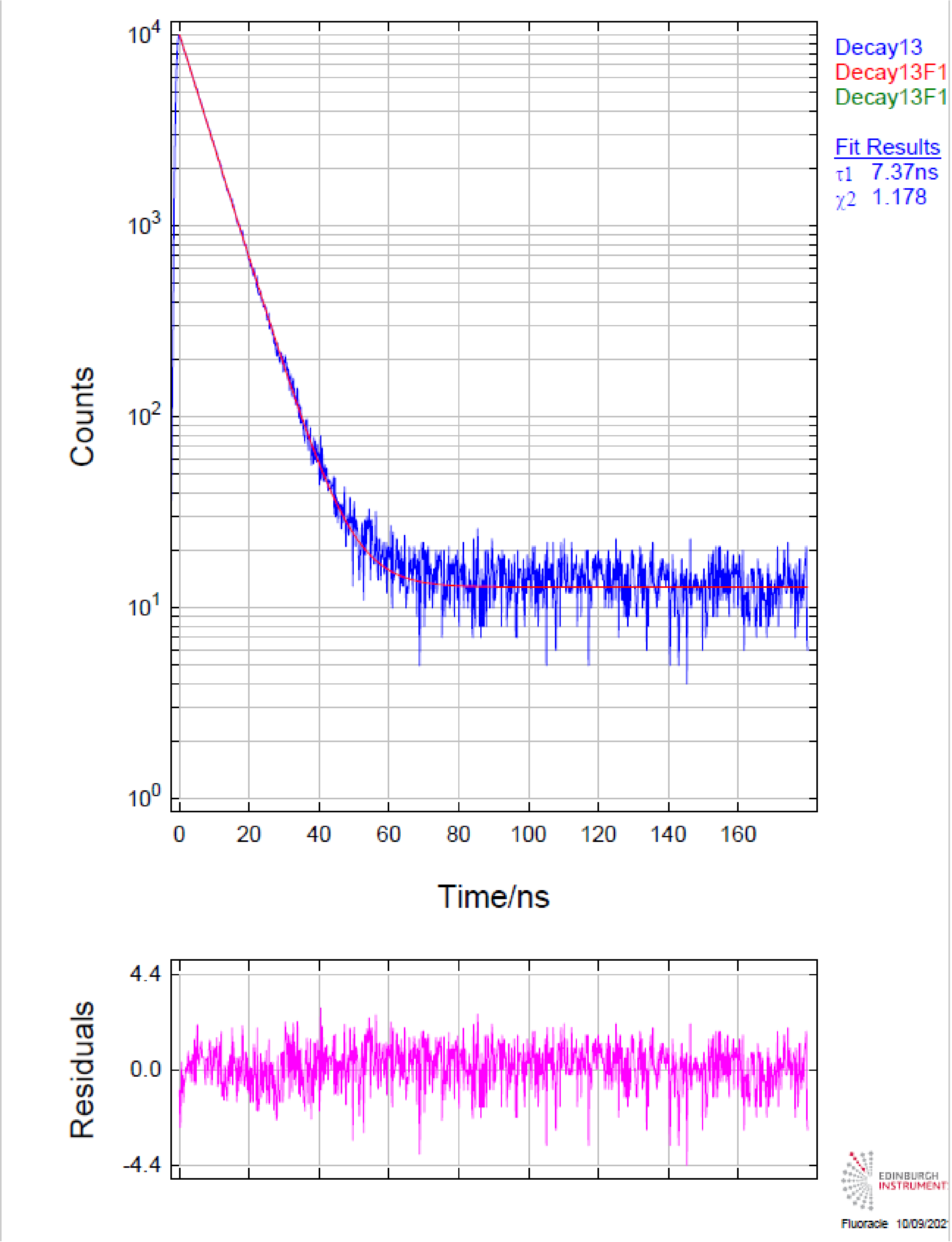

Figure S176. Fluorescence (experimental data in blue, fit in red, residue in pink) of compounds **6f** in acetonitrile.

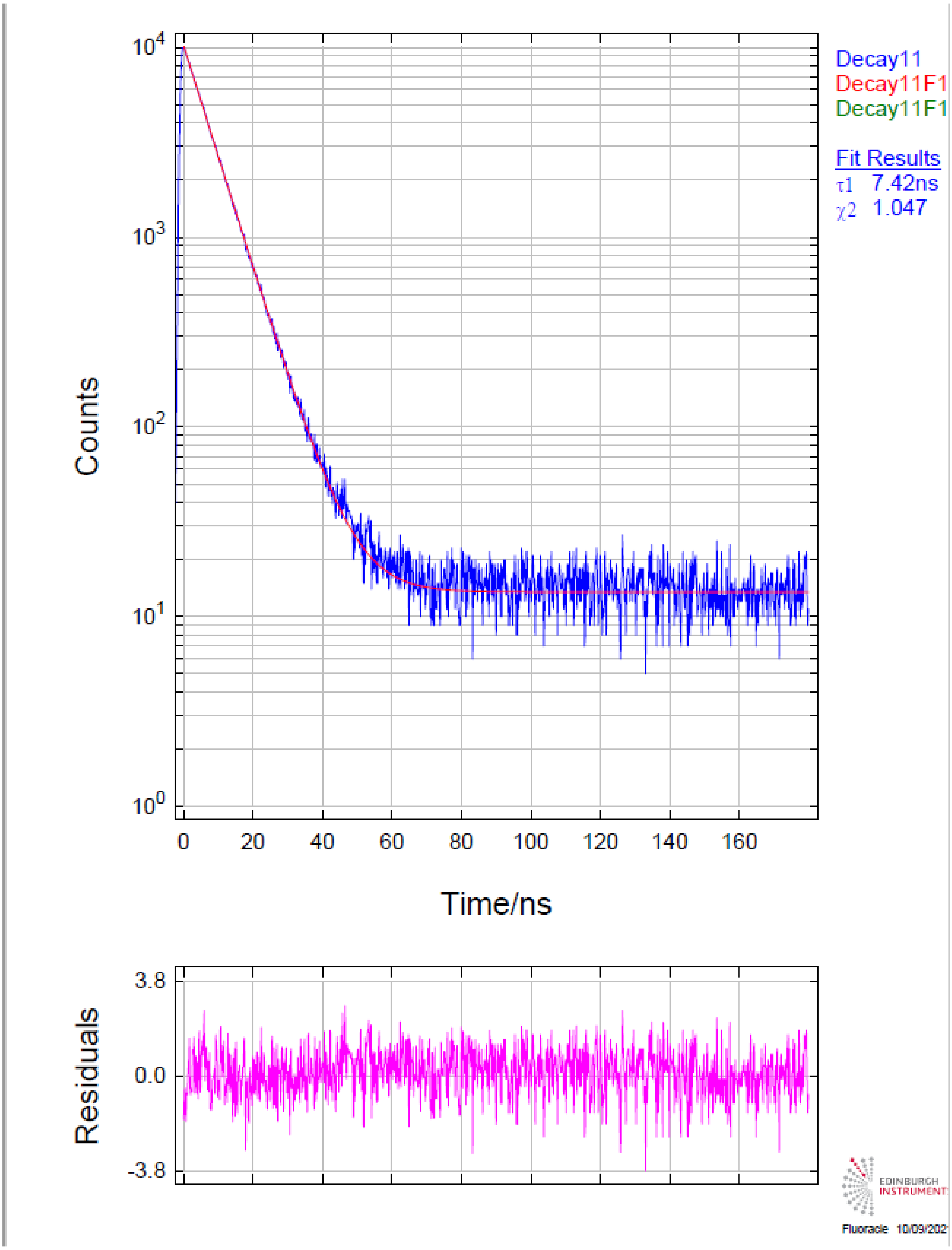

Figure S177. Fluorescence (experimental data in blue, fit in red, residue in pink) of compounds **6g** in acetonitrile.

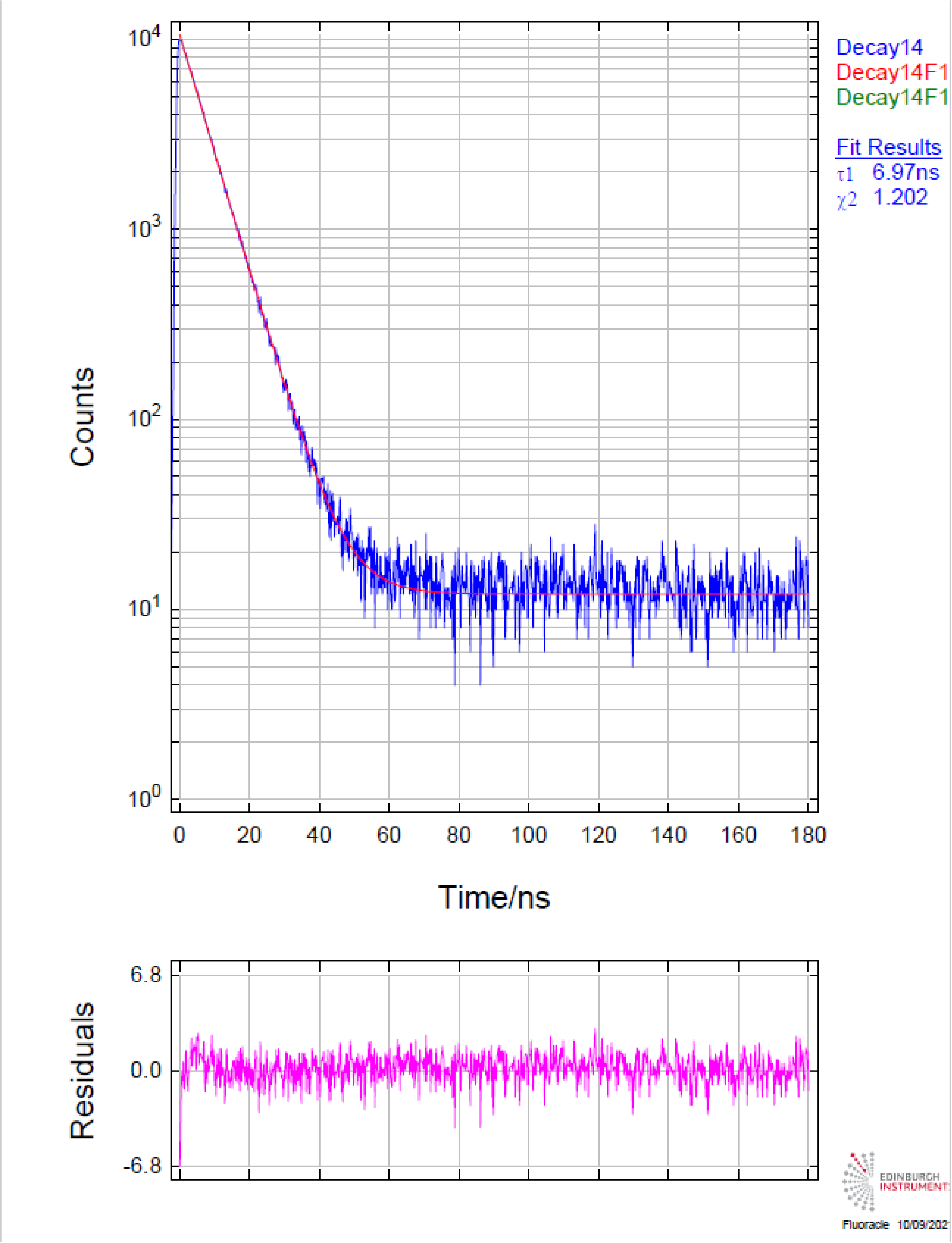

Figure S178. Fluorescence (experimental data in blue, fit in red, residue in pink) of compounds **6h** in acetonitrile.

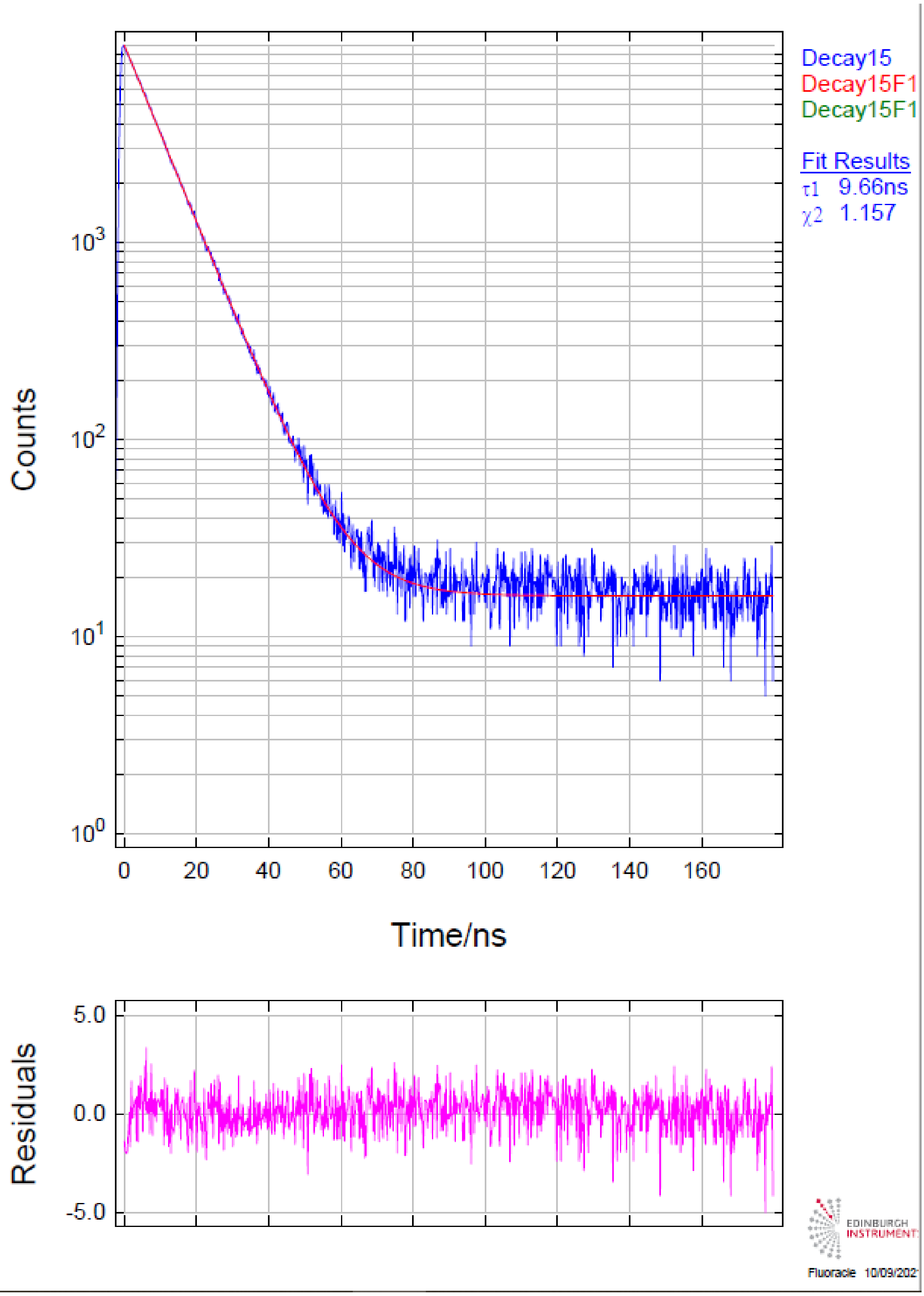

Figure S179. Fluorescence (experimental data in blue, fit in red, residue in pink) of compounds **6i** in acetonitrile.

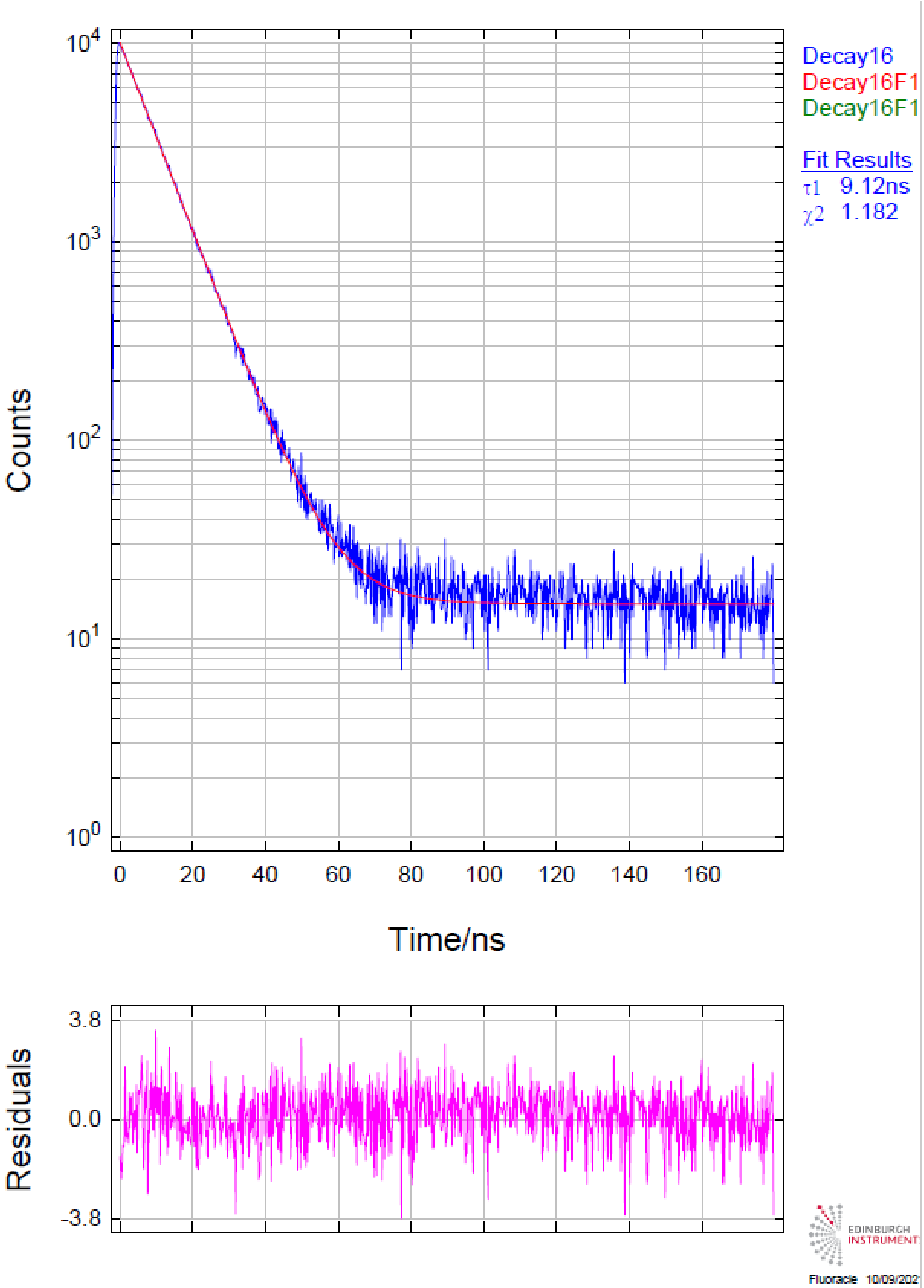

Figure S180. Fluorescence (experimental data in blue, fit in red, residue in pink) of compounds **6j** in acetonitrile.

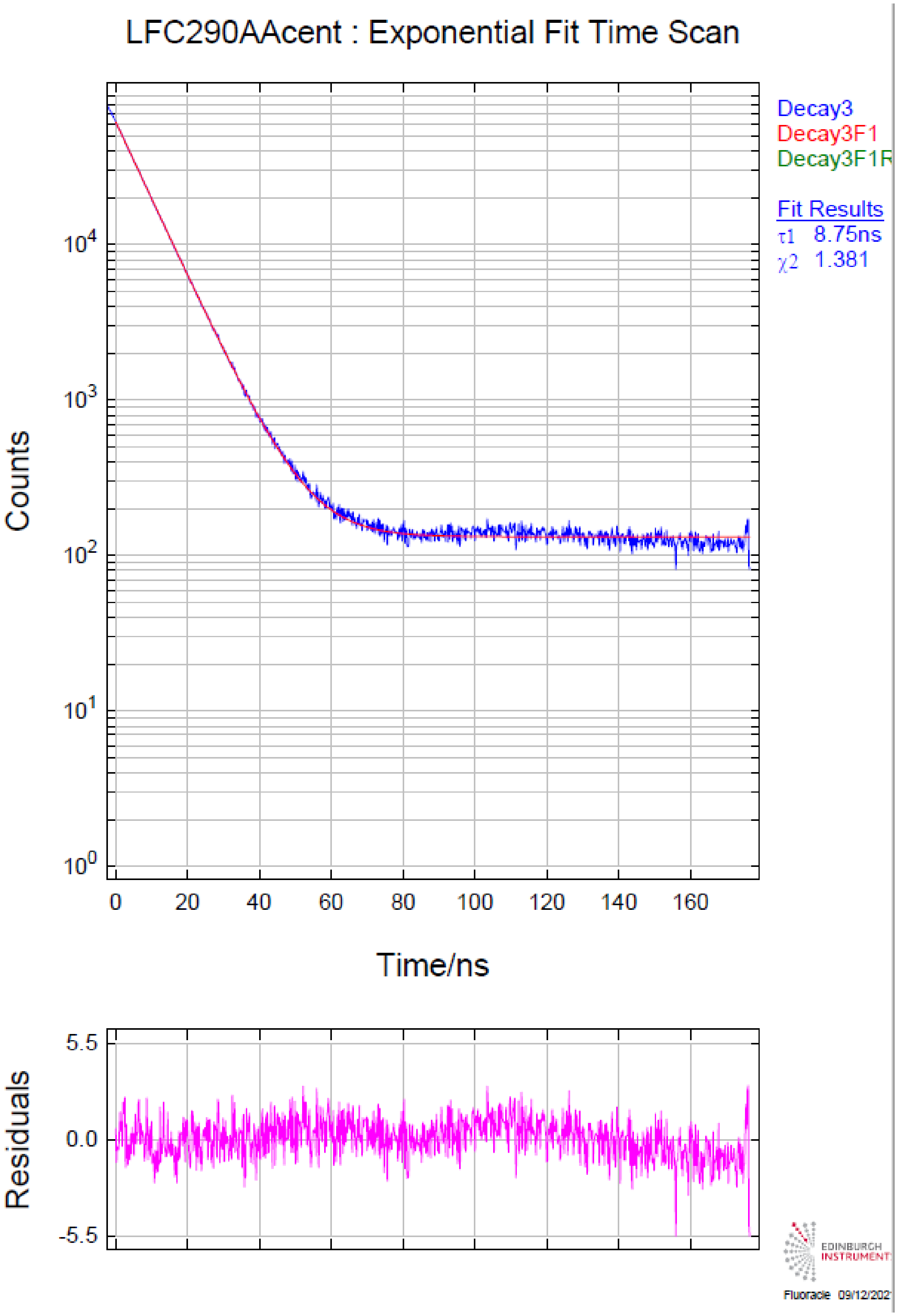

Figure S181. Fluorescence (experimental data in blue, fit in red, residue in pink) of compounds **6k** in acetonitrile.

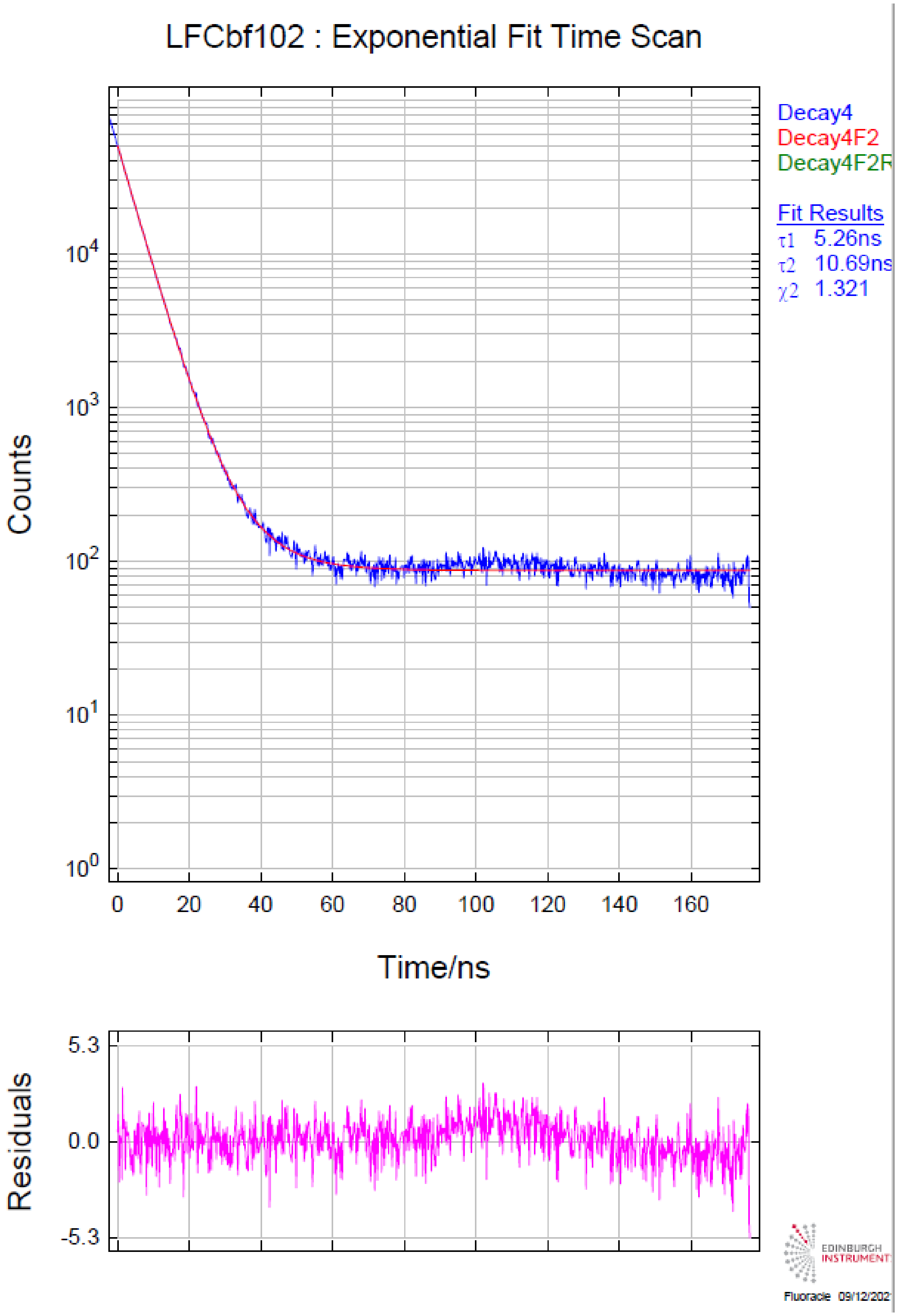

Figure S182. Fluorescence (experimental data in blue, fit in red, residue in pink) of compounds **6l** in acetonitrile.

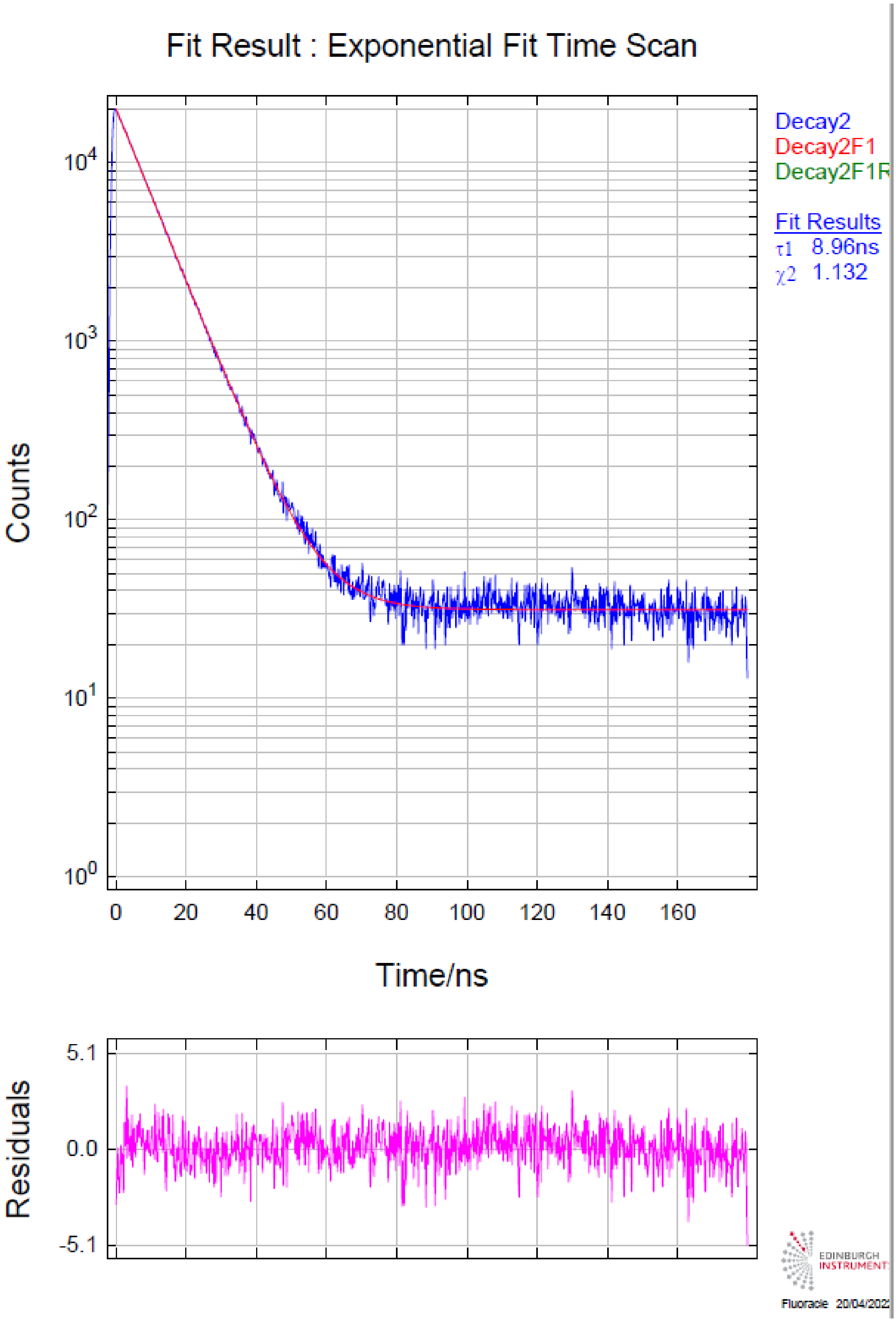

Figure S183. Fluorescence (experimental data in blue, fit in red, residue in pink) of compounds **6m** in acetonitrile.

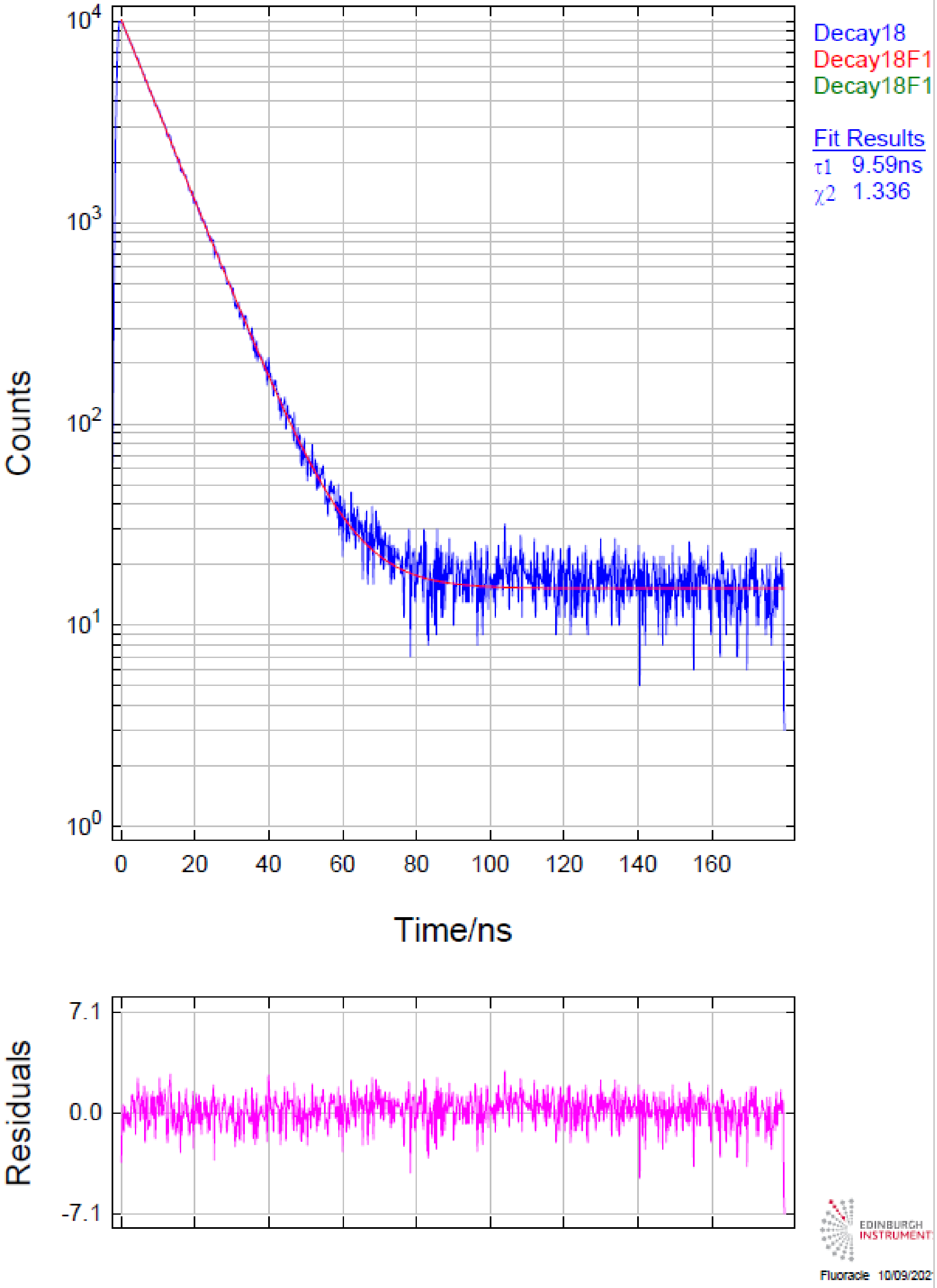

Figure S184. Fluorescence (experimental data in blue, fit in red, residue in pink) of compounds **6n** in acetonitrile.

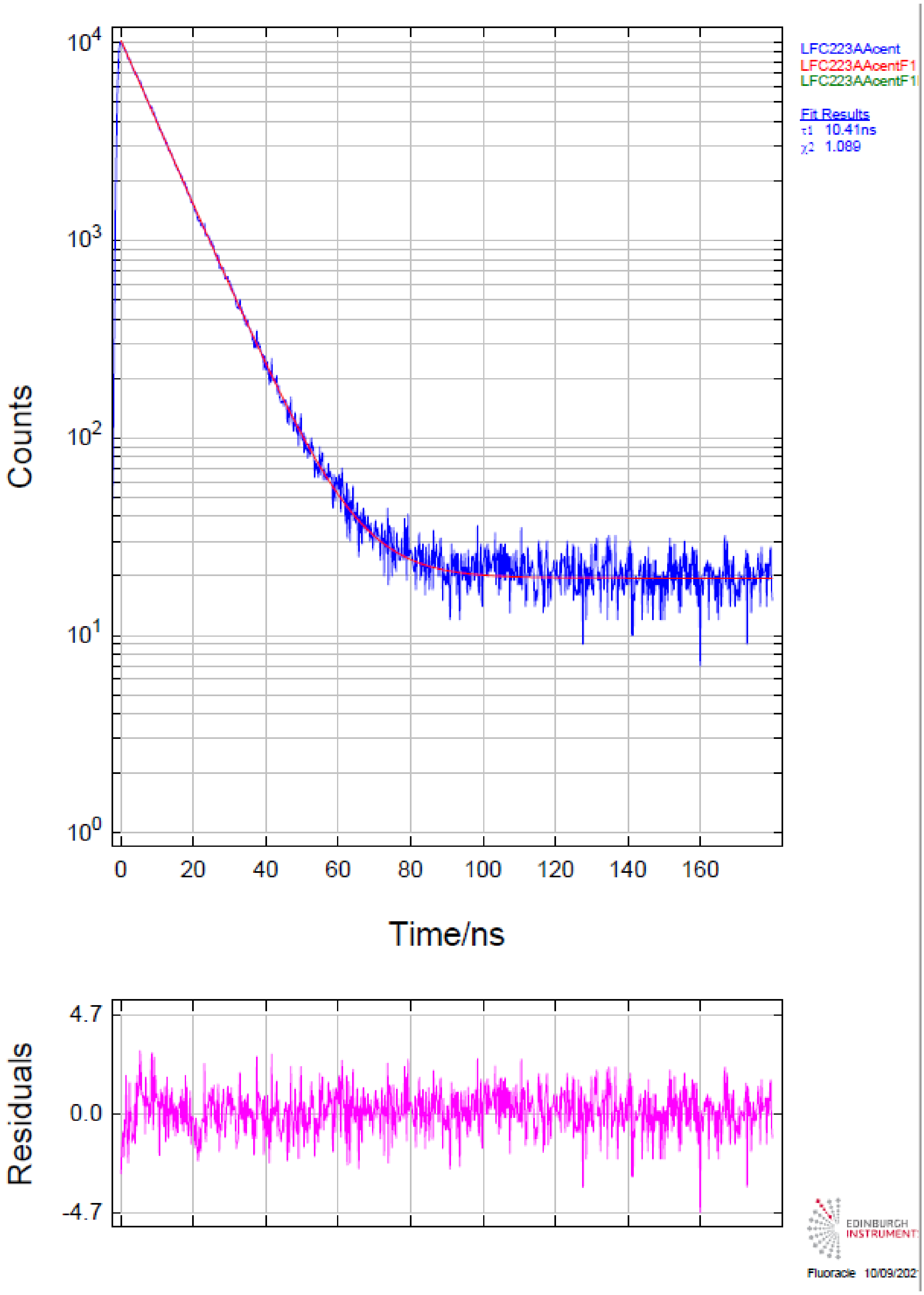

Figure S185. Fluorescence (experimental data in blue, fit in red, residue in pink) of compounds **6o** in acetonitrile.

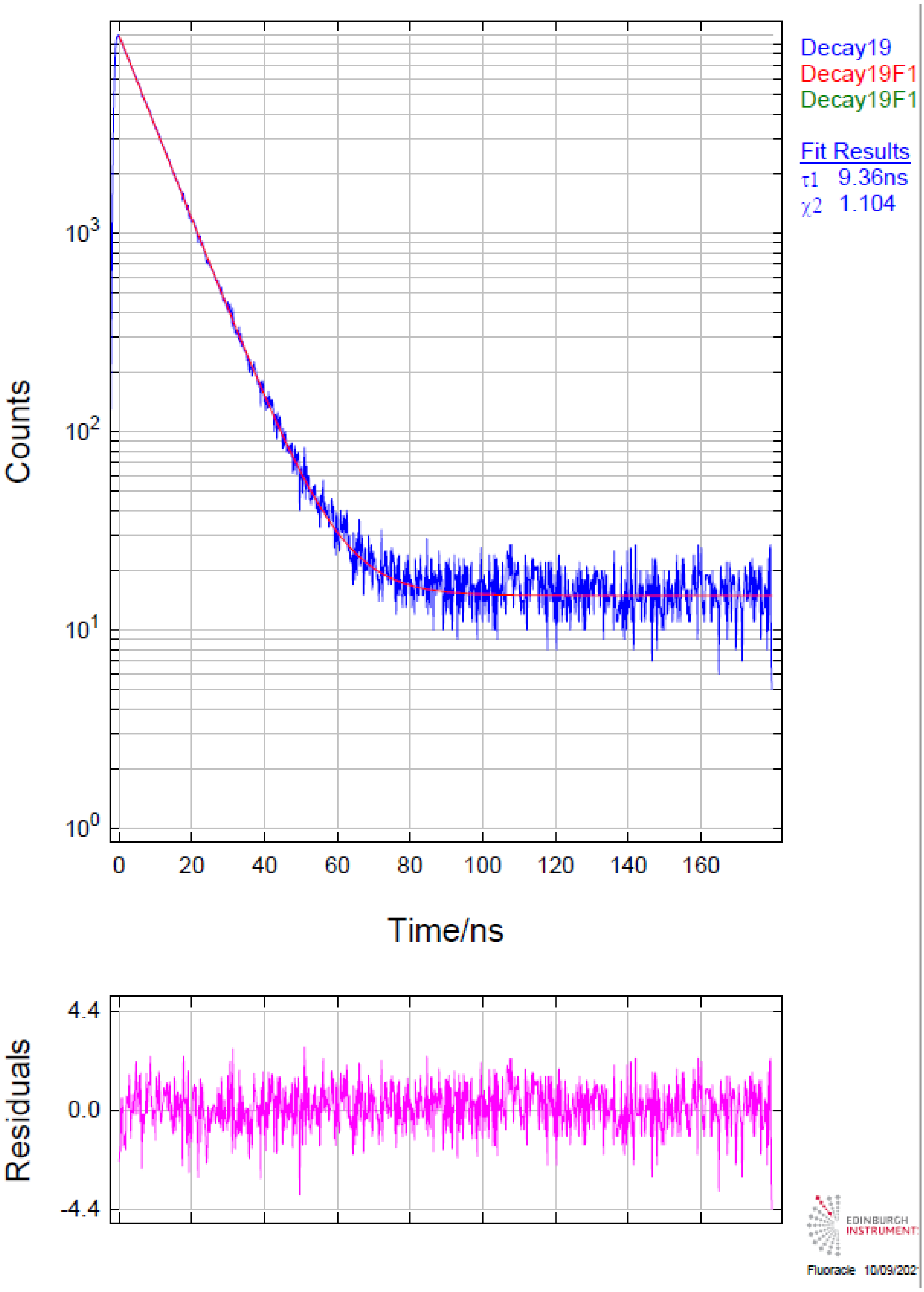

Figure S186. Fluorescence (experimental data in blue, fit in red, residue in pink) of compounds **6p** in acetonitrile.

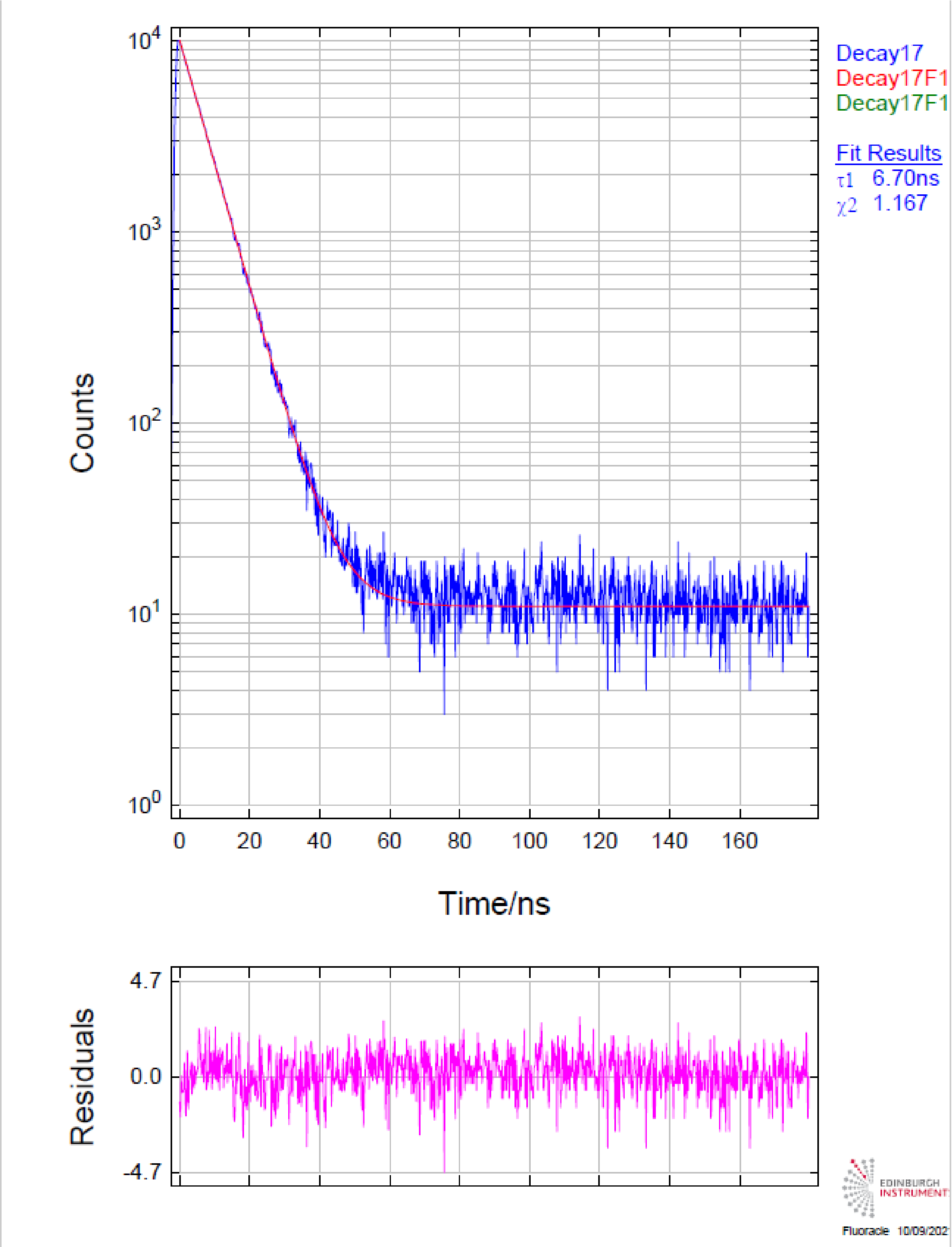

Figure S187. Fluorescence (experimental data in blue, fit in red, residue in pink) of compounds **6q** in acetonitrile.

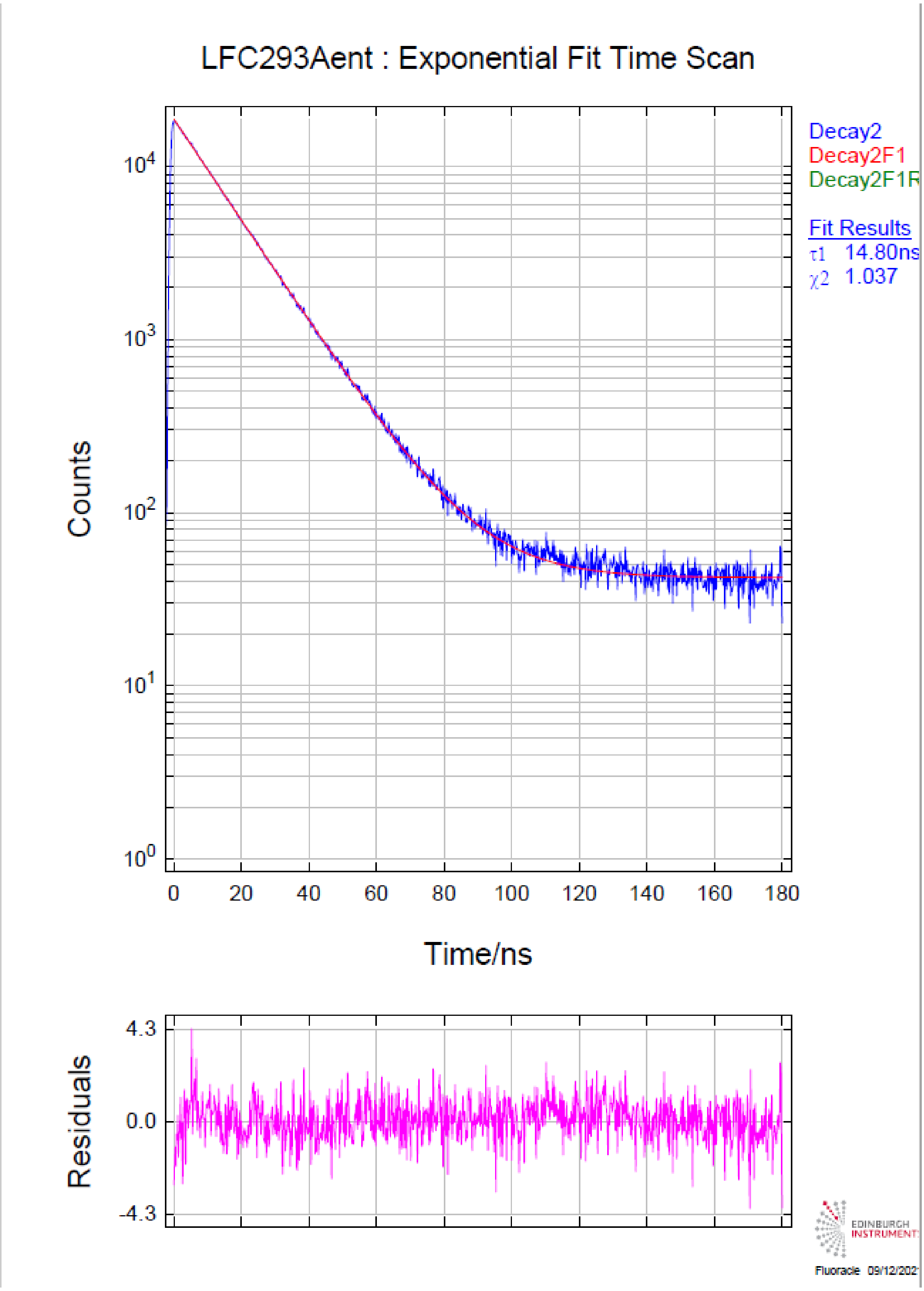

Figure S188. Fluorescence (experimental data in blue, fit in red, residue in pink) of compounds **9** in acetonitrile.

References

1 F. Zinna, T. Bruhn, C. A. Guido, J. Ahrens, M. Bröring, L. Di Bari and G. Pescitelli, *Chem. Eur. J.*, 2016, **22**, 16089-16098.

2 CrysAlisPro 1.171.41.89a (Rigaku Oxford Diffraction, 2020)

3 O. V. Dolomanov, L. J. Bourhis, R. J. Gildea, J. A. K. Howard and H. Puschmann, *J. Appl. Crystallogr.*, 2009, **42**, 339-341.

4 G. M. Sheldrick, *Acta Crystallogr., Sect. C: Struct. Chem.*, 2015, **71**, 3-8.

5 L. J. Farrugia, *J. Appl. Crystallogr.*, 2012, **45**, 849-854.

6 B. Laleu, P. Mobian, C. Herse, B. W. Laursen, G. Hopfgartner, G. Bernardinelli and J. Lacour, *Angew. Chem. Int. Ed.*, 2005, **44**, 1879-1883.
